# An unusual stereoretentive 1,3-quaternary carbon shift resulting in an enantioselective Rh^II^-catalyzed formal [4+1]-cycloaddition between diazo compounds and vinyl ketenes†
†Electronic supplementary information (ESI) available. CCDC 1557298 and 1557297. For ESI and crystallographic data in CIF or other electronic format see DOI: 10.1039/c8sc00020d


**DOI:** 10.1039/c8sc00020d

**Published:** 2018-02-19

**Authors:** Kevin X. Rodriguez, Tara C. Pilato, Brandon L. Ashfeld

**Affiliations:** a Department of Chemistry and Biochemistry , University of Notre Dame , Notre Dame , Indiana 46556 , USA . Email: bashfeld@nd.edu

## Abstract

Enantioselective quaternary carbon construction in the assembly of cyclopentenones employing a Rh^II^-catalyzed, formal [4+1]-cycloaddition is described. A Rh_2_(*S*-TCPTTL)_4_-catalyzed cyclopropanation of a vinyl ketene with a disubstituted diazo compound initiates a stereoretentive, accelerated ring expansion to provide the cycloadduct in good to excellent yields and enantioselectivity.

## Abstract 2

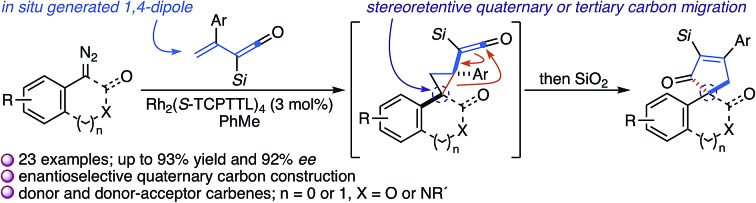
A Rh_2_(*S*-TCPTTL)_4_-catalyzed cyclopropanation of a vinyl ketene with a disubstituted diazo compound initiates a stereorententive, accelerated ring expansion to provide the cycloadduct in good to excellent yields and enantioselectivity.

## Introduction

**Fig. 1:**
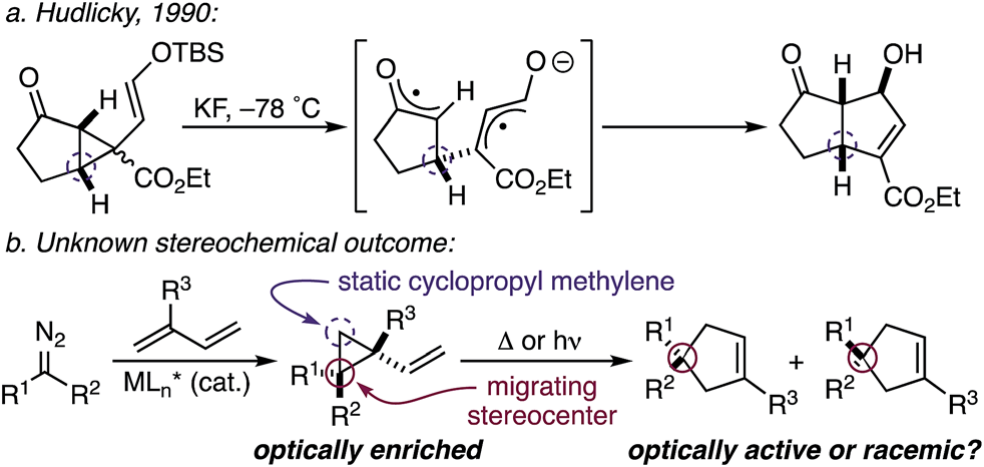
Origin of stereoinduction in vinyl cyclopropane rearrangements.

While the evolution of new enantioselective [4+1]-cycloadditions has advanced considerably in recent years,^1^ enabling the stereoselective polyfunctionalization of a disubstituted C1 subunit to yield quaternary stereogenic carbons remains a significant challenge. The majority of asymmetric [4+1]-cycloadditions rely heavily on the polarization of a compatible 1,3-diene component to provide optically pure heterocycles. For example, in 2007 Fu disclosed an enantioselective Cu^I^-catalyzed [4+1]-cycloaddition of 2,3-dihydrofurans employing enones and diazoesters to control absolute stereochemistry at a tertiary center.^2^ More recently, Shi employed a phosphine-mediated, Morita–Baylis–Hillman-like [4+1]-annulation of electron-deficient alkylidene oxindoles to provide dihydrofurans in high selectivity.^3^ Similarly, the chiral Lewis acid-catalyzed addition of sulfur and nitrogen ylides to electrophilic Michael acceptors (*e.g.*, *ortho*-quinone methides, aza-dienes, *etc.*) has been shown to effectively construct the corresponding 5-membered heterocycle through an overall [4+1]-cycloannulation with good to excellent levels of enantioselectivity.^4^,^5^ However, carbocycle generation requires a complementary 1,3-diene addition that is complicated by a competitive cyclopropanation.^2^,^3^,^5^,^6^ Although a subsequent rearrangement of the resulting vinyl cyclopropane (VCP) yields the formal [4+1]-cycloadduct, the high activation energy and commonly accepted diradical mechanism renders absolute stereocontrol inherently difficult.^7^ Hudlicky and others have shown that optically active cyclopropanes undergo stereoselective migrations, but the static (non-migrating) cyclopropane carbon directs a diastereochemical outcome (Fig. 1a).^8^ However, cyclopropanation of a 2-substituted-1,3-diene places a non-stereogenic methylene at the static position. To the best of our knowledge, chirality transfer in VCP-rearrangements that rely solely on the configurational stability of a *migrating quaternary center* has not been established (Fig. 1b).

**Fig. 2:**
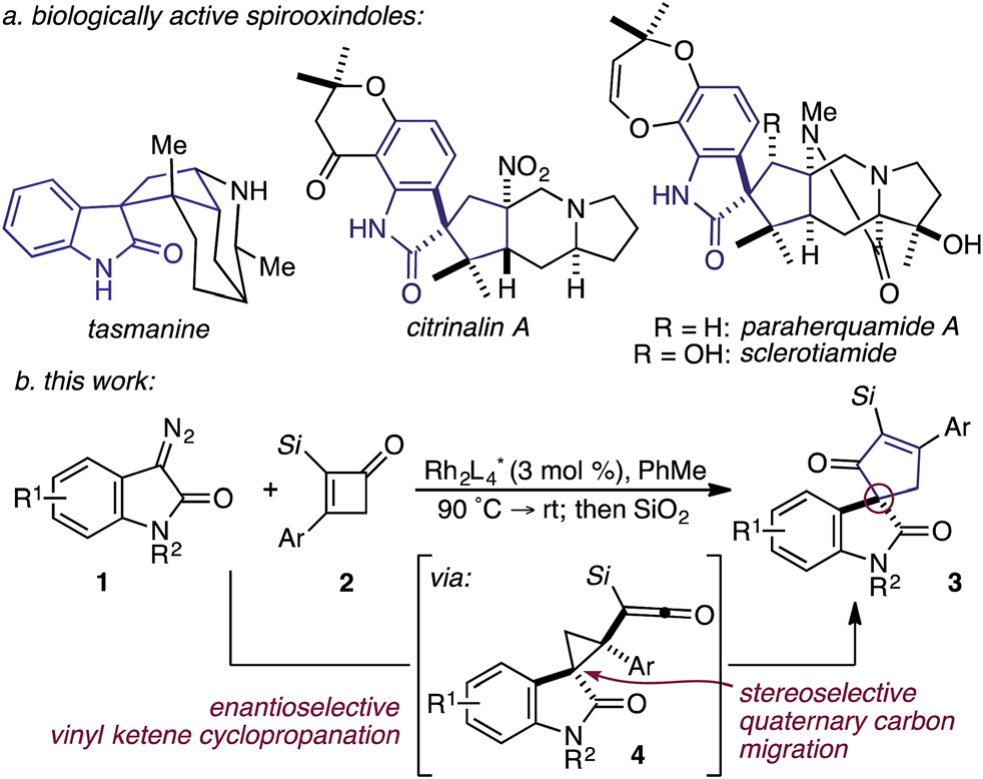
(a) Representative spirooxindole alkaloids; (b) Rh^II^-catalyzed, enantioselective formal [4+1]-cycloaddition.

Based on our previous studies employing vinyl ketenes diazo compounds in [4+1]-cycloadditions,^9^ we speculated that improved orbital alignment between the ketene's orthogonal π-system and that of the migrating C–C bond would lead to greater enantiocontrol throughout the rearrangement.^10^ Motivated by the therapeutic potential of biologically active oxindole natural products, and synthetic challenge that the C3-spirooxindole stereocenter presents, we strategically chose diazooxindoles as our C1-synthon (Fig. 2a).^11^ While diastereoselective strategies are known,11a,11c,^12^ few asymmetric approaches to assemble this quaternary center exist.1b,^13^ Herein, we describe the first enantioselective, formal [4+1]-cycloaddition between a diazo compound **1** and the vinyl ketene generated *in situ* from cyclobutenone **2** to provide cycloadduct **3** involving a stereoselective quaternary carbon migration from cyclopropyl ketene **4** (Fig. 2b).7a,^14^

Despite the preponderance of chiral Rh^II^ cyclopropanation catalysts,^15^ a number of critical issues threatened to derail our efforts prior to undertaking this study. First, examples of diazooxindoles reacting as C1 synthons in asymmetric cycloannulations with high levels of enantioselectivity are rare.1d,^16^ Additionally, the combination of a Lewis basic ketene carbonyl oxygen and electrophilic metallocarbene could complicate the initial cyclopropanation event.^17^ Likewise, we were cognizant that the inherent reactivity of vinyl ketenes to undergo dimerization and other side reactions may lead to unproductive or non-stereoselective pathways.17a,17c–f Based on Danheiser's seminal work with vinyl ketenes,^18^ we opted to generate these relatively underutilized, formal 1,4-dipoles *in situ* from the corresponding α-silyl cyclobutenones.^19^

## Results and discussion

In spite of these potential complications, we began by examining the enantioselective construction of spirooxindole **3a** from diazooxindole **1a** and cyclobutenone **2a** (Table 1).^18^,^19^ While Davies' Rh_2_(*R*-DOSP)_4_ catalyst provided **3a** in 81% yield and 14% *ee* (entry 1), the adamantyl-derived Rh_2_(*R*-PTAD)_4_ improved selectivity to 52% *ee*, albeit with a modest loss in yield (entry 2).^20^ Employing either Rh_2_(*S*-IBAZ)_4_ or Rh_2_(*R*-BTPCP)_4_ resulted in diminished levels of enantioselectivity (entries 3 and 4),^21^ but the tetrachloropthalimide-derived carboxylate Rh_2_(*S*-TCPTTL)_4_ gave **3a** in 95% yield and 72% *ee* (entry 5).^22^ Tetrafluoropthalimide Rh_2_(*S*-TFPTTL)_4_ and naphthaloyl-*tert*-leucine tetracarboxylate Rh_2_(*S*-NTTL)_4_ failed to improve selectivity (entries 6 and 7).^23^

**Table 1:** Optimization of yield and enantioselectivity for **3a**^*a*^

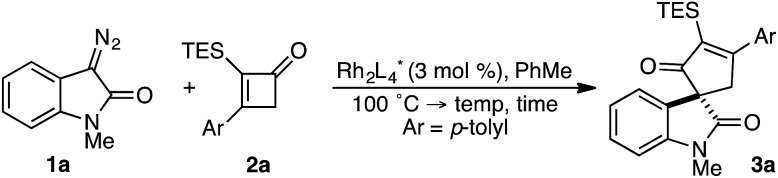					
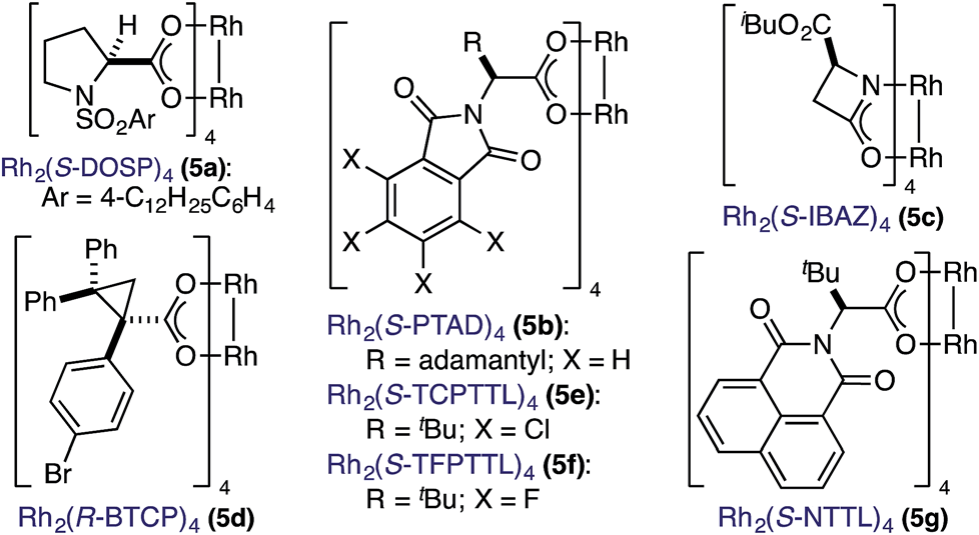					
Entry	**5**	Time (h)	Temp (°C)	Yield (%)	*ee* (%)
1	**5a**	3	100	81	14
2	**5b**	3	100	60	52
3	**5c**	3	100	68	8
4	**5d**	3	90	87	28
*5*	***5e***	*3*	*90*	*95*	*72*
6	**5f**	3	90	91	62
7	**5g**	3	90	81	40
8	**5e**	48	25	32	88
***9***	***5e*** ^*b*^	***30***	***25***	***90***	***90***

^*a*^Conditions: slow addition of **1a** (0.12 mmol) over 1 h to **2a** (0.10 mmol) and **5** (3 mol%) in PhMe (0.1 M). See ESI for detailed experimental procedures.

^*b*^Addition of SiO_2_ (10 mmol) after 2 h.

Lowering the reaction temperature following vinyl ketene formation improved selectivity to 88% *ee*, but the longer reaction times had a negative impact on the yield of **3a** (entry 8). While vinyl ketene cyclopropanation occurred rapidly at 25 °C, a sluggish ring expansion resulted in substantial amounts of undesired side products. Attempts to isolate the cyclopropyl ketene intermediate led to the serendipitous discovery that conversion to the cyclopentenone was accelerated upon exposure to silica gel.8a,^24^ Thus, introduction of SiO_2_ following cyclopropanation yielded cycloadduct **3a** in 90% yield and 90% *ee* (entry 9).^25^ Although speculative at this stage, it would appear that the mild Lewis acidic environment created by the addition of SiO_2_ facilitates the ring expansion event without negatively impacting the chiral integrity of the migrating oxindole C3-stereocenter. A survey of various Lewis acids (*e.g.*, BF_3_·OEt_2_, MgI_2_, Yb(OTf)_3_) failed to provide a marked improvement in the yield of cycloadduct **3a** over SiO_2_. With an optimized set of conditions in hand, we next assessed the extent of chirality transfer in the cyclopropyl ketene rearrangement.

**Scheme 1:**
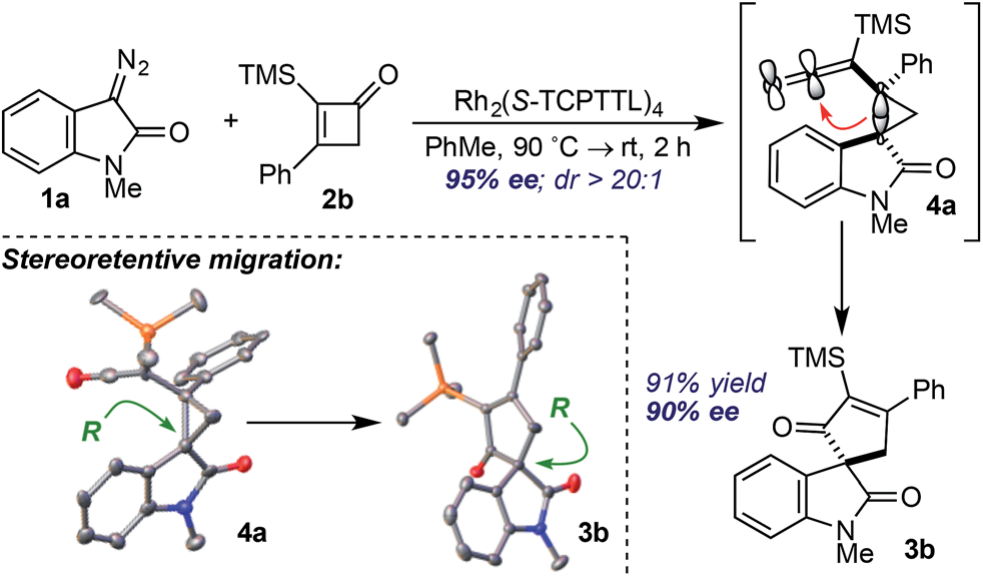
Stereochemical Progression.

Careful monitoring of the formal [4+1]-cycloaddition between **1a** and the vinyl ketene from **2b** in the presence of Rh_2_(TCPTTL)_4_ enabled us to track the stereochemical progression *en route* to cycloadduct **3b** (Scheme 1). After 2 h at room temperature, cyclopropyl ketene **4a** was observed as a single diastereomer in 95% *ee*. Subsequent addition of SiO_2_ converted **4a** to cycloadduct **3b** in 73% yield and 90% *ee*. The modest loss of 5% optical enrichment would indicate a well-defined step-wise or competing step-wise and concerted cyclopropyl ketene rearrangements. In a separate experiment, cooling of the reaction mixture (*ca.* –20 °C) after 2 h led to selective crystallization of **4a**, and X-ray crystal diffraction revealed a *syn* relationship of the oxindole arene and ketene across the cyclopropane and an *R* configuration of the C3-oxindole quaternary center. Comparison of the X-ray crystal structure obtained for isolated **3b** indicated a net stereoretentive migration of the C3-oxindole.^26^ Interestingly, the diastereoselectivity observed in the formation of cyclopropane **4a** is in stark contrast to the major aryl–aryl *syn* diastereomers observed in many Rh^II^-catalyzed cyclopropanations of styrene derivatives and donor–acceptor metallocarbenes derived from aryl diazo esters.15a,^27^ While speculative at this stage, the observed aryl–aryl *anti*-stereoisomer of **4a** may arise due to minimization of eclipsing interactions between the oxindole arene and vinyl ketene *p*-tolyl group.27b,27c

To determine the catalyst influence on the conversion of intermediate **4** to cycloadduct **3**, we conducted a series of control experiments to monitor the formation of **3b** from isolated cyclopropyl ketene **4a** (Table 2). Exposure of racemic **4a** to Rh_2_(TCPTTL)_4_ under our optimized conditions led to a quantitative yield of racemic **3b** (entry 1). Treatment of optically enriched **4a** (94% *ee*) with either Rh_2_(OAc)_4_ or no Rh^II^ catalyst resulted in comparable yield of **3b** with modest loss of optical purity (entries 2 and 3). These results would indicate that the catalyst is not influencing the stereochemical outcome of the cyclopropyl ketene rearrangement.

**Table 2:** Impact of catalyst on rearrangement of **4a**

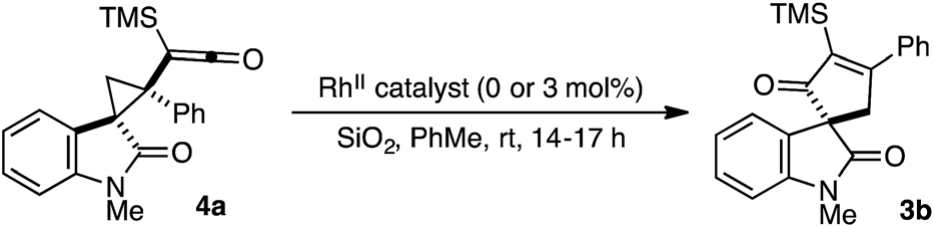				
Entry	% *ee* of **4a**	Catalyst	Yield (%)	% *ee* of **3b**
1	0	Rh_2_(*S*-TCPTTL)_4_	>99	0
2	94	Rh_2_(OAc)_4_	>99	84
3	94	No catalyst	>99	86

Evaluation of the structural diversity across diazooxindole **1** and cyclobutenone **2** in the Rh_2_(TCPTTL)_4_-catalyzed [4+1]-cycloaddition provided the corresponding enantioselectivities (Table 3). While variations of the α-silyl group on **2** did not significantly affect selectivity, yields decreased with increasing size of the vinyl silane (**3c–e**). Additionally, *ortho*-substituents on the phenyl ring resulted in a decrease in both yield and enantioselectivity (**3j**). Various *N*-alkyl, acyl, benzyl, allyl, and propargyl diazooxindoles gave the corresponding cyclopentenones **3l–p** in 79–90% *ee*. Notably, *N*-allyl cyclopropanation in **3n** was not observed. Oxindole arene substitution did not adversely affect the cycloaddition, resulting in stereoselective quaternary carbon assembly in 77–90% *ee* (**3q–u**). It is worth noting that, modest improvements in selectivity were observed by performing the reaction at 4 °C over 48 h for cycloadducts **3m**, **3n**, and **3p–r**. Subsequent recrystallization of **3b** led to further optical enrichment (≥98% *ee*), and the absolute stereochemistry of all substrates was assigned by analogy.

**Table 3:** Structural diversity of the formal [4+1]-cycloannulation^*a*^

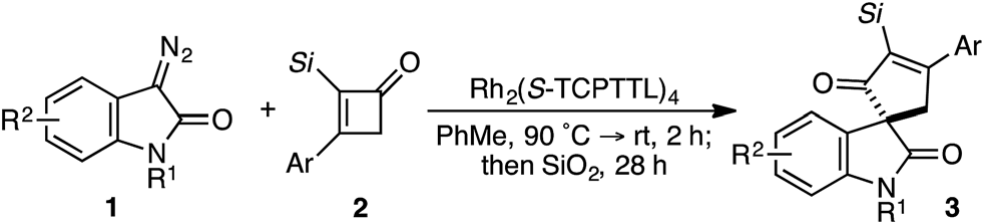
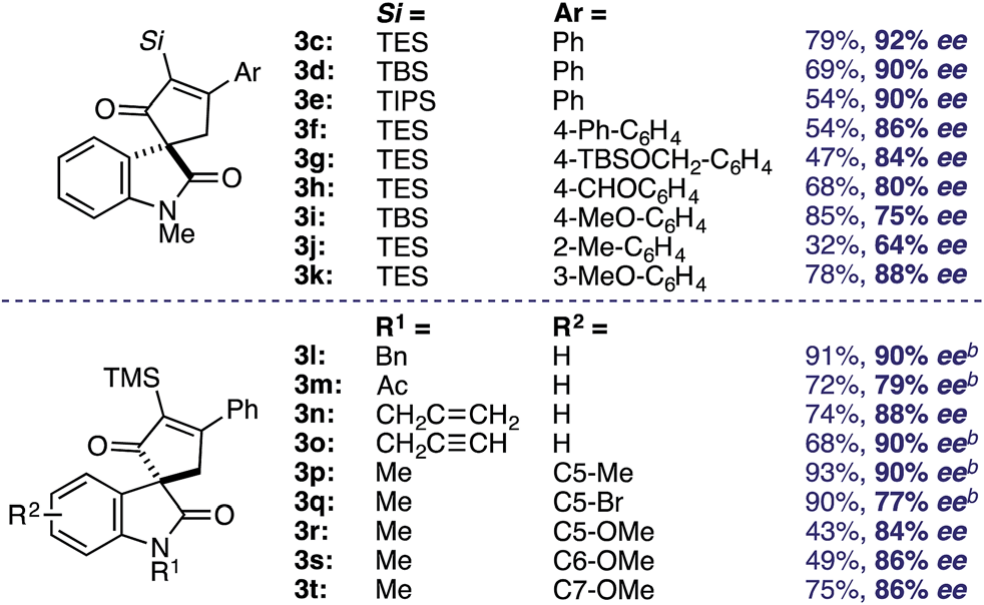

^*a*^Conditions: slow addition of **1** (0.12 mmol) over 1 h to **2b** (0.10 mmol) and Rh_2_(*S*-TCPTTL)_4_ (3 mol%) in PhMe (0.1 M) at 25 °C.

^*b*^Reaction performed at 4 °C for 48 h.

Based on the crown configuration of Rh_2_(TCPTTL)_4_, Charette and co-workers proposed a π–π stacking interaction between the carbene donor aryl ring and the pthalimido groups of the catalyst wall as key to the observe stereoinduction in cyclopropanations.^26^ Speculating that a non-aryl diazo compound would result in reduced enantioinduction, we subjected ethyl diazoacetate (**6a**) to our standard conditions, but failed to observe formation of cyclopentenone **7a** even after prolonged reaction times (Table 4). The stalled migration led to cyclopropane **8a** in a 1.4 : 1 ratio of diastereomers and 0% *ee*. However, employing phenyl diazoester **6b** resulted in a 1 : 2.3 ratio of cycloadduct **7b** and ketene **8b** after 20 h at 60 °C. While adduct **7b** was obtained in 80% *ee*, we isolated cyclopropane **8b** as a single diastereomer in 56% *ee*. Curiously, neither prolonged reaction times (≥48 h) nor the addition of other Lewis acids (*i.e.*, MgI_2_) improved the conversion of either **8a** or **8b** to the corresponding cyclopentenones. Although elevated temperatures failed to provide cycloadduct **7a** or improve the yield of cyclopropane **8a** from diazoester **6a**, conducting the formal [4+1]-cycloaddition of **6b** above 60 °C led to diminished levels of optical enrichment for **7b** while not affecting the amount of each product obtained. In contrast, phenyl diazomethane (**6c**) underwent rapid conversion to cycloadduct **7c** in 63% yield and 82% *ee* from presumptive cyclopropyl ketene **8c**. Likewise, diazochromanone **6d** yielded spirocycle **7d** in 75% yield and 86% *ee* and no cyclopropane **8d**. These composite results would indicate that the presence of an α-aryl substituent is key to enantioselectivity and enabling a facile cyclopropyl ketene rearrangement *en route* to the formal [4+1]-cycloadduct.^28^

**Table 4:** Impact of Diazo Compound^*a*^

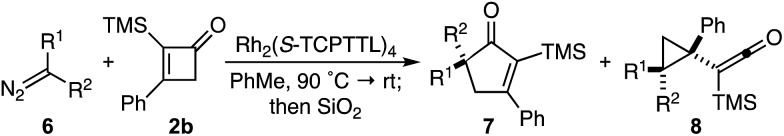	
Diazo **6**	Distribution of 3- *vs.* 5-membered cycloadducts
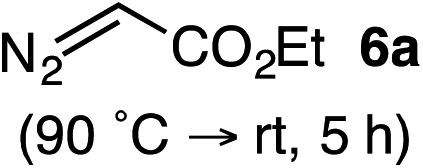	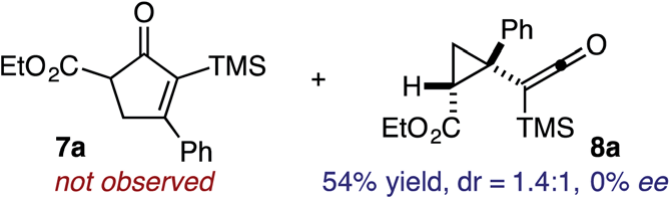
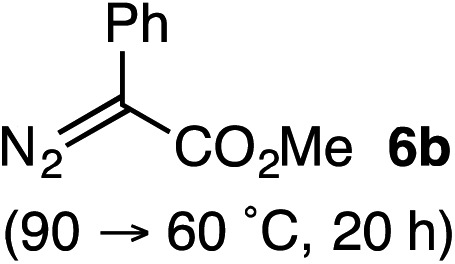	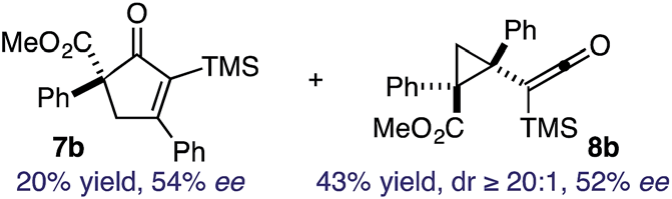
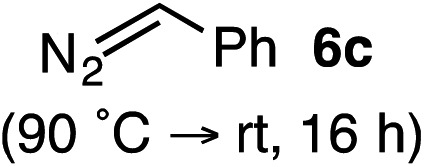	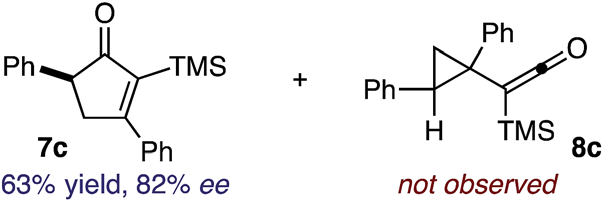
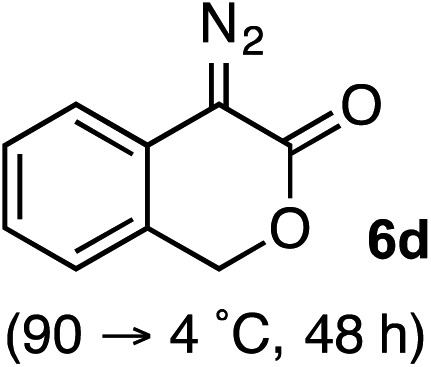	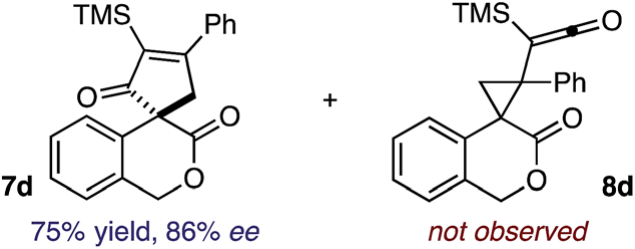

^*a*^Conditions: see ESI for detailed experimental procedures.

**Scheme 2:**
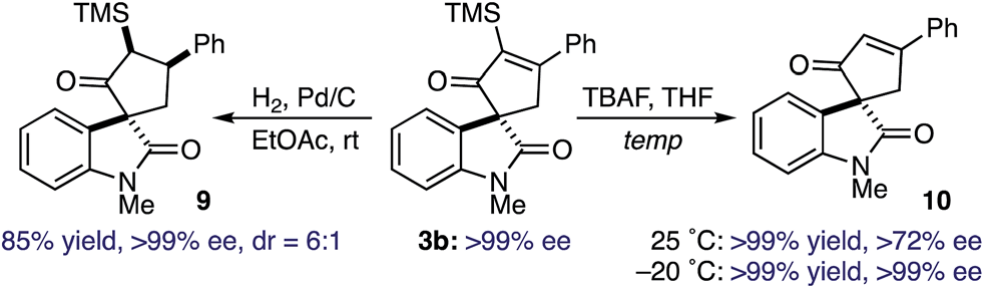
Stereochemical progression.

Subsequent efforts toward functionalizing the spirooxindole cycloadducts focused on evaluating the stability of the quaternary spirocenter. Catalytic hydrogenation of optically enriched spirooxindole **3b** (>99% *ee via* recrystallization) provided spirocyclopentane **9** in 85%, 6 : 1 diastereoselectivity favoring catalyst approach from the C2-oxindole face, and >99% *ee* (Scheme 2). Curiously, protodesilylation of **3b** at room temperature using TBAF proceeded in quantitative yield, but gave enone **10** in 72% *ee*. However, conducting the reaction at –20 °C effectively removed the α-TMS group to provide enone **10** in comparable yield and without loss of optical purity. While stereochemical stability of the C3-quaternary center is a reasonable expectation, the combination of our results and those representative examples in the literature would indicate that the 1,3-dicarbonyl arrangement of the spirocycle in **3** can lead to α-epimerization under selected conditions^29^,^30^

## Conclusions

In summary, we have developed an asymmetric Rh^II^-catalyzed formal [4+1]-cycloaddition between diazo compounds and *in situ* generated vinyl ketenes that leads to the direct stereoselective assembly of quaternary stereogenic carbons. The reaction produces cyclopentenones in good to excellent yields with up to 92% *ee*. A detailed mechanistic study of the cyclopropyl ketene rearrangement, origin of the observed chirality transfer, and applications toward target-directed total synthesis are currently under investigation and will be reported in due course.

## Conflicts of interest

There are no conflicts to declare.

## Supplementary Material

Supplementary information

Crystal structure data
