# Supplementary material for: An unusual stereoretentive 1,3-quaternary carbon shift resulting in an enantioselective RhII-catalyzed formal [4+1]-cycloaddition between diazo compounds and vinyl ketenes
Source: Chem Sci. 2018 Feb 19;9(12):3221–6. doi: 10.1039/c8sc00020d (PMC5931190; doi:10.1039/c8sc00020d)
Supplement: Supplementary file 1 [file SC-009-C8SC00020D-s001.pdf]

Electronic Supplementary Material (ESI) for Chemical Science.
This journal is © The Royal Society of Chemistry 2018

SUPPORTING INFORMATION

FOR

An Unusual Stereoretentive 1,3-Quaternary Carbon Shift Resulting in a Rh^{II}-Catalyzed Enantioselective Formal [4+1]-Cycloaddition Between Diazo Compounds and Vinyl Ketenes

Kevin X. Rodriguez, Tara C. Pilato, and Brandon L. Ashfeld*

Department of Chemistry and Biochemistry, University of Notre Dame
305 McCourtney Hall, Notre Dame, IN 46556

bashfeld@nd.edu

Contents

General	S2
Experimental Procedures	S2-S17
Crystallographic Data for **3b**	S18-S24
Crystallographic Data for **4a**	S25-S31
References	S32
^{1}H and ^{13}C NMR Spectra	S33-S146

1. General

Solvents and reagents were ACS reagent grade and used without further purification unless noted below. Dimethylformamide (DMF), tetrahydrofuran (THF), dichloromethane (CH_2Cl_2) and diethyl ether (Et_2O) were passed through a column of molecular sieves and stored under argon. All reactions were carried out in flame-dried glassware under an argon atmosphere unless otherwise specified. Diazooxindoles **1a-1j**,[1] **6b-6d**,[2] cyclobutenone **2b**,[3] 3-phenyl-2-(triethylsilyl)-2-cyclobutenone,[3] Rh_2(*S*-TCPTTL)$_4$,[4] Rh_2(*S*-TFPTTL)$_4$,[5] Rh_2(*S*-NTTL)$_4$,[6] Rh_2(*S*-IBAZ)$_4$,[7] were prepared according to literature procedures, and spectral data (^1H and ^{13}C NMR) were consistent with those reported.

^1H Nuclear magnetic resonance (NMR) spectra were obtained at 400, 500 or 600 MHz, and ^{13}C NMR spectra at 100, 125 or 150 MHz. Chemical shifts are reported in parts per million (ppm, δ), and referenced to residual solvent or tetramethylsilane (TMS). Coupling constants are reported in Hertz (Hz). Spectral splitting patterns are designated as s, singlet; d, doublet; t, triplet; q, quartet; p, pentet; m, multiplet; comp, complex; app, apparent; hom, higher order multiplet; and br, broad. Infrared (IR) spectra were obtained using a Thermo Electron Nicolet 380 FT-IR using a silicon (Si) crystal in an attenuated total reflectance (ATR) tower and reported as wavenumbers (cm^{-1}). High and Low resolution electrospray ionization (ESI) measurements were made with a Bruker MicroTOF II mass spectrometer. Analytical thin layer chromatography (TLC) was performed using EMD 250 micron 60 F_{254} silica gel plates, visualized with UV light and stained with a *p*-anisaldehyde solution. Flash column chromatography was performed according to Still's procedure (Still, W. C.; Kahn, M.; Mitra, A. *J. Org. Chem.* **1978**, *43*, 2923) using EMD 40-63 μm 60 Å silica gel.

2. Experimental Procedures

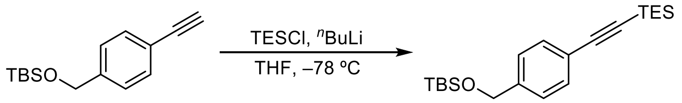

***tert*-Butyldimethyl((4-((triethylsilyl)ethynyl)benzyl)oxy)silane.** A solution of ^{n}BuLi (3.7 mmol, 2.0 M in hexanes, 1.84 mL) was slowly added to a solution of *tert*-butyl((4-ethynylbenzyl)oxy)dimethylsilane[8] (0.76 g, 3.06 mmol) in THF (10 mL) at –78 °C and stirred for 30 min. Chlorotriethylsilane (0.55 g, 3.67 mmol, 0.62 mL) was then added dropwise, allowed to warm to room temperature by removal of the dry ice/acetone bath, and the resulting solution stirred for an additional 2 h. The crude mixture was diluted with H_2O (15 mL), the layers separated, and the aqueous phase extracted with Et_2O (3 x 15 mL). The combined organic extracts were washed sequentially with saturated aqueous $NaHCO_3$ (3 x 15 mL) and saturated aqueous NaCl (3 x 15 mL), dried (Na_2SO_4) and concentrated under reduced pressure. The resulting crude mixture was purified by flash chromatography eluting with hexanes/EtOAc (40:1) to provide 1.07 g (97%) of the title compound as a clear, colorless oil. ^1H NMR (500 MHz, $CDCl_3$) δ 7.44 (d, J = 8.4 Hz, 2 H), 7.24 (d, J = 8.4 Hz, 2 H), 4.73 (s, 2 H), 1.05 (t, J = 8.0 Hz, 9 H), 0.93 (s, 9 H), 0.67 (q, J = 8.0 Hz, 6 H), 0.09 (s, 6 H); ^{13}C NMR (125 MHz, $CDCl_3$) δ 142.0, 132.1, 125.9, 121.9, 106.6, 91.2, 64.8, 26.1, 7.6, 4.6, 3.5, -5.1; IR (neat) 2955, 2874, 2156, 1506 cm^{-1}; HRMS (ESI) *m/z* 361.2368 [$C_{21}H_{37}OSi_2$(M+H) requires 361.2377].

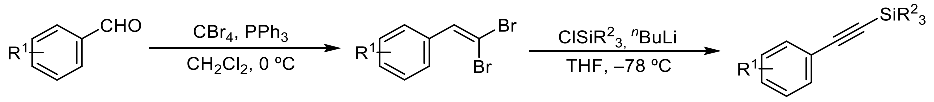

General procedure for the synthesis of silyl acetylenes: A solution of aryl aldehyde (15 mmol) was added to a solution of CBr_4 (5.64 g, 17 mmol) in CH_2Cl_2 (25 mL) at 0 °C and the mixture was stirred for 5 min. A solution of PPh_3 (8.39 g, 32 mmol) in CH_2Cl_2 (25 mL) was then added dropwise over 10 min,

the reaction was allowed to warm to room temperature by removal of the ice bath, and stirred for an additional 5 h. The resulting heterogeneous mixture reaction was filtered through a pad of celite eluting with hexanes (40 mL) and the filtrate was concentrated under reduced pressure. The resulting crude mixture was purified by flash chromatography eluting with hexanes/EtOAc (4:1) to provide the target vinyl dibromide.

A solution of dibromoalkene (13 mmol) in THF (40 mL) was cooled to –78 °C, a solution of ^{n}BuLi (31 mmol, 1.97 M in hexanes, 16 mL) was added dropwise, and the mixture stirred for 45 min. The corresponding trialkyl chlorosilane silane (16 mmol) was added, stirring continued for an additional 30 min, and then allowed to warm to room temperature by removal of the dry-ice/acetone bath. The resulting solution was diluted with H_2O (50 mL), the layers separated, and the aqueous phase extracted with Et_2O (2 x 30 mL). The combined organic extracts were washed sequentially with saturated aqueous $NaHCO_3$ (1 x 30 mL) and saturated aqueous NaCl (1 x 30 mL), dried (Na_2SO_4) and concentrated under reduced pressure. The crude mixture was purified by flash chromatography eluting with hexanes/EtOAc at the indicated ratio (20:1-50:1) to provide the title silyl alkyne.

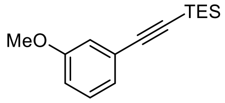

Triethyl((4-methoxyphenyl)ethynyl)silane. The alkynylation of 3-methoxybenzaldehyde was conducted on a 15 mmol scale. Purification was performed by flash chromatography eluting with hexanes/EtOAc (30:1) to afford 4.01 g (91%) of the title compound as a clear, colorless oil. ^{1}H NMR (500 MHz, $CDCl_3$) δ 7.21 (t, J = 7.8 Hz, 1 H), 7.08 (td, J = 7.6, 1.2 Hz, 1 H), 7.00 (dd, J = 2.5, 1.4 Hz, 1 H), 6.87 (ddd, J = 8.4, 2.5, 1.4 Hz, 1 H), 3.81 (s, 3 H), 1.06 (t, J = 7.9 Hz, 9 H), 0.68 (q, J = 7.9 Hz, 6 H); ^{13}C NMR (125 MHz, $CDCl_3$) δ 129.5, 124.9, 116.9, 115.3, 106.5, 91.7, 55.5, 7.7, 4.6; IR (neat) 2954, 2874, 2153, 1576 cm^{-1}; HRMS (ESI) *m/z* 247.1543 [$C_{15}H_{23}OSi$(M+H) requires 247.1512].

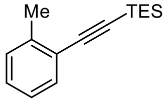

Triethyl(*o*-tolylethynyl)silane. The alkynylation of 2-methylbenzaldehyde was conducted on a 8.0 mmol scale. Purification was performed by flash chromatography eluting with hexanes/EtOAc (30:1) to afford 1.73 g (94%) of the title compound as a clear, colorless oil. ^{1}H NMR (500 MHz, $CDCl_3$) δ 7.45 (d, J = 7.6 Hz, 1 H), 7.23-7.19 (m, 2 H), 7.14-7.0 (m, 1 H), 2.46 (s, 3 H), 1.07 (t, J = 8.0 Hz, 9 H), 0.69 (q, J = 8.0 Hz, 6 H); ^{13}C NMR (125 MHz, $CDCl_3$) δ 140.7, 132.4, 129.5, 128.5, 125.5, 123.3, 105.3, 20.9, 7.7, 4.7; IR (neat) 2955, 2874, 2153, 2090 cm^{-1}; HRMS (ESI) *m/z* 231.1541 [$C_{15}H_{23}Si$(M+H) requires 231.1563].

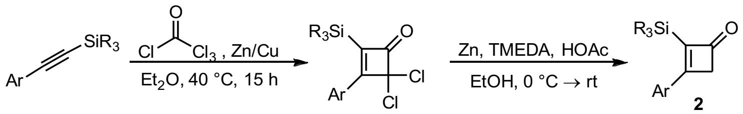

General procedure for α-trialkylsilylcyclobutenone 2 synthesis:[3] A solution of trichloroacetylchloride (3.64 g, 20 mmol, 2.2 mL) in Et_2O (50 mL) was added slowly to a refluxing solution of silyl alkyne (17 mmol) and Zn/Cu (3.8 g, 60 mmol) in Et_2O (35 mL) over 3 h and then stirred for an additional 15 h. The mixture was cooled to room temperature by removal of the oil bath and the heterogeneous mixture filtered through a pad of celite eluting with Et_2O (50 mL). The filtrate was washed sequentially with saturated aqueous $NaHCO_3$ (3 x 100 mL), H_2O (100 mL), and saturated aqueous NaCl (30 mL), dried (Na_2SO_4) and concentrated under reduced pressure. The resulting crude 4,4-dichlorocyclobutenone (5 mmol) was dissolved in EtOH (17 mL) and added slowly to a mixture of zinc

(1.9 g, 29 mmol), *N*,*N*,*N*′,*N*′-tetramethylethylenediamine (3.37 g, 29 mmol, 4.3 mL) and acetic acid (1.74 g, 29 mmol, 1.7 mL) in EtOH (25 mL) at 0 ˚C over 20 min. The mixture was allowed to warm up to room temperature by removal of the ice bath, stirred for 3 h, then diluted with 1:1 hexanes/Et_2O (20 mL) and filtered through a pad of celite. The filtrate was washed sequentially with saturated aqueous $NaHCO_3$ (3 x 50 mL), H_2O (50 mL) and saturated aqueous NaCl (1 x 50 mL), dried (Na_2SO_4) and concentrated under reduced pressure [note: rotary evaporator bath temperature not to exceed 40 ˚C]. The crude mixture was purified by flash chromatography eluting with hexanes/EtOAc at the indicated ratio (8:1-50:1) to provide the title α-silylcyclobutenone **5**. [Note: the cyclobutenones **5** were stored in a 4 ˚C refrigerator until needed (~1-2 weeks). Prolonged storage required re-purification prior to use.]

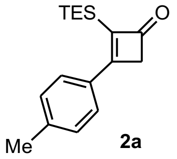

2a

3-(*p*-Tolyl)-2-(triethylsilyl)cyclobut-2-en-1-one (2a). The cycloaddition of triethyl(*p*-tolylethynyl)silane[9] was conducted on a 2.6 mmol scale. Purification was performed by flash chromatography eluting with hexanes/EtOAc (50:1 to 40:1) to provide 270 mg (38%) of **2a** as a yellow oil. ^{1}H NMR (600 MHz, $CDCl_3$) δ 7.52 (d, *J* = 7.8 Hz, 2 H), 7.30 (d, *J* = 7.8 Hz, 2 H), 3.70 (s, 2 H), 2.43 (s, 3 H), 0.96 (t, *J* = 8.4 Hz, 9 H), 0.84 (q, *J* = 8.4 Hz, 6 H); ^{13}C NMR (150 MHz, $CDCl_3$) δ 190.5, 184.5, 156.2, 132.6, 128.2, 127.4, 127.3, 55.7, 19.9, 7.2, 2.6; IR (neat) 2954, 2875, 1742, 1678, 1598, 1509, 1284 cm^{-1}; HRMS (ESI) *m/z* 273.1659 [$C_{17}H_{25}OSi$(M+H) requires 273.1669].

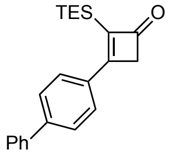

3-([1,1′-Biphenyl]-4-yl)-2-(triethylsilyl)cyclobut-2-en-1-one. The cycloaddition of ([1,1'-biphenyl]-4-ylethynyl)triethylsilane[10] was conducted on a 2.1 mmol scale. Purification was performed by flash chromatography eluting with hexanes/EtOAc (30:1) to provide 189 mg (27%) of the title compound as a yellow oil. ^{1}H NMR (600 MHz, $CDCl_3$) δ 7.74 (d, *J* = 8.3 Hz, 2 H), 7.69 (d, *J* = 8.3 Hz, 2 H), 7.66 (d, *J* = 8.5 Hz, 2 H), 7.48 (t, *J* = 7.4 Hz, 2 H), 7.41 (t, *J* = 7.4 Hz, 1 H) 0.99 (t, *J* = 8.1 Hz, 9 H), 0.87 (q, *J* = 8.1 Hz, 6 H); ^{13}C NMR (150 MHz, $CDCl_3$) δ 191.6, 177.5, 147.0, 144.1, 139.8, 132.4, 129.6, 129.0, 128.2, 127.4, 52.1, 7.5, 3.4; IR (neat) 2953, 2083, 1731 cm^{-1}; HRMS (ESI) *m/z* 335.1813 [$C_{22}H_{27}OSi$(M+H) requires 335.1825].

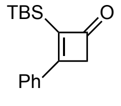

2-(*tert*-Butyldimethylsilyl)-3-phenylcyclobut-2-en-1-one. The cycloaddition of *tert*-butyldimethyl(*p*-tolylethynyl)silane[11] was conducted on a 3.2 mmol scale. Purification was performed by flash chromatography eluting with hexanes/EtOAc (30:1) to provide 181 mg (22%) of the title compound as a yellow oil. ^{1}H NMR (500 MHz, $CDCl_3$) δ 7.67-7.65 (m, 2 H), 7.49-7.48 (m, 3 H), 3.73 (s, 2 H), 0.97 (s, 9 H), 0.29 (s, 6 H); ^{13}C NMR (150 MHz, $CDCl_3$) δ 191.5, 178.0, 147.8, 133.6, 131.6, 129.3, 128.8, 52.4, 26.8, 18.0, -4.8; IR (neat) 2954, 2857, 1752, 1681, 1591, 1471, 1252 cm^{-1}; HRMS (ESI) *m/z* 259.1518 [$C_{16}H_{23}OSi$(M+H) requires 259.1513].

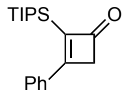

3-Phenyl-2-(triisopropylsilyl)cyclobut-2-en-1-one. The cycloaddition of triisopropyl(phenylethynyl)silane[11] was conducted on 3.3 mmol scale. Purification was performed by flash chromatography eluting with hexanes/EtOAc (30:1) to provide 626 mg (63%) of the title compound as a yellow oil. ^{1}H NMR (600 MHz, $CDCl_3$) δ 7.67-7.65 (m, 2 H), 7.49-7.48 (m, 3 H), 3.76 (s, 2 H), 1.49 (sep, J = 7.7 Hz, 3 H), 1.09 (d, J = 7.7 Hz, 18 H); ^{13}C NMR (150 MHz, $CDCl_3$) δ 192.1, 179.3, 146.9, 134.0, 131.6, 129.0, 128.9, 52.6, 19.0, 12.2; IR (neat) 2943, 2865, 2080, 1741 cm^{-1}; HRMS (ESI) *m/z* 301.1964 [$C_{19}H_{29}OSi$(M+H) requires 301.1982].

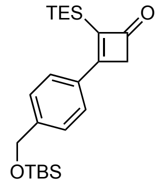

3-(4-(((*tert*-Butyldimethylsilyl)oxy)methyl)phenyl)-2-(triethylsilyl)cyclobut-2-en-1-one. The cycloaddition of *tert*-butyldimethyl((4-((triethylsilyl)ethynyl)benzyl)oxy)silane was conducted on a 1.70 mmol scale. Purification was performed by flash chromatography eluting with hexanes/EtOAc (25:1) to provide 164 mg (24%) of the title compound as a yellow oil. ^{1}H NMR (600 MHz, $CDCl_3$) δ 7.59 (d, J = 7.8 Hz, 2 H), 7.45 (d, J = 7.8 Hz, 2 H), 4.81 (s, 2 H), 3.72 (s, 2 H), 0.98-0.94 (m, 20 H), 0.86 (t, J = 8.4 Hz, 6 H), 0.13 (s, 6 H); ^{13}C NMR (150 MHz, $CDCl_3$) δ 191.9, 178.1, 146.6, 145.6, 132.4, 129.3, 126.2, 64.7, 52.2, 26.1, 18.6, 7.64, 3.53, -5.1; IR (neat) 2955, 2877, 1737, 1703, 1605, 1547, 1414 cm^{-1}; HRMS (ESI) *m/z* 403.2488 [$C_{23}H_{39}O_2Si_2$(M+H) requires 403.2483].

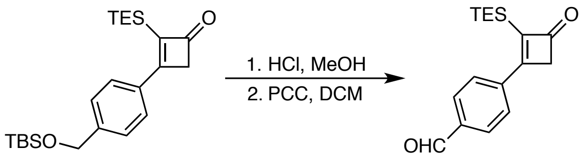

4-(3-Oxo-2-(triethylsilyl)cyclobut-1-en-1-yl)benzaldehyde. Concentrated HCl (37% in H_2O, 54 μL) was added to a solution of 3-(4-(((*tert*-Butyldimethylsilyl)oxy)methyl)phenyl)-2-(triethylsilyl)cyclobut-2-en-1-one (125 mg, 0.27 mmol) in MeOH (5.4 mL) at 0 ˚C. The solution was allowed to warm to room temperature by removal of the ice bath, stirred for 2 h, then diluted with Et_2O (5 mL). The solution was neutralized to pH = 7 with saturated aqueous $NaHCO_3$ (0.4 mL), the layers were separated and the aqueous phase extracted with Et_2O (3 x 5 mL). The combined organic fractions were washed with saturated aqueous NaCl (1 x 10 mL), dried (Na_2SO_4) and concentrated under reduced pressure [note: rotary evaporator bath temperature not to exceed 40 ˚C]. The resulting crude mixture was reconstituted in CH_2Cl_2 (2.7 mL) followed by the addition of pyridinium chlorochromate (172 mg, 1.2 mmol) and silica gel (100 mg) in one portion each. The heterogeneous mixture was stirred at room temperature for 1 h, filtered through a pad of celite eluting with CH_2Cl_2 (10 mL), then concentrated under reduced pressure. The crude mixture was purified by flash chromatography eluting with hexanes/EtOAc (20:1 to 15:1) to afford 25 mg (65%) of the title compound as a yellow oil. ^{1}H NMR (500 MHz, $CDCl_3$) δ 10.09 (s, 1 H), 8.00 (d, J = 8.5 Hz, 2 H), 7.76 (d, J = 8.5 Hz, 2 H), 3.80 (s, 2 H), 0.95 (t, J = 8 Hz, 9 H), 0.86 (q, J = 8 Hz, 6 H); ^{13}C NMR (125 MHz, $CDCl_3$) δ 191.4, 176.3, 151.5, 137.6, 130.0, 129.4, 127.7, 52.6, 7.6, 3.5; IR (neat) 2955, 2875, 2085, 1742, 1703, 1604, 1546 cm^{-1}; HRMS (ESI) *m/z* 287.1459 [$C_{17}H_{23}O_2Si$(M+H) requires 287.1461].

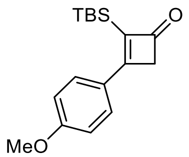

2-(*tert*-Butyldimethylsilyl)-3-(4-methoxyphenyl)cyclobut-2-en-1-one. The cycloaddition of *tert*-butyl((4-methoxyphenyl)ethynyl)dimethylsilane[12] was conducted on a 1.93 mmol scale. Purification was performed by flash chromatography eluting with hexanes/EtOAc (20:1) to provide 228 mg (41%) of the title compound as a yellow oil. ^{1}H NMR (500 MHz, $CDCl_3$) δ 7.62 (d, J = 8.9 Hz, 2 H), 6.98 (d, J = 8.9 Hz, 2 H), 3.89 (s, 3 H), 3.69 (s, 2 H), 0.97 (s, 9 H), 0.29 (s, 3 H), 0.0 (s, 6 H); ^{13}C NMR (100 MHz, $CDCl_3$) δ 191.4, 177.1, 162.3, 144.4, 131.4, 126.3, 114.1, 55.5, 52.0, 26.7, 18.0, -4.9; IR (neat) 2948, 1740, 1682, 1510 cm^{-1}; HRMS (ESI) *m/z* 289.1616 [$C_{17}H_{25}O_2Si$(M+H) requires 289.1618].

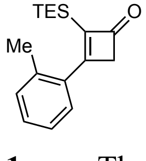

3-(*o*-Tolyl)-2-(triethylsilyl)cyclobut-2-en-1-one. The cycloaddition of triethyl(*o*-tolylethynyl)silane was conducted on a 2.13 mmol scale. Purification was performed by flash chromatography eluting with hexanes/EtOAc (20:1) to provide 157 mg (27%) of the title compound as a yellow oil. ^{1}H NMR (500 MHz, $CDCl_3$) δ 7.34-7.30 (m, 2 H), 7.24 (t, J = 7.1 Hz, 2 H), 3.79 (s, 2 H), 2.41 (s, 3 H), 0.89 (q, J = 8.1 Hz, 9 H), 0.66 (t, J = 8.1 Hz, 6 H); ^{13}C NMR (100 MHz, $CDCl_3$) δ 191.8, 182.9, 153.0, 135.6, 135.2, 130.9, 128.2, 125.8, 55.8, 20.9, 7.4, 3.3; IR (neat) 2950, 2831, 2083, 1741, 1580, 1548, cm^{-1}; HRMS (ESI) *m/z* 273.1654 [$C_{17}H_{25}OSi$(M+H) requires 273.1669].

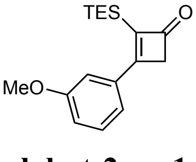

3-(3-Methoxyphenyl)-2-(triethylsilyl)cyclobut-2-en-1-one. The cycloaddition of triethyl((4-methoxyphenyl)ethynyl)silane was conducted on a 2.0 mmol scale. Purification was performed by flash chromatography eluting with hexanes/EtOAc (10:1) to provide 190 mg (33%) of the title compound as a yellow oil. ^{1}H NMR (500 MHz, $CDCl_3$) δ 7.41 (t, J = 8.0 Hz, 1 H), 7.19 (dq, J = 2.6, 1.7 Hz, 1 H), 7.13 (dd, J = 2.5, 1.7 Hz, 1 H), 7.03 (dd, J = 2.5, 1.0 Hz, 1 H), 3.86 (s, 3 H), 3.71 (s, 2 H), 0.95 (t, J = 7.5 Hz, 9 H), 0.85 (q, J = 7.5 Hz, 6 H); ^{13}C NMR (125 MHz, $CDCl_3$) δ 191.9, 178.3, 159.9, 147.6, 135.1, 130.0, 122.0, 117.7, 113.9, 55.7, 52.4, 7.7, 3.6; IR (neat) 2953, 2874, 2083, 1742, 1545, 1230 cm^{-1}; HRMS (ESI) *m/z* 289.1604 [$C_{17}H_{25}O_2Si$(M+1) requires 289.1618].

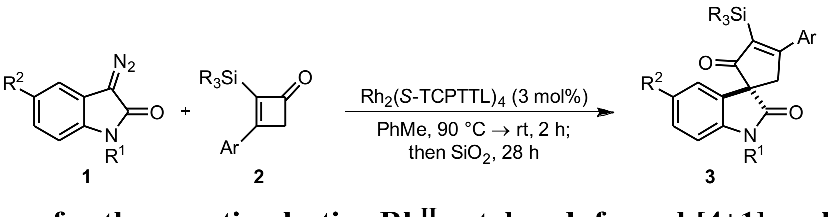

General procedure for the enantioselective Rh^{II}-catalyzed, formal [4+1]-cycloaddition of diazo oxindole 1 and cyclobutenone 2: A solution of **2** (0.1 mmol) and Rh_2(*S*-TCPTTL)$_4$ (5.4 mg, 3.0 μmol) in PhMe (0.33 mL) was stirred at 90 °C for 20 min then cooled to the indicated temperature. A solution of **1** (0.12 mmol) in PhMe (0.67 mL) was added slowly over 1 h, stirred for an additional 2 h, then SiO_2 (1 mmol) was added and stirred for the indicated time. The reaction mixture was concentrated under reduced

pressure and the crude residue purified by flash chromatography eluting with hexanes/EtOAc at the indicated ratio (2:1-10:1) to provide the title spirooxindole cyclopentenone **3**.

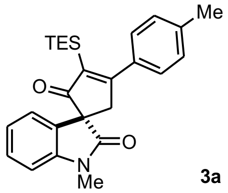
3a

(*R*)-1'-methyl-4-(*p*-tolyl)-3-(triethylsilyl)spiro[cyclopentane-1,3'-indolin]-3-ene-2,2'-dione (3a). The cycloaddition of **1a** and **2a** was performed on 0.08 mmol scale at room temperature for 28 h. Purification was performed by flash chromatography eluting with hexanes/EtOAc (6:1) to provide 30 mg (90%) of **3a** in 90% *ee* as a pink solid. ^{1}H NMR (600 MHz, $CDCl_3$) δ 7.32 (d, *J* = 7.8 Hz, 2 H), 7.30 (td, *J* = 7.2, 1.8 Hz, 1 H), 7.25 (d, *J* = 7.8 Hz, 2 H), 7.04-7.00 (m, 2 H), 6.88 (d, *J* = 7.8 Hz, 1 H), 3.59 (d, *J* = 18.6 Hz, 1 H), 3.26 (s, 3 H), 3.16 (d, *J* = 18.6 Hz, 1 H), 2.43 (s, 3 H), 0.80 (t, *J* = 7.8 Hz, 9 H), 0.58 (q, *J* = 7.8 Hz, 6 H); ^{13}C NMR (150 MHz, $CDCl_3$) δ 206.1, 186.9, 175.1, 144.9, 140.1, 137.5, 135.6, 130.5, 129.1, 128.7, 127.0, 122.9, 121.5, 108.7, 63.1, 46.2, 26.8, 21.6, 7.5, 3.6; IR (neat) 2954, 2875, 1718, 1694, 1612, 1470, 1167 cm^{-1}; HRMS (ESI) *m/z* 418.2221 [$C_{26}H_{32}NO_2Si$(M+H) requires 418.2196]; m.p. = 97-100 ˚C; Chiralpak AD, 25 cm, 97:3 hexanes/^{i}PrOH, 0.5 mL/min, 25 ˚C, 13 bar, t_r (major) = 21.3 min, t_r (minor) = 16.3 min. $[\alpha]_D^{20}$ +81.6 (c 1.00, $CHCl_3$).

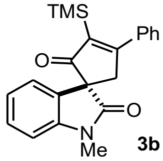
3b

(*R*)-1'-Methyl-4-phenyl-3-(trimethylsilyl)spiro[cyclopentane-1,3'-indolin]-3-ene-2,2'-dione (3b). The cycloaddition of **1a** and **2b** was performed on 0.08 mmol scale at room temperature for 28 h. Purification was performed by flash chromatography eluting with hexanes/EtOAc (5:1) to provide 26 mg (91%) of **3b** in 90% *ee* as a red solid. Recrystallization from $CHCl_3$/pentanes provided **3b** in 98% *ee*. ^{1}H NMR (500 MHz, $CDCl_3$) δ 7.47-7.42 (m, 3 H), 7.42-40 (m, 2 H), 7.32-7.29 (m, 1 H), 7.05-7.03 (m, 2 H), 6.89 (d, *J* = 7.9 Hz, 1 H), 3.60 (d, *J* = 19 Hz, 1 H), 3.27 (s, 3 H), 3.17 (d, *J* = 19 Hz, 1 H), 0.05 (s, 9 H); ^{13}C NMR (125 MHz, $CDCl_3$) δ 205.6, 185.3, 175.0, 144.9, 140.2, 138.3, 130.4, 129.9, 128.8, 128.5, 127.2, 123.0, 121.5, 108.7, 63.1, 45.9, 26.8, -0.6; IR (neat) 2954, 1715, 1693, 1609, 1171, cm^{-1}; HRMS (ESI) *m/z* 384.1378 [$C_{22}H_{23}NO_2Si$(M+Na) requires 384.1390]; m.p. = 185-188 ˚C. Chiralpak AD, 25 cm, 97:3 hexanes/^{i}PrOH, 0.5 mL/min, 25 ˚C, 13 bar, t_r (major) = 34.4 min, t_r (minor) = 19.3 min. $[\alpha]_D^{20}$ +121.4 (c 1.00, $CHCl_3$).

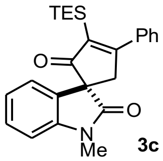
3c

(*R*)-1'-Methyl-4-phenyl-3-(triethylsilyl)spiro[cyclopentane-1,3'-indolin]-3-ene-2,2'-dione (3c). The cycloaddition of **1a** and 3-phenyl-2-(triethylsilyl)-2-cyclobutenone[3] was performed on 0.10 mmol scale at room temperature for 28 h. Purification was performed by flash chromatography eluting with hexanes/EtOAc (4:1) to provide 32 mg (79%) of **3c** in 92% *ee* as a red solid. ^{1}H NMR (500 MHz, $CDCl_3$) δ 7.46-7.44 (m, 3 H), 7.42-7.40 (m, 2 H), 7.32-7.28 (m, 1 H), 7.06-7.03 (m, 2 H), 6.89 (d, *J* = 7.8 Hz, 1 H), 3.60 (d, *J* = 18 Hz, 1 H), 3.27 (s, 3 H), 3.17 (d, *J* = 18 Hz, 1 H), 0.79 (t, *J* = 8.4 Hz, 9 H), 0.06 (d, *J* = 8.4 Hz, 6 H); ^{13}C NMR (150 MHz, $CDCl_3$) δ 206.0, 186.8, 175.0, 144.9, 138.6, 138.2, 130.4, 129.8, 128.8,

128.5, 126.9, 123.0, 121.5, 108.7, 63.1, 46.3, 26.8, 7.5, 3.5; IR (neat) 2953, 2874, 1717, 1693, 1610, 1491, 1469, 1165 cm^{-1}; HRMS (ESI) *m/z* 404.2054 [$C_{25}H_{30}NO_2Si$(M+H) requires 404.2040]; m.p. = 98-100 ˚C; Chiralpak AD, 25 cm, 98:2 hexanes/^{i}PrOH, 0.5 mL/min, 25 ˚C, 13 bar, t_r (major) = 25.7 min, t_r (minor) = 18.1 min.

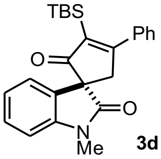

3d

(*R*)-3-(*tert*-Butyldimethylsilyl)-1'-methyl-4-phenylspiro[cyclopentane-1,3'-indolin]-3-ene-2,2'-dione (3d). The cycloaddition of **1a** and 2-(*tert*-butyldimethylsilyl)-3-phenylcyclobut-2-en-1-one was performed on 0.10 mmol scale at room temperature for 28 h. Purification was performed by flash chromatography eluting with hexanes/EtOAc (5:1) to provide 28 mg (69%) of **3d** in 90% *ee* as a pink solid. ^{1}H NMR (600 MHz, $CDCl_3$) δ 7.44-7.43 (m, 3 H), 7.40-7.39 (m, 2 H), 7.32-7.29 (m, 1 H), 7.06-7.05 (m, 2 H), 6.89 (d, *J* = 7.8 Hz, 1 H), 3.59 (d, *J* = 18.6 Hz, 1 H), 3.26 (s, 3 H), 3.14 (d, *J* = 18.6 Hz, 1 H), 0.89 (s, 9 H), -0.08 (s, 3 H), -0.19 (s, 3 H); ^{13}C NMR (150 MHz, $CDCl_3$) δ 206.0, 187.4, 175.0, 144.9, 138.9, 138.8, 130.3, 129.2, 128.8, 128.3, 126.7, 123.0, 121.4, 108.8, 63.0, 47.4, 27.5, 26.8, 17.9, -4.5, -.4.5; IR (neat) 2952, 2857, 1719, 1698, 1611, 1470, 1163 cm^{-1}; HRMS (ESI) *m/z* 404.2059 [$C_{25}H_{30}NO_2Si$(M+H) requires 404.2040]; m.p. = 135-138 ˚C; Chiralpak AD, 25 cm, 98:2 hexanes/^{i}PrOH, 0.5 mL/min, 25 ˚C, 13 bar, t_r (major) = 19.7 min, t_r (minor) = 16.2 min.

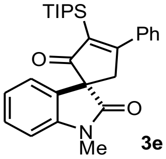

3e

(*R*)-1'-Methyl-4-phenyl-3-(triisopropylsilyl)spiro[cyclopentane-1,3'-indolin]-3-ene-2,2'-dione (3e). The cycloaddition of **1a** and 3-phenyl-2-(triisopropylsilyl)cyclobut-2-en-1-one was performed on 0.10 mmol scale at room temperature for 28 h. Purification was performed by flash chromatography eluting with hexanes/EtOAc (5:1) to provide 24 mg (54%) of **3e** in 90% *ee* as a red solid. ^{1}H NMR (500 MHz, $CDCl_3$) δ 7.43-7.42 (m, 5 H), 7.33-7.28 (m, 1 H), 7.05 (d, *J* = 4 Hz, 2 H), 6.88 (d, *J* = 7.8 Hz, 1 H), 3.60 (d, *J* = 19 Hz, 1 H), 3.26 (s, 3 H), 3.15 (d, *J* = 19 Hz, 1 H), 1.16 (sep, *J* = 8.3 Hz, 3 H), 0.95-0.91 (d, *J* = 8.3 Hz, 18 H); ^{13}C NMR (125 MHz, $CDCl_3$) δ 206.9, 187.9, 175.1, 145.0, 139.2, 137.7, 130.5, 128.9, 128.3, 126.7, 123.0, 121.6, 108.8, 62.9, 48.1, 26.9, 19.2, 19.1, 11.6; IR (neat) 2943, 2865, 1718, 1695, 1611, 1491 cm^{-1}; HRMS (ESI) *m/z* 468.2311 [$C_{28}H_{35}NO_2Si$(M+Na) requires 468.2329]; m.p. = 48 ˚C. Chiralpak AD, 25 cm, 98:2 hexanes/^{i}PrOH, 0.5 mL/min, 25 ˚C, 13 bar, t_r (major) = 17.4 min, t_r (minor) = 12.4 min.

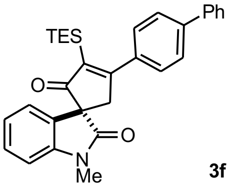

3f

(*R*)-4-([1,1'-biphenyl]-4-yl)-1'-methyl-3-(triethylsilyl)spiro[cyclopentane-1,3'-indolin]-3-ene-2,2'-dione (3f). The cycloaddition of **1a** and 3-([1,1'-biphenyl]-4-yl)-2-(triethylsilyl)cyclobut-2-en-1-one was performed on a 0.10 mmol scale at room temperature for 28 h. Purification was performed by flash chromatography eluting with hexanes/EtOAc (7:1) to provide 19 mg (54%) of **3f** in 86% *ee* as a pink solid. ^{1}H NMR (500 MHz, $CDCl_3$) δ 7.71-7.66 (m, 4 H), 7.52-7.46 (m, 4 H), 7.42-7.38 (m, 1 H), 7.33-7.29 (m, 1 H), 7.05-7.04 (m, 2 H), 6.89 (d, *J* = 7.8 Hz, 1 H), 3.65 (d, *J* = 18 Hz, 1 H), 3.27 (s, 3 H), 3.25

(d, J = 18 Hz, 1 H), 0.82 (t, J = 8.2 Hz, 3 H), 0.61 (q, J = 8.2 Hz, 6 H); ^{13}C NMR (100 MHz, $CDCl_3$) δ 205.9, 186.1, 174.9, 144.7, 142.5, 140.1, 138.1, 137.2, 130.3, 128.9, 128.6, 127.9, 127.5, 127.1, 126.9, 122.8, 121.4, 108.6, 62.9, 46.0, 26.7, 7.41, 3.4; IR (neat) 3019, 2953, 1715, 1693, 1612, 1572, 1486, 1350 cm^{-1}; HRMS (ESI) *m/z* 480.2344 [$C_{31}H_{34}NO_2Si$(M+H) requires 480.2353]; m.p. = 128 ˚C; Chiralpak AD, 25 cm, 96:4 hexanes/^{i}PrOH, 0.5 mL/min, 25 ˚C, 13 bar, t_r (major) = 29.4 min, t_r (minor) = 20.7 min. $[\alpha]_D^{20}$ +74.8 (c 1.00, $CHCl_3$).

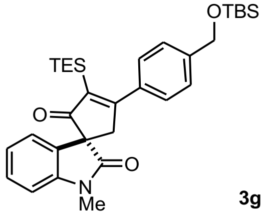

3g

(*R*)-4-(4-(((*tert*-Butyldimethylsilyl)oxy)methyl)phenyl)-1'-methyl-3-(triethylsilyl)spiro[cyclopentane-1,3'-indolin]-3-ene-2,2'-dione (3g). The cycloaddition of **1a** and 3-(4-(((*tert*-butyldimethylsilyl)oxy)methyl)phenyl)-2-(triethylsilyl)cyclobut-2-en-1-one was performed on 0.08 mmol scale at room temperature for 28 h. Purification was performed by flash chromatography eluting with hexanes/EtOAc (5:1) to provide 21 mg (47%) of **3g** in 84% *ee* as an orange oil. ^{1}H NMR (600 MHz, $CDCl_3$) δ 7.42-7.38 (m, 4 H), 7.29 (td, J = 7.8, 1.8 Hz, 1 H), 7.05-7.01 (m, 2 H), 6.88 (d, J = 7.8 Hz, 1 H), 4.81 (s, 2 H), 3.59 (d, J = 18.6 Hz, 1 H), 3.26 (s, 3 H), 3.16 (d, J = 18.6 Hz, 1 H), 0.96 (s, 9 H), 0.80 (t, J = 7.8 Hz, 9 H), 0.57 (t, J = 7.8 Hz, 6 H), 0.12 (s, 6 H); ^{13}C NMR (150 MHz, $CDCl_3$) δ 204.9, 185.6, 174.4, 144.0, 140.1, 137.9, 132.2, 131.6, 130.1, 128.6, 127.3, 124.9, 115.5, 110.1, 62.9, 45.6, 27.0, -0.6; IR (neat) 2953, 2875, 1716, 1693, 1611, 1470, 1165 cm^{-1}; HRMS (ESI) *m/z* 548.3034 [$C_{32}H_{46}NO_3Si_2$(M+H) requires 548.3010]; Chiralpak AD, 25 cm, 97:3 hexanes/^{i}PrOH, 0.5 mL/min, 25 ˚C, 13 bar, t_r (major) = 10.1 min, t_r (minor) = 9.0 min.

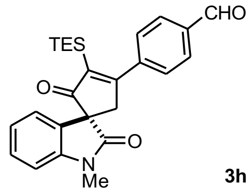

3h

(*R*)-4-(1'-Methyl-2,2'-dioxo-3-(triethylsilyl)spiro[cyclopentane-1,3'-indolin]-3-en-4-yl)benzaldehyde (3h). The cycloaddition of **1a** and 4-(3-oxo-2-(triethylsilyl)cyclobut-1-en-1-yl)benzaldehyde was performed on 0.08 mmol scale at room temperature for 28 h. Purification was performed by flash chromatography eluting with hexanes/EtOAc (2:1) to provide 24 mg (68%) of **3h** in 80% *ee* as a pink solid. ^{1}H NMR (500 MHz, $CDCl_3$) δ 10.12 (s, 1 H), 7.99 (d, J = 7.8 Hz, 2 H), 7.58 (d, J = 7.8 Hz, 2 H), 7.32 (td, J = 7.8, 1.8 Hz, 1 H), 7.07-7.03 (m, 2 H), 6.90 (d, J = 7.2 Hz, 1 H), 3.58 (d, J = 18.6 Hz, 1 H), 3.27 (s, 3 H), 3.18 (d, J = 18.6 Hz, 1 H), 0.80 (t, J = 7.8 Hz, 9 H), 0.53 (d, J = 7.8 Hz, 6 H); ^{13}C NMR (150 MHz, $CDCl_3$) δ 205.6, 191.7, 184.6, 174.7, 144.9, 144.7, 140.1, 136.9, 130.0, 129.8, 129.0, 127.6, 123.1, 121.6, 108.9, 63.0, 46.4, 26.9, 7.5, 3.3; IR (neat) 2950, 2874, 2085, 1712, 1686, 1612, 1504, 1154 cm^{-1}; HRMS (ESI) *m/z* 454.1802 [$C_{26}H_{29}NO_3Si$(M+Na) requires 454.1808]; m.p. = 152-154 ˚C; Chiralpak AD, 25 cm, 90:10 hexanes/^{i}PrOH, 0.5 mL/min, 25 ˚C, 13 bar, t_r (major) = 24.2 min, t_r (minor) = 17.9 min. $[\alpha]_D^{20}$ +86.5 (c 1.00, $CHCl_3$).

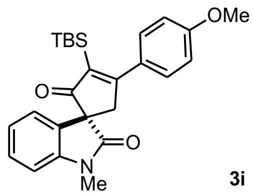

3i

(*R*)-3-(*tert*-butyldimethylsilyl)-4-(4-methoxyphenyl)-1'-methylspiro[cyclopentane-1,3'-indolin]-3-ene-2,2'-dione (3i). The cycloaddition of **1a** and 2-(*tert*-Butyldimethylsilyl)-3-(4-methoxyphenyl)cyclobut-2-en-1-one was performed on 0.08 mmol scale at room temperature for 28 h. Purification was performed by flash chromatography eluting with hexanes/EtOAc (4:1) to provide 35 mg (85%) of **3i** in 75% *ee* as a light red solid. ^{1}H NMR (500 MHz, $CDCl_3$) δ 7.384 (d, *J* = 8.8 Hz, 2 H), 7.29 (m, 1 H), 7.06-7.01 (m, 2 H), 6.96 (d, *J* = 8.8 Hz, 2 H), 6.88 (d, *J* = 8.1 Hz, 1 H), 3.86 (s, 3 H), 3.59 (d, *J* = 18.5 Hz, 1 H), 3.26 (s, 3 H), 3.13 (d, *J* = 18.5 Hz, 1 H), 0.92 (s, 9 H), -0.03 (s, 3 H), -0.14 (s, 3 H). ^{13}C NMR (100 MHz, $CDCl_3$) δ 204.9, 185.8, 174.0, 159.6, 143.6, 136.7, 129.5, 127.5, 121.8, 120.3, 112.5, 107.6, 61.9, 54.3, 45.7, 26.5, 25.7, 17.0, -0.0, -1.0, -5.3; IR (neat); 2948, 2080, 1709, 1682; m.p. = 131-133 ˚C. Chiralpak AD, 25 cm, 97:3 hexanes/^{i}PrOH, 0.5 mL/min, 25 ˚C, 13 bar, t_r (major) = 14.46 min, t_r (minor) = 13.36 min.

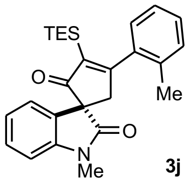

3j

(*R*)-1'-Methyl-4-(*o*-tolyl)-3-(triethylsilyl)spiro[cyclopentane-1,3'-indolin]-3-ene-2,2'-dione (3j). The cycloaddition of **1a** and 3-(*o*-tolyl)-2-(triethylsilyl)cyclobut-2-en-1-one was performed on a 0.10 mmol scale at room temperature for 28 h. Purification was performed by flash chromatography eluting with hexanes/EtOAc (4:1) to provide 13 mg (32%) of **3j** in 64% *ee* as a red oil. ^{1}H NMR (400 MHz, $CDCl_3$) δ 7.33-7.30 (m, 2 H), 7.28-7.25 (m, 2 H), 7.16 (br s, 1 H), 7.09-7.05 (m, 2 H), 6.89 (d, *J* = 7.8 Hz, 1 H), 3.46 (br d, *J* = 18.5 Hz, 1 H), 3.26 (s, 3 H), 3.12 (br d, *J* = 18.5 Hz, 1 H), 2.43 (br s, 3 H), 0.79 (t, *J* = 8.1 Hz, 9 H), 0.43 (t, *J* = 8.1 Hz, 6 H); ^{13}C NMR (125 MHz, $CDCl_3$) δ 206.2, 188.6, 174.9, 145.0, 139.5, 138.6, 132.5, 130.4, 128.8, 128.7, 126.5, 125.8, 123.0, 121.7, 108.7, 107.8, 62.9, 46.9, 26.8, 19.7, 7.5, 2.5; IR (neat) 2951, 2873, 2081, 1717, 1700, 1609 cm^{-1}; HRMS (ESI) *m/z* 418.2158 [$C_{26}H_{32}NO_2Si$(M+H) requires 418.2196]; Chiralpak AD, 25 cm, 95:5 hexanes/^{i}PrOH, 0.5 mL/min, 25 ˚C, 13 bar, t_r (major) = 12.6 min, t_r (minor) = 10.4 min.

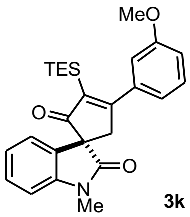

3k

(*R*)-4-(3-Methoxyphenyl)-1'-methyl-3-(triethylsilyl)spiro[cyclopentane-1,3'-indolin]-3-ene-2,2'-dione (3k). The cycloaddition of **1a** and 3-(3-methoxyphenyl)-2-(triethylsilyl)cyclobut-2-en-1-one was performed on 0.13 mmol scale at room temperature for 28 h. Purification was performed by flash chromatography eluting with hexanes/EtOAc (6:1) to provide 44 mg (78%) of **3k** in 88% *ee* as a red oil. ^{1}H NMR (500 MHz, $CDCl_3$) δ 7.36 (t, *J* = 7.9 Hz, 1 H), 7.30 (dd, *J* = 6.9, 2.0 Hz, 1 H), 7.06-7.01 (m, 4 H), 6.93-6.92, (m, 1 H), 6.89 (d, *J* = 7.8 Hz, 1 H); ^{13}C NMR (150 MHz, $CDCl_3$) δ 3.87 (s, 3 H), 3.59 (d, *J* = 18 Hz, 1 H), 3.26 (s, 3H), 3.16 (d, *J* = 18 Hz, 1 H), 0.81 (t, *J* = 8.1 Hz, 9 H), 0.59 (q, *J* = 8.1 Hz, 6 H); ^{13}C NMR (125 MHz, $CDCl_3$) δ 206.1, 186.7, 175.1, 159.6, 145.0, 140.0, 138.3, 130.5, 129.7, 128.9, 123.1,

121.6, 119.4, 115.3, 112.6, 108.9, 63.1, 55.6, 46.4, 26.9, 7.6, 3.5; IR (neat) 2956, 2874, 1718, 1695, 1611, 1569, 1470, 1220, 1152; HRMS (ESI) *m/z* 456.1970 [$C_{26}H_{31}NNaO_3Si$(M+Na) requires 456.1971]; Chiralpak AD, 25 cm, 96:4 hexanes/^{i}PrOH, 0.5 mL/min, 25 ˚C, 13 bar, t_r (major) = 18.1 min, t_r (minor) = 14.8 min. $[\alpha]_D^{20}$ +79.7 (c 1.00, $CHCl_3$).

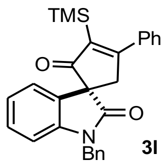

3l

(*R*)-1'-Benzyl-4-phenyl-3-(trimethylsilyl)spiro[cyclopentane-1,3'-indolin]-3-ene-2,2'-dione (3l). The cycloaddition of **1b** and **2b** was performed on 0.08 mmol scale at 4 ˚C for 48 h. Purification was performed by flash chromatography eluting with hexanes/EtOAc (5:1) to provide 32 mg (91%) of **3l** in 90% *ee* as a red solid. ^{1}H NMR (500 MHz, $CDCl_3$) δ 7.47-7.45 (m, 3 H), 7.44-7.42 (m, 2 H), 7.38-7.33 (m, 4 H), 7.28-7.25 (m, 1 H), 7.16 (td, *J* = 7.9, 1.2 Hz, 1 H), 7.05-6.99 (m, 3 H), 6.71 (d, *J* = 7.9 Hz, 1 H), 5.12 (d, *J* = 16 Hz, 1 H), 4.84 (d, *J* = 16 Hz, 1 H), 3.68 (d, *J* = 19 Hz, 1 H), 3.23 (d, *J* = 19 Hz, 1 H), 0.07 (s, 9 H); ^{13}C NMR (150 MHz, $CDCl_3$) δ 205.4, 185.3, 175.2, 143.9, 140.2, 138.3, 135.6, 130.5, 129.9, 129.0, 128.7, 128.5, 127.7, 127.2, 127.2, 123.0, 121.5, 109.8, 63.1, 46.0, 44.2, -0.5; IR (neat) 3056, 2954, 1714, 1692, 1608, 1581, 1565, 1162, cm^{-1}; HRMS (ESI) *m/z* 438.1871 [$C_{28}H_{28}NO_2Si$(M+Na) requires 438.1884]; m.p. = 164-170 ˚C. Chiralpak AD, 25 cm, 94:6 hexanes/^{i}PrOH, 0.75 mL/min, 25 ˚C, 13 bar, t_r (major) = 21.4 min, t_r (minor) = 13.3 min. $[\alpha]_D^{20}$ +68.5 (c 1.00, $CHCl_3$).

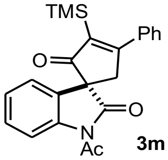

3m

(*R*)-1'-Acetyl-4-phenyl-3-(trimethylsilyl)spiro[cyclopentane-1,3'-indolin]-3-ene-2,2'-dione (3m). The cycloaddition of **1c** and **2b** was performed on 0.08 mmol scale at 4 ˚C for 48 h. Purification was performed by flash chromatography eluting with hexanes/EtOAc (7:1) to provide 22 mg (72%) of **3m** in 79% *ee* as a pink solid. ^{1}H NMR (600 MHz, $CDCl_3$) δ 8.29 (d, *J* = 8.3 Hz, 1 H), 7.48-7.47 (m, 3 H), 7.42-41 (m, 2 H), 7.35 (t, *J* = 8.3 Hz, 1 H), 7.20 (t, *J* = 7.6 Hz, 1 H), 7.05 (d, *J* = 6.9 Hz, 1 H), 3.65 (d, *J* = 19 Hz, 1 H), 3.23 (d, *J* = 19 Hz, 1 H), 2.69 (s, 3 H), 0.06 (s, 9 H); ^{13}C NMR (150 MHz, $CDCl_3$) δ 204.5, 185.6, 176.0, 170.7, 141.1, 139.5, 137.8, 130.2, 129.2, 129.1, 128.6, 127.2, 125.7, 121.1, 117.2, 63.7, 46.9, 26.7, -0.6; IR (neat) 3056, 2954, 1754, 1698, 1603, 1581, 1563, 1151, cm^{-1}; HRMS (ESI) *m/z* 390.1508 [$C_{23}H_{24}NO_3Si$(M+H) requires 390.1519]; m.p. = 123-128 ˚C. Chiralpak AD, 25 cm, 98:2 hexanes/^{i}PrOH, 0.5 mL/min, 25 ˚C, 13 bar, t_r (major) = 28.3 min, t_r (minor) = 18.2 min. $[\alpha]_D^{20}$ +73.8 (c 1.00, $CHCl_3$).

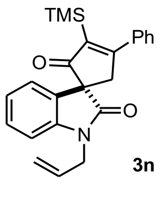

3n

(*R*)-1'-Allyl-4-phenyl-3-(trimethylsilyl)spiro[cyclopentane-1,3'-indolin]-3-ene-2,2'-dione (3n). The cycloaddition of **1d** and **2b** was performed on 0.08 mmol scale at room temperature for 28 h. Purification was performed by flash chromatography eluting with hexanes/EtOAc (4:1) to provide 23 mg (74%) of **3n** in 77% *ee* as a yellow solid. ^{1}H NMR (600 MHz, $CDCl_3$) δ 7.47-7.45 (m, 3 H), 7.43-7.41 (m, 2 H), 7.28-7.25 (m, 1 H), 7.04-7.03 (m, 2 H), 6.87 (d, *J* = 7.8 Hz, 1 H), 5.87 (ddd, *J* = 17.0, 10.8, 4.8 Hz, 1 H), 5.35 (dd, *J* = 17.0, 1.2 Hz, 1 H), 5.25 (dd, *J* = 10.8, 1.2 Hz, 1 H), 4.47 (dd, *J* = 16.4, 4.8 Hz, 1 H),

4.30 (dd, J = 16.4, 4.8 Hz, 1 H), 3.63 (d, J = 18.6 Hz, 1 H), 3.19 (d, J = 18.6 Hz, 1 H), 0.05 (s, 9 H); ^{13}C NMR (150 MHz, $CDCl_3$) δ 205.5, 185.4, 174.8, 144.0, 140.1, 138.3, 131.1, 130.4, 130.0 128.6, 128.5, 127.2, 122.9, 121.5, 117.7, 109.6, 63.0, 45.9, 42.8, -0.5; IR (neat) 2360, 1717, 1693, 1609, 1564, 1487, 1355, 1163 cm^{-1}; HRMS (ESI) *m/z* 388.1746 [$C_{24}H_{26}NO_2Si$(M+H) requires 388.1727]; m.p. = 101-104 ˚C. Chiralpak AD, 25 cm, 97:3 hexanes/^{i}PrOH, 0.5 mL/min, 25 ˚C, 13 bar, t_r (major) = 40.9 min, t_r (minor) = 11.2 min. $[\alpha]_D^{20}$ +84.8 (c 1.00, $CHCl_3$).

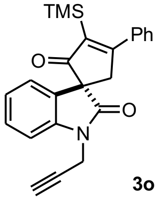

3o

(*R*)-4-Phenyl-1'-(prop-2-yn-1-yl)-3-(trimethylsilyl)spiro[cyclopentane-1,3'-indolin]-3-ene-2,2'-dione (3o). The cycloaddition of **1e** (40% PhMe in CH_2Cl_2) and **2b** was performed on 0.08 mmol scale at 4 ˚C for 48 h. Purification was performed by flash chromatography eluting with hexanes/EtOAc (6:1) to provide 21 mg (68%) of **3o** in 90% *ee* as a red solid. ^{1}H NMR (500 MHz, $CDCl_3$) δ 7.47-7.45 (m, 3 H), 7.42-7.40 (m, 2 H), 7.33 (td, J = 7.3, 1.6 Hz, 1 H), 7.13 (d, J = 7.3 Hz, 1 H), 7.06 (td, J = 7.3, 1.0 Hz, 1 H), 7.06-7.04 (m, 1 H), 4.71 (dd, J = 17.8, 2.6 Hz, 1 H), 4.42 (dd, J = 17.8, 2.6 Hz, 1 H), 3.63 (d, J = 18.6 Hz, 1 H), 3.19 (d, J = 18.6 Hz, 1 H), 2.26 (t, J = 2.6 Hz, 1 H), 0.05 (s, 9 H); ^{13}C NMR (150 MHz, $CDCl_3$) δ 205.2, 185.4, 174.2, 142.8, 140.1, 138.1, 130.2, 130.0, 128.8, 128.5, 127.2, 123.4, 121.6, 109.8, 76.9, 72.7, 62.9, 45.9, 29.8, -0.6; IR (neat) 3294, 2954, 2362, 2092, 1720, 1693, 1610, 1487, 1162 cm^{-1}; HRMS (ESI) *m/z* 386.1562 [$C_{24}H_{23}NO_2Si$(M+H) requires 386.1571]; m.p. = 115-118 ˚C. Chiralpak AD, 25 cm, 97:3 hexanes/^{i}PrOH, 0.5 mL/min, 25 ˚C, 13 bar, t_r (major) = 15.2 min, t_r (minor) = 9.1 min. $[\alpha]_D^{20}$ +53.4 (c 1.00, $CHCl_3$).

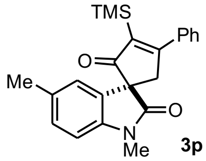

3p

(*R*)-1',5'-Dimethyl-4-phenyl-3-(trimethylsilyl)spiro[cyclopentane-1,3'-indolin]-3-ene-2,2'-dione (3p). The cycloaddition of **1f** and **2b** was performed on 0.08 mmol scale at 4 ˚C for 48 h. Purification was performed by flash chromatography eluting with hexanes/EtOAc (5:1) to provide 28 mg (93%) of **3p** in 90% *ee* as a white solid. ^{1}H NMR (500 MHz, $CDCl_3$) δ 7.47-7.45 (m, 3 H), 7.44-7.42 (m, 2 H), 7.10 (dd, J = 7.8, 0.6 Hz, 1 H), 6.83 (d, J = 0.6 Hz, 1 H), 6.78 (d, J = 7.8 Hz, 1 H), 3.58 (d, J = 18.6 Hz, 1 H), 3.25 (s, 3 H), 3.16 (d, J = 18.6 Hz, 1 H), 2.32 (s, 3 H), 0.05 (s, 9 H); ^{13}C NMR (150 MHz, $CDCl_3$) δ 205.9, 185.5, 174.9, 142.5, 140.2, 138.3, 132.6, 130.3, 129.9, 129.0, 128.5, 127.3, 122.4, 108.4, 63.1, 45.9, 26.9, 21.3, -0.5; IR (neat) 3057, 2954, 1714, 1691, 1602, 1581, 1498, 1166, cm^{-1}; HRMS (ESI) *m/z* 376.1702 [$C_{23}H_{26}NO_2Si$(M+H) requires 376.1727]; m.p. = 150-153 ˚C. Chiralpak AD, 25 cm, 97:3 hexanes/^{i}PrOH, 0.5 mL/min, 25 ˚C, 13 bar, t_r (major) = 35.7 min, t_r (minor) = 16.8 min. $[\alpha]_D^{20}$ +71.7 (c 1.00, $CHCl_3$).

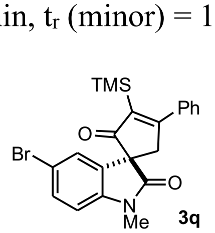

3q

(*R*)-5'-Bromo-1'-methyl-4-phenyl-3-(trimethylsilyl)spiro[cyclopentane-1,3'-indolin]-3-ene-2,2'-dione (3q). The cycloaddition of **1g** (10% CH_2Cl_2 in PhMe) and **2b** was performed on 0.08 mmol scale at 4 ˚C for 48 h. Purification was performed by flash chromatography eluting with hexanes/EtOAc (5:1) to

provide 31 mg (90%) of **3q** in 77% *ee* as a red solid. ^{1}H NMR (500 MHz, $CDCl_3$) δ 7.47-7.46 (m, 3 H), 7.44-7.41 (m, 3 H), 7.13 (d, *J* = 1.9 Hz, 1 H), 6.77 (d, *J* = 8.3 Hz, 1 H), 3.59 (d, *J* = 18.6 Hz, 1 H), 3.25 (s, 3 H), 3.17 (d, *J* = 18.6 Hz, 1 H), 0.06 (s, 9 H); ^{13}C NMR (150 MHz, $CDCl_3$) δ 204.9, 185.6, 174.4, 144.0, 140.1, 137.9, 132.2, 131.6, 130.1, 128.6, 127.3, 124.9, 115.5, 110.1, 62.9, 45.6, 27.0, -0.6; IR (neat) 3060, 2925, 2338, 2084, 1718, 1695, 1606, 1581, 1565, 1167, cm^{-1}; HRMS (ESI) *m/z* 440.0683 [$C_{22}H_{23}BrNO_2Si$(M+H) requires 440.0658]; m.p. = 80-85 ˚C. Chiralpak AD, 25 cm, 96:4 hexanes/^{i}PrOH, 0.75 mL/min, 25 ˚C, 13 bar, t_r (major) = 17.7 min, t_r (minor) = 10.7 min. $[\alpha]_D^{20}$ +62.1 (c 1.00, $CHCl_3$).

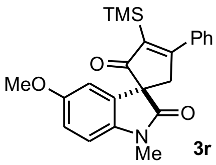

3r

(*R*)-5'-Methoxy-1'-methyl-4-phenyl-3-(trimethylsilyl)spiro[cyclopentane-1,3'-indolin]-3-ene-2,2'-dione (3r). The cycloaddition of **1h** and **2b** was performed on 0.08 mmol scale at room temperature for 28 h. Purification was performed by flash chromatography eluting with hexanes/EtOAc (4:1) to provide 14 mg (43%) of **3r** in 84% *ee* as a purple solid. ^{1}H NMR (500 MHz, $CDCl_3$) δ 7.46-7.44 (m, 3 H), 7.42-7.40 (m, 2 H), 6.83-6.78 (m, 2 H), 6.64 (d, *J* = 2.25 Hz, 1 H), 3.77 (s, 3 H), 3.61 (d, *J* = 18.6 Hz, 1 H), 3.24 (s, 3 H), 3.15 (d, *J* = 18.6 Hz, 1 H), 0.04 (s, 9 H); ^{13}C NMR (150 MHz, $CDCl_3$) δ 205.6, 185.4, 174.7, 156.3, 140.2, 138.4, 138.2, 131.6, 129.9, 128.5, 127.2, 112.3, 109.7, 108.9, 63.4, 56.0, 45.9, 26.9, -0.6; IR (neat) 3058, 2953, 1714, 1689, 1602, 1581, 1566, 1496, 1169, cm^{-1}; HRMS (ESI) *m/z* 392.1667 [$C_{23}H_{26}NO_3Si$(M+H) requires 392.1676]; m.p. = 75-80 ˚C. Chiralpak AD, 25 cm, 96:4 hexanes/^{i}PrOH, 1.0 mL/min, 25 ˚C, 13 bar, t_r (major) = 25.1 min, t_r (minor) = 12.4 min. $[\alpha]_D^{20}$ +53.3 (c 1.00, $CHCl_3$).

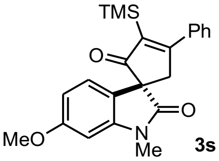

3s

(*R*)-6'-Methoxy-1'-methyl-4-phenyl-3-(trimethylsilyl)spiro[cyclopentane-1,3'-indolin]-3-ene-2,2'-dione (3s). The annulation of **1i** and **2b** was performed on a 0.10 mmol scale at room temperature for 28 h. Purification was performed by flash chromatography eluting with hexanes/EtOAc (3:1) to provide 19 mg (49%) of **3s** in 86% *ee* as a beige solid. ^{1}H NMR (400 MHz, $CDCl_3$) δ 7.46-7.44 (m, 3 H), 7.42-7.39 (m, 2 H), 6.92 (d, *J* = 8.2 Hz, 1 H), 6.54 (dd, *J* = 8.2, 2.3 Hz, 1 H), 6.48 (d, *J* = 2.3 Hz, 1 H), 3.83 (s, 3 H), 3.58 (d, *J* = 19 Hz, 1 H), 3.24 (s, 3H), 3.13 (d, *J* = 19 Hz, 1 H), 0.04 (s, 9 H); ^{13}C NMR (100 MHz, $CDCl_3$) δ 206.6, 185.8, 176.1, 161.3, 146.6, 140.1, 138.9, 130.4, 129.0, 127.8, 122.8, 122.7, 107.4, 97.4, 63.1, 56.3, 46.5, 27.4; IR (neat) 3023, 1717, 1692, 1626, 1375 cm^{-1}; HRMS (ESI) *m/z* 392.1697 [$C_{23}H_{26}NO_3Si$(M+H) requires 392.1676]; m.p. = 164-166 ˚C. Chiralpak AD, 25 cm, 93:7 hexanes/^{i}PrOH, 0.5 mL/min, 25 ˚C, 13 bar, t_r (major) = 28.5 min, t_r (minor) = 18.6 min. $[\alpha]_D^{20}$ +75.9 (c 1.00, $CHCl_3$).

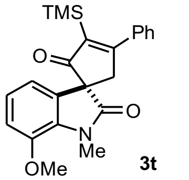

3t

(*R*)-7'-Methoxy-1'-methyl-4-phenyl-3-(trimethylsilyl)spiro[cyclopentane-1,3'-indolin]-3-ene-2,2'-dione (3t). The annulation of **1j** and **2b** was performed on a 0.08 mmol scale at room temperature for 28 h. Purification was performed by flash chromatography eluting with hexanes/EtOAc (4:1) to provide 24 mg (75%) of **3t** in 88% *ee* as a red solid. ^{1}H NMR (400 MHz, $CDCl_3$) δ 7.45-7.43 (m, 2 H), 7.41-7.39

(m, 2 H), 6.97 (m, 1 H), 6.86 (app d, J = 8.4 Hz, 1 H), 6.64 (dd, J = 8.4, 0.9 Hz, 1 H), 3.86 (s, 3 H), 3.58 (d, J = 19 Hz, 1 H), 3.53 (s, 3 H), 3.13 (d, J = 19 Hz, 1 H), 0.04 (s, 9 H), ^{13}C NMR (125 MHz, $CDCl_3$) δ 205.5, 185.3, 175.1, 145.7, 140.1, 138.3,132.7, 131.8, 129.8, 128.5, 127.2, 123.6, 114.2, 112.7, 63.3, 56.2, 46.2, 30.1, -0.6; IR (neat) 2951, 1717, 1695, 1490 cm^{-1}; HRMS (ESI) *m/z* 392.1637 [$C_{23}H_{26}NO_3Si$(M+H) requires 392.1676]; m.p. = 128-131 ˚C; Chiralpak AD, 25 cm, 92:8 hexanes/^{i}PrOH, 0.5 mL/min, 25 ˚C, 13 bar, t_r (major) = 20.3 min, t_r (minor) = 11.3 min. $[\alpha]_D^{20}$ +66.4 (c 1.00, $CHCl_3$).

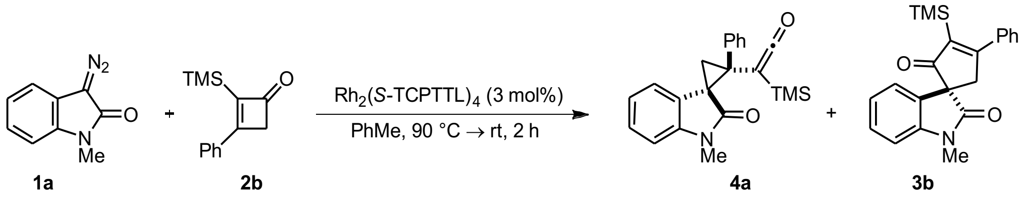

(1*R*,2*R*)-1'-Methyl-2-(2-oxo-1-(trimethylsilyl)vinyl)-2-phenylspiro[cyclopropane-1,3'-indolin]-2'-one (4a). A solution of **2b** (38 mg, 0.18 mmol) and Rh_2(*S*-TCPTTL)$_4$ (9.4 mg, 3.0 μmol) in PhMe (0.75 mL) was stirred for 20 min at 90 ˚C then cooled to room temperature by removal of the oil bath. A solution of **1a** (36 mg, 0.21 mmol) in PhMe (1.0 mL) was then added slowly over 1 h and the resulting mixture stirred for an additional 1 h. The mixture was filtered through a pad of SiO_2 eluting first with hexanes (10 mL) then hexanes/EtOAc (2:1) and the filtrate concentrated under reduced pressure [note: rotary evaporator bath temperature not to exceed 40 ˚C]. The crude residue was purified by flash chromatography eluting with hexanes/EtOAc (6:1) to provide 38 mg (60%) of **4a** in 95% *ee* as a pink solid (isolated as a 9:1 mixture with **3b** determined by ^{1}H NMR (500 MHz) analysis of the crude mixture (**4a**: 2.60 (d, 1 H); **3b**: 3.60 (d, 1 H)). ^{1}H NMR (500 MHz, $CDCl_3$) δ 7.29 (td, J = 7.7, 1.3 Hz, 1 H), 7.30-7.27 (m, 2 H), 7.25-7.22 (m, 3 H), 7.20 (dd, J = 7.5, 1.0 Hz, 1 H), 7.09 (td, J = 7.6, 1.0 Hz, 1 H), 6.90 (d, J = 7.7, 1 H), 3.17 (s, 3 H), 2.60 (d, J = 5.1, 1 H), 1.90 (d, J = 5.1, 1 H), 0.09 (s, 9 H); ^{13}C NMR (125 MHz, $CDCl_3$) δ 181.1, 173.8, 144.7, 141.3, 129.1, 128.2, 127.5, 127.4, 123.0, 122.5, 121.6, 108.7, 108.0, 40.27, 38.8, 28.5, 26.7, -0.4; IR (neat) 2955, 2084, 1717, 1614, 1492, 1469, 1252 cm^{-1}; HRMS (ESI) *m/z* 362.1561 [$C_{22}H_{24}NO_2Si$(M+H) requires 362.1570]. m.p. = 196-199 ˚C. Chiralpak AD, 25 cm, 97:3 hexanes/^{i}PrOH, 0.5 mL/min, 25 ˚C, 13 bar, t_r (major) = 13.3 min, t_r (minor) = 20.6 min.

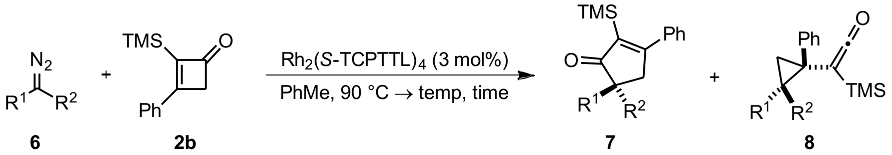

General procedure for the enantioselective Rh^{II}-catalyzed, formal [4+1]-cycloaddition of diazo compound 1 and cyclobutenone 2: A solution of **2b** (0.1 mmol) and Rh_2(*S*-TCPTTL)$_4$ (5.4 mg, 3.0 μmol) in PhMe (0.33 mL) was stirred at 90 ˚C for 20 min then cooled to the indicated temperature. A solution of **1** (0.12 mmol) in PhMe (0.67 mL) was added slowly over 1 h, stirred for an additional 2 h, then SiO_2 (1 mmol) was added and stirred for the indicated time. The reaction mixture was concentrated under reduced pressure and the crude residue purified by flash chromatography eluting with hexanes/EtOAc at the indicated ratio (10:1-20:1) to provide the title cyclopentenone **7** or cyclopropyl ketene **8**.

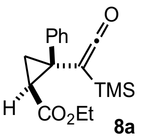

8a

Methyl (1*R*,2*R*)-2-(2-oxo-1-(trimethylsilyl)vinyl)-2-phenylcyclopropane-1-carboxylate (8a). The cyclopropanation of **6a** and **2b** was performed on a 0.08 mmol scale at room temperature for 5 h. Purification was performed by flash chromatography eluting with hexanes/EtOAc (20:1) to provide 13 mg (54%) of **4a** in a 1.4:1 mixture of diastereomers. Diastereoselectivity was determined by ^{1}H NMR (500 MHz) analysis of the crude mixture (**4a**: 2.16 (dd, 1 H); **minor**: 2.38 (dd, 1 H)). **4a:** Yellow oil. ^{1}H NMR (500 MHz, $CDCl_3$) δ 7.29-7.27 (m, 2 H), 7.24-7.22 (m, 2 H), 7.20-7.17 (comp, 1 H), 3.83 (qd, J = 7.2, 1.2 Hz, 2 H), 2.16 (dd, J = 8.2, 5.8 Hz, 1 H), 2.07 (dd, J = 5.8, 5.0 Hz, 1 H), 1.33 (dd, J = 8.2, 5.0 Hz, 1 H), 0.97 (t, J = 7.2 Hz, 3 H), 0.22 (s, 9 H); ^{13}C NMR (100 MHz, $CDCl_3$) δ 184.5, 161.5, 130.9, 129.6, 128.2, 127.8, 127.3, 60.3, 48.7, 33.8, -1.8; IR (neat) 2929, 2361, 1719, 1174 cm^{-1}; HRMS (ESI) *m/z* 303.1437 [$C_{17}H_{22}O_3Si$(M+H) requires 362.1416].

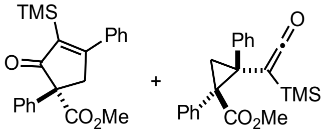

7b **8b**

Methyl (*R*)-2-oxo-1,4-diphenyl-3-(trimethylsilyl)cyclopent-3-ene-1-carboxylate (7b). The cycloaddition of **6b** and **2b** was performed on a 0.10 mmol scale at 60 ˚C for 20 h. Purification was performed by flash chromatography eluting with hexanes/EtOAc (20:1 then 15:1) to provide 27 mg (63%) of **8b** and **7b** in a 2.3:1 ratio. Ratio determined by ^{1}H NMR (500 MHz) analysis of the crude mixture (**8b**: 2.67 (d, 1 H); **7b**: 4.07 (d, 1 H)). **7b:** Colorless oil, 54% *ee*. ^{1}H NMR (500 MHz, $CDCl_3$) δ 7.41-7.39 (m, 3 H), 7.36-7.35 (m, 4 H), 7.31-7.29 (m, 3 H), 4.07 (d, J = 19 Hz, 1 H), 3.75 (s, 3 H), 3.28 (d, J = 19 Hz, 1 H), 0.68 (s, 9 H); ^{13}C NMR (125 MHz, $CDCl_3$) δ 206.7, 183.2, 171.9, 140.0, 138.7, 136.0, 130.2, 129.3, 128.9, 128.6, 128.0, 127.7, 65.6, 53.8, 49.7, 0.6; IR (neat) 2952, 1726, 1698, 1249 cm^{-1}; HRMS (ESI) *m/z* 365.1540 [$C_{22}H_{25}O_3Si$(M+H) requires 365.1567]. Chiralpak AD, 25 cm, 99:1 hexanes/^{i}PrOH, 1.0 mL/min, 25 ˚C, 13 bar, t_r (major) = 11.9 min, t_r (minor) = 13.2 min. **Methyl (1*R*,2*R*)-2-(2-oxo-1-(trimethylsilyl)vinyl)-1,2-diphenylcyclopropane-1-carboxylate (8b):** Colorless oil, 52% *ee*. Stereochemistry determined by ROESY analysis (correlation between 2.67 (d, 1 H) and 7.34 (m, 2 H); 1.70 (d, 1 H) and 7.60 (m, 2 H) ppm). ^{1}H NMR (500 MHz, $CDCl_3$) δ 7.60-7.57 (m, 2 H), 7.42-7.34 (m, 5 H), 7.33-7.29 (m, 2 H), 7.21 (td, J = 6.7, 1.4 Hz, 1 H), 7.09 (td, J = 7.6, 1.0 Hz, 1 H), 3.20 (s, 3 H), 2.67 (d, J = 5.6 Hz, 1 H), 1.70 (d, J = 5.6 Hz, 1 H), -0.12 (s, 9 H); ^{13}C NMR (125 MHz, $CDCl_3$) δ 179.6, 170.6, 142.6, 136.2, 131.9, 128.7, 128.3, 128.1, 128.0, 127.1, 52.2, 42.5, 36.2, 23.5, 22.0, -0.25; IR (neat) 2952, 2080, 1726, 1249 cm^{-1}; HRMS (ESI) *m/z* 365.1548 [$C_{22}H_{25}O_3Si$(M+H) requires 365.1567]; Chiralpak AD, 25 cm, 99:1 hexanes/^{i}PrOH, 0.5 mL/min, 25 ˚C, 13 bar, t_r (major) = 12.6 min, t_r (minor) = 17.3 min. $[\alpha]_D^{20}$ +14.2 (c 1.00, $CHCl_3$).

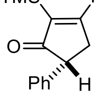

7c

(*R*)-3,5-Diphenyl-2-(trimethylsilyl)cyclopent-2-en-1-one (7c). The cycloaddition of **6c** and **2b** was performed on a 0.08 mmol scale at rt for 16 h. Purification was performed by flash chromatography eluting with hexanes/EtOAc (30:1) to provide 15 mg (63%) of **7c** in 82% *ee* as a colorless oil. ^{1}H NMR (500 MHz, $CDCl_3$) δ 7.37-7.41 (m, 3 H), 7.35-7.32 (m, 4 H), 7.24 (comp, 1 H), 7.20-7.19 (m, 2 H), 3.71, (dd,

J = 7.5, 3.1 Hz, 1 H), 4.43 (dd, J = 19,7.5 Hz, 1 H), 3.06 (dd, J = 19, 3.1 Hz, 1 H), 0.2 (s, 9 H); ^{13}C NMR (100 MHz, $CDCl_3$) δ 212.3, 183.9, 141.1, 140.2, 139.1, 129.4, 128.9, 128.4, 127.7, 126.9, 53.0, 44.6, -0.4; IR (neat) 2953, 1712, 1694, 1248 cm^{-1}; HRMS (ESI) *m/z* 307.1535 [$C_{20}H_{23}OSi$(M+H) requires 307.1512]. Chiralpak AD, 25 cm, 97:3 hexanes/^{i}PrOH, 0.5 mL/min, 25 ˚C, 13 bar, t_r (major) = 13.5 min, t_r (minor) = 10.4 min. $[\alpha]_D^{20}$ +54.6 (c 1.00, $CHCl_3$).

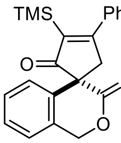

7d

(*R*)-4-Phenyl-3-(trimethylsilyl)spiro[cyclopentane-1,4'-isochroman]-3-ene-2,3'-dione (7d). The cycloaddition of **6d** and **2b** was performed on a 0.10 mmol scale at 4 ˚C for 48 h. Purification was performed by flash chromatography eluting with hexanes/EtOAc (20:1) to provide 27 mg (75%) of **7d** in 86% *ee* as a colorless oil. ^{1}H NMR (500 MHz, $CDCl_3$) δ 7.49-7.48 (m, 3 H), 7.46-7.35 (m, 2 H), 7.34-7.32 (m, 2 H), 7.26-7.24 (m, 2 H), 7.09 (comp, 1 H), 6.13, (d, J = 14 Hz, 1 H), 5.32 (d, J = 14 Hz, 1 H), 4.22 (d, J = 19 Hz, 1 H), 3.36 (d, J = 19 Hz, 1 H), -0.2 (s, 9 H); ^{13}C NMR (125 MHz, $CDCl_3$) δ 206.2, 185.9, 169.6, 138.1, 136.1, 133.0, 131.6, 130.1, 128.7, 128.6, 127.9, 127.2, 125.3, 123.7, 71.2, 61.6, 46.4, -0.6; IR (neat) 2955, 1731, 1698, 1248 cm^{-1}; HRMS (ESI) *m/z* 363.1412 [$C_{22}H_{23}O_3Si$(M+H) requires 363.1410]. Chiralpak AD, 25 cm, 97:3 hexanes/^{i}PrOH, 0.75 mL/min, 25 ˚C, 13 bar, t_r (major) = 20.5 min, t_r (minor) = 15.3 min. $[\alpha]_D^{20}$ +28.7 (c 1.00, $CHCl_3$).

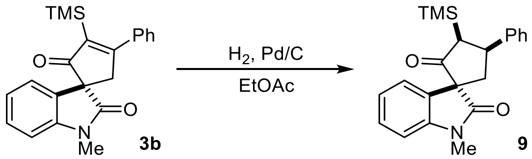

(1*R*,3*S*,4*R*)-1'-Methyl-4-phenyl-3-(trimethylsilyl)spiro[cyclopentane-1,3'-indoline]-2,2'-dione (9). To a stirring solution of Pd/C (4.2 mg, 20 μmol) in ethyl acetate (0.1 mL) was added a solution of **3b** (36 mg, 0.1 mmol) in ethyl acetate (0.4 mL). The mixture stirred for 5 h and then filtered through a pad of celite and concentrated under reduced pressure. The crude residue was purified by flash chromatography eluting with hexanes/EtOAc (3:1) to provide 31 mg (85%) of **9** in a 6:1 mixture of diastereomers. Stereochemistry determined by ROESY analysis (correlation between 7.39 (m, 4 H) and -0.07 (s, 9 H); 7.39 (m, 4 H) and 6.88 (d, 1 H) ppm). Diastereoselectivity was determined by ^{1}H NMR (500 MHz) analysis of the crude mixture (**9**: 4.35 (comp, 1 H); **minor**: 4.83 (comp, 1 H)). **9:** white solid, 99% *ee*. ^{1}H NMR (500 MHz, $CDCl_3$) δ 7.39-7.36 (m, 4 H), 7.32-7.28 (m, 2 H), 7.25 (d, J = 7.6 Hz, 1 H), 7.03 (td, J = 7.6, 1.0 Hz, 1 H), 6.88 (d, J = 7.7 Hz, 1 H), 4.35 (comp, 1 H), 3.28 (d, J = 13 Hz, 1 H), 3.26 (s, 3 H), 2.84 (dd, J = 8.6, 2.1 Hz, 1 H), 2.52 (ddd, J = 13, 5.7, 2.1 Hz, 1 H), -0.07 (s, 9 H); ^{13}C NMR (125 MHz, $CDCl_3$) δ 211.9, 175.8, 144.4, 139.9, 131.8, 128.8, 128.7, 128.4, 127.4, 122.9, 122.4, 108.7, 64.5, 51.5, 43.3, 37.1, 26.7, -0.09; IR (neat) 2923, 1698, 1610, 1492 cm^{-1}; m.p. 165-168 ˚C; HRMS (ESI) *m/z* 364.1693 [$C_{22}H_{26}NO_2Si$(M+H) requires 364.1727]. Chiralpak AD, 25 cm, 97:3 hexanes/^{i}PrOH, 0.5 mL/min, 25 ˚C, 13 bar, t_r (major) = 15.9 min, t_r (minor) = 11.2 min. $[\alpha]_D^{20}$ +39.7 (c 1.00, $CHCl_3$).

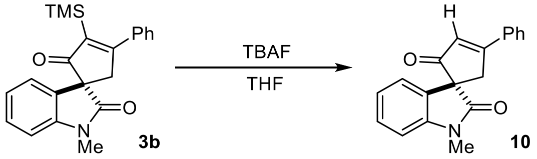

(*R*)-1'-Methyl-4-phenylspiro[cyclopentane-1,3'-indolin]-3-ene-2,2'-dione (10). To a stirring solution of **3b** (18 mg, 0.05 mmol) in THF (0.4 mL) was added dropwise a solution of TBAF (0.1 mL, 1.0

M solution) at –20 ˚C. The reaction mixture stirred for 3 h after which 1.0 mL of saturated ammonium chloride was added. The reaction mixture was diluted with EtOAc (2 mL) and the layers were separated. The organic layer was washed with brine, sodium sulfate and concentrated under reduced pressure. The crude residue was purified by flash chromatography eluting with hexanes/EtOAc (1:1) to provide 15 mg (99%) of **10** in 99% *ee* as a white solid. ^{1}H NMR (500 MHz, $CDCl_3$) δ 7.74-7.72 (m, 2 H), 7.54-7.49 (m, 3 H), 7.32 (comp, 1 H), 7.05-7.03 (m, 2 H), 6.90 (d, *J* = 7.8, 1 H), 6.68 (t, *J* = 1.8, 1 H), 3.71 (dd, *J* = 18, 2 Hz, 1 H), 3.33 (dd, *J* = 18, 2 Hz, 1 H), 3.28 (s, 3 H); ^{13}C NMR (125 MHz, $CDCl_3$) δ 201.7, 174.6, 144.9, 133.2, 132.2, 130.1, 129.2, 129.0, 127.4, 125.3, 123.1, 122.1, 108.8, 62.1, 39.9, 26.9; IR (neat) 2924, 1716, 1693, 1598 cm^{-1}; HRMS (ESI) *m/z* 312.0990 [$C_{19}H_{15}NO_2$(M+Na) requires 312.0994]. m.p. = 185-188 ˚C. Chiralpak AS, 25 cm, 80:20 hexanes/^{i}PrOH, 1.0 mL/min, 25 ˚C, 13 bar, t_r (major) = 35.9 min, t_r (minor) = 30.1 min. $[\alpha]_D^{20}$ +80.6 (c 1.00, $CHCl_3$).

3. Crystal Data for 3b:

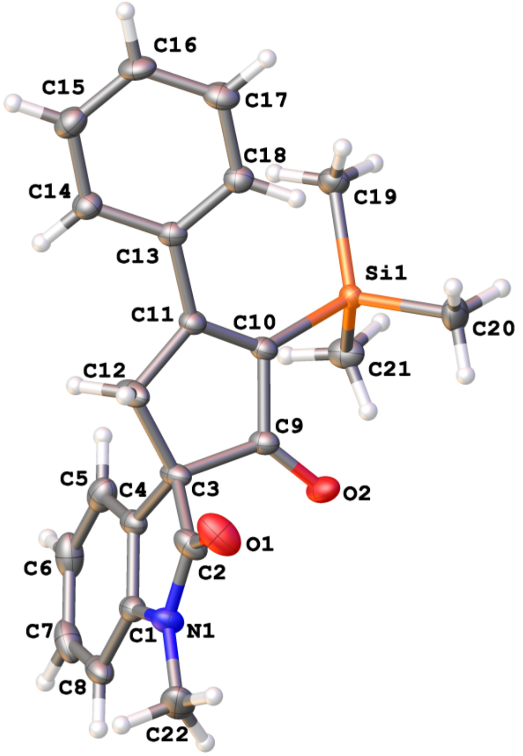

Crystal data for $C_{22}H_{23}NO_2Si$; M_r = 361.50; Orthorhombic; space group $P2_12_12_1$; a = 9.4164(3) Å; b = 11.6775(4) Å; c = 17.2070(6) Å; α = 90°; β = 90°; γ = 90°; V = 1892.08(11) $Å^3$; Z = 4; T = 120(2) K; λ = 1.54178 Å; μ = 1.214 mm^{-1}; d_{calc} = 1.269g.cm^{-3}; 40424 reflections collected; 3643 unique (R_{int} = 0.0290); giving R_1 = 0.0266, wR_2 = 0.0703 for 3638 data with [I>2σ(I)] and R_1 = 0.0266, wR_2 = 0.0703 for all 3643 data. Residual electron density (e^-.$Å^{-3}$) max/min: 0.131/-0.292.

Table 1. Crystal data and structure refinement for **3b**.

Identification code	KR41772	
Empirical formula	C22 H23 N O2 Si	
Formula weight	361.50	
Temperature	120(2) K	
Wavelength	1.54178 Å	
Crystal system	Orthorhombic	
Space group	$P2_12_12_1$	
Unit cell dimensions	a = 9.4164(3) Å	α = 90°
	b = 11.6775(4) Å	β = 90°
	c = 17.2070(6) Å	γ = 90°
Volume	1892.08(11) $Å^3$	

Z	4
Density (calculated)	1.269 g.cm^{-3}
Absorption coefficient (μ)	1.214 mm^{-1}
F(000)	768
Crystal size	0.334 × 0.301 × 0.222 mm^3
θ range for data collection	4.576 to 71.740°
Index ranges	-11 ≤ h ≤ 11, -12 ≤ k ≤ 13, -21 ≤ l ≤ 21
Reflections collected	40424
Independent reflections	3643 [R_{int} = 0.0290]
Completeness to θ = 67.679°	98.5 %
Absorption correction	Semi-empirical from equivalents
Max. and min. transmission	0.7535 and 0.6162
Refinement method	Full-matrix least-squares on F^2
Data / restraints / parameters	3643 / 0 / 239
Goodness-of-fit on F^2	1.103
Final R indices [I>2σ(I)]	R_1 = 0.0266, wR_2 = 0.0703
R indices (all data)	R_1 = 0.0266, wR_2 = 0.0703
Absolute structure parameter	0.048(3)
Extinction coefficient	n/a
Largest diff. peak and hole	0.131 and -0.292 e$^-$.Å^{-3}

Table 2. Atomic coordinates and equivalent isotropic displacement parameters (Å^2) for **3b**. U(eq) is defined as one third of the trace of the orthogonalized U_{ij} tensor.

	x	y	z	U(eq)
Si(1)	0.13678(4)	0.24407(4)	0.73453(3)	0.017(1)
O(1)	0.70659(16)	0.09997(15)	0.76997(10)	0.040(1)
O(2)	0.37734(14)	0.16140(12)	0.84872(7)	0.027(1)
N(1)	0.73230(17)	0.20403(14)	0.88291(9)	0.023(1)
C(1)	0.68153(19)	0.30862(16)	0.91239(10)	0.020(1)
C(2)	0.67722(19)	0.18175(18)	0.81098(11)	0.026(1)
C(3)	0.56926(18)	0.27590(17)	0.79253(10)	0.022(1)
C(4)	0.58633(19)	0.35737(16)	0.85961(10)	0.020(1)
C(5)	0.5218(2)	0.46069(18)	0.87583(12)	0.028(1)
C(6)	0.5550(2)	0.51465(19)	0.94587(14)	0.035(1)
C(7)	0.6509(2)	0.4662(2)	0.99717(12)	0.034(1)

C(8)	0.7166(2)	0.36158(18)	0.98144(11)	0.026(1)
C(9)	0.41737(18)	0.22395(16)	0.79662(10)	0.020(1)
C(10)	0.33595(17)	0.26139(15)	0.72781(9)	0.018(1)
C(11)	0.42786(18)	0.31047(16)	0.67770(10)	0.018(1)
C(12)	0.5767(2)	0.3249(2)	0.71004(10)	0.029(1)
C(13)	0.39902(18)	0.34380(15)	0.59652(10)	0.018(1)
C(14)	0.4699(2)	0.43522(17)	0.56111(11)	0.023(1)
C(15)	0.4421(2)	0.46315(18)	0.48407(11)	0.027(1)
C(16)	0.3440(2)	0.40072(18)	0.44141(11)	0.027(1)
C(17)	0.2750(2)	0.30869(18)	0.47572(11)	0.026(1)
C(18)	0.30294(19)	0.27965(16)	0.55227(10)	0.021(1)
C(19)	0.0334(2)	0.32505(18)	0.66005(11)	0.026(1)
C(20)	0.0947(2)	0.08845(16)	0.73137(13)	0.031(1)
C(21)	0.0906(2)	0.31043(18)	0.83009(11)	0.027(1)
C(22)	0.8314(2)	0.1300(2)	0.92330(14)	0.034(1)
H(5)	0.4566	0.4942	0.8403	0.034
H(6)	0.5111	0.5855	0.9585	0.042
H(7)	0.6726	0.5051	1.0442	0.041
H(8)	0.7825	0.3284	1.0167	0.032
H(12A)	0.6041	0.4067	0.7111	0.035
H(12B)	0.6465	0.2822	0.6782	0.035
H(14)	0.5374	0.4784	0.5898	0.028
H(15)	0.4906	0.5254	0.4605	0.033
H(16)	0.3242	0.4208	0.3890	0.033
H(17)	0.2082	0.2654	0.4466	0.031
H(18)	0.2565	0.2156	0.5749	0.026
H(19A)	0.0709	0.4031	0.6559	0.039
H(19B)	-0.0667	0.3280	0.6755	0.039
H(19C)	0.0418	0.2866	0.6096	0.039
H(20A)	-0.0078	0.0776	0.7374	0.046
H(20B)	0.1447	0.0492	0.7736	0.046
H(20C)	0.1253	0.0566	0.6814	0.046
H(21A)	0.1334	0.3868	0.8335	0.041
H(21B)	0.1269	0.2624	0.8723	0.041
H(21C)	-0.0129	0.3170	0.8346	0.041
H(22A)	0.7910	0.1072	0.9734	0.052
H(22B)	0.9208	0.1711	0.9319	0.052

H(22C)	0.8496	0.0616	0.8918	0.052

Table 3. Anisotropic displacement parameters ($Å^2$) for **3b**.
The anisotropic displacement factor exponent takes the form:
$-2\pi^2[h^2a^{*2}U_{11} + ... + 2hka^*b^*U_{12}]$

	U_{11}	U_{22}	U_{33}	U_{23}	U_{13}	U_{12}
Si(1)	0.0142(2)	0.0180(2)	0.0191(2)	0.0015(2)	-0.0009(2)	-0.0014(2)
O(1)	0.0269(7)	0.0485(9)	0.0440(9)	-0.0207(8)	0.0009(7)	0.0058(7)
O(2)	0.0225(7)	0.0383(8)	0.0198(6)	0.0097(5)	0.0006(5)	0.0007(6)
N(1)	0.0180(7)	0.0268(8)	0.0248(7)	0.0003(6)	-0.0044(6)	0.0039(6)
C(1)	0.0164(8)	0.0257(9)	0.0184(8)	0.0036(7)	0.0010(6)	-0.0027(7)
C(2)	0.0163(8)	0.0354(11)	0.0250(9)	-0.0054(8)	-0.0002(7)	0.0005(8)
C(3)	0.0154(8)	0.0359(11)	0.0154(8)	0.0013(7)	-0.0003(6)	0.0005(7)
C(4)	0.0156(8)	0.0273(10)	0.0174(8)	0.0029(7)	0.0006(6)	-0.0019(7)
C(5)	0.0214(9)	0.0277(11)	0.0361(10)	0.0079(8)	0.0035(8)	0.0003(8)
C(6)	0.0283(11)	0.0250(11)	0.0505(13)	-0.0072(9)	0.0120(9)	-0.0054(8)
C(7)	0.0325(11)	0.0396(12)	0.0314(10)	-0.0122(9)	0.0083(9)	-0.0169(9)
C(8)	0.0236(9)	0.0370(11)	0.0185(8)	0.0010(8)	-0.0014(7)	-0.0098(8)
C(9)	0.0164(8)	0.0270(10)	0.0161(8)	0.0002(7)	0.0005(7)	0.0022(7)
C(10)	0.0175(8)	0.0205(8)	0.0155(8)	-0.0015(7)	-0.0016(6)	0.0002(6)
C(11)	0.0167(8)	0.0223(9)	0.0154(8)	-0.0011(6)	-0.0005(6)	0.0005(7)
C(12)	0.0167(8)	0.0540(13)	0.0160(8)	0.0053(8)	-0.0004(7)	-0.0064(9)
C(13)	0.0167(8)	0.0233(9)	0.0145(8)	-0.0001(6)	0.0011(6)	0.0034(7)
C(14)	0.0211(9)	0.0271(10)	0.0210(9)	0.0010(7)	0.0005(7)	-0.0013(7)
C(15)	0.0273(10)	0.0309(10)	0.0243(9)	0.0071(8)	0.0032(8)	0.0014(8)
C(16)	0.0299(10)	0.0377(11)	0.0139(8)	0.0035(7)	0.0007(7)	0.0060(8)
C(17)	0.0262(9)	0.0332(10)	0.0177(8)	-0.0041(7)	-0.0023(7)	0.0026(8)
C(18)	0.0224(8)	0.0234(9)	0.0182(8)	-0.0014(7)	0.0010(7)	0.0017(7)
C(19)	0.0192(8)	0.0299(11)	0.0287(9)	0.0036(8)	-0.0031(7)	0.0028(7)
C(20)	0.0295(10)	0.0212(9)	0.0420(11)	0.0041(8)	-0.0047(9)	-0.0058(8)
C(21)	0.0230(9)	0.0358(11)	0.0232(9)	-0.0004(8)	0.0043(7)	0.0050(8)
C(22)	0.0230(10)	0.0336(12)	0.0469(12)	0.0105(9)	-0.0085(9)	0.0040(8)

Table 4. Bond lengths [Å] for **3b**.

atom-atom	distance	atom-atom	distance	
Si(1)-C(20)	1.861(2)	Si(1)-C(19)	1.8666(19)	Si(1)-
C(21)	1.8691(19)	Si(1)-C(10)	1.8899(17)	O(1)-
C(2)	1.219(2)	O(2)-C(9)	1.216(2)	N(1)-
C(2)	1.367(2)	N(1)-C(1)	1.406(2)	N(1)-
C(22)	1.450(2)	C(1)-C(8)	1.380(3)	C(1)-
C(4)	1.397(3)	C(2)-C(3)	1.531(3)	C(3)-
C(4)	1.504(2)	C(3)-C(12)	1.532(2)	C(3)-
C(9)	1.555(2)	C(4)-C(5)	1.379(3)	C(5)-
C(6)	1.395(3)	C(5)-H(5)	0.9500	C(6)-
C(7)	1.384(3)	C(6)-H(6)	0.9500	C(7)-
C(8)	1.396(3)	C(7)-H(7)	0.9500	C(8)-
H(8)	0.9500	C(9)-C(10)	1.477(2)	C(10)-
C(11)	1.349(2)	C(11)-C(13)	1.475(2)	C(11)-
C(12)	1.517(2)	C(12)-H(12A)	0.9900	C(12)-
H(12B)	0.9900	C(13)-C(14)	1.399(3)	C(13)-
C(18)	1.400(2)	C(14)-C(15)	1.390(3)	C(14)-
H(14)	0.9500	C(15)-C(16)	1.387(3)	C(15)-
H(15)	0.9500	C(16)-C(17)	1.388(3)	C(16)-
H(16)	0.9500	C(17)-C(18)	1.385(3)	C(17)-
H(17)	0.9500	C(18)-H(18)	0.9500	C(19)-
H(19A)	0.9800	C(19)-H(19B)	0.9800	C(19)-
H(19C)	0.9800	C(20)-H(20A)	0.9800	C(20)-
H(20B)	0.9800	C(20)-H(20C)	0.9800	C(21)-
H(21A)	0.9800	C(21)-H(21B)	0.9800	C(21)-
H(21C)	0.9800	C(22)-H(22A)	0.9800	C(22)-
H(22B)	0.9800	C(22)-H(22C)	0.9800	

Symmetry transformations used to generate equivalent atoms:

Table 5. Bond angles [°] for **3b**.

atom-atom-atom	angle	atom-atom-atom	angle	
C(20)-Si(1)-C(19)	111.33(10)	C(20)-Si(1)-C(21)	112.40(10)	C(19)-
Si(1)-C(21)	105.81(9)	C(20)-Si(1)-C(10)	108.29(9)	C(19)-
Si(1)-C(10)	114.92(8)	C(21)-Si(1)-C(10)	103.92(8)	C(2)-
N(1)-C(1)	111.27(15)	C(2)-N(1)-C(22)	124.39(18)	C(1)-
N(1)-C(22)	124.33(17)	C(8)-C(1)-C(4)	122.07(19)	C(8)-
C(1)-N(1)	128.20(18)	C(4)-C(1)-N(1)	109.72(16)	O(1)-
C(2)-N(1)	125.94(19)	O(1)-C(2)-C(3)	126.38(18)	N(1)-
C(2)-C(3)	107.65(16)	C(4)-C(3)-C(2)	102.95(14)	C(4)-
C(3)-C(12)	118.02(17)	C(2)-C(3)-C(12)	115.47(16)	C(4)-
C(3)-C(9)	108.07(14)	C(2)-C(3)-C(9)	108.74(15)	C(12)-

C(3)-C(9)	103.28(14)	C(5)-C(4)-C(1)	120.49(18)	C(5)-
C(4)-C(3)	131.40(17)	C(1)-C(4)-C(3)	108.04(16)	C(4)-
C(5)-C(6)	118.11(19)	C(4)-C(5)-H(5)	120.9	C(6)-
C(5)-H(5)	120.9	C(7)-C(6)-C(5)	120.8(2)	C(7)-
C(6)-H(6)	119.6	C(5)-C(6)-H(6)	119.6	C(6)-
C(7)-C(8)	121.55(19)	C(6)-C(7)-H(7)	119.2	C(8)-
C(7)-H(7)	119.2	C(1)-C(8)-C(7)	116.94(19)	C(1)-
C(8)-H(8)	121.5	C(7)-C(8)-H(8)	121.5	O(2)-
C(9)-C(10)	127.44(16)	O(2)-C(9)-C(3)	123.55(16)	C(10)-
C(9)-C(3)	109.01(14)	C(11)-C(10)-C(9)	107.77(14)	C(11)-
C(10)-Si(1)	136.14(13)	C(9)-C(10)-Si(1)	115.75(12)	C(10)-
C(11)-C(13)	126.80(16)	C(10)-C(11)-C(12)	113.90(15)	C(13)-
C(11)-C(12)	119.21(15)	C(11)-C(12)-C(3)	104.84(15)	C(11)-
C(12)-H(12A)	110.8	C(3)-C(12)-H(12A)	110.8	C(11)-
C(12)-H(12B)	110.8	C(3)-C(12)-H(12B)	110.8	H(12A)-
C(12)-H(12B)	108.9	C(14)-C(13)-C(18)	118.69(16)	C(14)-
C(13)-C(11)	121.73(16)	C(18)-C(13)-C(11)	119.53(16)	C(15)-
C(14)-C(13)	120.28(18)	C(15)-C(14)-H(14)	119.9	C(13)-
C(14)-H(14)	119.9	C(16)-C(15)-C(14)	120.48(18)	C(16)-
C(15)-H(15)	119.8	C(14)-C(15)-H(15)	119.8	C(15)-
C(16)-C(17)	119.59(17)	C(15)-C(16)-H(16)	120.2	C(17)-
C(16)-H(16)	120.2	C(18)-C(17)-C(16)	120.32(18)	C(18)-
C(17)-H(17)	119.8	C(16)-C(17)-H(17)	119.8	C(17)-
C(18)-C(13)	120.60(18)	C(17)-C(18)-H(18)	119.7	C(13)-
C(18)-H(18)	119.7	Si(1)-C(19)-H(19A)	109.5	Si(1)-
C(19)-H(19B)	109.5	H(19A)-C(19)-H(19B)	109.5	Si(1)-
C(19)-H(19C)	109.5	H(19A)-C(19)-H(19C)	109.5	H(19B)-
C(19)-H(19C)	109.5	Si(1)-C(20)-H(20A)	109.5	Si(1)-
C(20)-H(20B)	109.5	H(20A)-C(20)-H(20B)	109.5	Si(1)-
C(20)-H(20C)	109.5	H(20A)-C(20)-H(20C)	109.5	H(20B)-
C(20)-H(20C)	109.5	Si(1)-C(21)-H(21A)	109.5	Si(1)-
C(21)-H(21B)	109.5	H(21A)-C(21)-H(21B)	109.5	Si(1)-
C(21)-H(21C)	109.5	H(21A)-C(21)-H(21C)	109.5	H(21B)-
C(21)-H(21C)	109.5	N(1)-C(22)-H(22A)	109.5	N(1)-
C(22)-H(22B)	109.5	H(22A)-C(22)-H(22B)	109.5	N(1)-
C(22)-H(22C)	109.5	H(22A)-C(22)-H(22C)	109.5	H(22B)-
C(22)-H(22C)	109.5			

Symmetry transformations used to generate equivalent atoms:

Table 6. Torsion angles [°] for **3b**.

atom-atom-atom-atom	angle	atom-atom-atom-atom	angle	
C(2)-N(1)-C(1)-C(8)	-177.51(18)	C(22)-N(1)-C(1)-C(8)	1.9(3)	C(2)-
N(1)-C(1)-C(4)	1.5(2)	C(22)-N(1)-C(1)-C(4)	-179.09(18)	C(1)-
N(1)-C(2)-O(1)	176.8(2)	C(22)-N(1)-C(2)-O(1)	-2.6(3)	C(1)-
N(1)-C(2)-C(3)	-4.9(2)	C(22)-N(1)-C(2)-C(3)	175.72(17)	O(1)-
C(2)-C(3)-C(4)	-175.6(2)	N(1)-C(2)-C(3)-C(4)	6.06(19)	O(1)-
C(2)-C(3)-C(12)	-45.5(3)	N(1)-C(2)-C(3)-C(12)	136.12(17)	O(1)-
C(2)-C(3)-C(9)	70.0(2)	N(1)-C(2)-C(3)-C(9)	-108.40(16)	C(8)-
C(1)-C(4)-C(5)	-1.1(3)	N(1)-C(1)-C(4)-C(5)	179.83(16)	C(8)-
C(1)-C(4)-C(3)	-178.30(16)	N(1)-C(1)-C(4)-C(3)	2.6(2)	C(2)-
C(3)-C(4)-C(5)	178.02(19)	C(12)-C(3)-C(4)-C(5)	49.5(3)	C(9)-
C(3)-C(4)-C(5)	-67.0(2)	C(2)-C(3)-C(4)-C(1)	-5.18(19)	C(12)-
C(3)-C(4)-C(1)	-133.66(17)	C(9)-C(3)-C(4)-C(1)	109.76(17)	C(1)-
C(4)-C(5)-C(6)	0.3(3)	C(3)-C(4)-C(5)-C(6)	176.76(19)	C(4)-
C(5)-C(6)-C(7)	0.6(3)	C(5)-C(6)-C(7)-C(8)	-0.8(3)	C(4)-
C(1)-C(8)-C(7)	0.9(3)	N(1)-C(1)-C(8)-C(7)	179.82(17)	C(6)-
C(7)-C(8)-C(1)	0.0(3)	C(4)-C(3)-C(9)-O(2)	-65.9(2)	C(2)-
C(3)-C(9)-O(2)	45.2(2)	C(12)-C(3)-C(9)-O(2)	168.35(19)	C(4)-
C(3)-C(9)-C(10)	115.17(16)	C(2)-C(3)-C(9)-C(10)	-133.75(15)	C(12)-
C(3)-C(9)-C(10)	-10.6(2)	O(2)-C(9)-C(10)-C(11)	-167.89(19)	C(3)-
C(9)-C(10)-C(11)	11.0(2)	O(2)-C(9)-C(10)-Si(1)	17.8(3)	C(3)-
C(9)-C(10)-Si(1)	-163.30(12)	C(20)-Si(1)-C(10)-C(11)	117.7(2)	C(19)-
Si(1)-C(10)-C(11)	-7.4(2)	C(21)-Si(1)-C(10)-C(11)	-122.6(2)	C(20)-
Si(1)-C(10)-C(9)	-70.06(15)	C(19)-Si(1)-C(10)-C(9)	164.77(13)	C(21)-
Si(1)-C(10)-C(9)	49.64(15)	C(9)-C(10)-C(11)-C(13)	169.61(17)	Si(1)-
C(10)-C(11)-C(13)	-17.8(3)	C(9)-C(10)-C(11)-C(12)	-6.9(2)	Si(1)-
C(10)-C(11)-C(12)	165.73(16)	C(10)-C(11)-C(12)-C(3)	0.0(2)	C(13)-
C(11)-C(12)-C(3)	-176.78(16)	C(4)-C(3)-C(12)-C(11)	-112.74(19)	C(2)-
C(3)-C(12)-C(11)	124.94(18)	C(9)-C(3)-C(12)-C(11)	6.4(2)	C(10)-
C(11)-C(13)-C(14)	150.64(19)	C(12)-C(11)-C(13)-C(14)	-33.0(3)	C(10)-
C(11)-C(13)-C(18)	-32.2(3)	C(12)-C(11)-C(13)-C(18)	144.11(18)	C(18)-
C(13)-C(14)-C(15)	1.7(3)	C(11)-C(13)-C(14)-C(15)	178.87(17)	C(13)-
C(14)-C(15)-C(16)	-0.1(3)	C(14)-C(15)-C(16)-C(17)	-1.0(3)	C(15)-
C(16)-C(17)-C(18)	0.4(3)	C(16)-C(17)-C(18)-C(13)	1.2(3)	C(14)-
C(13)-C(18)-C(17)	-2.3(3)	C(11)-C(13)-C(18)-C(17)	-179.48(17)	

Symmetry transformations used to generate equivalent atoms:

4. CRYSTAL DATA FOR 4A:

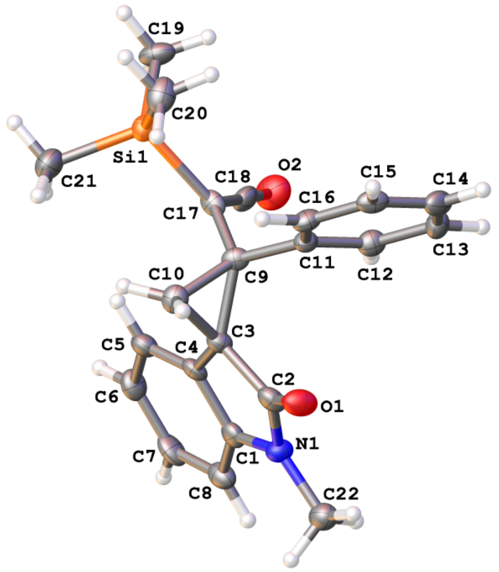

Crystal data for $C_{22}H_{23}NO_2$ Si; M_r = 361.50; Monoclinic; space group $P2_1$; *a* = 10.4794(8) Å; *b* = 7.0464(6) Å; *c* = 13.4791(10) Å; α = 90°; β = 100.094(5)°; γ = 90°; V = 979.92(13) $Å^3$; Z = 2; T = 120(2) K; λ = 1.54178 Å; μ = 1.173 mm^{-1}; d_{calc} = 1.235g.cm^{-3}; 21771 reflections collected; 3869 unique (R_{int} = 0.0979); giving R_1 = 0.0596, wR_2 = 0.1657 for 3398 data with [I>2σ(I)] and R_1 = 0.0774, wR_2 = 0.2024 for all 3869 data. Residual electron density (e^-.$Å^{-3}$) max/min: 0.231/-0.514.

Table 1. Crystal data and structure refinement for **4A**.

Identification code	kr565-a	
Empirical formula	C_{22} H_{23} N O_2 Si	
Formula weight	361.50	
Temperature	120(2) K	
Wavelength	1.54178 Å	
Crystal system	Monoclinic	
Space group	$P2_1$	
Unit cell dimensions	*a* = 10.4794(8) Å	α = 90°
	b = 7.0464(6) Å	β = 100.094(5)°
	c = 13.4791(10) Å	γ = 90°
Volume	979.92(13) $Å^3$	
Z	2	
Density (calculated)	1.235 g.cm^{-3}	

Absorption coefficient (μ)	1.173 mm^{-1}
F(000)	390
Crystal size	0.198 × 0.175 × 0.118 mm^3
θ range for data collection	3.330 to 72.241°
Index ranges	$-12 \leq h \leq 12$, $-8 \leq k \leq 8$, $-16 \leq l \leq 16$
Reflections collected	21771
Independent reflections	3869 [R_{int} = 0.0979]
Completeness to θ = 67.679°	99.8 %
Absorption correction	Semi-empirical from equivalents
Refinement method	Full-matrix least-squares on F^2
Data / restraints / parameters	3869 / 1 / 239
Goodness-of-fit on F^2	1.269
Final R indices [I>2σ(I)]	R_1 = 0.0596, wR_2 = 0.1657
R indices (all data)	R_1 = 0.0774, wR_2 = 0.2024
Absolute structure parameter	-0.01(4)
Extinction coefficient	n/a
Largest diff. peak and hole	0.231 and -0.514 $e^-.Å^{-3}$

Table 2. Atomic coordinates and equivalent isotropic displacement parameters ($Å^2$) for **4A**. U(eq) is defined as one third of the trace of the orthogonalized U_{ij} tensor.

	x	y	z	U(eq)
Si(1)	0.43987(13)	0.5779(2)	0.17725(10)	0.032(1)
O(1)	1.0223(4)	0.7060(6)	0.2383(3)	0.041(1)
O(2)	0.6056(4)	0.2012(7)	0.3387(3)	0.049(1)
N(1)	1.0238(4)	0.6380(7)	0.4066(3)	0.034(1)
C(1)	0.9330(5)	0.6305(7)	0.4700(4)	0.033(1)
C(2)	0.9658(5)	0.6758(8)	0.3081(4)	0.035(1)
C(3)	0.8222(5)	0.6779(8)	0.3079(4)	0.033(1)
C(4)	0.8068(5)	0.6556(8)	0.4151(4)	0.033(1)
C(5)	0.7024(5)	0.6583(8)	0.4633(4)	0.036(1)
C(6)	0.7220(6)	0.6316(8)	0.5684(4)	0.039(1)
C(7)	0.8477(6)	0.6082(9)	0.6219(4)	0.042(1)
C(8)	0.9539(6)	0.6074(8)	0.5743(4)	0.038(1)
C(9)	0.7292(5)	0.5870(8)	0.2177(3)	0.030(1)
C(10)	0.7387(5)	0.7981(8)	0.2276(4)	0.035(1)

C(11)	0.7913(5)	0.4849(8)	0.1391(4)	0.031(1)
C(12)	0.8452(5)	0.3063(9)	0.1613(4)	0.036(1)
C(13)	0.8981(6)	0.2062(9)	0.0900(4)	0.040(1)
C(14)	0.8974(5)	0.2830(10)	-0.0059(4)	0.039(1)
C(15)	0.8435(5)	0.4609(9)	-0.0274(4)	0.038(1)
C(16)	0.7906(5)	0.5631(9)	0.0444(4)	0.035(1)
C(17)	0.6060(5)	0.4939(8)	0.2381(4)	0.030(1)
C(18)	0.6091(5)	0.3391(8)	0.2914(4)	0.034(1)
C(19)	0.3235(6)	0.3836(10)	0.1854(5)	0.044(1)
C(20)	0.4480(6)	0.6399(10)	0.0447(5)	0.046(2)
C(21)	0.3905(6)	0.7882(10)	0.2453(5)	0.045(1)
C(22)	1.1630(5)	0.6300(8)	0.4398(4)	0.039(1)
H(5)	0.6175	0.6779	0.4263	0.044
H(6)	0.6502	0.6296	0.6028	0.047
H(7)	0.8603	0.5924	0.6929	0.051
H(8)	1.0389	0.5915	0.6116	0.045
H(10A)	0.6642	0.8662	0.2471	0.042
H(10B)	0.7831	0.8672	0.1795	0.042
H(12)	0.8459	0.2526	0.2260	0.043
H(13)	0.9351	0.0846	0.1062	0.048
H(14)	0.9332	0.2144	-0.0552	0.047
H(15)	0.8425	0.5144	-0.0922	0.046
H(16)	0.7545	0.6853	0.0285	0.042
H(19A)	0.3529	0.2680	0.1557	0.066
H(19B)	0.2379	0.4195	0.1485	0.066
H(19C)	0.3181	0.3601	0.2562	0.066
H(20A)	0.5183	0.7311	0.0434	0.069
H(20B)	0.3656	0.6964	0.0127	0.069
H(20C)	0.4645	0.5249	0.0080	0.069
H(21A)	0.4049	0.7619	0.3178	0.068
H(21B)	0.2985	0.8149	0.2215	0.068
H(21C)	0.4423	0.8984	0.2324	0.068
H(22A)	1.2063	0.6305	0.3810	0.058
H(22B)	1.1855	0.5136	0.4787	0.058
H(22C)	1.1912	0.7405	0.4822	0.058

Table 3. Anisotropic displacement parameters ($Å^2$) for kr565-a.

The anisotropic displacement factor exponent takes the form:
$-2\pi^2[h^2a^{*2}U_{11} + ... + 2hka^*b^*U_{12}]$

	U_{11}	U_{22}	U_{33}	U_{23}	U_{13}	U_{12}
Si(1)	0.0311(7)	0.0320(7)	0.0330(7)	0.0008(6)	0.0048(5)	0.0013(6)
O(1)	0.037(2)	0.050(2)	0.0369(19)	-0.0038(18)	0.0090(15)	-0.0091(18)
O(2)	0.048(2)	0.040(2)	0.058(2)	0.013(2)	0.0083(19)	-0.0018(19)
N(1)	0.033(2)	0.032(2)	0.036(2)	-0.0006(18)	0.0021(17)	-0.0017(17)
C(1)	0.040(3)	0.022(2)	0.037(3)	-0.0027(19)	0.004(2)	-0.0034(19)
C(2)	0.039(3)	0.031(3)	0.034(2)	-0.004(2)	0.005(2)	-0.008(2)
C(3)	0.037(3)	0.034(3)	0.029(2)	-0.003(2)	0.0069(19)	-0.005(2)
C(4)	0.039(3)	0.028(2)	0.031(2)	-0.004(2)	0.006(2)	-0.005(2)
C(5)	0.041(3)	0.033(3)	0.035(3)	-0.005(2)	0.008(2)	-0.005(2)
C(6)	0.051(3)	0.038(3)	0.032(3)	-0.001(2)	0.017(2)	-0.004(2)
C(7)	0.059(3)	0.038(3)	0.030(2)	-0.003(2)	0.009(2)	-0.005(3)
C(8)	0.046(3)	0.031(3)	0.034(2)	0.000(2)	0.001(2)	0.000(2)
C(9)	0.030(2)	0.031(2)	0.027(2)	0.000(2)	0.0013(17)	0.000(2)
C(10)	0.041(3)	0.033(3)	0.034(3)	-0.001(2)	0.008(2)	-0.001(2)
C(11)	0.030(2)	0.033(3)	0.029(2)	-0.004(2)	0.0028(18)	-0.005(2)
C(12)	0.035(3)	0.039(3)	0.032(2)	0.002(2)	0.004(2)	0.001(2)
C(13)	0.037(3)	0.044(3)	0.040(3)	0.000(2)	0.008(2)	0.000(2)
C(14)	0.031(3)	0.049(3)	0.040(3)	-0.008(3)	0.013(2)	-0.004(2)
C(15)	0.032(3)	0.052(4)	0.029(2)	0.000(2)	0.004(2)	-0.011(2)
C(16)	0.035(2)	0.039(3)	0.029(2)	0.002(2)	0.0039(19)	-0.006(2)
C(17)	0.030(2)	0.029(2)	0.030(2)	-0.0010(19)	0.0033(18)	0.004(2)
C(18)	0.030(2)	0.032(3)	0.039(3)	0.002(2)	0.002(2)	0.004(2)
C(19)	0.033(3)	0.050(4)	0.046(3)	0.006(3)	0.001(2)	-0.009(3)
C(20)	0.046(3)	0.051(4)	0.043(3)	0.011(3)	0.013(2)	0.015(3)
C(21)	0.041(3)	0.046(3)	0.049(3)	-0.011(3)	0.010(3)	0.011(3)
C(22)	0.032(3)	0.037(3)	0.047(3)	0.000(2)	0.002(2)	-0.002(2)

Table 4. Bond lengths [Å] for **4A**.

atom-atom	distance	atom-atom	distance	
Si(1)-C(19)	1.850(6)	Si(1)-C(20)	1.856(6)	Si(1)-
C(21)	1.863(6)	Si(1)-C(17)	1.885(5)	O(1)-

C(2)	1.215(7)	O(2)-C(18)	1.167(7)	N(1)-
C(2)	1.385(7)	N(1)-C(1)	1.387(7)	N(1)-
C(22)	1.450(6)	C(1)-C(8)	1.395(7)	C(1)-
C(4)	1.409(7)	C(2)-C(3)	1.505(8)	C(3)-
C(4)	1.490(7)	C(3)-C(10)	1.523(7)	C(3)-
C(9)	1.556(7)	C(4)-C(5)	1.367(8)	C(5)-
C(6)	1.409(7)	C(5)-H(5)	0.9500	C(6)-
C(7)	1.395(8)	C(6)-H(6)	0.9500	C(7)-
C(8)	1.378(8)	C(7)-H(7)	0.9500	C(8)-
H(8)	0.9500	C(9)-C(10)	1.495(8)	C(9)-
C(17)	1.516(7)	C(9)-C(11)	1.517(7)	C(10)-
H(10A)	0.9900	C(10)-H(10B)	0.9900	C(11)-
C(16)	1.390(7)	C(11)-C(12)	1.390(8)	C(12)-
C(13)	1.384(8)	C(12)-H(12)	0.9500	C(13)-
C(14)	1.400(8)	C(13)-H(13)	0.9500	C(14)-
C(15)	1.384(9)	C(14)-H(14)	0.9500	C(15)-
C(16)	1.396(8)	C(15)-H(15)	0.9500	C(16)-
H(16)	0.9500	C(17)-C(18)	1.303(8)	C(19)-
H(19A)	0.9800	C(19)-H(19B)	0.9800	C(19)-
H(19C)	0.9800	C(20)-H(20A)	0.9800	C(20)-
H(20B)	0.9800	C(20)-H(20C)	0.9800	C(21)-
H(21A)	0.9800	C(21)-H(21B)	0.9800	C(21)-
H(21C)	0.9800	C(22)-H(22A)	0.9800	C(22)-
H(22B)	0.9800	C(22)-H(22C)	0.9800	

Symmetry transformations used to generate equivalent atoms:

Table 5. Bond angles [°] for **4A**.

atom-atom-atom	angle	atom-atom-atom	angle	
C(19)-Si(1)-C(20)	111.9(3)	C(19)-Si(1)-C(21)	108.5(3)	C(20)-
Si(1)-C(21)	110.5(3)	C(19)-Si(1)-C(17)	108.1(3)	C(20)-
Si(1)-C(17)	107.3(3)	C(21)-Si(1)-C(17)	110.5(2)	C(2)-
N(1)-C(1)	111.4(4)	C(2)-N(1)-C(22)	123.5(5)	C(1)-
N(1)-C(22)	124.7(4)	N(1)-C(1)-C(8)	128.5(5)	N(1)-
C(1)-C(4)	110.7(4)	C(8)-C(1)-C(4)	120.7(5)	O(1)-
C(2)-N(1)	125.8(5)	O(1)-C(2)-C(3)	128.3(5)	N(1)-
C(2)-C(3)	105.9(5)	C(4)-C(3)-C(2)	106.0(4)	C(4)-
C(3)-C(10)	126.9(5)	C(2)-C(3)-C(10)	117.1(5)	C(4)-
C(3)-C(9)	123.7(5)	C(2)-C(3)-C(9)	119.1(4)	C(10)-
C(3)-C(9)	58.1(3)	C(5)-C(4)-C(1)	120.5(5)	C(5)-
C(4)-C(3)	133.7(5)	C(1)-C(4)-C(3)	105.7(5)	C(4)-
C(5)-C(6)	119.2(5)	C(4)-C(5)-H(5)	120.4	C(6)-
C(5)-H(5)	120.4	C(7)-C(6)-C(5)	119.6(5)	C(7)-
C(6)-H(6)	120.2	C(5)-C(6)-H(6)	120.2	C(8)-
C(7)-C(6)	121.8(5)	C(8)-C(7)-H(7)	119.1	C(6)-
C(7)-H(7)	119.1	C(7)-C(8)-C(1)	118.2(5)	C(7)-
C(8)-H(8)	120.9	C(1)-C(8)-H(8)	120.9	C(10)-

C(9)-C(17)	117.4(5)	C(10)-C(9)-C(11)	120.3(5)	C(17)-
C(9)-C(11)	113.9(5)	C(10)-C(9)-C(3)	59.9(3)	C(17)-
C(9)-C(3)	118.2(4)	C(11)-C(9)-C(3)	117.0(4)	C(9)-
C(10)-C(3)	62.1(3)	C(9)-C(10)-H(10A)	117.6	C(3)-
C(10)-H(10A)	117.6	C(9)-C(10)-H(10B)	117.6	C(3)-
C(10)-H(10B)	117.6	H(10A)-C(10)-H(10B)	114.6	C(16)-
C(11)-C(12)	119.5(5)	C(16)-C(11)-C(9)	121.3(5)	C(12)-
C(11)-C(9)	119.2(5)	C(13)-C(12)-C(11)	120.7(5)	C(13)-
C(12)-H(12)	119.7	C(11)-C(12)-H(12)	119.7	C(12)-
C(13)-C(14)	120.4(6)	C(12)-C(13)-H(13)	119.8	C(14)-
C(13)-H(13)	119.8	C(15)-C(14)-C(13)	118.6(6)	C(15)-
C(14)-H(14)	120.7	C(13)-C(14)-H(14)	120.7	C(14)-
C(15)-C(16)	121.3(5)	C(14)-C(15)-H(15)	119.4	C(16)-
C(15)-H(15)	119.4	C(11)-C(16)-C(15)	119.6(6)	C(11)-
C(16)-H(16)	120.2	C(15)-C(16)-H(16)	120.2	C(18)-
C(17)-C(9)	121.5(5)	C(18)-C(17)-Si(1)	115.7(4)	C(9)-
C(17)-Si(1)	122.4(4)	O(2)-C(18)-C(17)	176.8(6)	Si(1)-
C(19)-H(19A)	109.5	Si(1)-C(19)-H(19B)	109.5	H(19A)-
C(19)-H(19B)	109.5	Si(1)-C(19)-H(19C)	109.5	H(19A)-
C(19)-H(19C)	109.5	H(19B)-C(19)-H(19C)	109.5	Si(1)-
C(20)-H(20A)	109.5	Si(1)-C(20)-H(20B)	109.5	H(20A)-
C(20)-H(20B)	109.5	Si(1)-C(20)-H(20C)	109.5	H(20A)-
C(20)-H(20C)	109.5	H(20B)-C(20)-H(20C)	109.5	Si(1)-
C(21)-H(21A)	109.5	Si(1)-C(21)-H(21B)	109.5	H(21A)-
C(21)-H(21B)	109.5	Si(1)-C(21)-H(21C)	109.5	H(21A)-
C(21)-H(21C)	109.5	H(21B)-C(21)-H(21C)	109.5	N(1)-
C(22)-H(22A)	109.5	N(1)-C(22)-H(22B)	109.5	H(22A)-
C(22)-H(22B)	109.5	N(1)-C(22)-H(22C)	109.5	H(22A)-
C(22)-H(22C)	109.5	H(22B)-C(22)-H(22C)	109.5	

Symmetry transformations used to generate equivalent atoms:

Table 6. Torsion angles [°] for **4A**.

atom-atom-atom-atom	angle	atom-atom-atom-atom	angle	
C(2)-N(1)-C(1)-C(8)	-175.8(5)	C(22)-N(1)-C(1)-C(8)	-2.6(9)	C(2)-
N(1)-C(1)-C(4)	2.5(6)	C(22)-N(1)-C(1)-C(4)	175.7(5)	C(1)-
N(1)-C(2)-O(1)	173.5(6)	C(22)-N(1)-C(2)-O(1)	0.2(9)	C(1)-
N(1)-C(2)-C(3)	-4.6(6)	C(22)-N(1)-C(2)-C(3)	-177.9(5)	O(1)-
C(2)-C(3)-C(4)	-173.1(6)	N(1)-C(2)-C(3)-C(4)	4.9(6)	O(1)-
C(2)-C(3)-C(10)	-25.0(8)	N(1)-C(2)-C(3)-C(10)	153.1(5)	O(1)-
C(2)-C(3)-C(9)	41.8(9)	N(1)-C(2)-C(3)-C(9)	-140.2(5)	N(1)-
C(1)-C(4)-C(5)	-178.2(5)	C(8)-C(1)-C(4)-C(5)	0.2(8)	N(1)-
C(1)-C(4)-C(3)	0.8(6)	C(8)-C(1)-C(4)-C(3)	179.2(5)	C(2)-
C(3)-C(4)-C(5)	175.4(6)	C(10)-C(3)-C(4)-C(5)	31.4(10)	C(9)-
C(3)-C(4)-C(5)	-41.6(10)	C(2)-C(3)-C(4)-C(1)	-3.5(6)	C(10)-
C(3)-C(4)-C(1)	-147.5(5)	C(9)-C(3)-C(4)-C(1)	139.6(5)	C(1)-
C(4)-C(5)-C(6)	-1.3(8)	C(3)-C(4)-C(5)-C(6)	179.9(6)	C(4)-

C(5)-C(6)-C(7)	1.8(9)	C(5)-C(6)-C(7)-C(8)	-1.0(9)	C(6)-
C(7)-C(8)-C(1)	-0.1(9)	N(1)-C(1)-C(8)-C(7)	178.7(5)	C(4)-
C(1)-C(8)-C(7)	0.5(8)	C(4)-C(3)-C(9)-C(10)	115.8(6)	C(2)-
C(3)-C(9)-C(10)	-105.6(6)	C(4)-C(3)-C(9)-C(17)	8.9(8)	C(2)-
C(3)-C(9)-C(17)	147.5(5)	C(10)-C(3)-C(9)-C(17)	-107.0(6)	C(4)-
C(3)-C(9)-C(11)	-133.2(6)	C(2)-C(3)-C(9)-C(11)	5.4(7)	C(10)-
C(3)-C(9)-C(11)	111.0(6)	C(17)-C(9)-C(10)-C(3)	108.3(5)	C(11)-
C(9)-C(10)-C(3)	-105.6(5)	C(4)-C(3)-C(10)-C(9)	-110.4(6)	C(2)-
C(3)-C(10)-C(9)	108.9(5)	C(10)-C(9)-C(11)-C(16)	-37.6(7)	C(17)-
C(9)-C(11)-C(16)	109.5(6)	C(3)-C(9)-C(11)-C(16)	-106.8(6)	C(10)-
C(9)-C(11)-C(12)	144.9(5)	C(17)-C(9)-C(11)-C(12)	-67.9(6)	C(3)-
C(9)-C(11)-C(12)	75.7(6)	C(16)-C(11)-C(12)-C(13)	0.0(8)	C(9)-
C(11)-C(12)-C(13)	177.5(5)	C(11)-C(12)-C(13)-C(14)	-0.3(8)	C(12)-
C(13)-C(14)-C(15)	0.3(9)	C(13)-C(14)-C(15)-C(16)	0.0(8)	C(12)-
C(11)-C(16)-C(15)	0.4(8)	C(9)-C(11)-C(16)-C(15)	-177.1(5)	C(14)-
C(15)-C(16)-C(11)	-0.3(8)	C(10)-C(9)-C(17)-C(18)	-136.4(5)	C(11)-
C(9)-C(17)-C(18)	75.4(6)	C(3)-C(9)-C(17)-C(18)	-67.8(7)	C(10)-
C(9)-C(17)-Si(1)	50.4(6)	C(11)-C(9)-C(17)-Si(1)	-97.7(5)	C(3)-
C(9)-C(17)-Si(1)	119.1(5)	C(19)-Si(1)-C(17)-C(18)	-13.1(5)	C(20)-
Si(1)-C(17)-C(18)	-134.0(5)	C(21)-Si(1)-C(17)-C(18)	105.5(5)	C(19)-
Si(1)-C(17)-C(9)	160.4(4)	C(20)-Si(1)-C(17)-C(9)	39.6(5)	C(21)-
Si(1)-C(17)-C(9)	-81.0(5)			

Symmetry transformations used to generate equivalent atoms:

5. References:

(1) (a) Muthusamy, S.; Kumar, S. G., *Org. Biomol. Chem.* **2016,** *14*, 2228. (b) Murphy, G. K.; Abbas, F. Z.; Poulton, A. V., *Adv. Synth. Catal.* **2014,** *356*, 2919. (c) Cao, Z.-Y.; Wang, X.; Tan, C.; Zhao, X.-L.; Zhou, J.; Ding, K., *J. Am. Chem. Soc.* **2013,** *135*, 8197. (d) Muthusamy, S.; Azhagan, D., *Tetrahedron Lett.* **2011,** *52*, 6732.

(2) (a) Hansen, J.; Davies, H. M. L., *Coord. Chem. Rev.* **2008,** *252*, 545. (b) Javed, M. I.; Brewer, M., *Org. Lett.* **2007,** *9*, 1789. (c) Ren, A.; Lang, B.; Lin, J.; Lu, P.; Wang, Y., *J. Org. Chem.* **2017**.

(3) Loebach, J. L.; Bennett, D. M.; Danheiser, R. L., *J. Org. Chem.* **1998,** *63*, 8380.

(4) Yamawaki, M.; Tsutsui, H.; Kitagaki, S.; Anada, M.; Hashimoto, S., *Tetrahedron Lett.* **2002,** *43*, 9561.

(5) Tsutsui, H.; Yamaguchi, Y.; Kitagaki, S.; Nakamura, S.; Anada, M.; Hashimoto, S., *Tetrahedron: Asymmetry* **2003,** *14*, 817.

(6) Tsutsui, H.; Abe, T.; Nakamura, S.; Anada, M.; Hashimoto, S., *Chemical and Pharmaceutical Bulletin* **2005,** *53*, 1366.

(7) Lindsay, V. N. G.; Fiset, D.; Gritsch, P. J.; Azzi, S.; Charette, A. B., *J. Am. Chem. Soc.* **2013,** *135*, 1463.

(8) Allen, C. P.; Benkovics, T.; Turek, A. K.; Yoon, T. P., *J. Am. Chem. Soc.* **2009,** *131*, 12560.

(9) Köllhofer, A.; Plenio, H., *Chem. Eur. J.* **2003,** *9*, 1416.

(10) Kownacki, I.; Orwat, B.; Marciniec, B.; Kownacka, A., *Tetrahedron Lett.* **2014,** *55*, 548.

(11) Tanaka, K.; Hoshino, Y.; Honda, K., *Tetrahedron Lett.* **2016,** *57*, 2448.

(12) Moegling, J.; Benischke, A. D.; Hammann, J. M.; Vepřek, N. A.; Zoller, F.; Rendenbach, B.; Hoffmann, A.; Sievers, H.; Schuster, M.; Knochel, P.; Herres-Pawlis, S., *Eur. J. Org. Chem.* **2015,** *2015*, 7475.

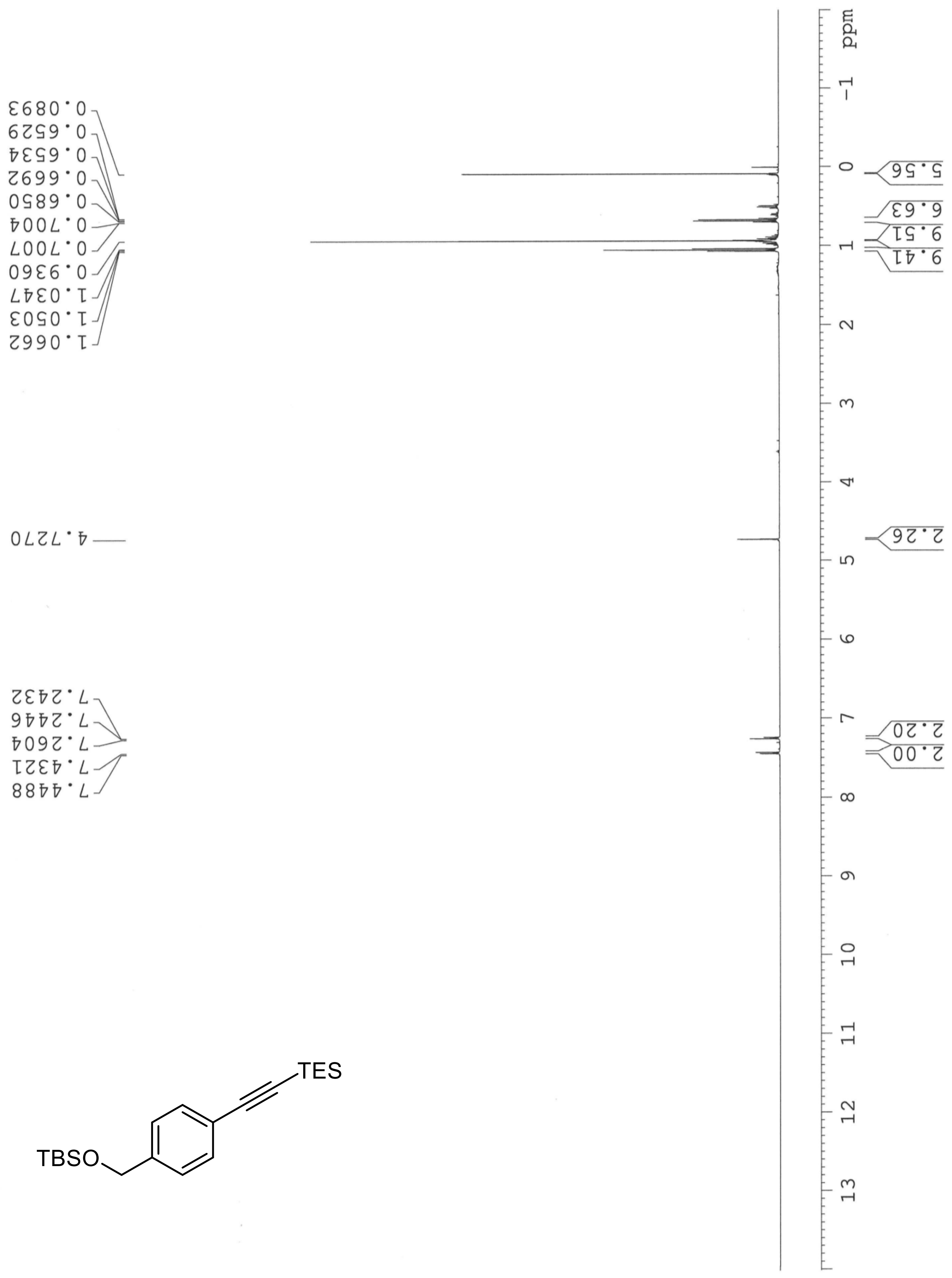
0.0893
0.6529
0.6534
0.6692
0.6850
0.7004
0.7007
0.9360
1.0347
1.0503
1.0662
4.7270
7.2432
7.2446
7.2604
7.4321
7.4488
5.56
6.63
9.51
9.41
2.26
2.20
2.00
ppm
TES
TBSO

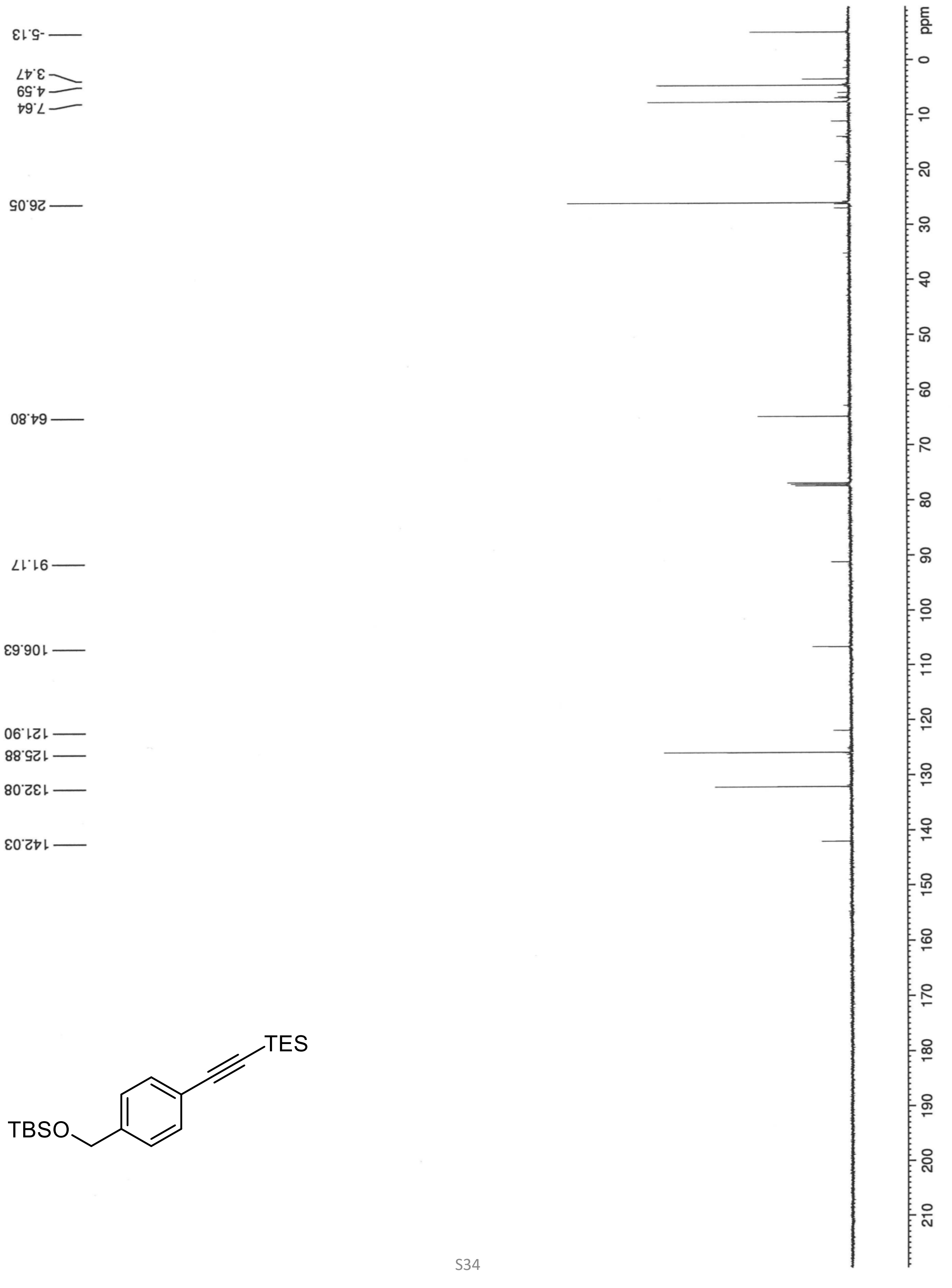
-5.13
3.47
4.59
7.64
26.05
64.80
91.17
106.63
121.90
125.88
132.08
142.03
ppm
TES
TBSO

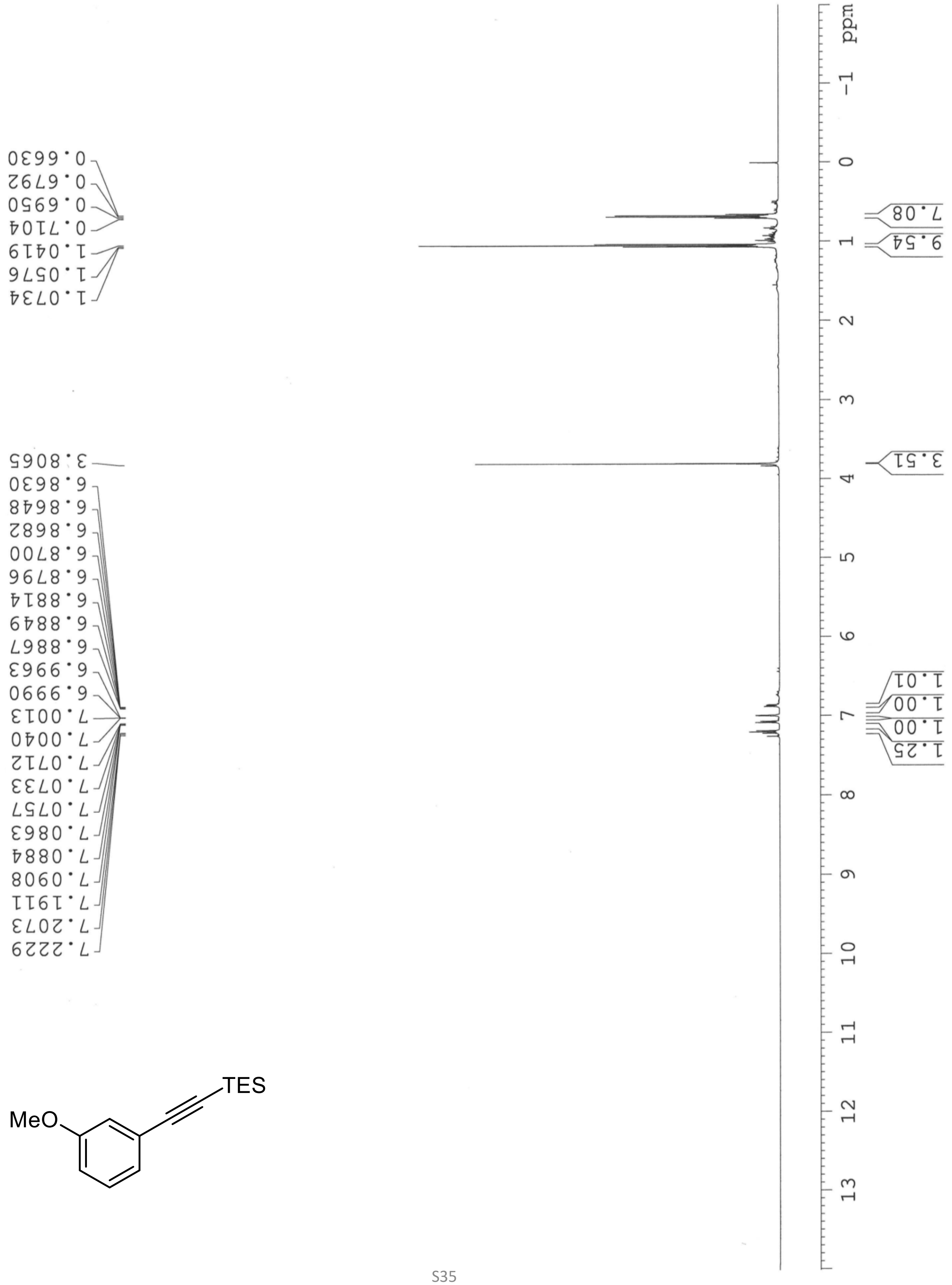
0.6630
0.6792
0.6950
0.7104
1.0419
1.0576
1.0734
3.8065
6.8630
6.8648
6.8682
6.8700
6.8796
6.8814
6.8849
6.8867
6.9963
6.9990
7.0013
7.0040
7.0712
7.0733
7.0757
7.0863
7.0884
7.0908
7.1911
7.2073
7.2229
7.08
9.54
3.51
1.01
1.00
1.00
1.25
ppm
MeO
TES

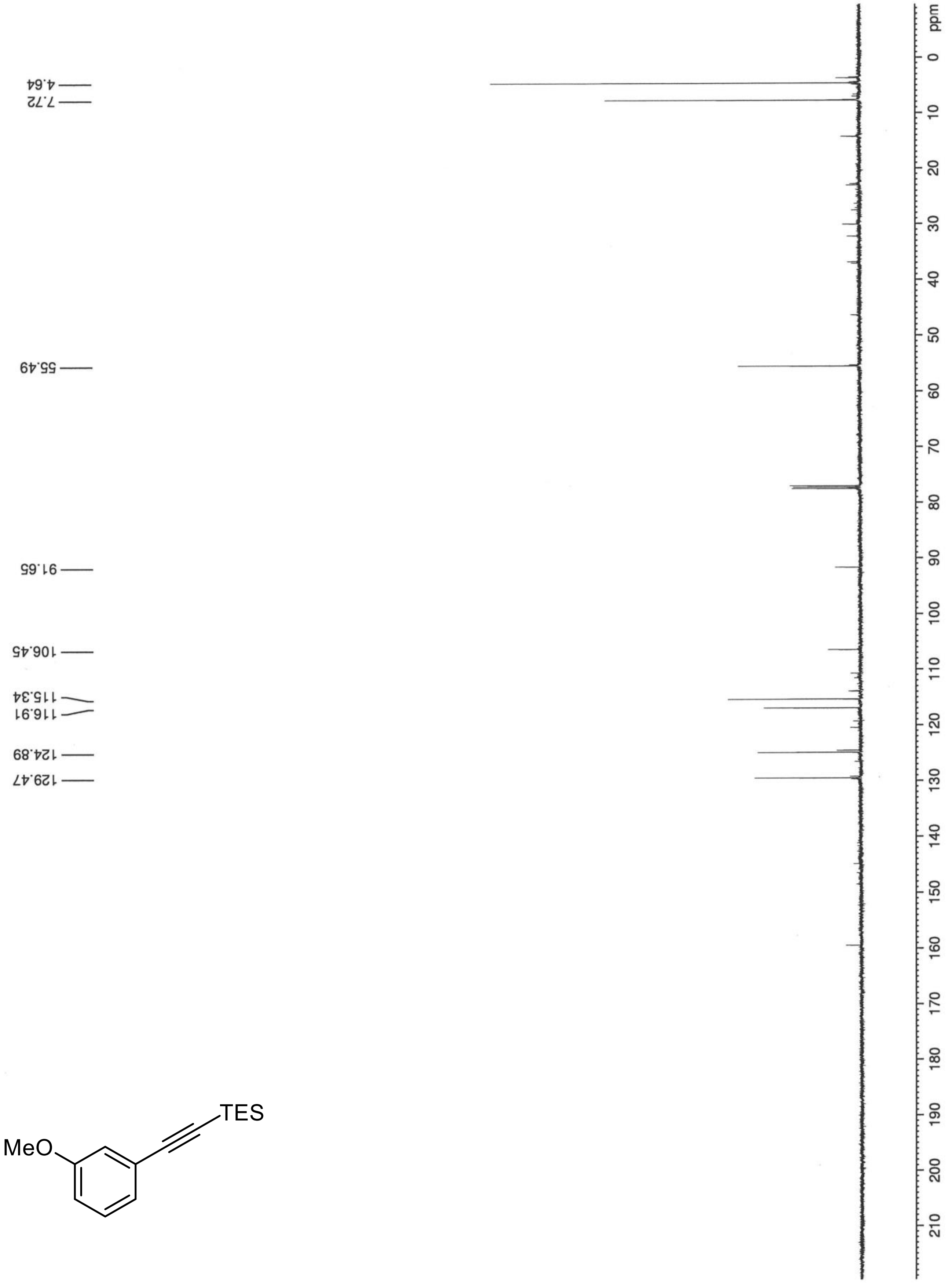
4.64
7.72
55.49
91.65
106.45
115.34
116.91
124.89
129.47
ppm
MeO
TES

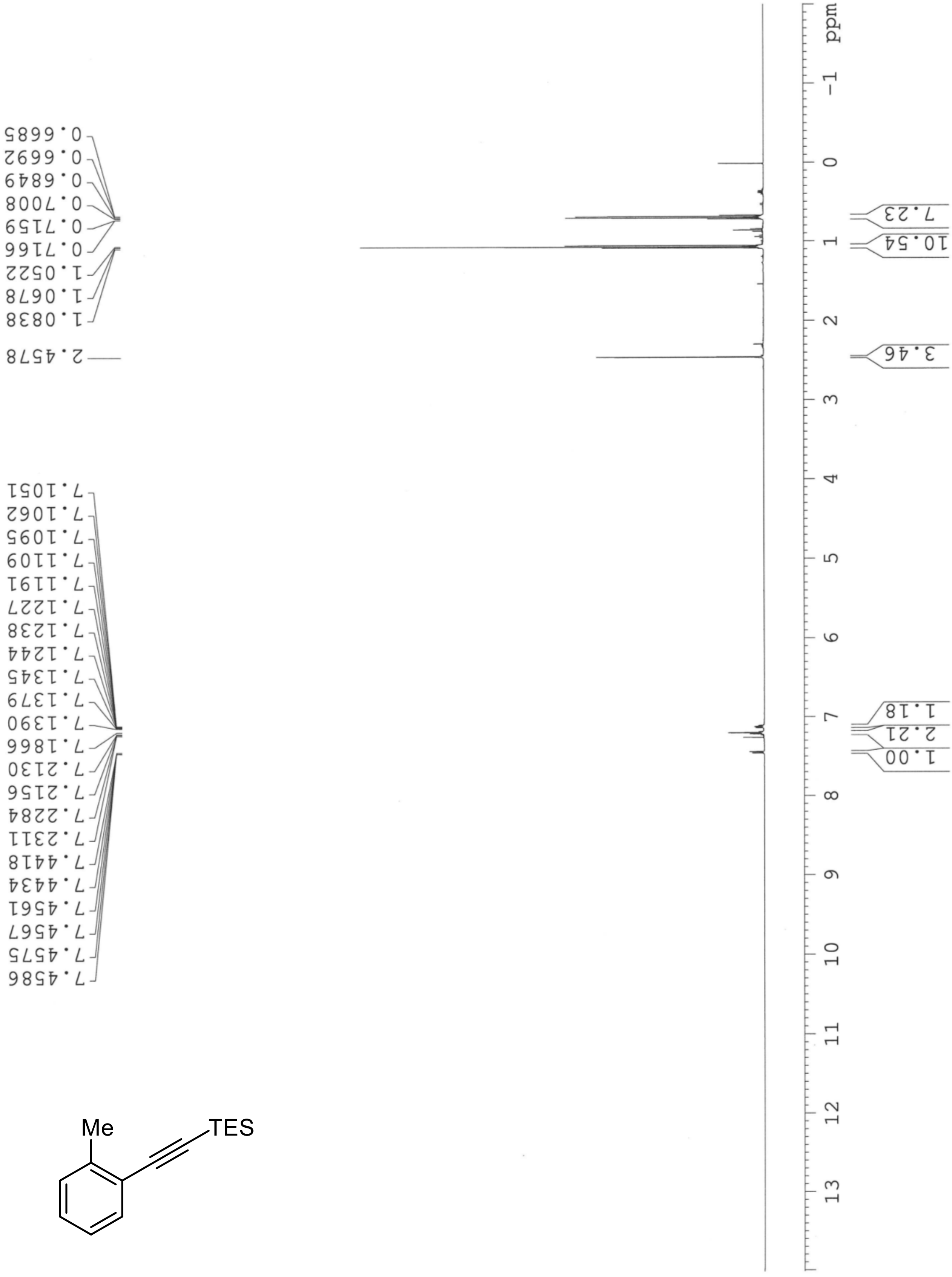
7.4586
7.4575
7.4567
7.4561
7.4434
7.4418
7.2311
7.2284
7.2156
7.2130
7.1866
7.1390
7.1379
7.1345
7.1244
7.1238
7.1227
7.1191
7.1109
7.1095
7.1062
7.1051
2.4578
1.0838
1.0678
1.0522
0.7166
0.7159
0.7008
0.6849
0.6692
0.6685
Me
TES
13
12
11
10
9
8
7
6
5
4
3
2
1
0
−1
ppm
1.00
2.21
1.18
3.46
10.54
7.23

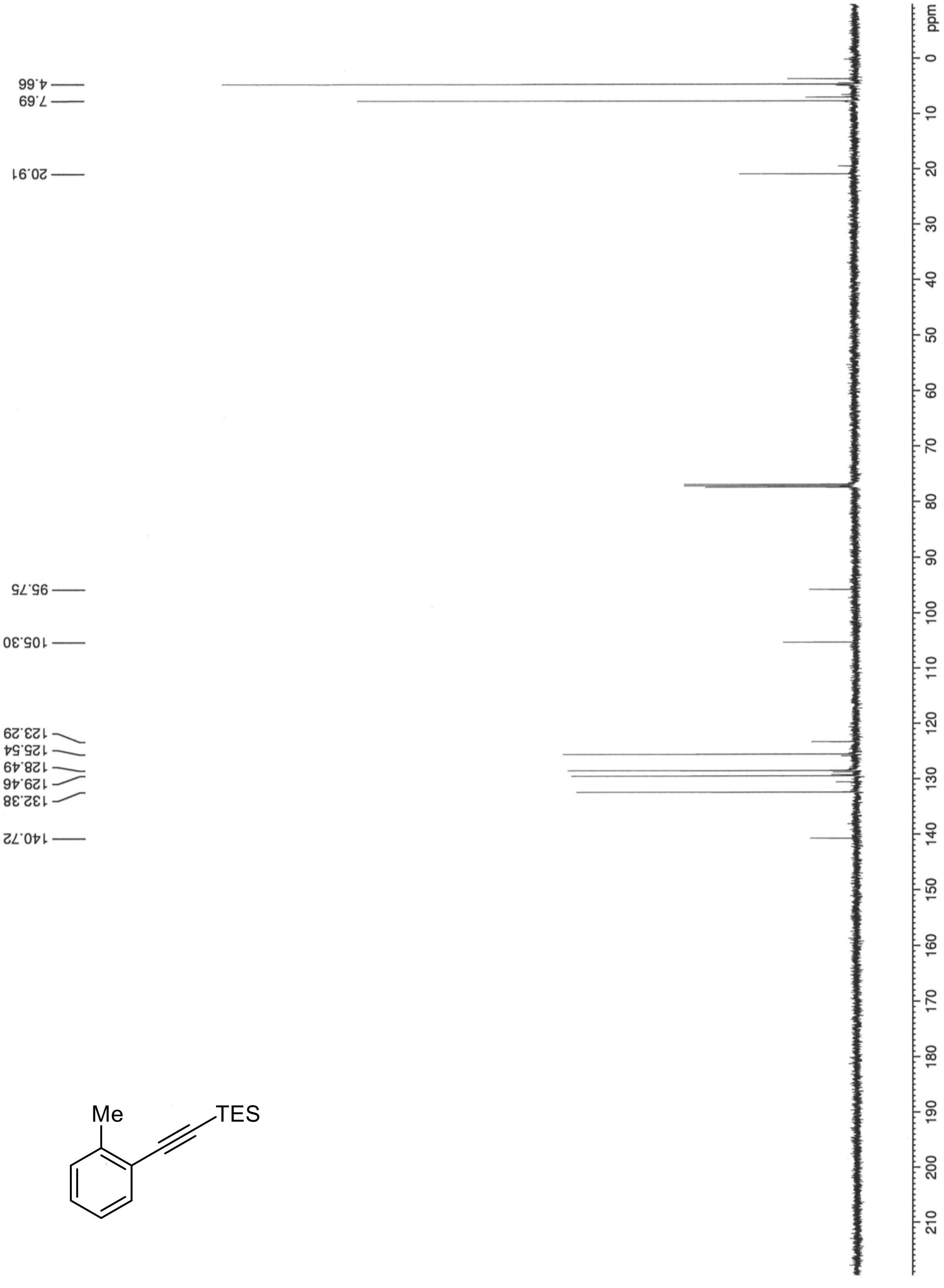
4.66
7.69
20.91
95.75
105.30
123.29
125.54
128.49
129.46
132.38
140.72
ppm
0
10
20
30
40
50
60
70
80
90
100
110
120
130
140
150
160
170
180
190
200
210
Me
TES

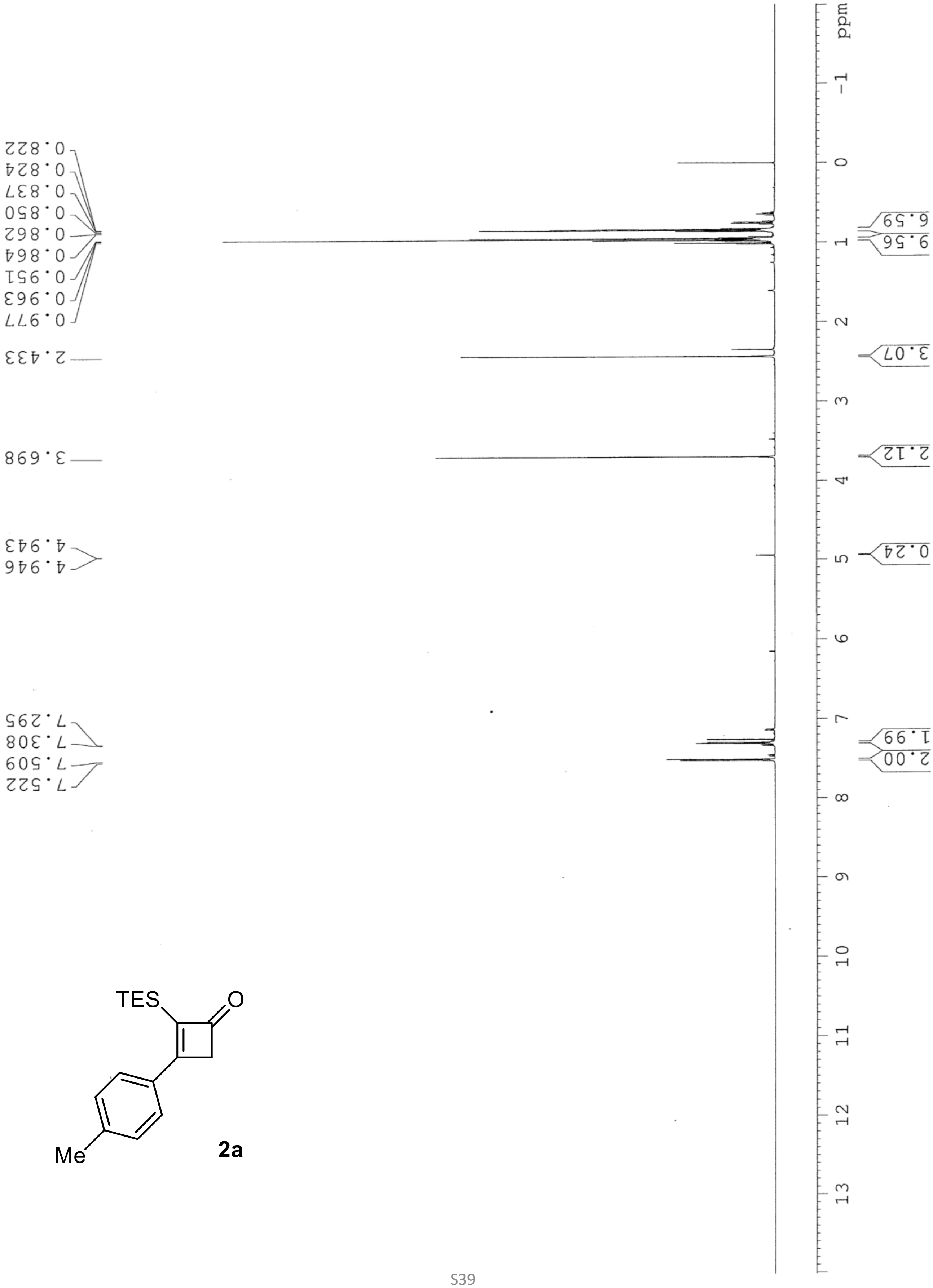
0.822
0.824
0.837
0.850
0.862
0.864
0.951
0.963
0.977
2.433
3.698
4.943
4.946
7.295
7.308
7.509
7.522
6.59
9.56
3.07
2.12
0.24
1.99
2.00
ppm
TES
O
Me

2a

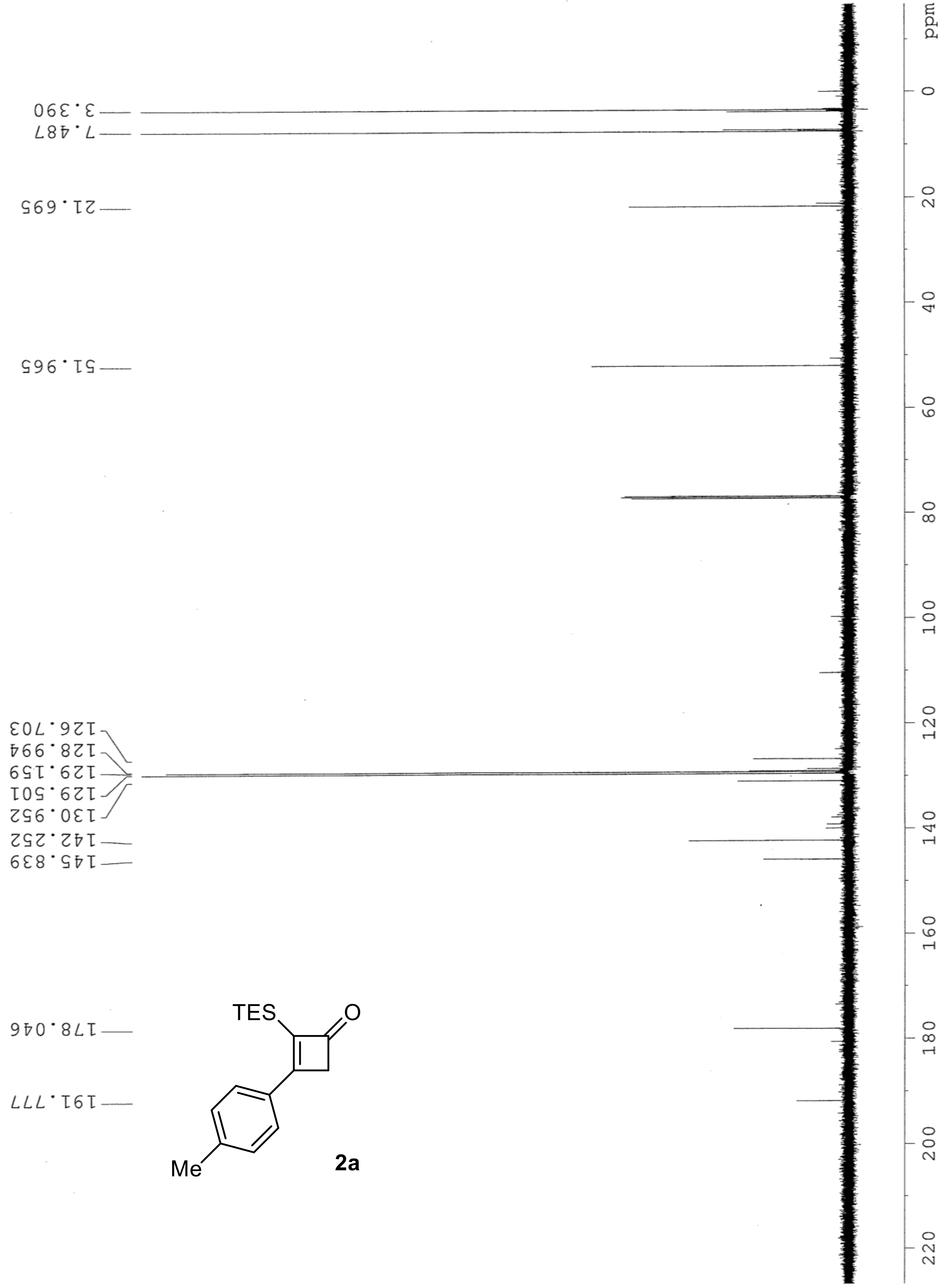
3.390
7.487
21.695
51.965
126.703
128.994
129.159
129.501
130.952
142.252
145.839
178.046
191.777
TES
O
Me
2a
ppm
0
20
40
60
80
100
120
140
160
180
200
220

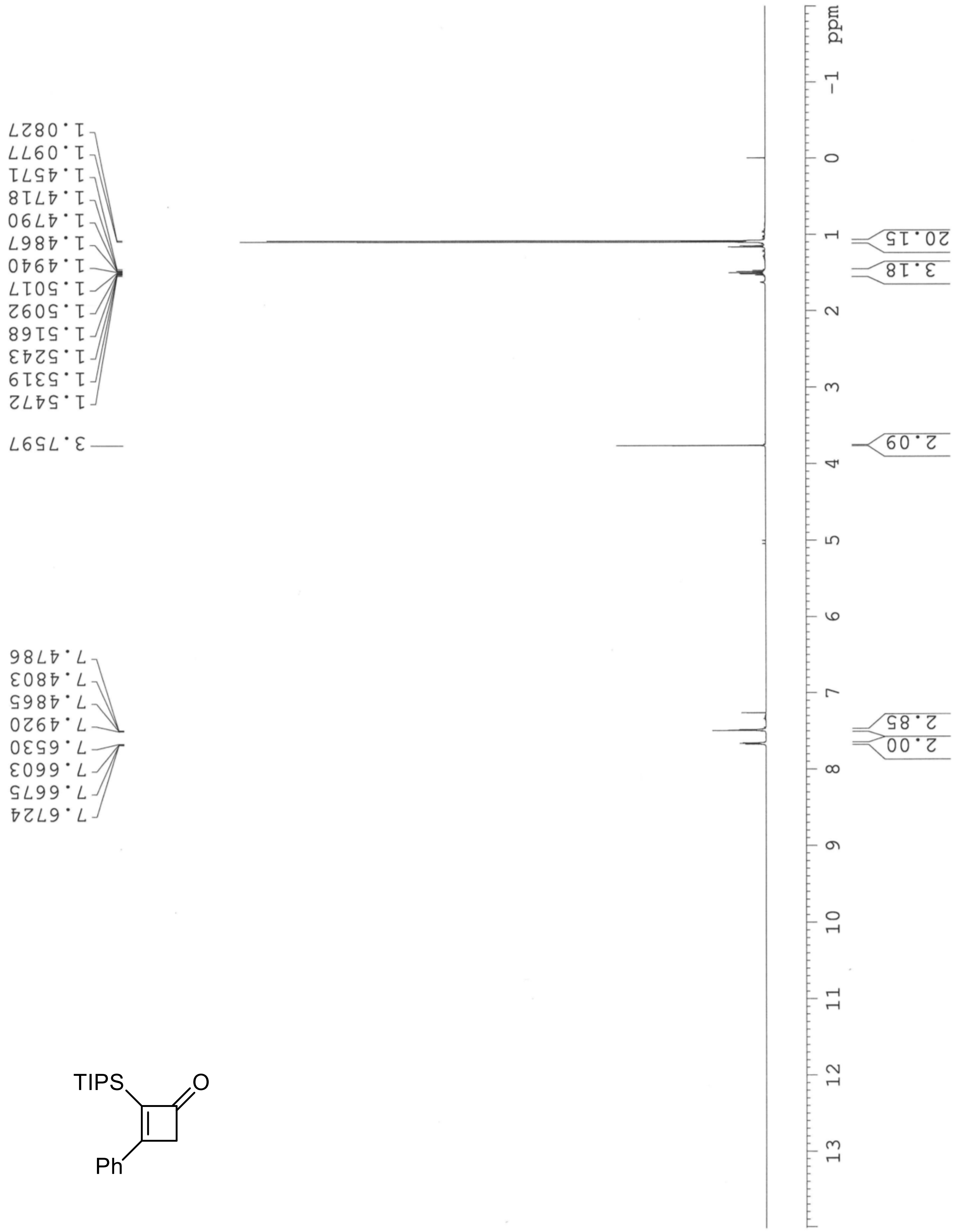
1.0827
1.0977
1.4571
1.4718
1.4790
1.4867
1.4940
1.5017
1.5092
1.5168
1.5243
1.5319
1.5472
3.7597
7.4786
7.4803
7.4865
7.4920
7.6530
7.6603
7.6675
7.6724
20.15
3.18
2.09
2.85
2.00
ppm
TIPS
O
Ph

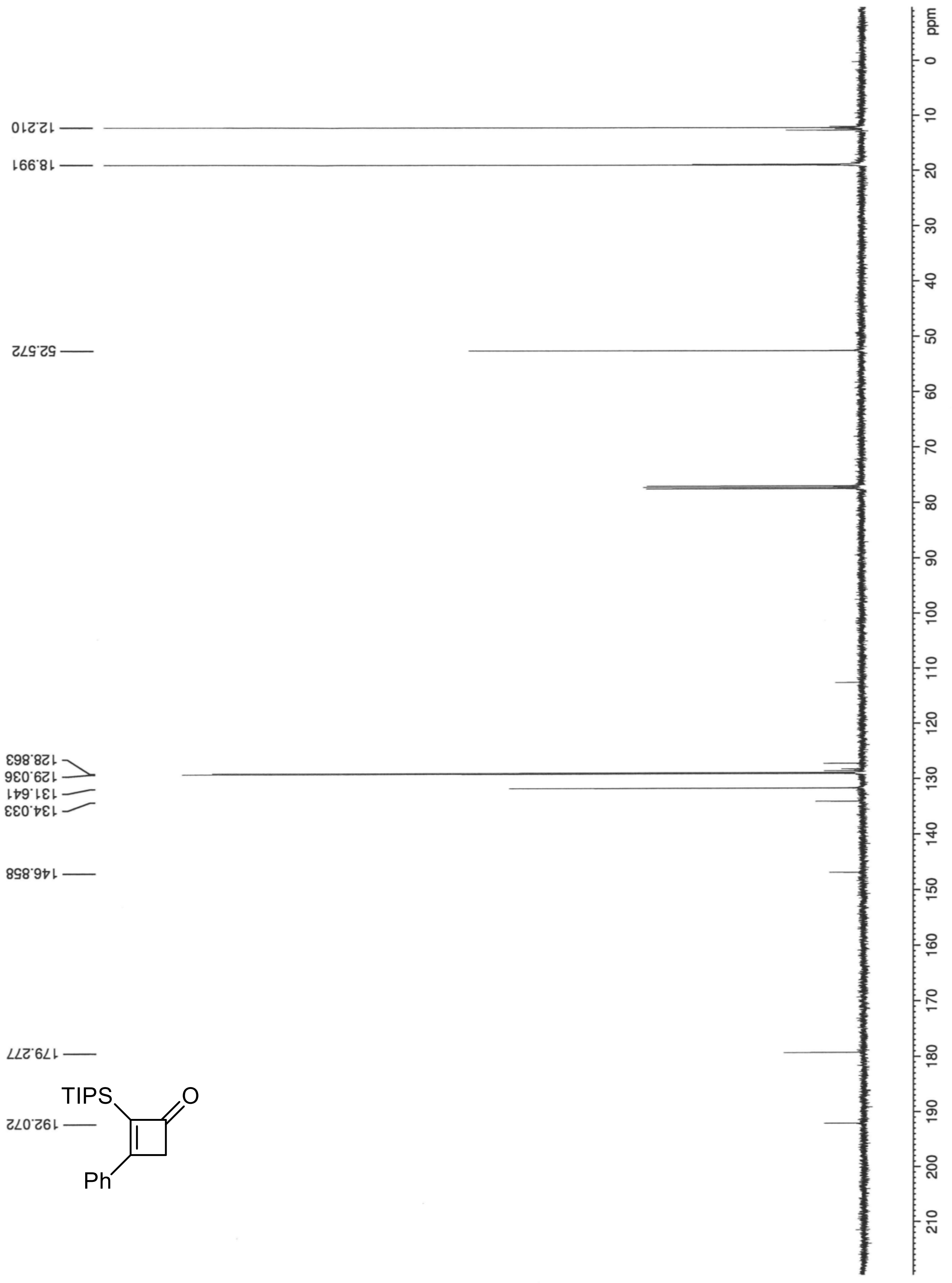
12.210
18.991
52.572
128.863
129.036
131.641
134.033
146.858
179.277
192.072
TIPS
O
Ph
ppm
0
10
20
30
40
50
60
70
80
90
100
110
120
130
140
150
160
170
180
190
200
210

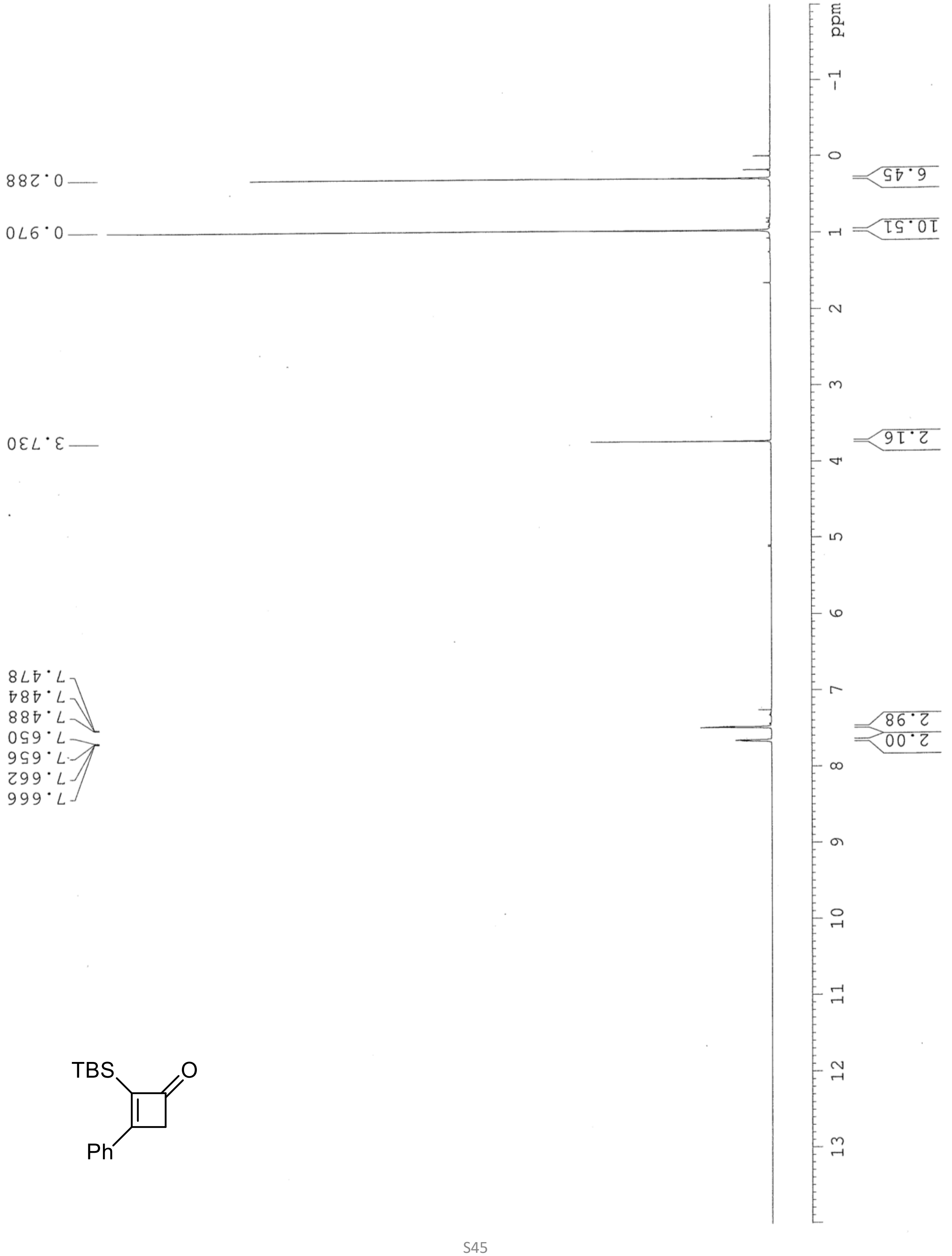
0.288
0.970
3.730
7.478
7.484
7.488
7.650
7.656
7.662
7.666
6.45
10.51
2.16
2.98
2.00
ppm
TBS
O
Ph

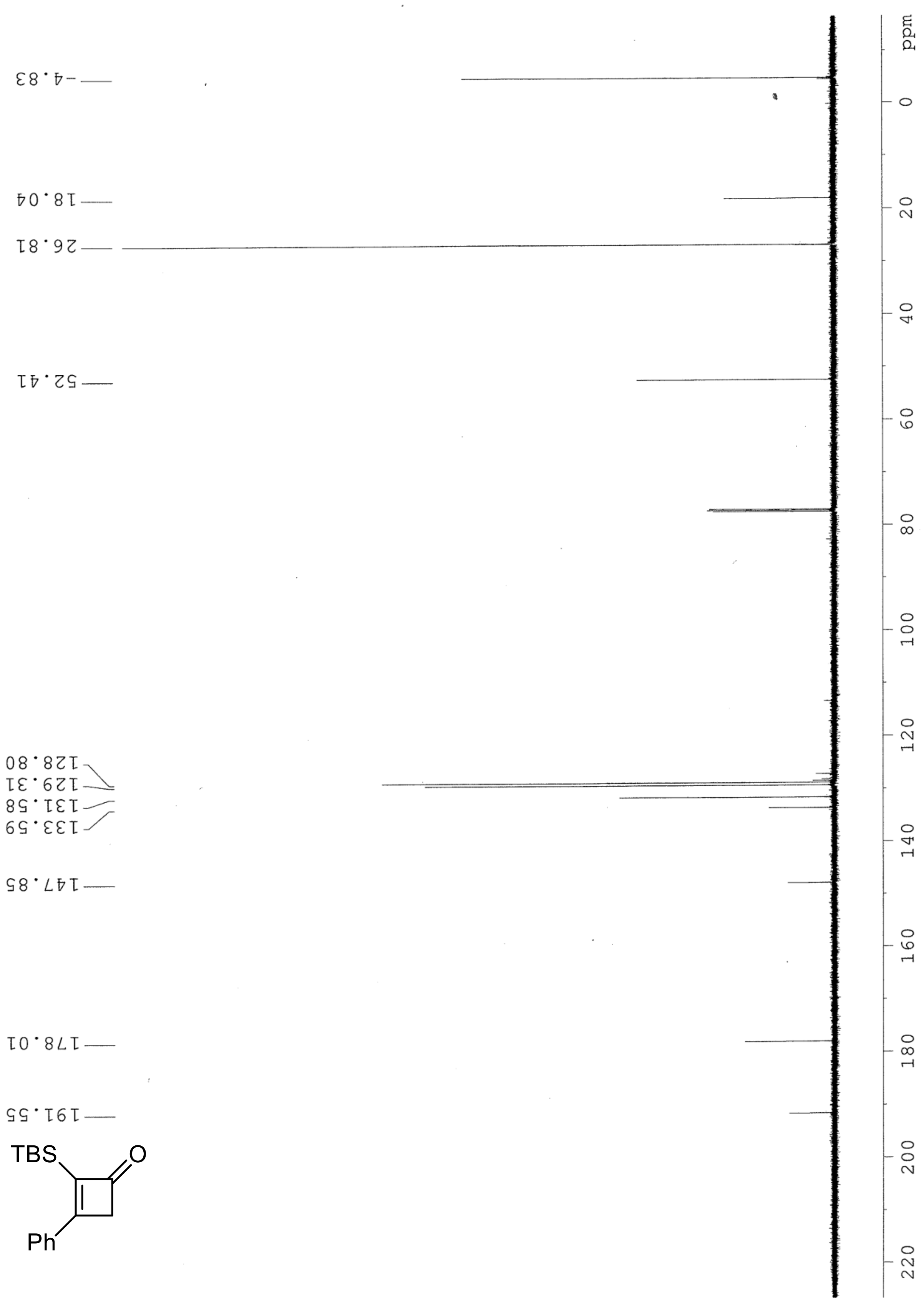
-4.83
18.04
26.81
52.41
128.80
129.31
131.58
133.59
147.85
178.01
191.55
ppm
0
20
40
60
80
100
120
140
160
180
200
220
TBS
O
Ph

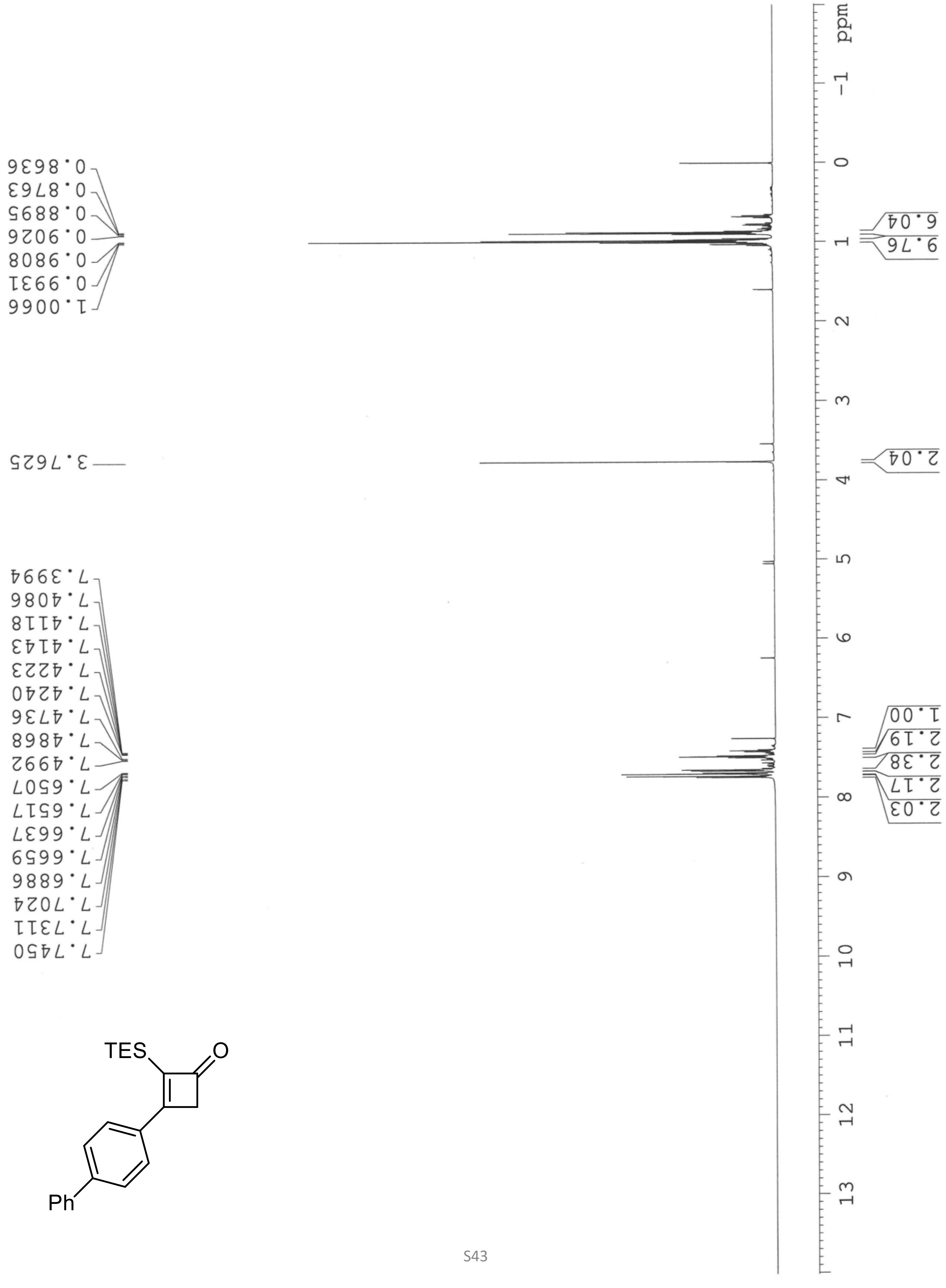
0.8636
0.8763
0.8895
0.9026
0.9808
0.9931
1.0066
3.7625
7.3994
7.4086
7.4118
7.4143
7.4223
7.4240
7.4736
7.4868
7.4992
7.6507
7.6517
7.6637
7.6659
7.6886
7.7024
7.7311
7.7450
6.04
9.76
2.04
1.00
2.19
2.38
2.17
2.03
ppm
-1
0
1
2
3
4
5
6
7
8
9
10
11
12
13
TES
O
Ph

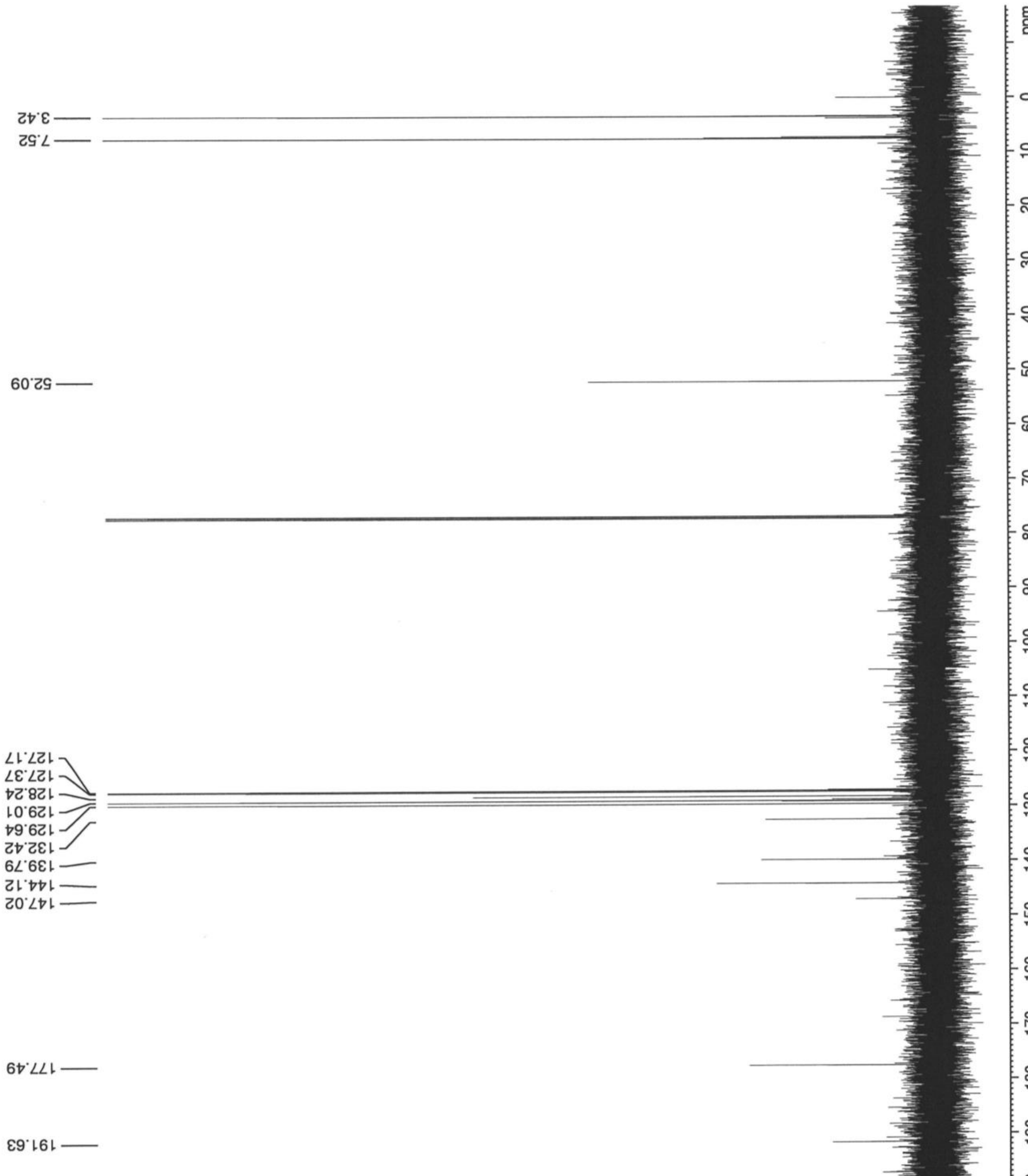
3.42
7.52
52.09
127.17
127.37
128.24
129.01
129.64
132.42
139.79
144.12
147.02
177.49
191.63
ppm

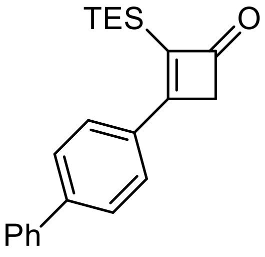
TES
O
Ph

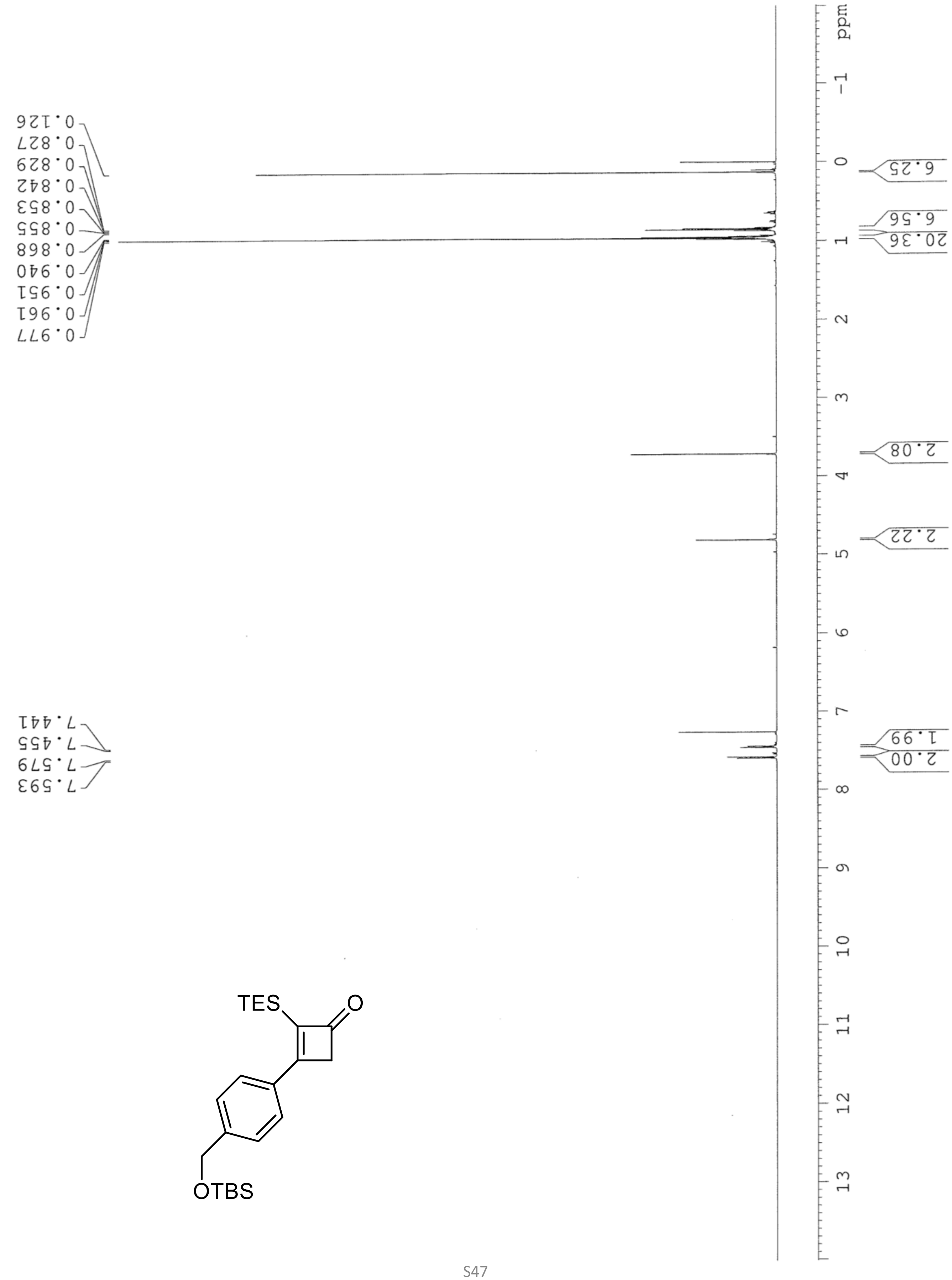
0.126
0.827
0.829
0.842
0.853
0.855
0.868
0.940
0.951
0.961
0.977
7.441
7.455
7.579
7.593
6.25
6.56
20.36
2.08
2.22
1.99
2.00
ppm
TES
O
OTBS

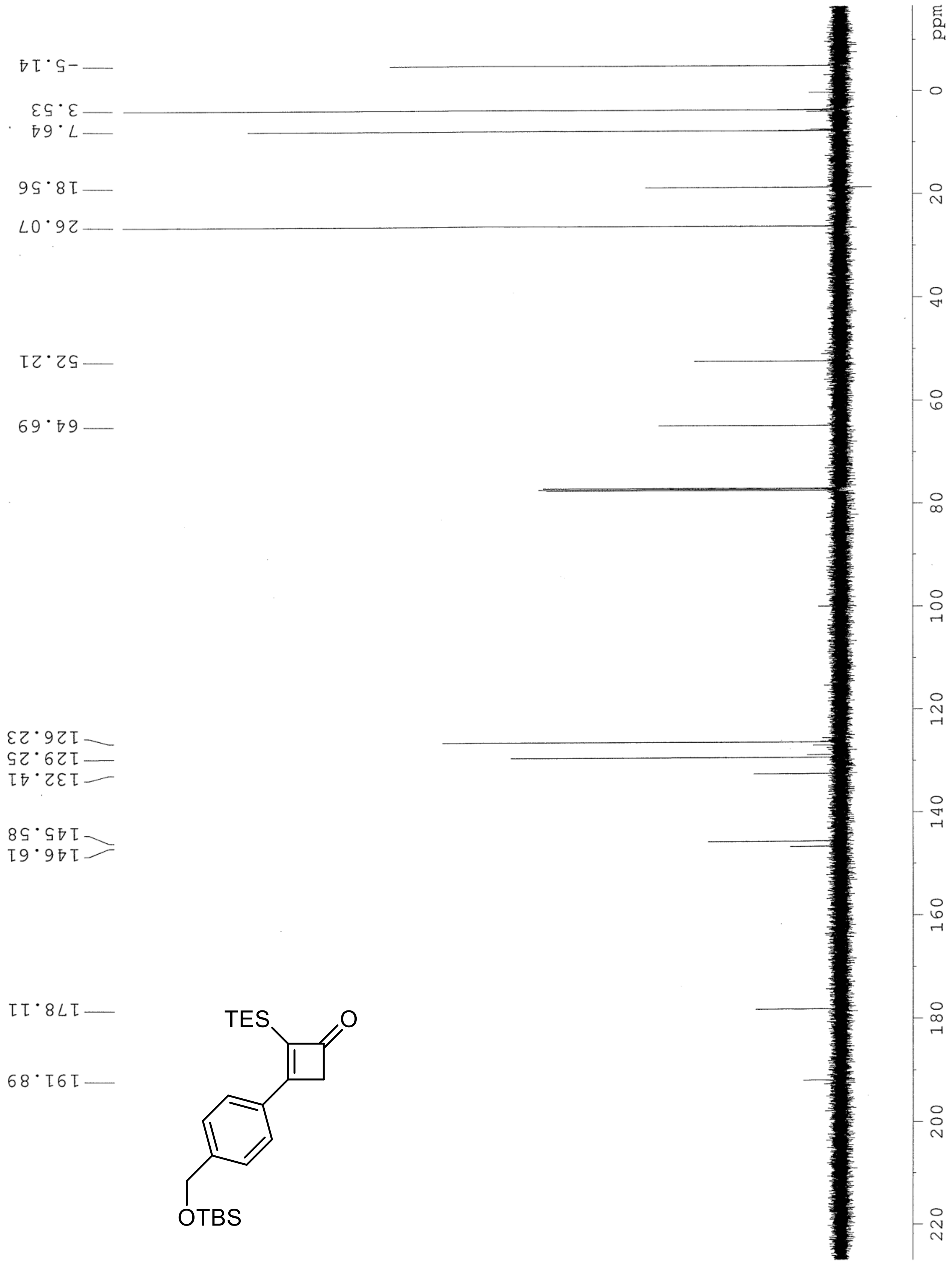
-5.14
3.53
7.64
18.56
26.07
52.21
64.69
126.23
129.25
132.41
145.58
146.61
178.11
191.89
ppm
0
20
40
60
80
100
120
140
160
180
200
220
TES
O
OTBS

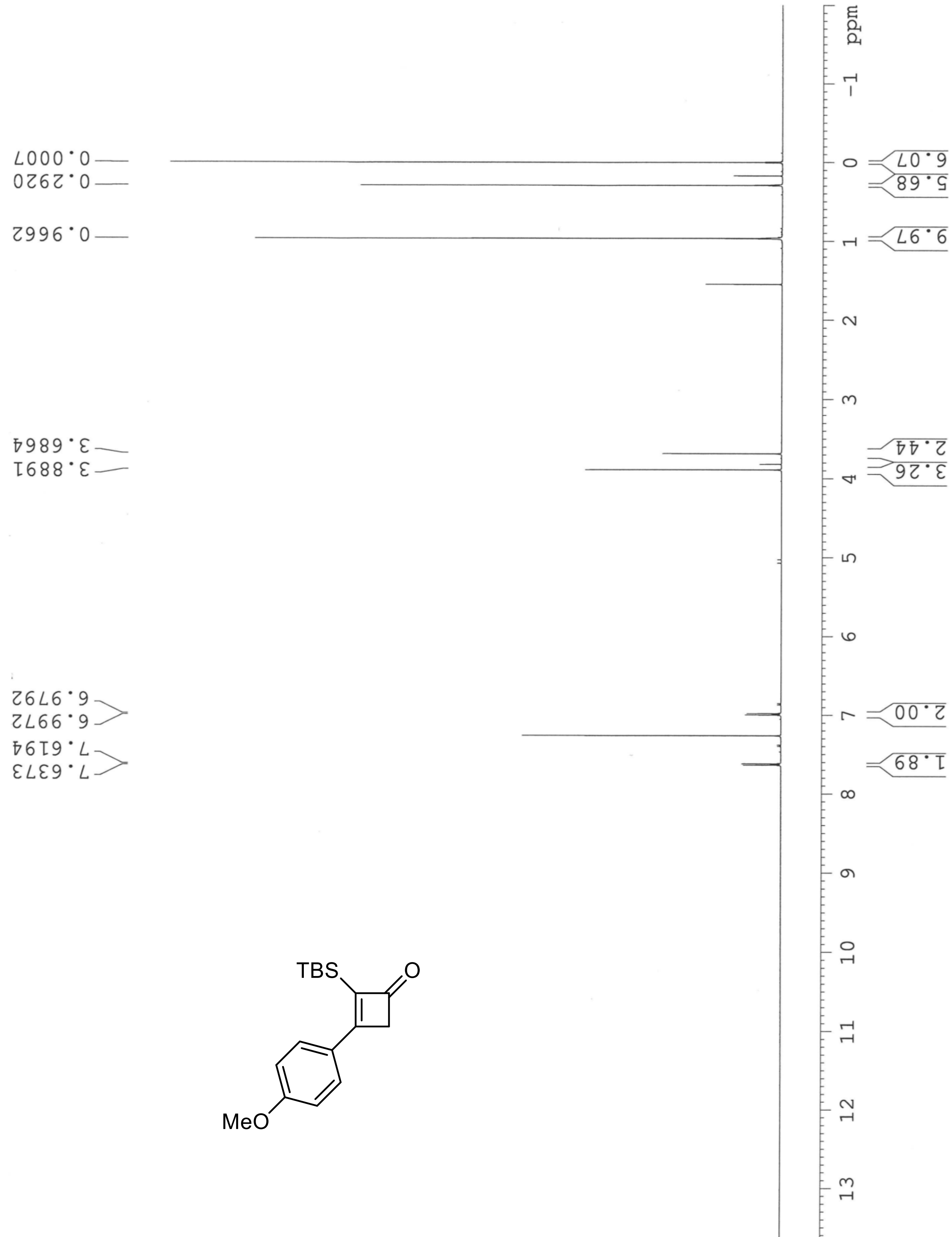
0.0007
0.2920
0.9662
3.6864
3.8891
6.9792
6.9972
7.6194
7.6373
6.07
5.68
9.97
2.44
3.26
2.00
1.89
ppm
TBS
O
MeO

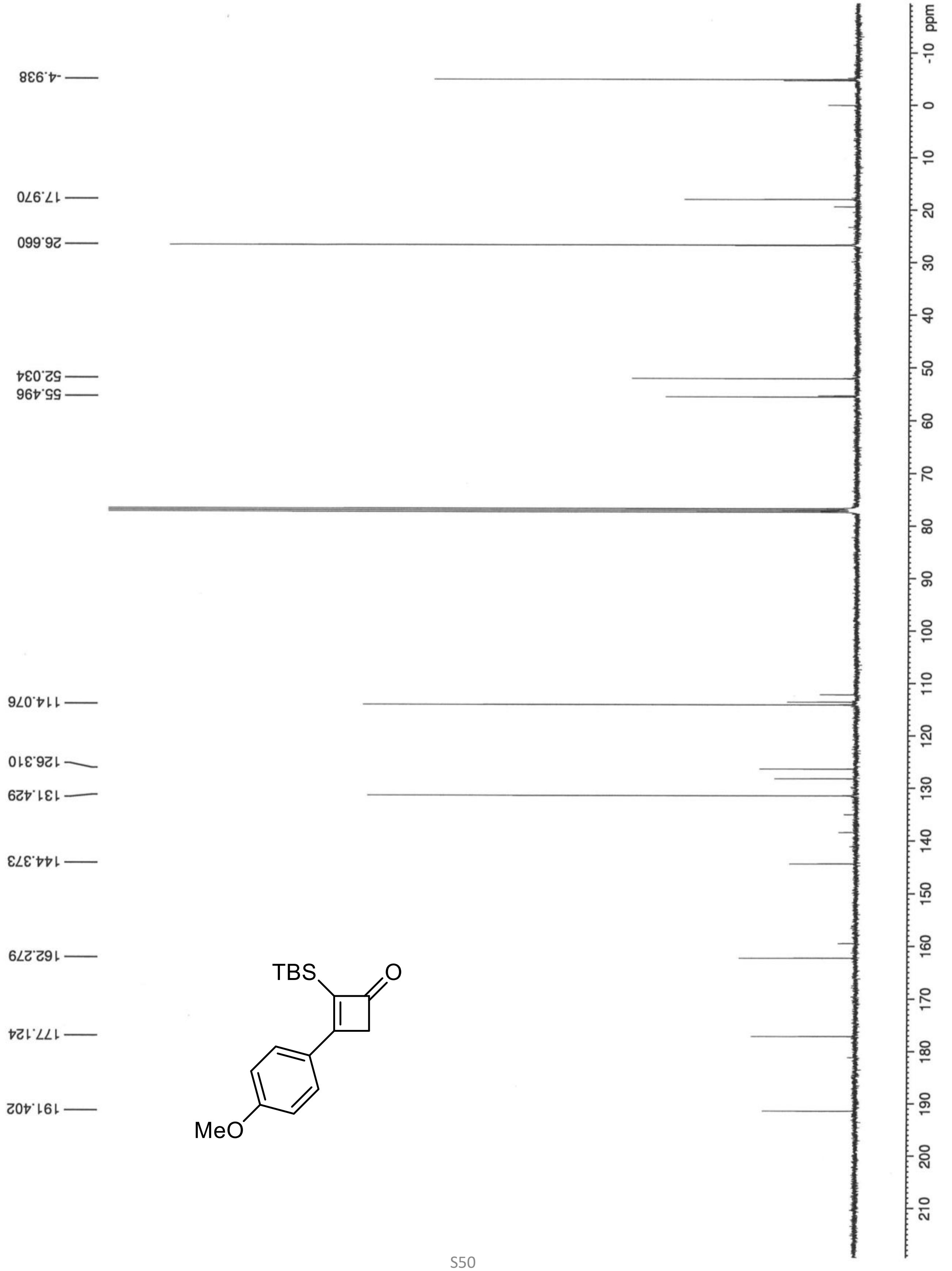
-4.938
17.970
26.660
52.034
55.496
114.076
126.310
131.429
144.373
162.279
177.124
191.402
TBS
O
MeO
ppm

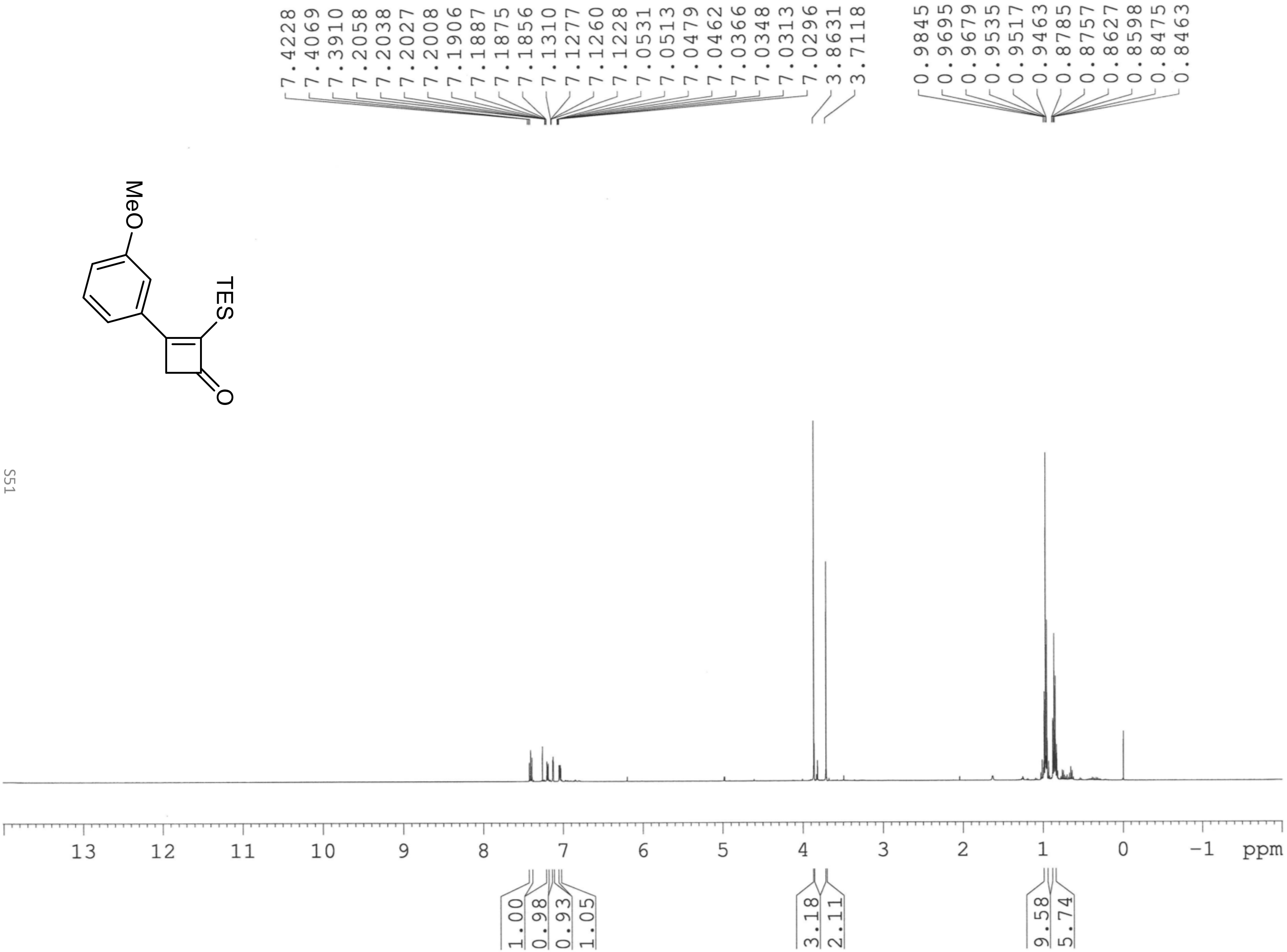
7.4228
7.4069
7.3910
7.2058
7.2038
7.2027
7.2008
7.1906
7.1887
7.1875
7.1856
7.1310
7.1277
7.1260
7.1228
7.0531
7.0513
7.0479
7.0462
7.0366
7.0348
7.0313
7.0296
3.8631
3.7118
0.9845
0.9695
0.9679
0.9535
0.9517
0.9463
0.8785
0.8757
0.8627
0.8598
0.8475
0.8463
MeO
TES
O
13 12 11 10 9 8 7 6 5 4 3 2 1 0 −1 ppm
1.00
0.98
0.93
1.05
3.18
2.11
9.58
5.74

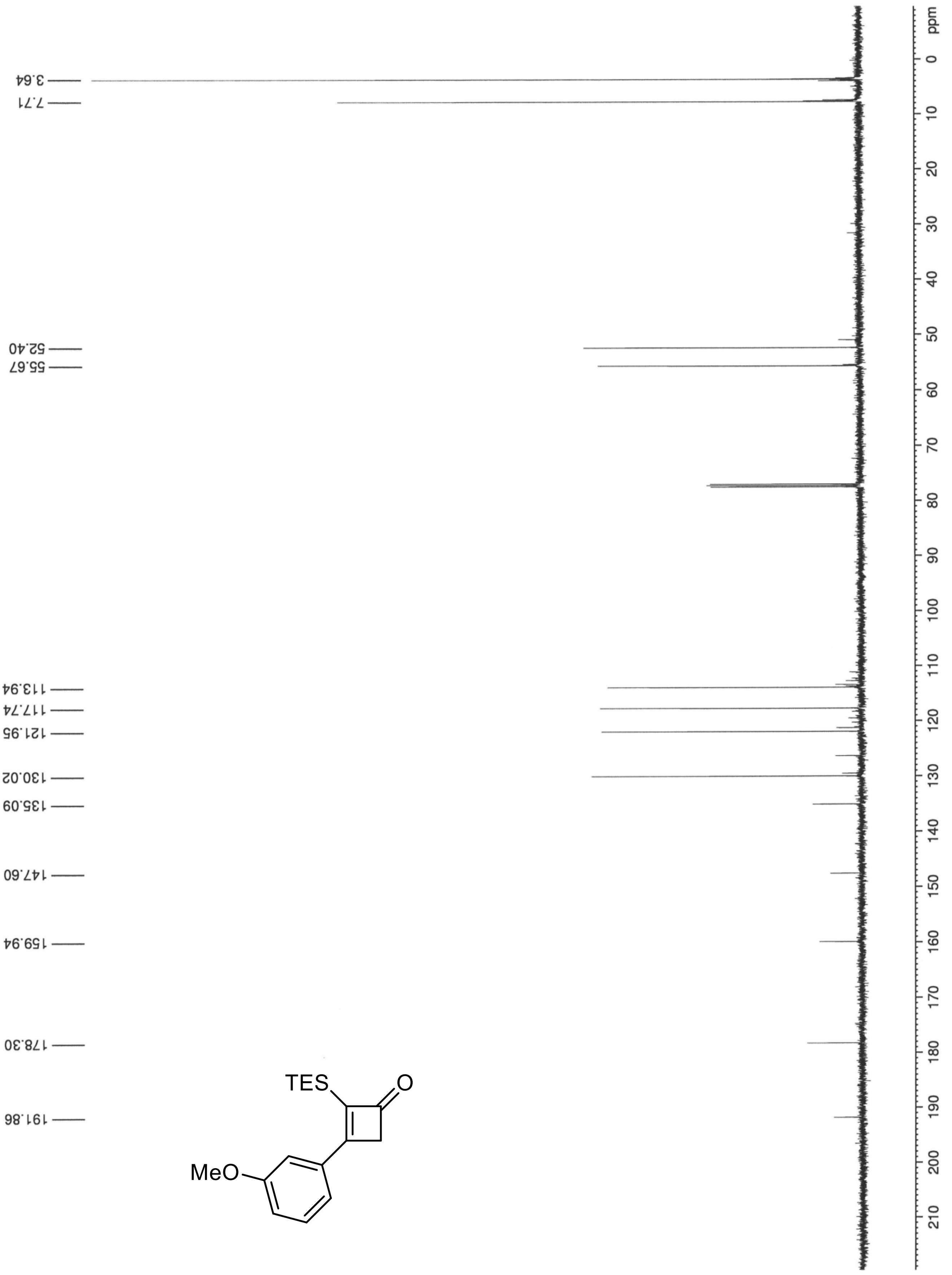
3.64
7.71
52.40
55.67
113.94
117.74
121.95
130.02
135.09
147.60
159.94
178.30
191.86
TES
O
MeO
ppm

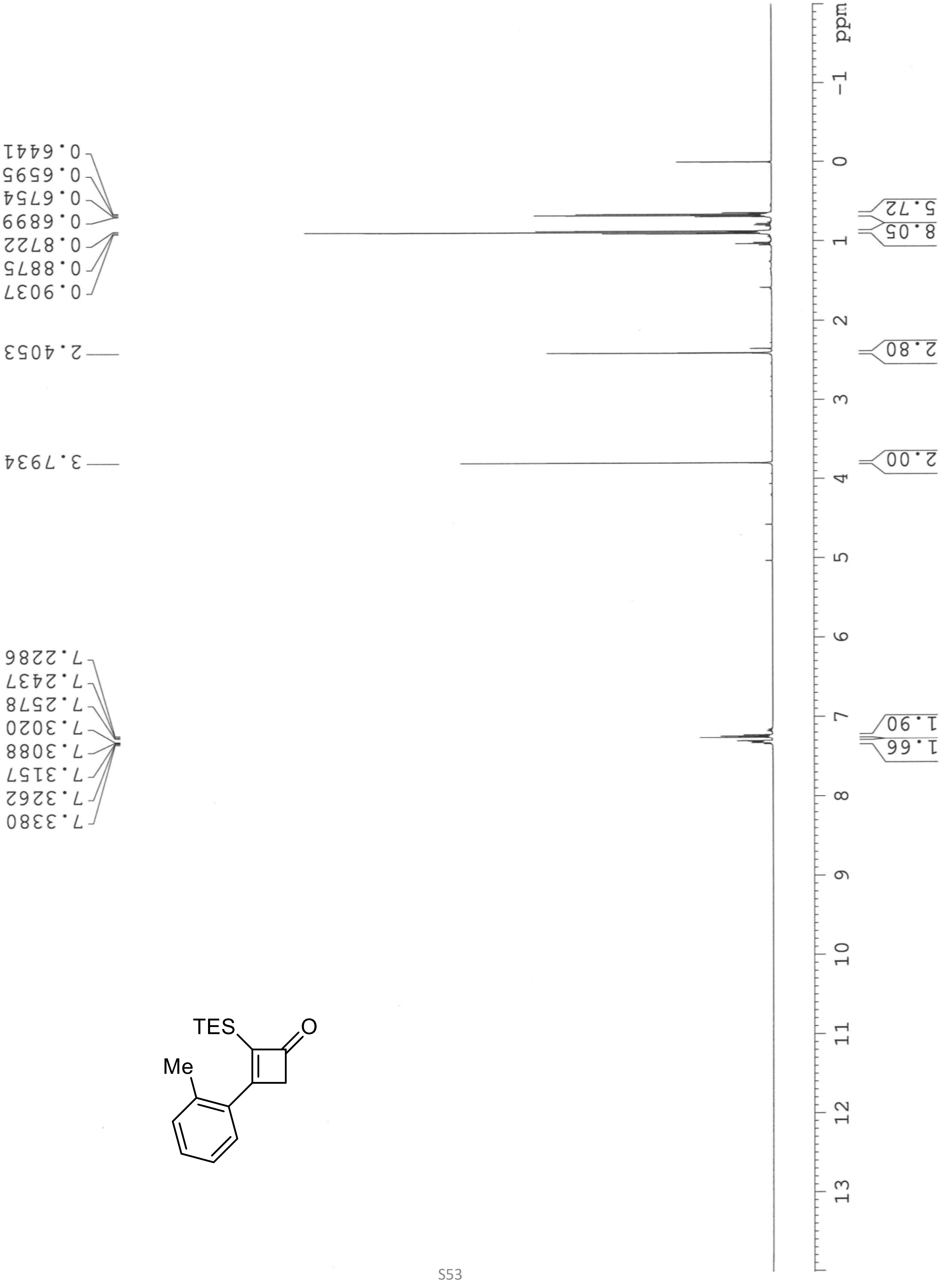
0.6441
0.6595
0.6754
0.6899
0.8722
0.8875
0.9037
2.4053
3.7934
7.2286
7.2437
7.2578
7.3020
7.3088
7.3157
7.3262
7.3380
5.72
8.05
2.80
2.00
1.90
1.66
ppm
TES
O
Me

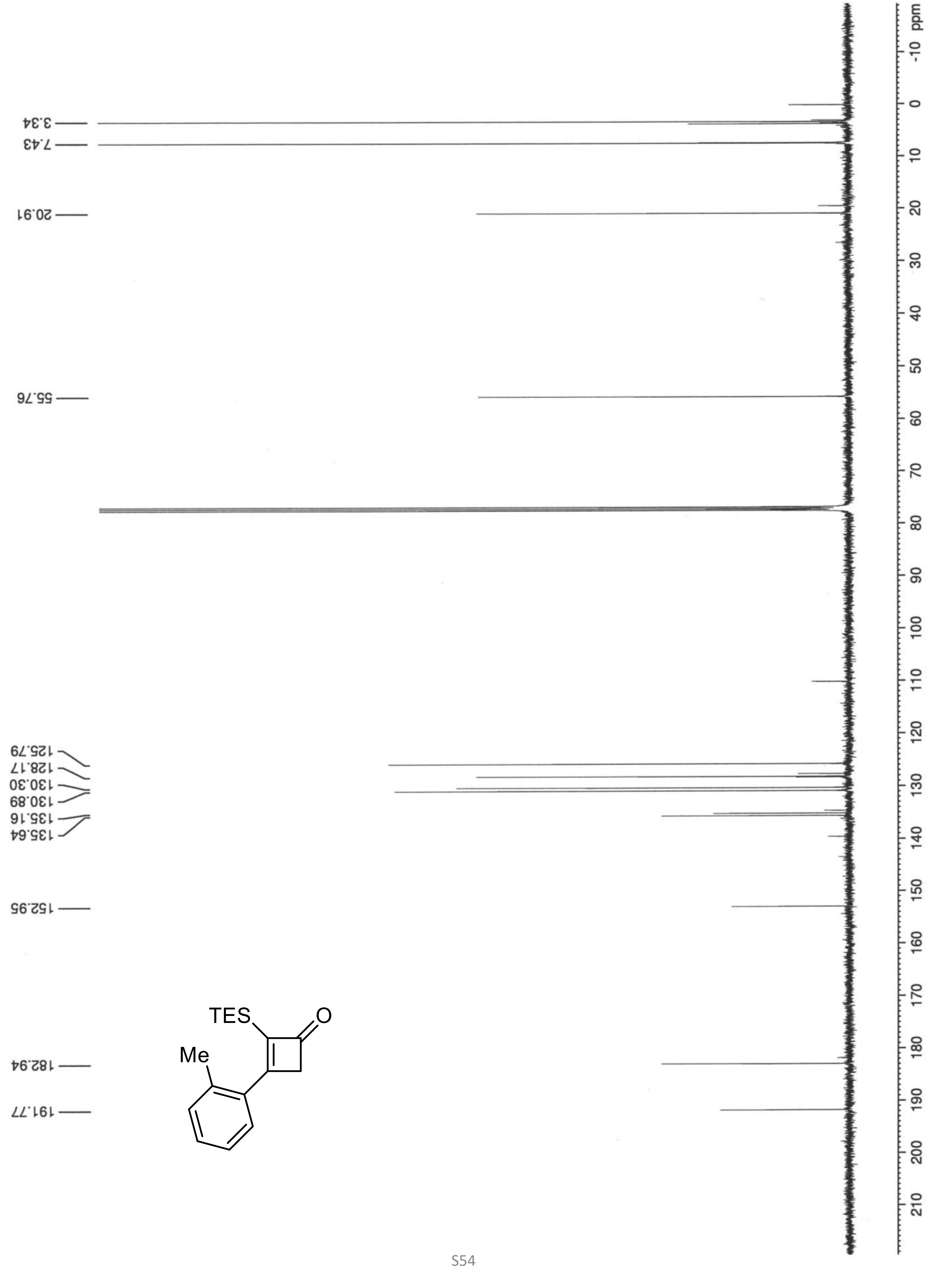
3.34
7.43
20.91
55.76
125.79
128.17
130.30
130.89
135.16
135.64
152.95
182.94
191.77
TES
O
Me
ppm

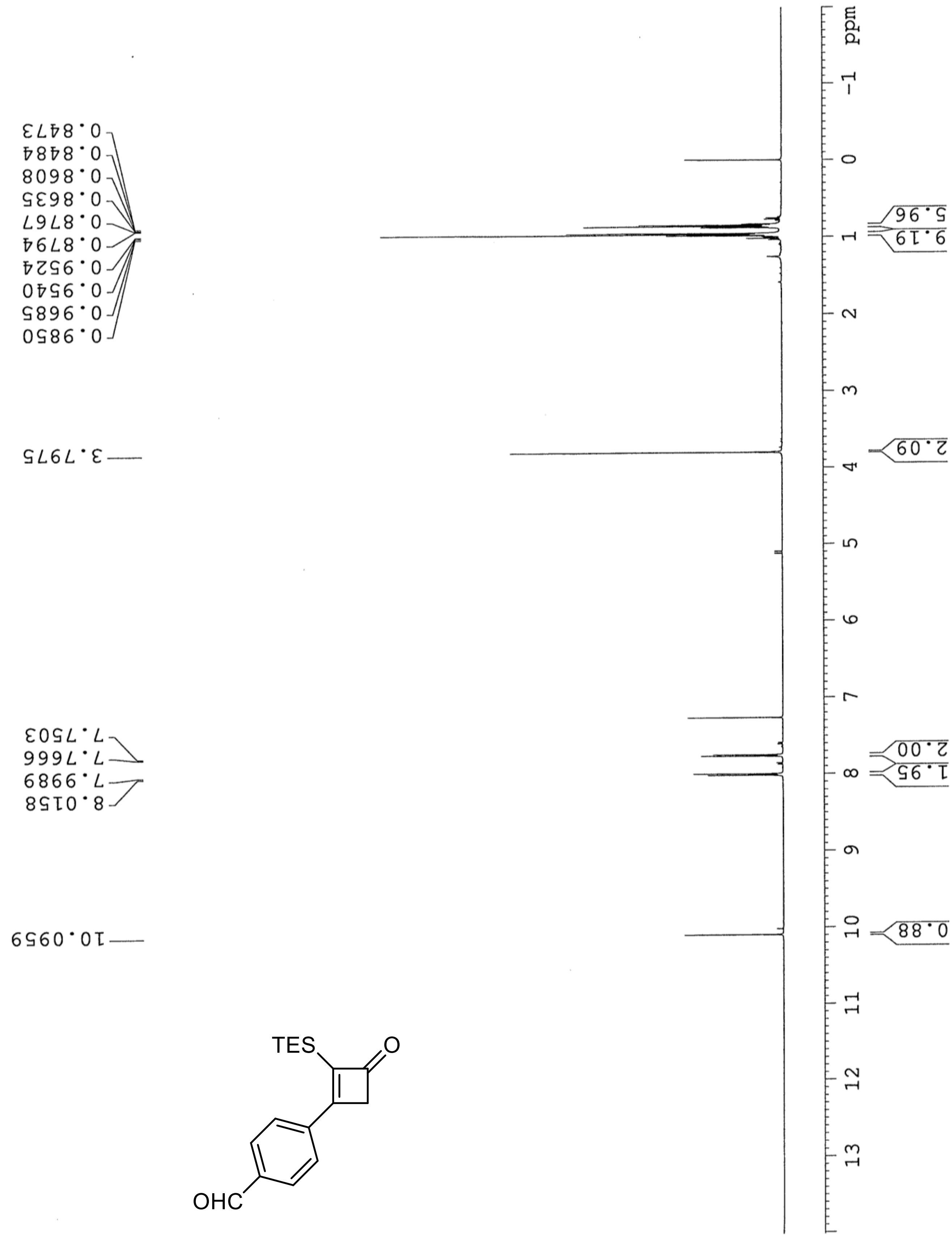
TES
O
OHC
0.8473
0.8484
0.8608
0.8635
0.8767
0.8794
0.9524
0.9540
0.9685
0.9850
3.7975
7.7503
7.7666
7.9989
8.0158
10.0959
5.96
9.19
2.09
2.00
1.95
0.88
ppm

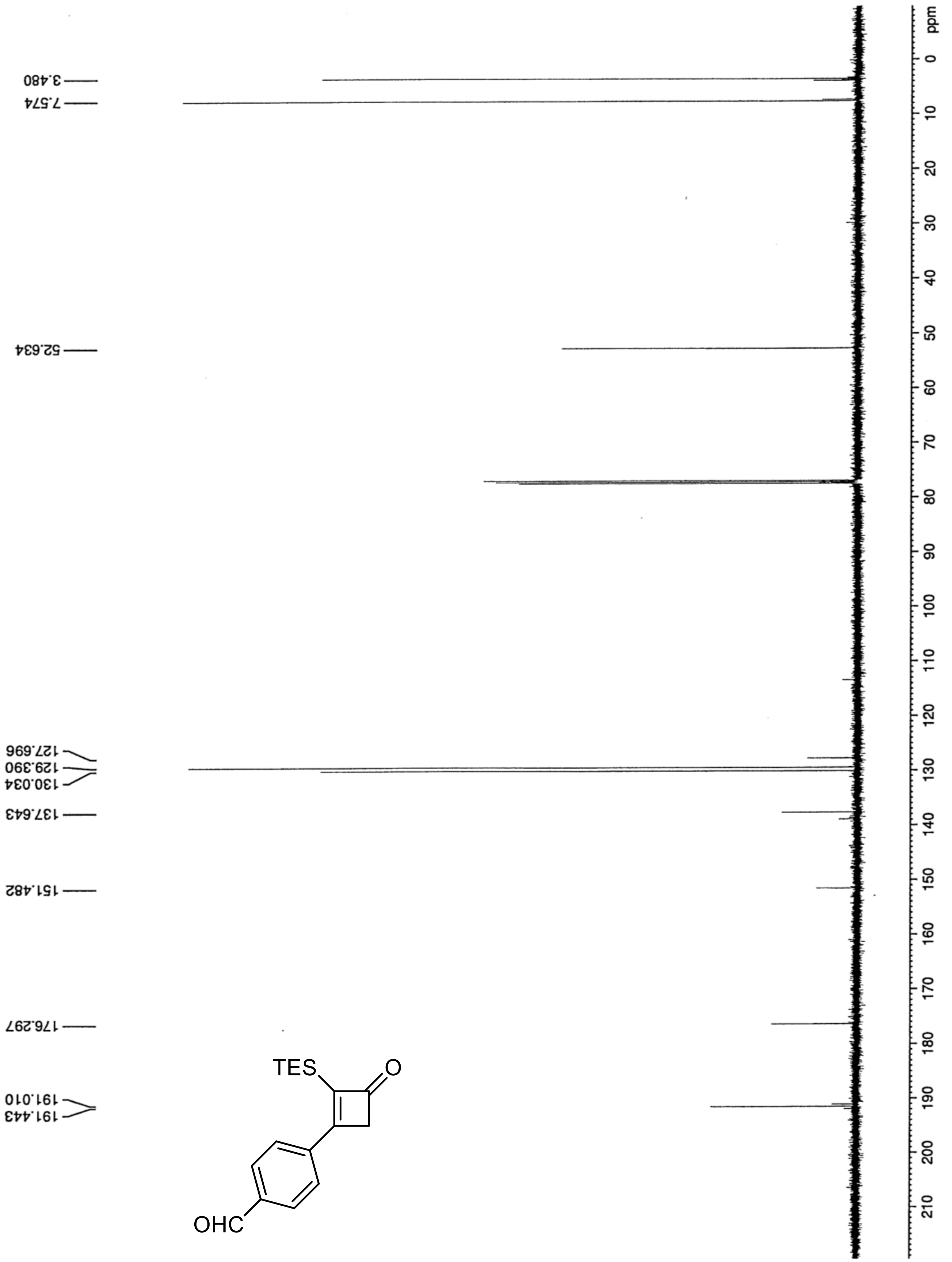

3.480
7.574
52.634
127.696
129.390
130.034
137.643
151.482
176.297
191.010
191.443
ppm
0
10
20
30
40
50
60
70
80
90
100
110
120
130
140
150
160
170
180
190
200
210
TES
O
OHC

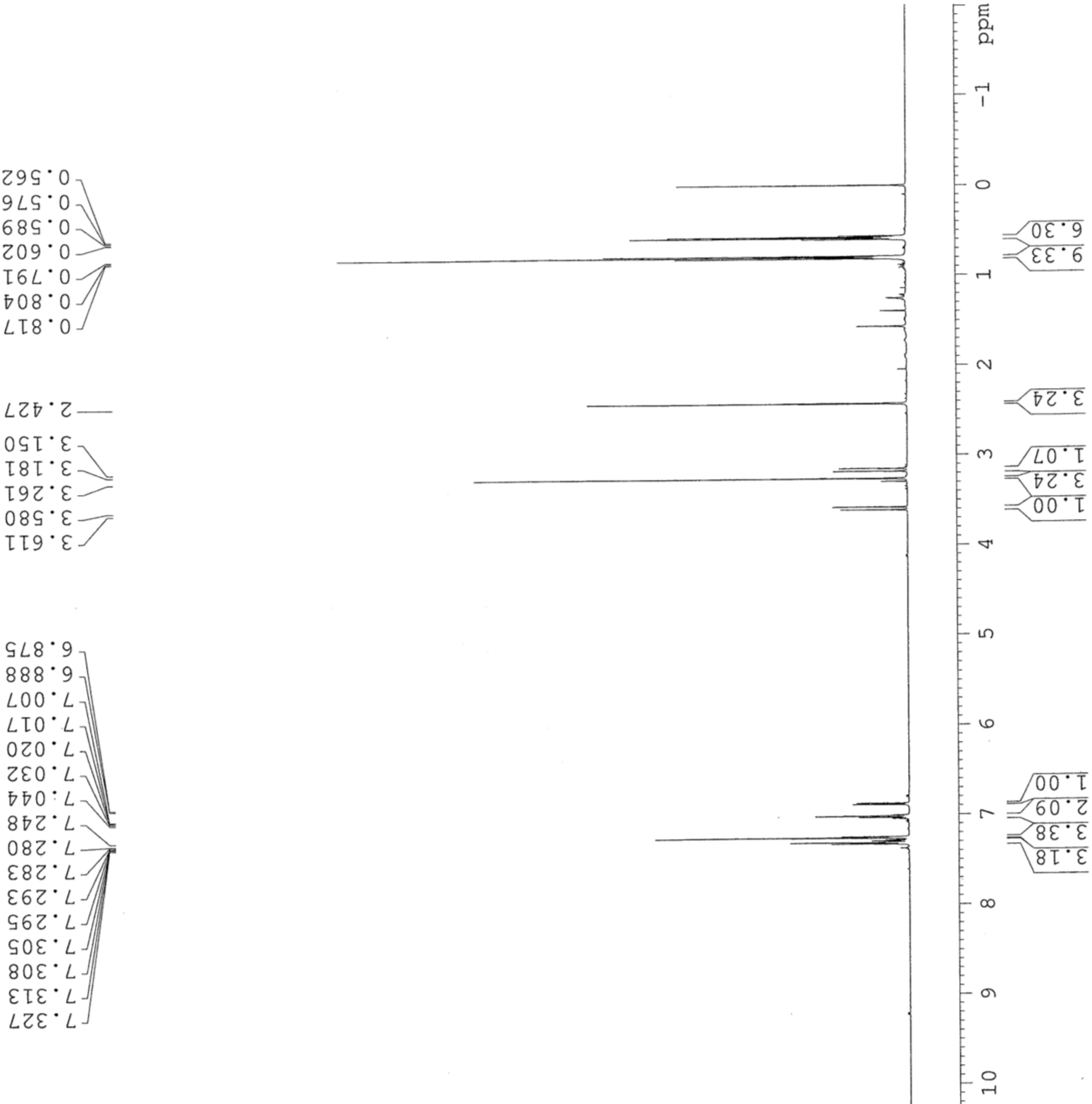
0.562
0.576
0.589
0.602
0.791
0.804
0.817
2.427
3.150
3.181
3.261
3.580
3.611
6.875
6.888
7.007
7.017
7.020
7.032
7.044
7.248
7.280
7.283
7.293
7.295
7.305
7.308
7.313
7.327
6.30
9.33
3.24
1.07
3.24
1.00
1.00
2.09
3.38
3.18
ppm

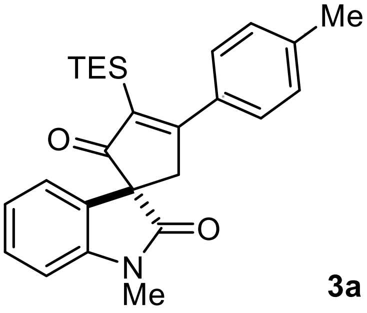
Me
TES
O
O
N
Me

3a

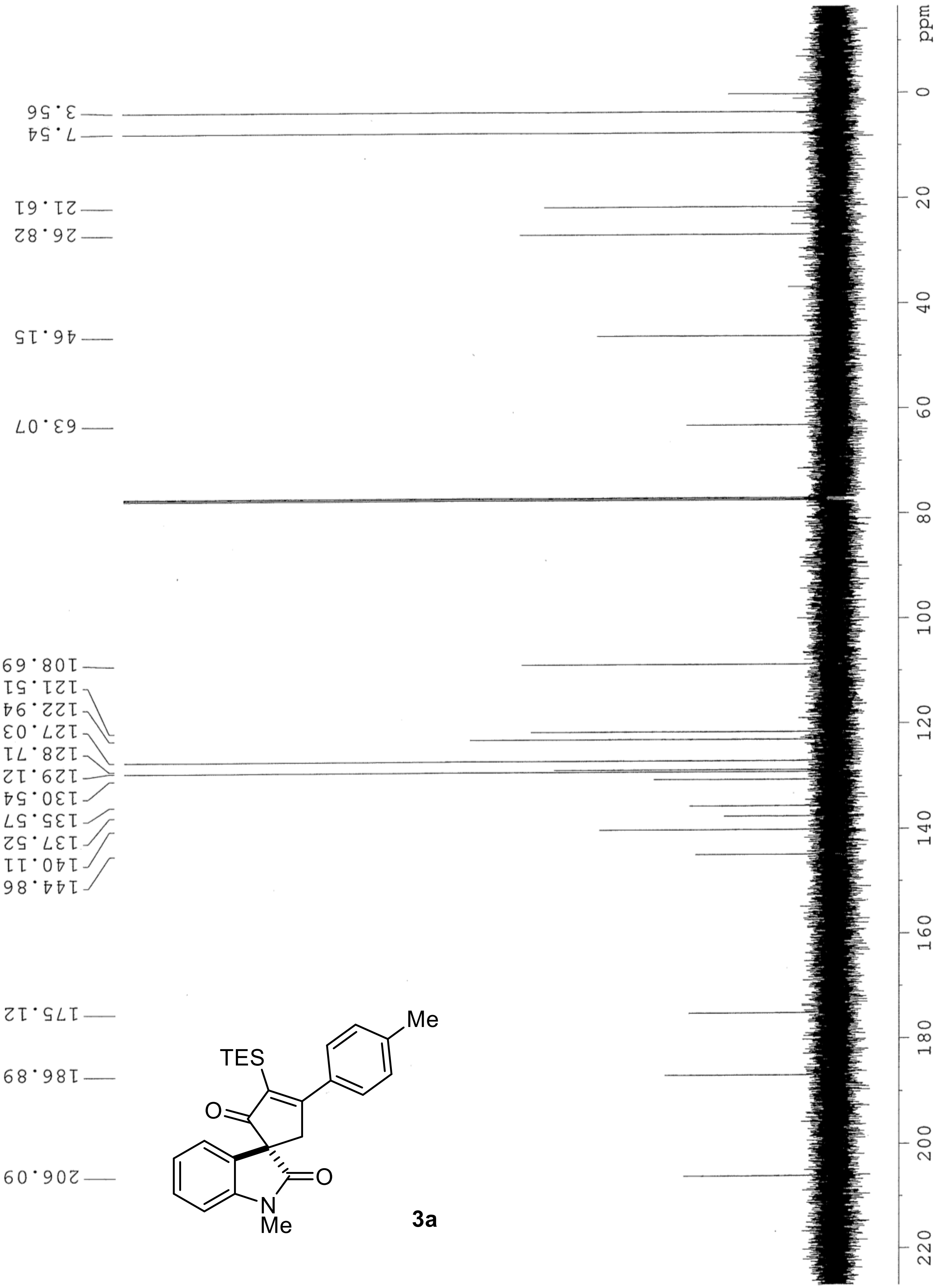
3.56
7.54
21.61
26.82
46.15
63.07
108.69
121.51
122.94
127.03
128.71
129.12
130.54
135.57
137.52
140.11
144.86
175.12
186.89
206.09
ppm
0
20
40
60
80
100
120
140
160
180
200
220
TES
Me
O
O
N
Me

3a

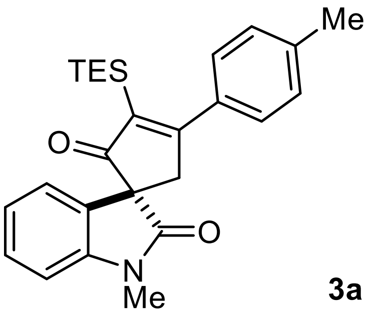

3a

Racemic

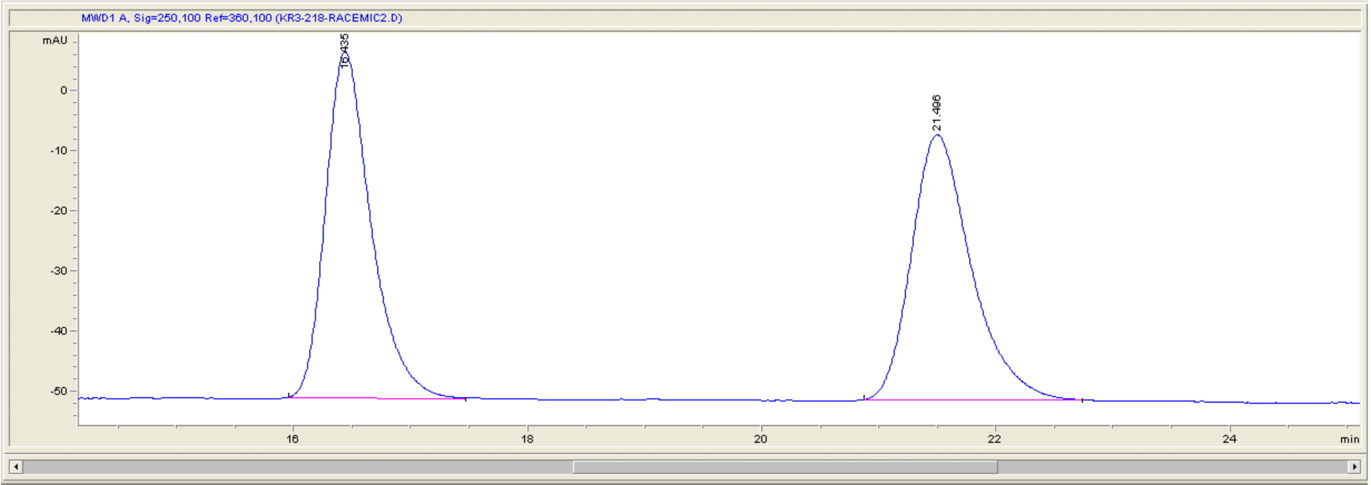

File Information	
LC-File	KR3-218-RACEMIC2.D
File Path	C:\CHEM32\2\DATA\ERIN\

#	Time	Area	Height	Width	Area%	Symmetry
1	16.435	1569.2	57.8	0.4125	49.747	0.696
2	21.496	1585.2	44.3	0.5417	50.253	0.713

Rh_2(*S*-TCPTTL)$_4$: 90% ee, 25 ^oC

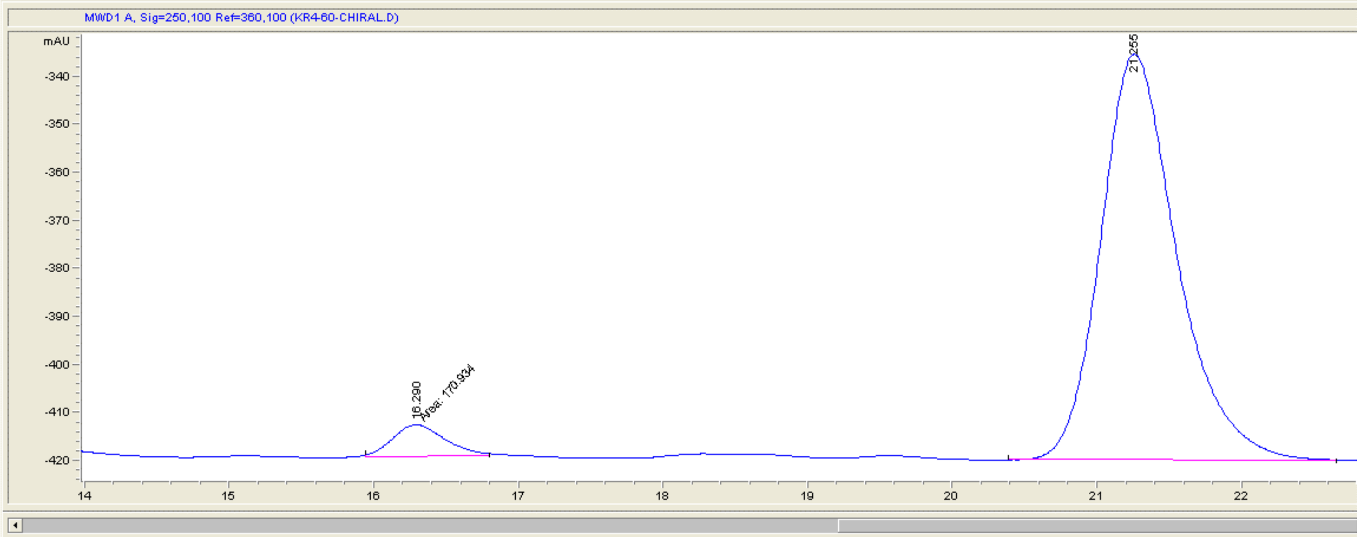

File Information	
LC-File	KR4-60-CHIRAL.D
File Path	C:\CHEM32\2\DATA\ERIN\

#	Time	Area	Height	Width	Area%	Symmetry
1	16.29	170.9	6.7	0.4268	5.238	0.805
2	21.255	3092.4	84.5	0.5569	94.762	0.746

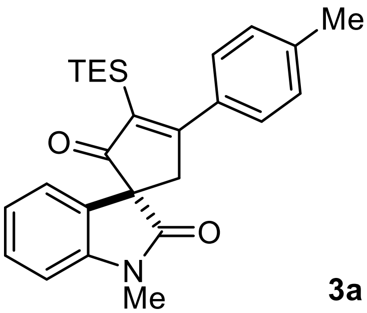

3a

Rh_2(*S*-TCPTTL)$_4$: 80% ee, 40 ^oC

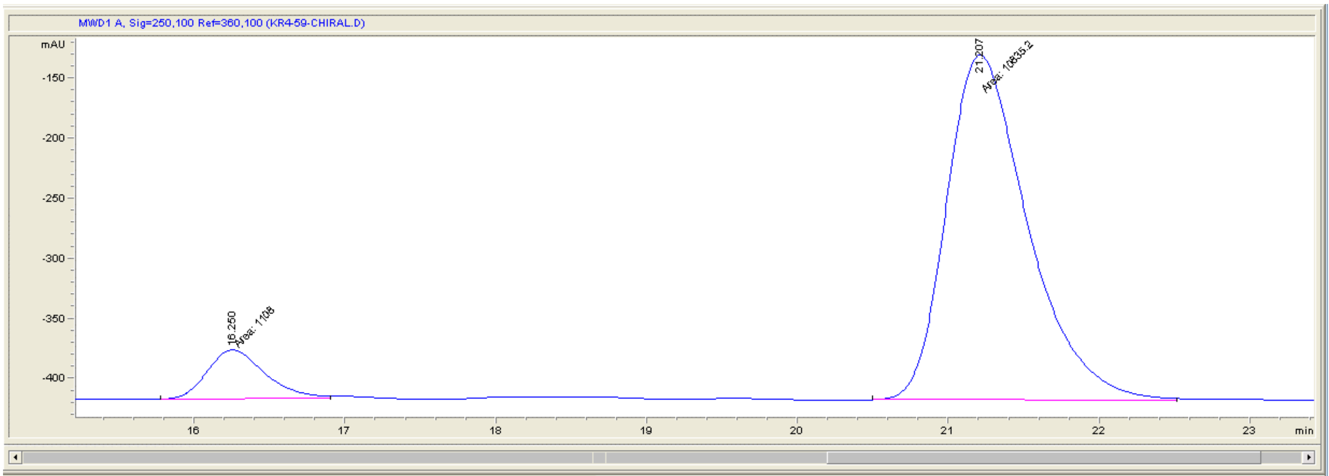

File Information

LC-File	KR4-59-CHIRAL.D

#	Time	Area	Height	Width	Area%	Symmetry
1	16.25	1108	41.1	0.4491	9.277	0.767
2	21.207	10835.2	287.6	0.628	90.723	0.676

Rh_2(*S*-TCPTTL)$_4$: 72% ee, 90 ^oC

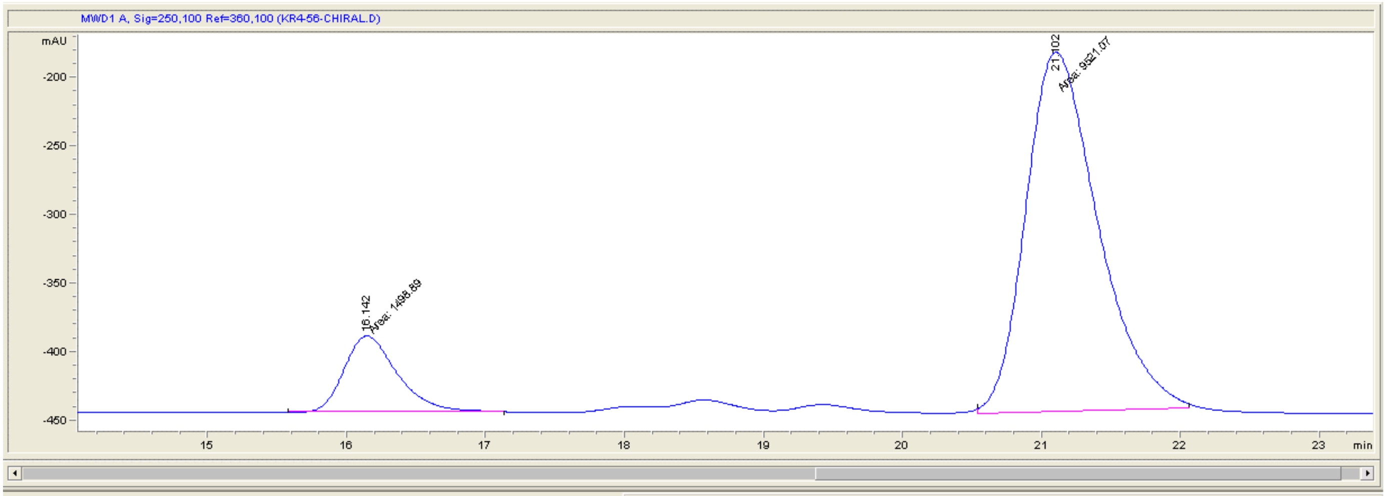

File Information

LC-File	KR4-56-CHIRAL.D

#	Time	Area	Height	Width	Area%	Symmetry
1	16.142	1498.9	55.7	0.4488	13.602	0.701
2	21.102	9521.1	262.3	0.6051	86.398	0.698

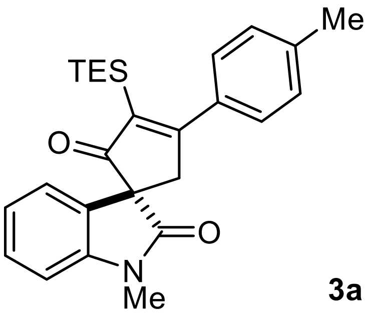

3a

Rh$_2$(*S*-TFPTTL)$_4$: 64% ee, 90 ^{o}C

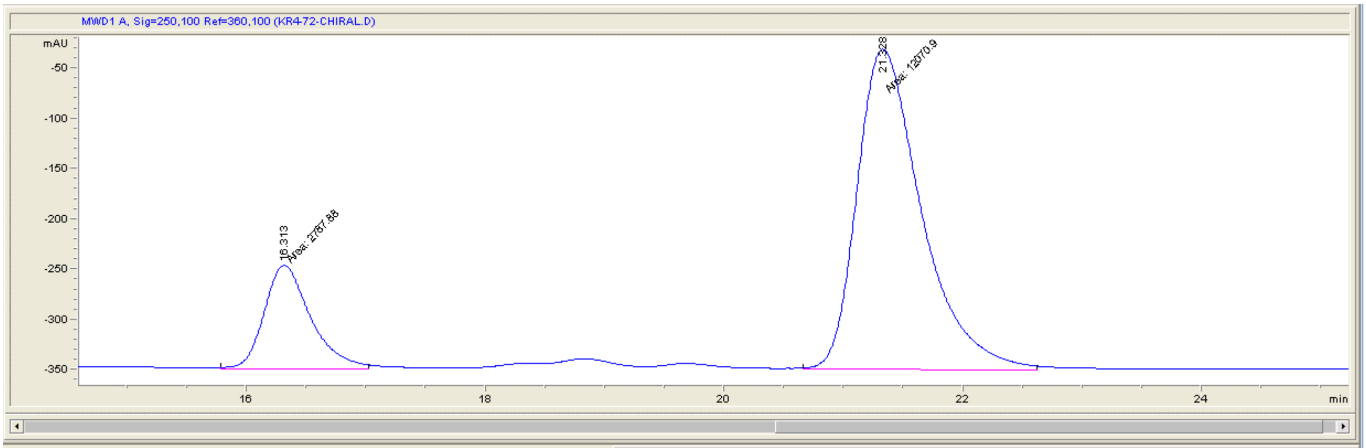

File Information

LC-File	KR4-72-CHIRAL.D

#	Time	Area	Height	Width	Area%	Symmetry
1	16.313	2787.9	103	0.4511	18.763	0.725
2	21.328	12070.9	319.4	0.6299	81.237	0.641

Rh$_2$(*R*-DOSP)$_4$: 16% ee, 90 ^{o}C

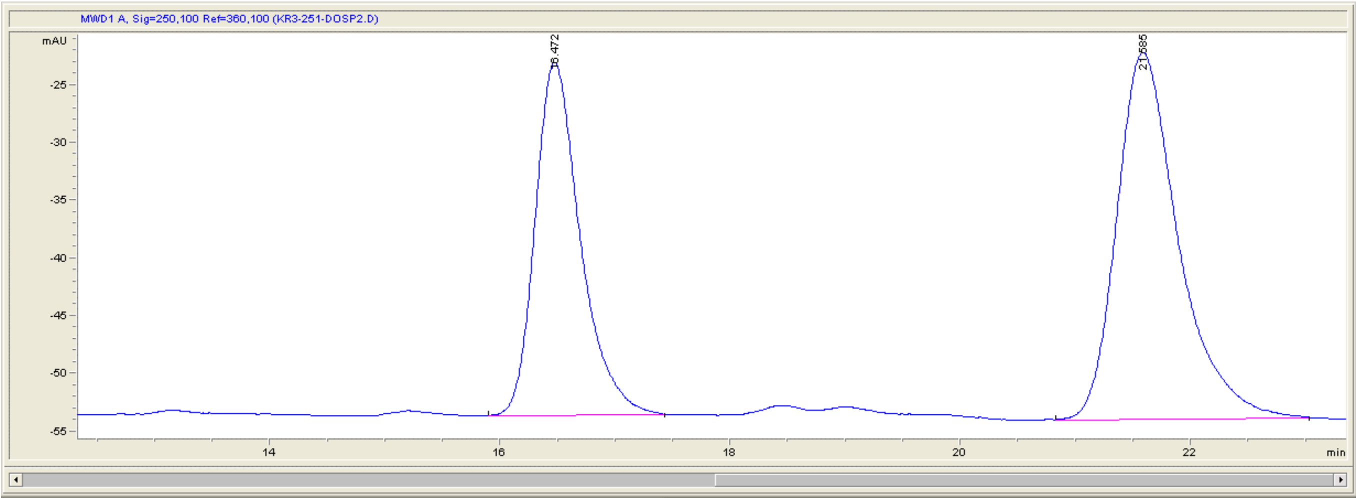

File Information

LC-File	KR3-251-DOSP2.D
File Path	C:\CHEM32\2\DATA\ERIN\

#	Time	Area	Height	Width	Area%	Symmetry
1	16.472	837.9	30.6	0.4153	41.405	0.712
2	21.585	1185.7	31.8	0.555	58.595	0.7

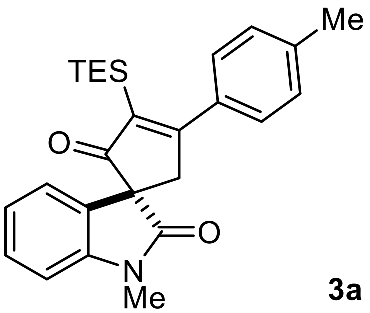

3a

Rh$_2$(*S*-NTTL)$_4$: 70% ee, 90 ^{o}C

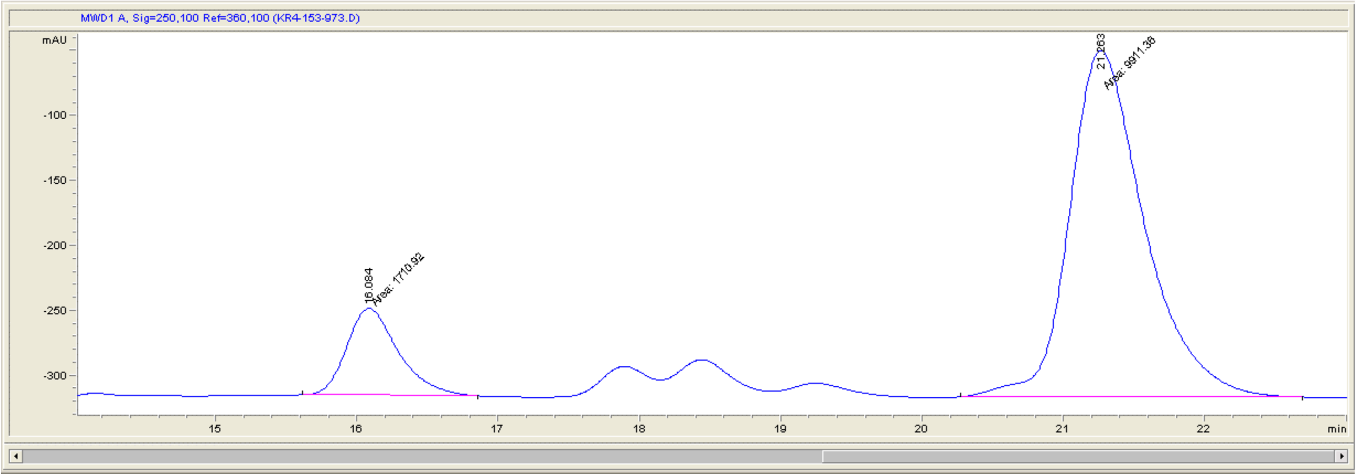

File Information	
LC-File	KR4-153-973.D
File Path	C:\CHEM32\2\DATA\ERIN\

#	Time	Area	Height	Width	Area%	Symmetry
1	16.084	1710.9	66.9	0.4261	14.721	0.727
2	21.263	9911.4	266.9	0.6188	85.279	0.711

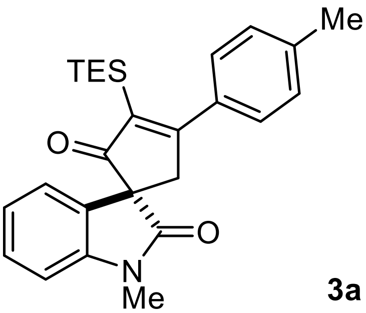

3a

Racemic, flow rate = 1.0 uL/min

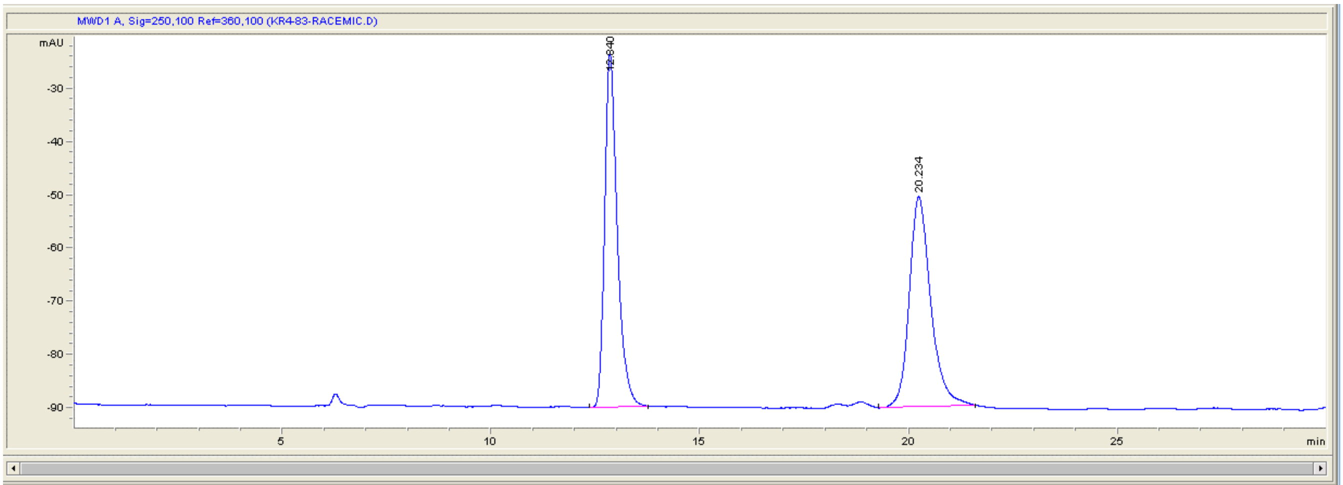

File Information	
LC-File	KR4-83-RACEMIC.D
File Path	C:\CHEM32\2\DATA\ERIN\

#	Time	Area	Height	Width	Area%	Symmetry
1	12.84	1396.6	66.3	0.3176	49.517	0.69
2	20.234	1423.8	39.5	0.5361	50.483	0.736

Rh_2(*R*-BTCP)$_4$: 28% ee, 90 ^oC

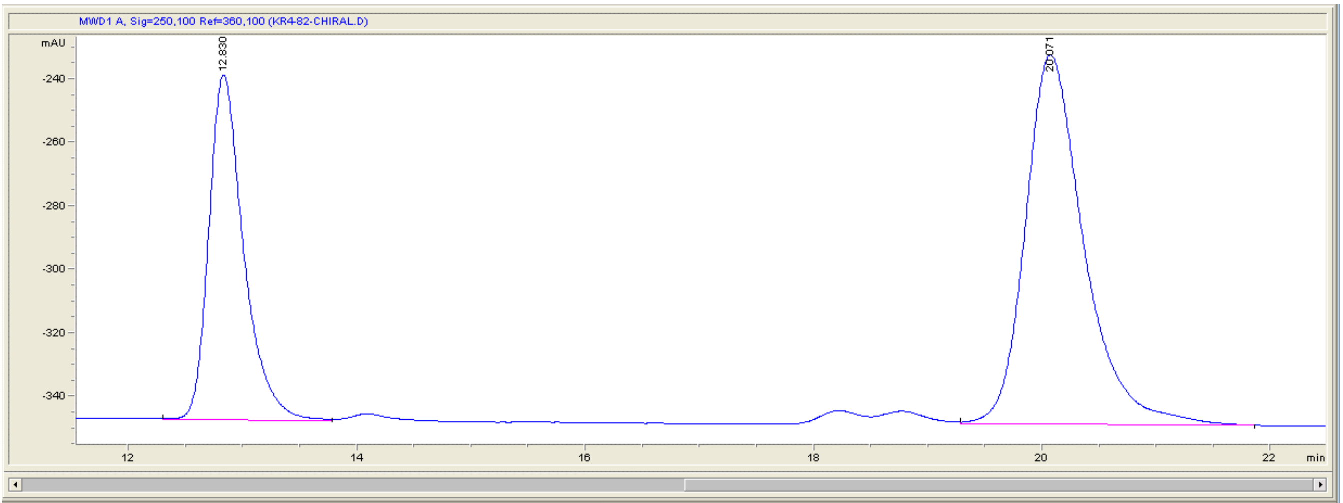

File Information	
LC-File	KR4-82-CHIRAL.D
File Path	C:\CHEM32\2\DATA\ERIN\

#	Time	Area	Height	Width	Area%	Symmetry
1	12.83	2318.3	108.8	0.3187	35.639	0.682
2	20.071	4186.7	116.6	0.5371	64.361	0.702

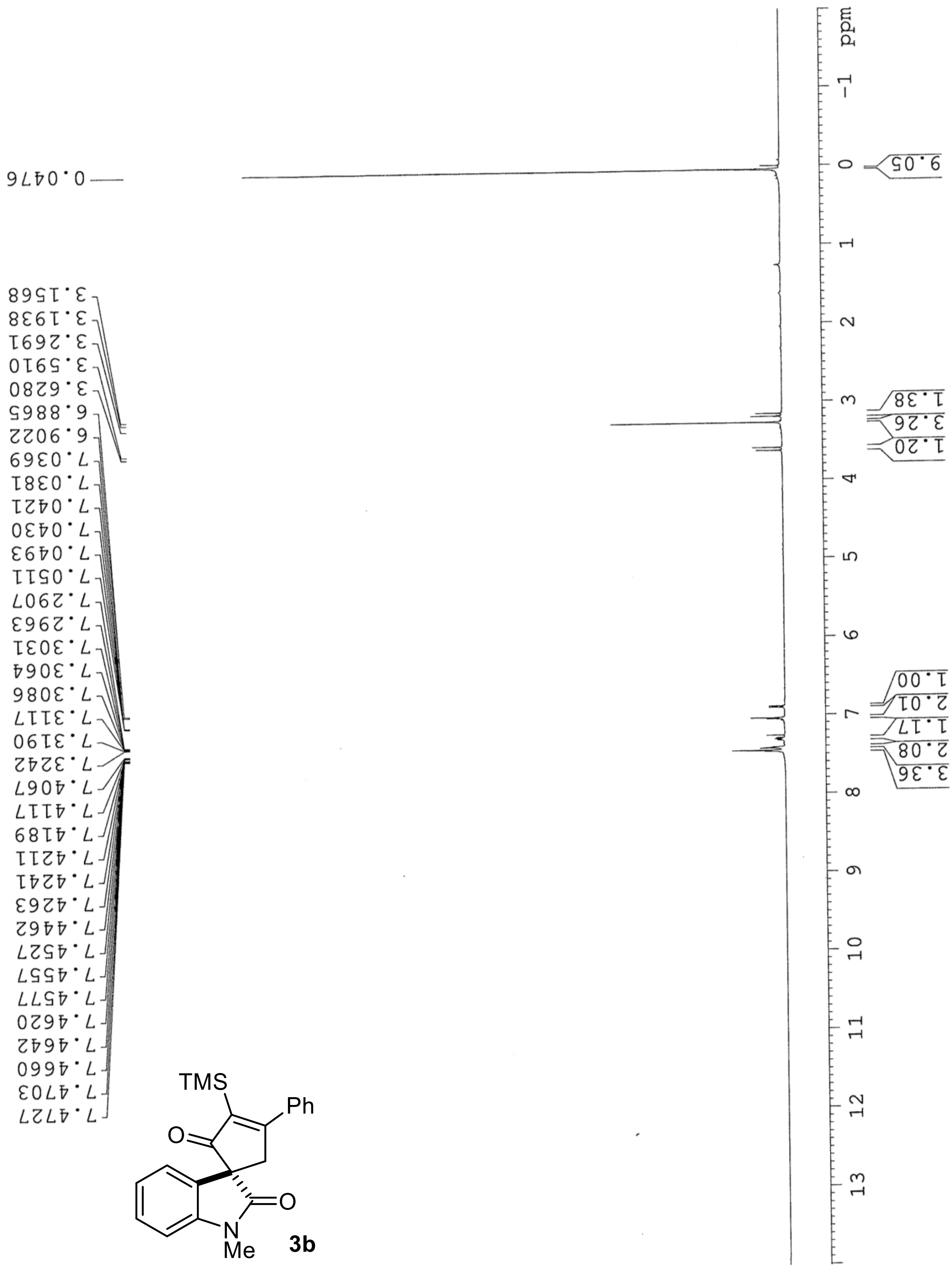
0.0476
3.1568
3.1938
3.2691
3.5910
3.6280
6.8865
6.9022
7.0369
7.0381
7.0421
7.0430
7.0493
7.0511
7.2907
7.2963
7.3031
7.3064
7.3086
7.3117
7.3190
7.3242
7.4067
7.4117
7.4189
7.4211
7.4241
7.4263
7.4462
7.4527
7.4557
7.4577
7.4620
7.4642
7.4660
7.4703
7.4727
9.05
1.38
3.26
1.20
1.00
2.01
1.17
2.08
3.36
ppm
TMS
Ph
O
O
N
Me

3b

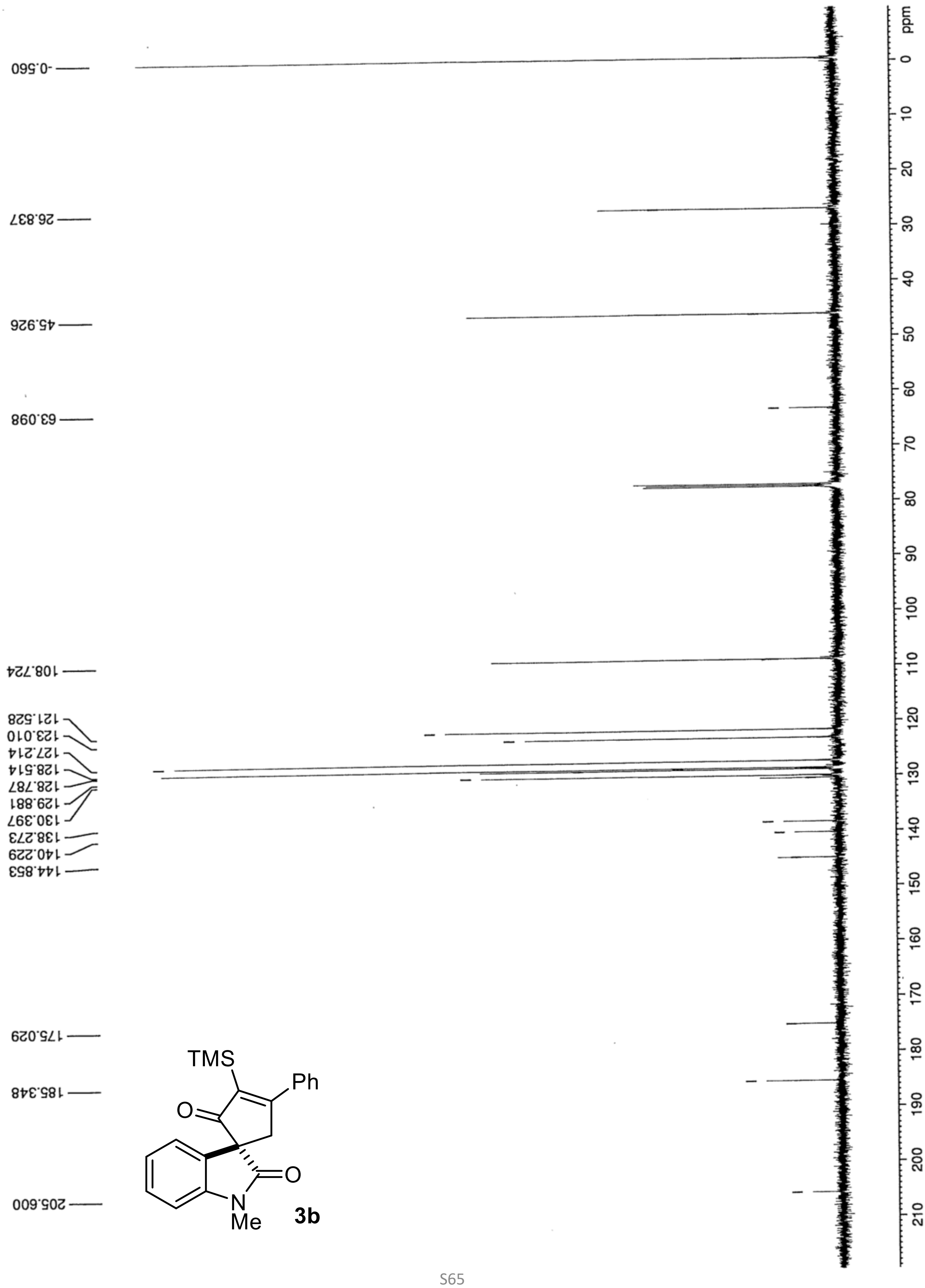
-0.560
26.837
45.926
63.098
108.724
121.528
123.010
127.214
128.514
128.787
129.881
130.397
138.273
140.229
144.853
175.029
185.348
205.600
ppm
TMS
Ph
O
N
Me
3b

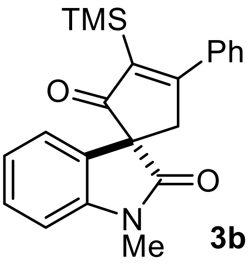

3b

Racemic

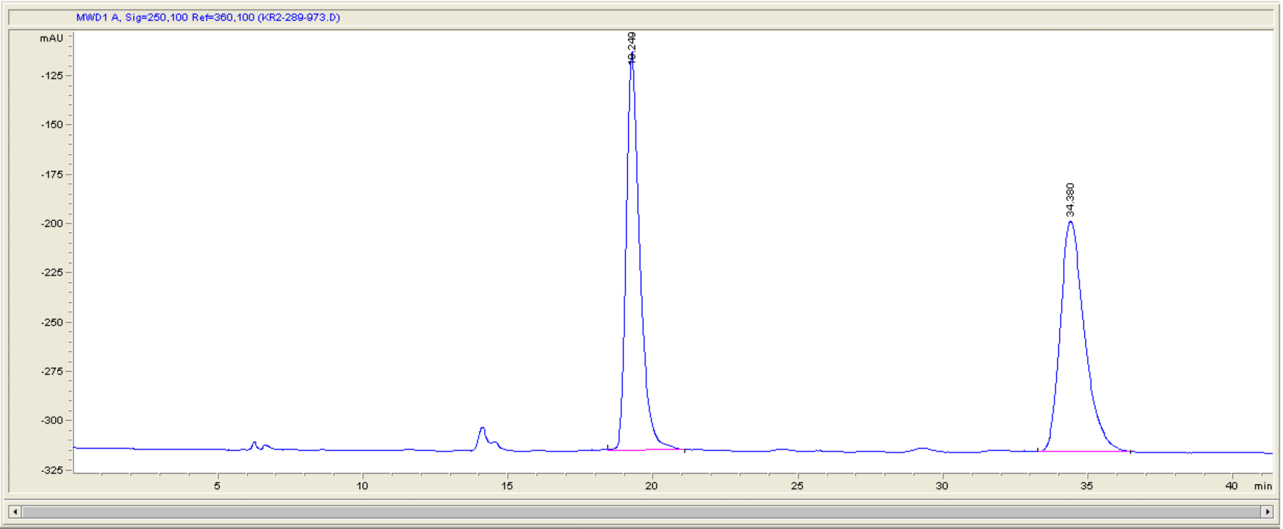

File Information	
LC-File	KR2-289-973.D
File Path	C:\CHEM32\2\DATA\ERIN\

#	Time	Area	Height	Width	Area%	Symmetry
1	19.249	6609.1	202.3	0.4912	49.175	0.648
2	34.38	6831	117.1	0.8706	50.825	0.701

Rh_2(*S*-TCPTTL)$_4$: 90% ee, 25 ^oC

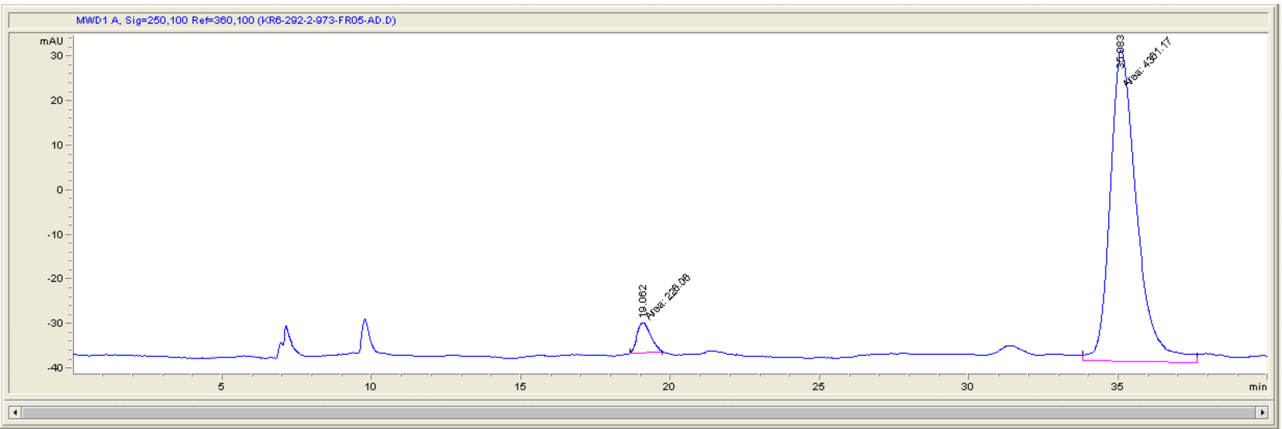

File Information	
LC-File	KR6-292-2-973-FR05-AD.D
File Path	C:\CHEM32\2\DATA\ERIN\

#	Time	Area	Height	Width	Area%	Symmetry
1	19.062	226.1	7	0.542	4.928	0.701
2	35.083	4361.2	69.9	1.0404	95.072	0.695

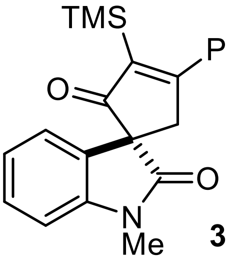

3b

Rh$_2$(*S*-TCPTTL)$_4$: >99% ee
After recrystallization

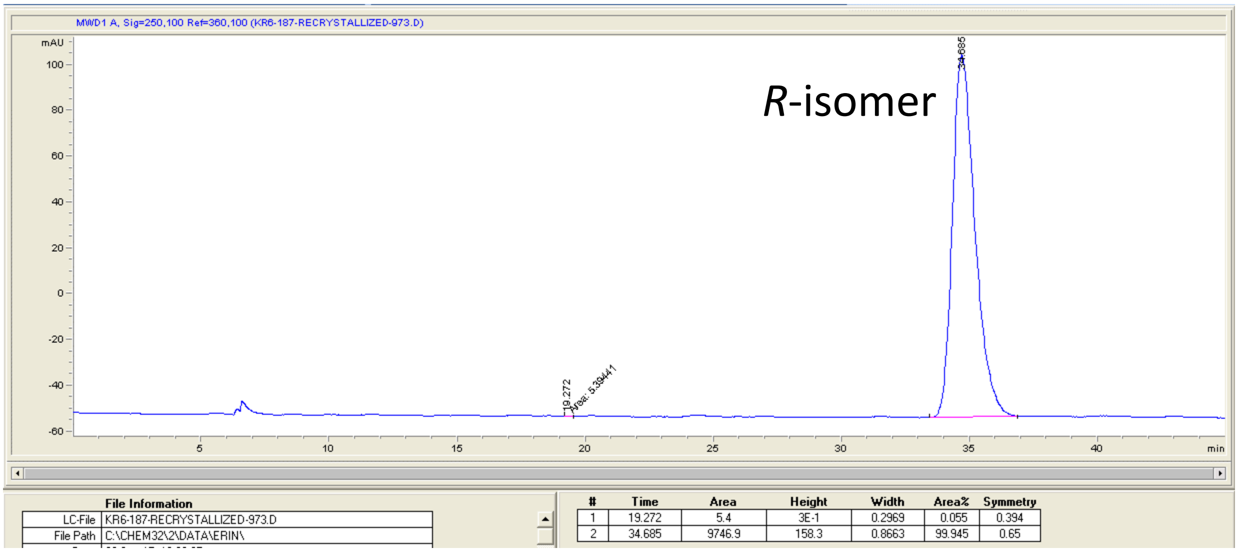

#	Time	Area	Height	Width	Area%	Symmetry
1	19.272	5.4	3E-1	0.2969	0.055	0.394
2	34.685	9746.9	158.3	0.8663	99.945	0.65

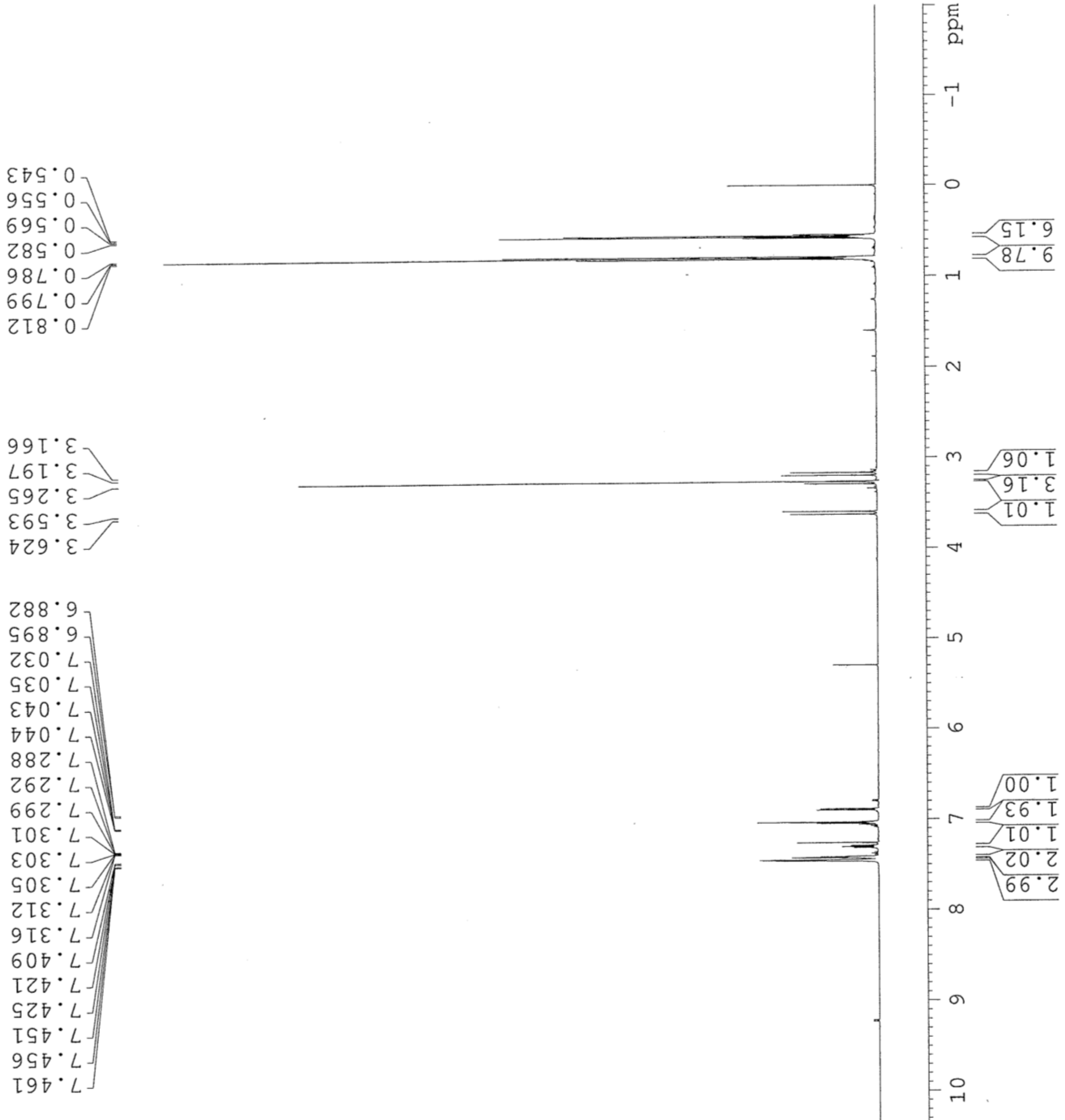

0.543
0.556
0.569
0.582
0.786
0.799
0.812
3.166
3.197
3.265
3.593
3.624
6.882
6.895
7.032
7.035
7.043
7.044
7.288
7.292
7.299
7.301
7.303
7.305
7.312
7.316
7.409
7.421
7.425
7.451
7.456
7.461
6.15
9.78
1.06
3.16
1.01
1.00
1.93
1.01
2.02
2.99
ppm

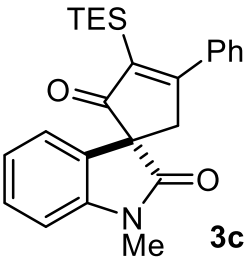

TES
Ph
O
O
N
Me

3c

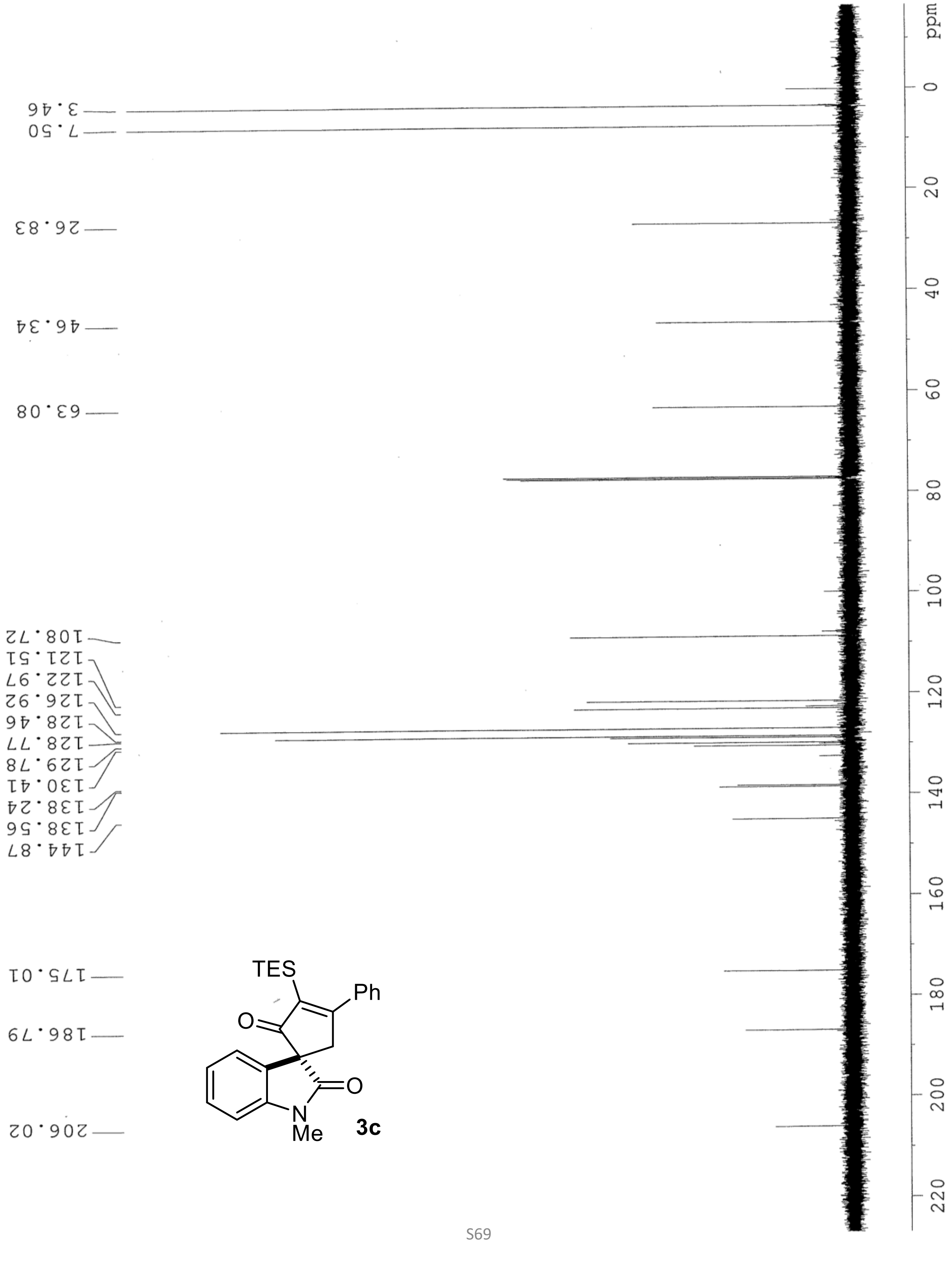
3.46
7.50
26.83
46.34
63.08
108.72
121.51
122.97
126.92
128.46
128.77
129.78
130.41
138.24
138.56
144.87
175.01
186.79
206.02
ppm
0
20
40
60
80
100
120
140
160
180
200
220
TES
Ph
O
O
N
Me
3c

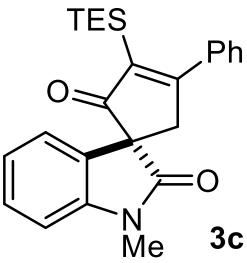

3c

Racemic

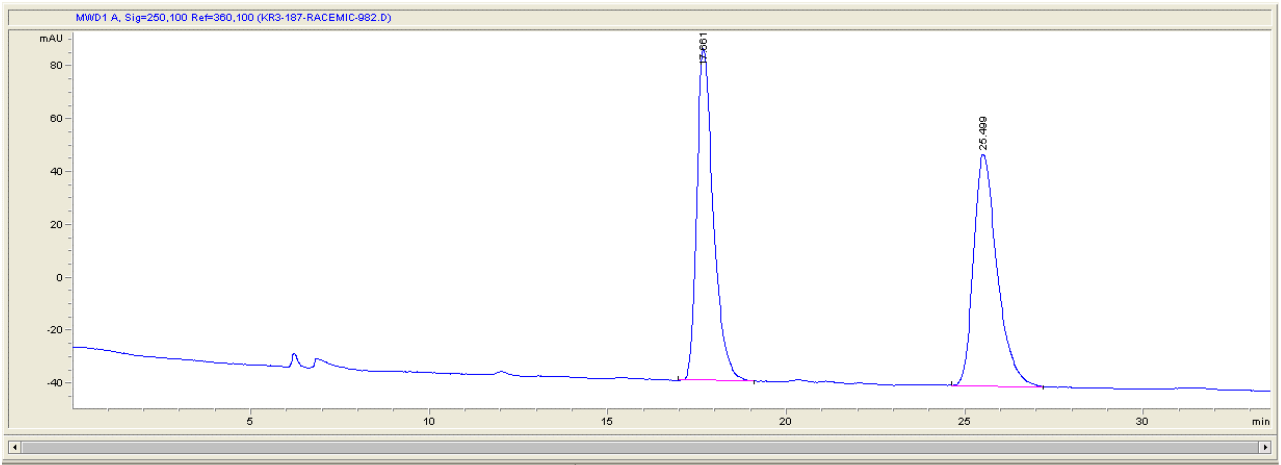

File Information	
LC-File	KR3-187-RACEMIC-982.D
File Path	C:\CHEM32\2\DATA\ERIN\

#	Time	Area	Height	Width	Area%	Symmetry
1	17.661	4066.6	125.4	0.4884	50.109	0.634
2	25.499	4048.9	87.7	0.6864	49.891	0.664

Rh_2(*S*-TCPTTL)$_4$: 92% ee

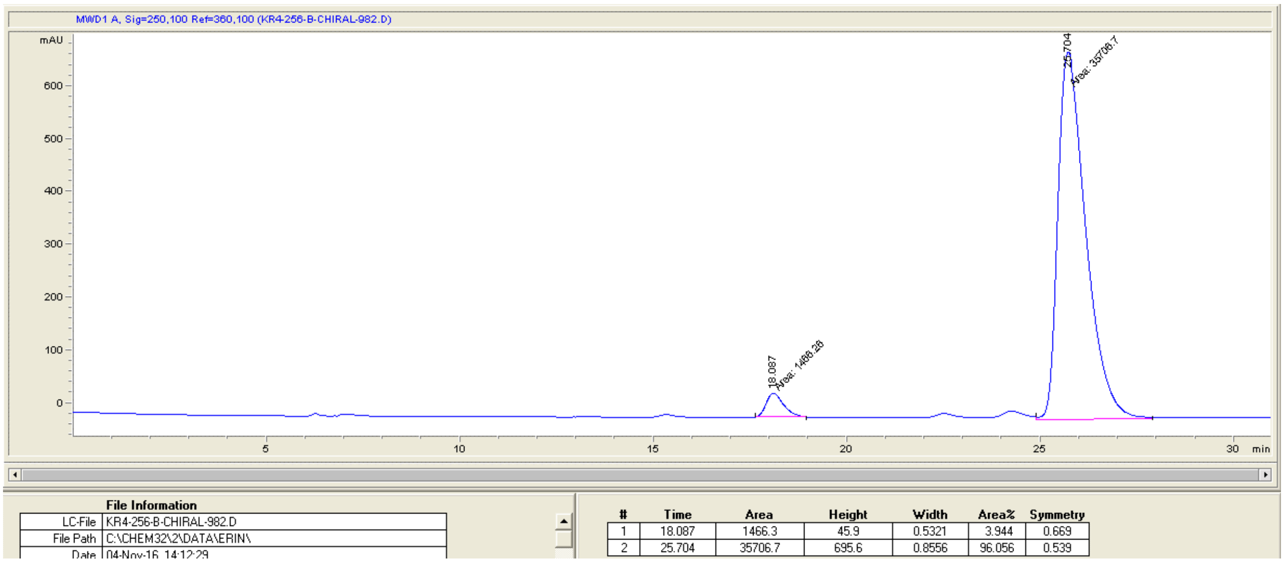

File Information	
LC-File	KR4-256-B-CHIRAL-982.D
File Path	C:\CHEM32\2\DATA\ERIN\
Date	04-Nov-16, 14:12:29

#	Time	Area	Height	Width	Area%	Symmetry
1	18.087	1466.3	45.9	0.5321	3.944	0.669
2	25.704	35706.7	695.6	0.8556	96.056	0.539

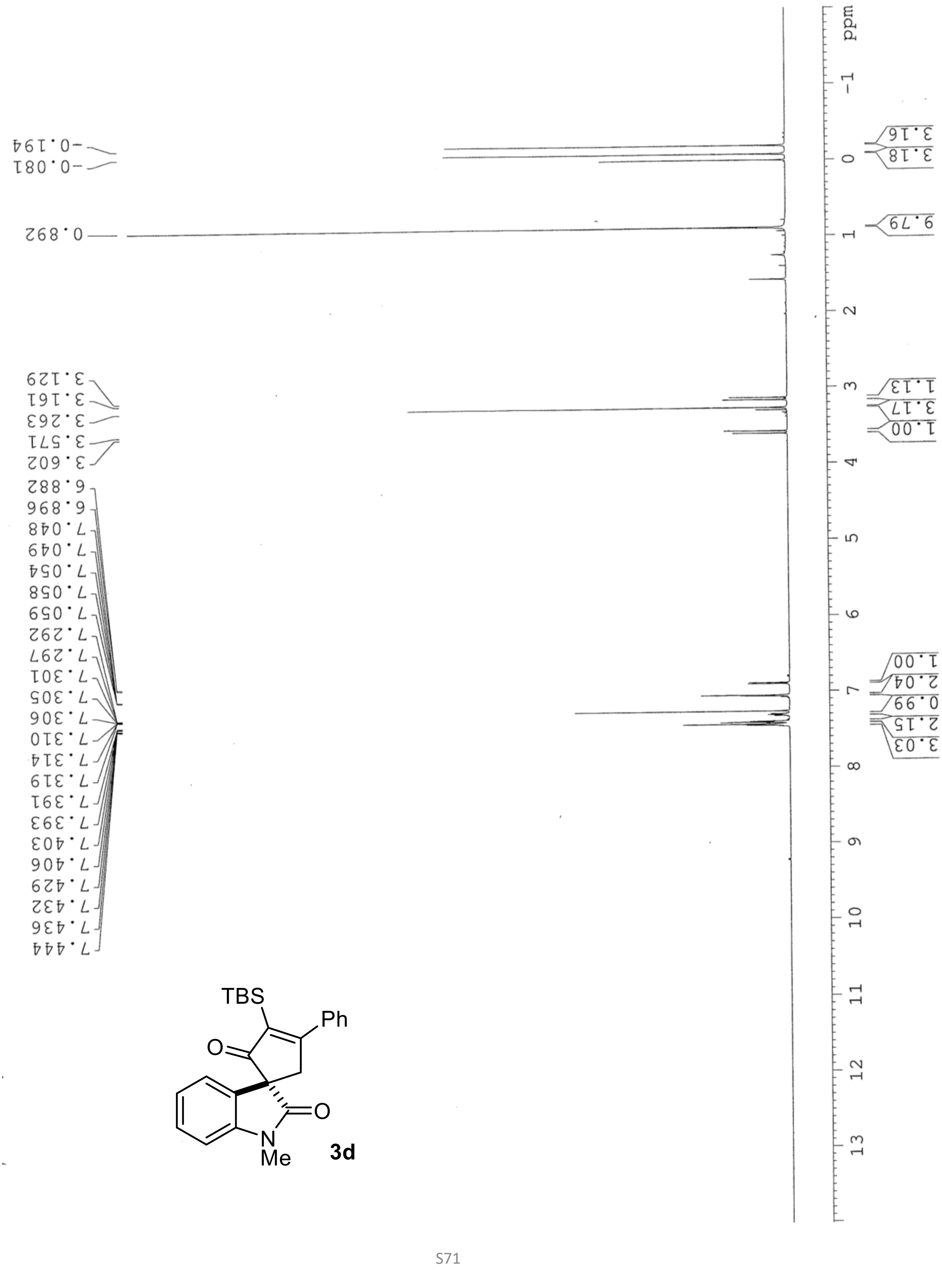
-0.194
-0.081
0.892
3.129
3.161
3.263
3.571
3.602
6.882
6.896
7.048
7.049
7.054
7.058
7.059
7.292
7.297
7.301
7.305
7.306
7.310
7.314
7.319
7.391
7.393
7.403
7.406
7.429
7.432
7.436
7.444
3.16
3.18
9.79
1.13
3.17
1.00
1.00
2.04
0.99
2.15
3.03
ppm
-1 0 1 2 3 4 5 6 7 8 9 10 11 12 13
TBS
Ph
O
O
N
Me
3d

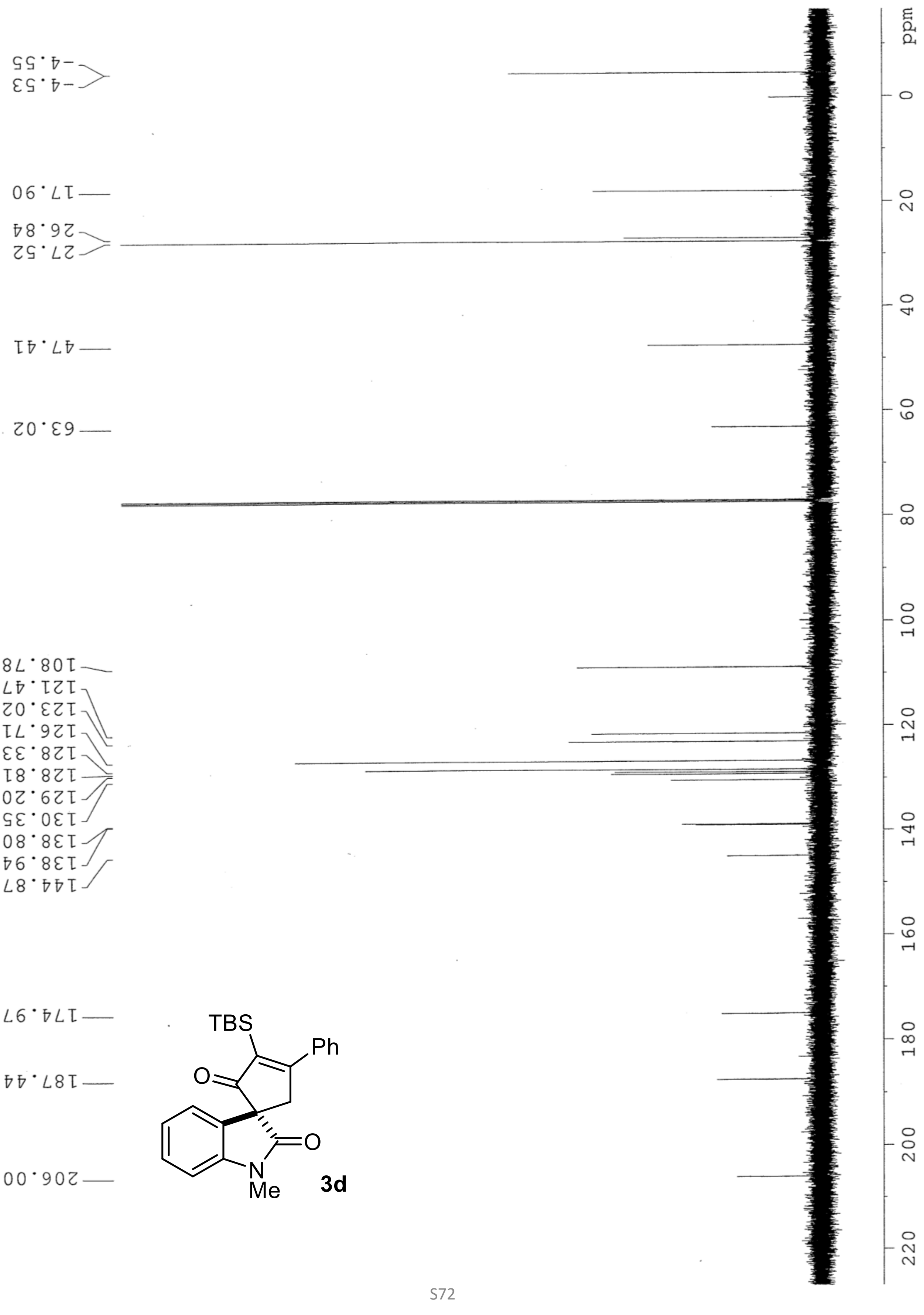
-4.55
-4.53
17.90
26.84
27.52
47.41
63.02
108.78
121.47
123.02
126.71
128.33
128.81
129.20
130.35
138.80
138.94
144.87
174.97
187.44
206.00
TBS
Ph
O
O
N
Me
3d
ppm
0
20
40
60
80
100
120
140
160
180
200
220

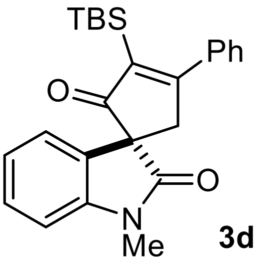

3d

Racemic

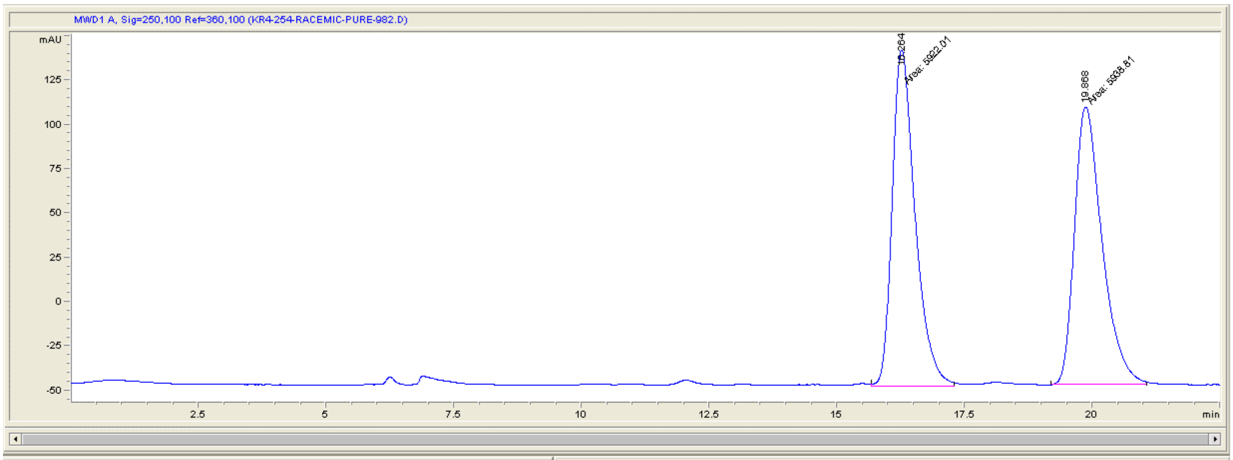

File Information

LC-File	KR4-254-RACEMIC-PURE-982.D
File Path	C:\CHEM32\2\DATA\ERIN\
Date	02-Nov-16, 15:28:09

#	Time	Area	Height	Width	Area%	Symmetry
1	16.264	5922	189.5	0.5209	49.929	0.671
2	19.868	5938.8	156.9	0.6309	50.071	0.653

Rh_2(*S*-TCPTTL)$_4$: 90% ee

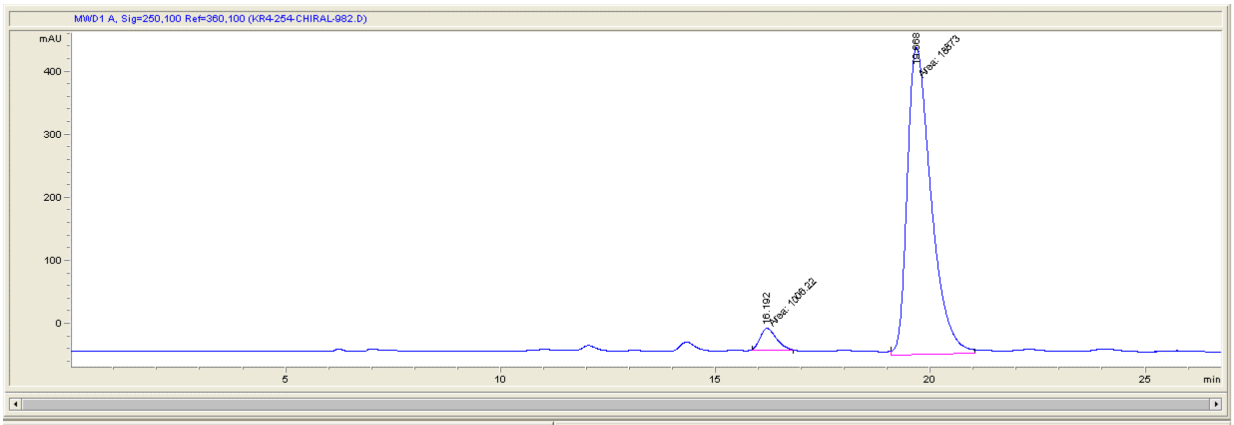

File Information

LC-File	KR4-254-CHIRAL-982.D
File Path	C:\CHEM32\2\DATA\ERIN\
Date	02-Nov-16, 14:08:49

#	Time	Area	Height	Width	Area%	Symmetry
1	16.192	1006.2	36.2	0.4635	5.062	0.698
2	19.668	18873	487.2	0.6456	94.938	0.595

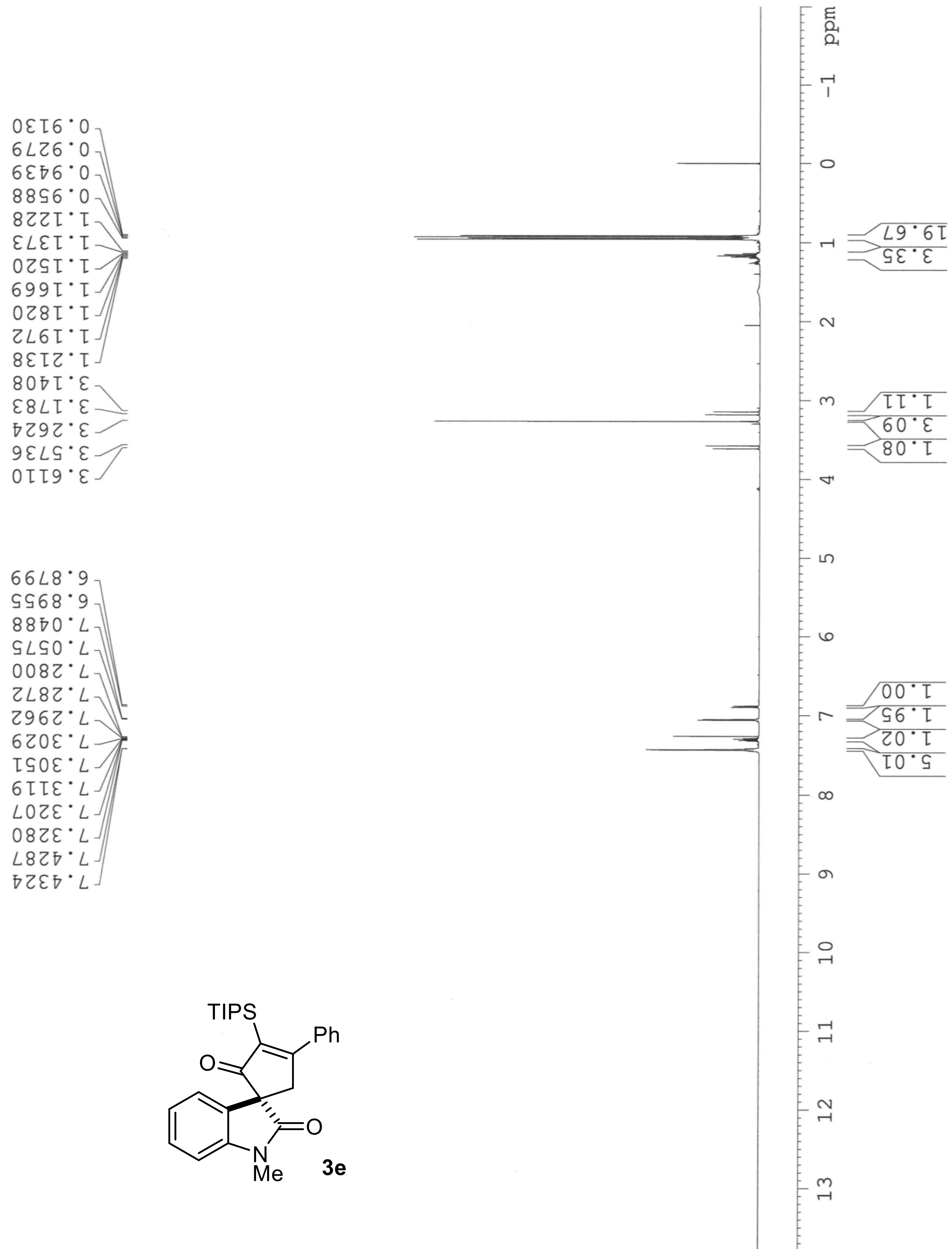
0.9130
0.9279
0.9439
0.9588
1.1228
1.1373
1.1520
1.1669
1.1820
1.1972
1.2138
3.1408
3.1783
3.2624
3.5736
3.6110
6.8799
6.8955
7.0488
7.0575
7.2800
7.2872
7.2962
7.3029
7.3051
7.3119
7.3207
7.3280
7.4287
7.4324
19.67
3.35
1.11
3.09
1.08
1.00
1.95
1.02
5.01
ppm
TIPS
Ph
O
O
N
Me
3e

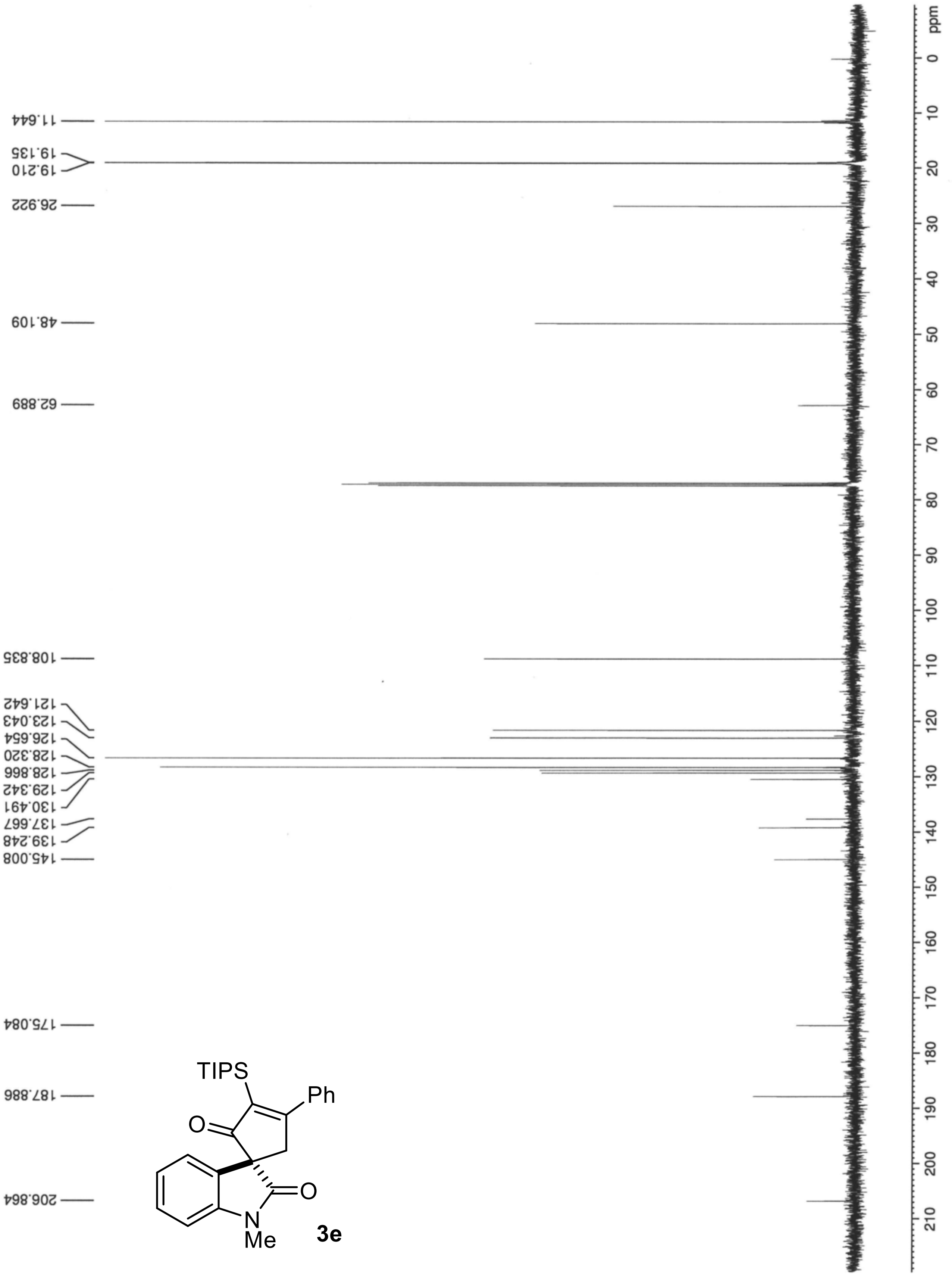

11.644
19.135
19.210
26.922
48.109
62.889
108.835
121.642
123.043
126.654
128.320
128.866
129.342
130.491
137.667
139.248
145.008
175.084
187.886
206.864
ppm
TIPS
Ph
O
O
N
Me
3e

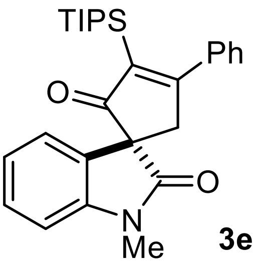

3e

Racemic

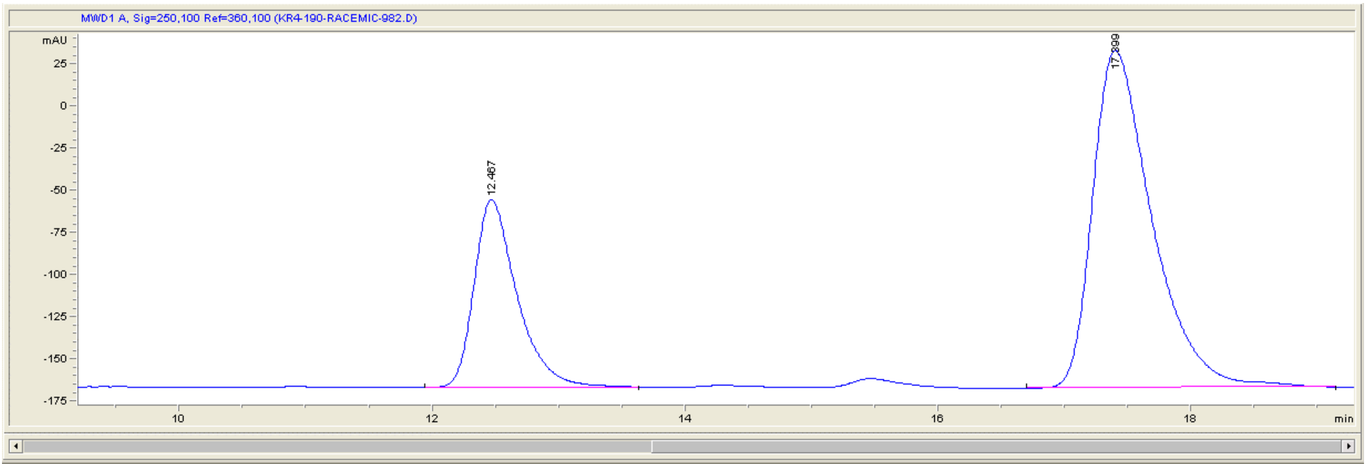

File Information

LC-File	KR4-190-RACEMIC-982.D
File Path	C:\CHEM32\2\DATA\ERIN\

#	Time	Area	Height	Width	Area%	Symmetry
1	12.467	2656	111.5	0.358	28.829	0.627
2	17.399	6556.9	200.2	0.4941	71.171	0.569

Rh_2(*S*-TCPTTL)$_4$: 90% ee

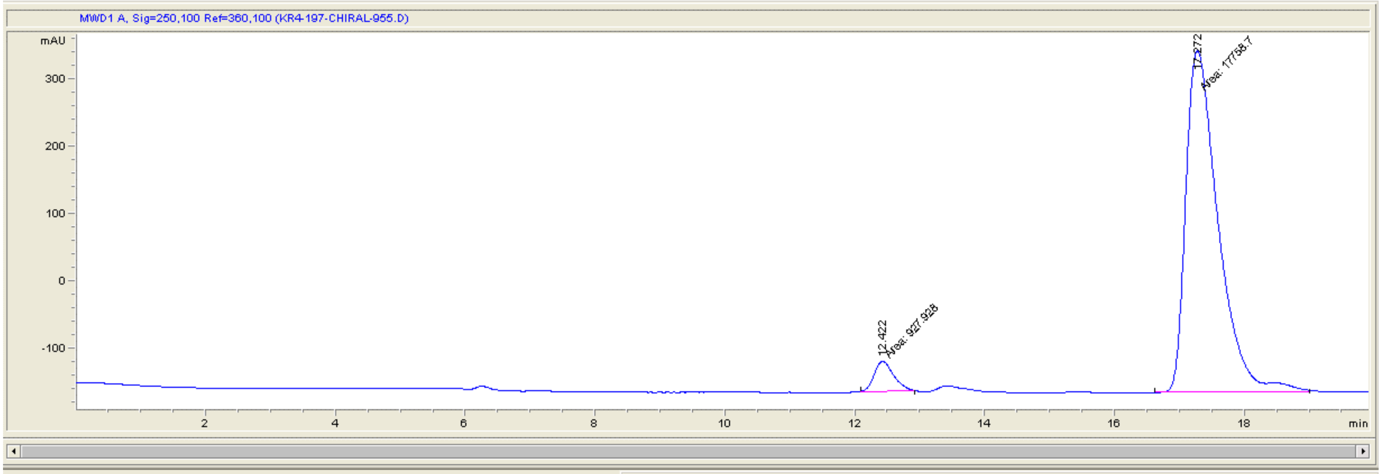

#	Time	Area	Height	Width	Area%	Symmetry
1	12.422	927.9	44.6	0.3465	4.966	0.74
2	17.272	17758.7	508.1	0.5825	95.034	0.532

File Information

LC-File	KR4-197-CHIRAL-955.D

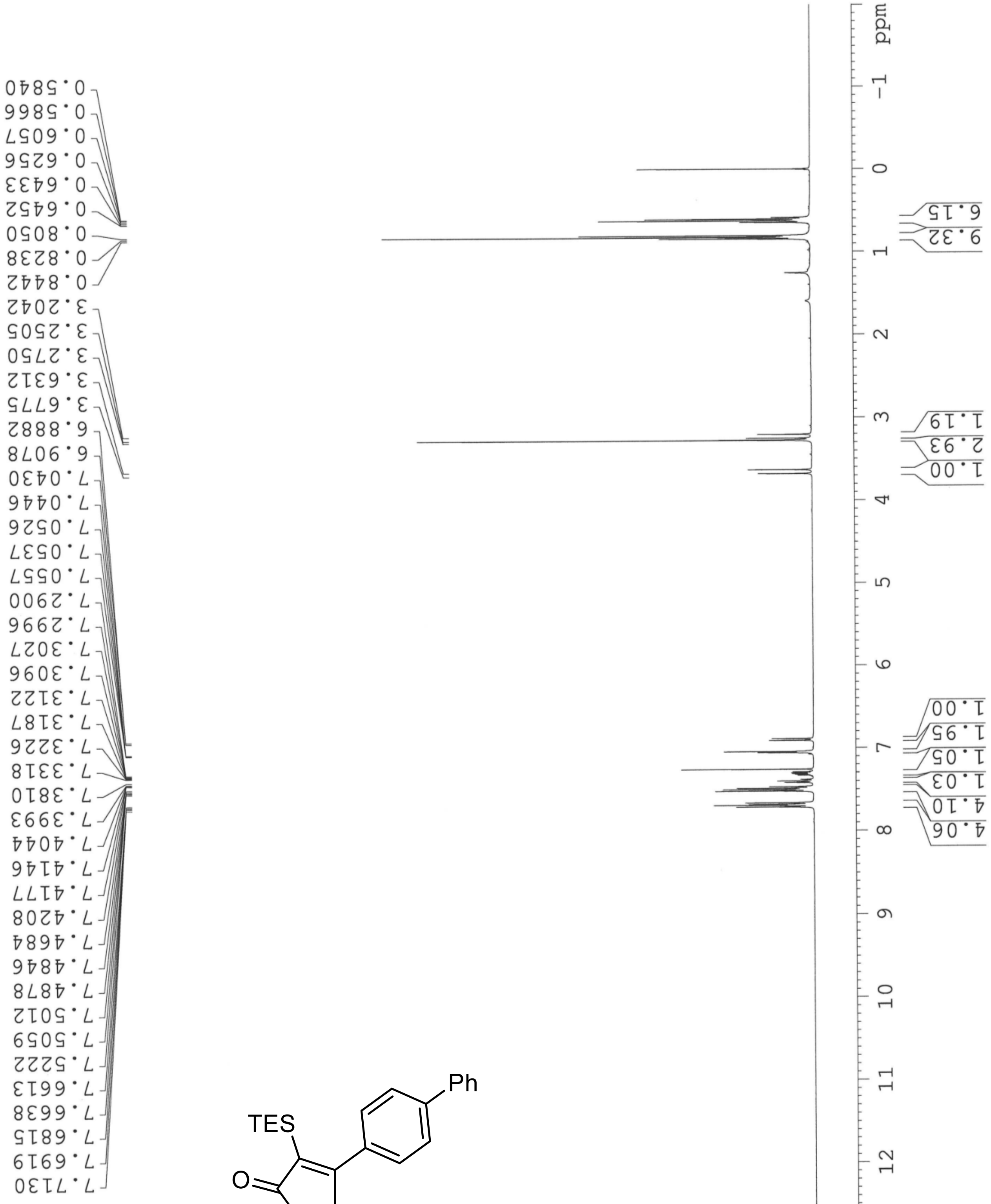

0.5840
0.5866
0.6057
0.6256
0.6433
0.6452
0.8050
0.8238
0.8442
3.2042
3.2505
3.2750
3.6312
3.6775
6.8882
6.9078
7.0430
7.0446
7.0526
7.0537
7.0557
7.2900
7.2996
7.3027
7.3096
7.3122
7.3187
7.3226
7.3318
7.3810
7.3993
7.4044
7.4146
7.4177
7.4208
7.4684
7.4846
7.4878
7.5012
7.5059
7.5222
7.6613
7.6638
7.6815
7.6919
7.7130
6.15
9.32
1.19
2.93
1.00
1.00
1.95
1.05
1.03
4.10
4.06
ppm

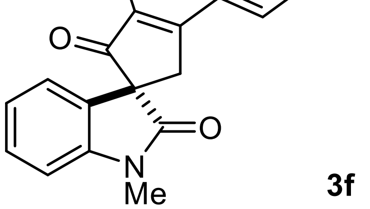

Ph
TES
O
O
N
Me

3f

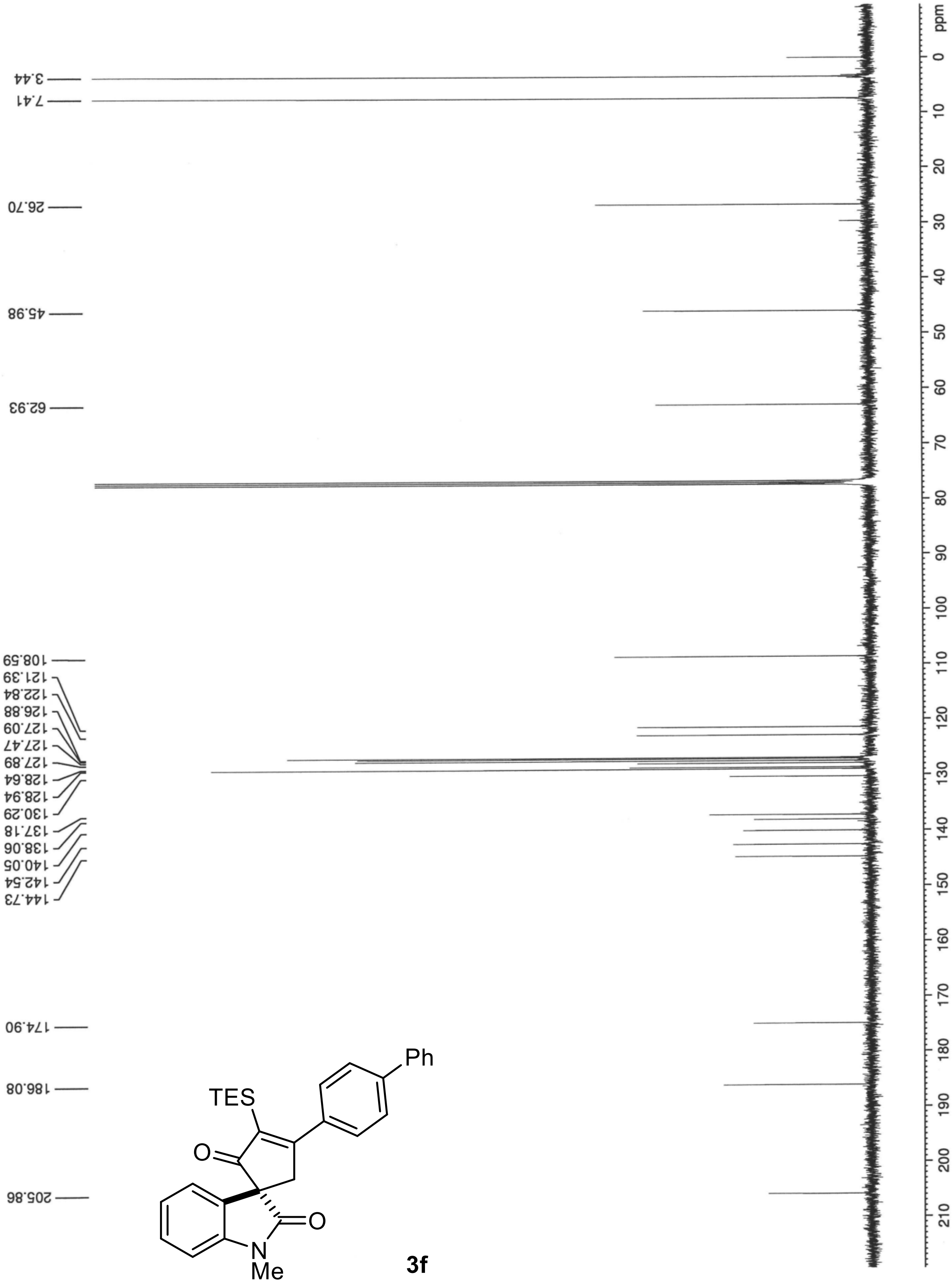
3.44
7.41
26.70
45.98
62.93
108.59
121.39
122.84
126.88
127.09
127.47
127.89
128.64
128.94
130.29
137.18
138.06
140.05
142.54
144.73
174.90
186.08
205.86
ppm
0 10 20 30 40 50 60 70 80 90 100 110 120 130 140 150 160 170 180 190 200 210
TES
Ph
O
O
N
Me

3f

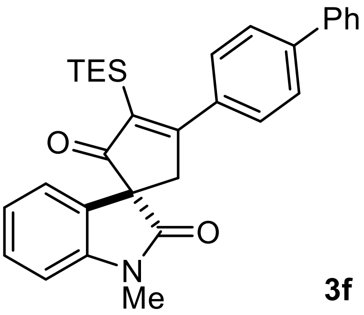

3f

Racemic

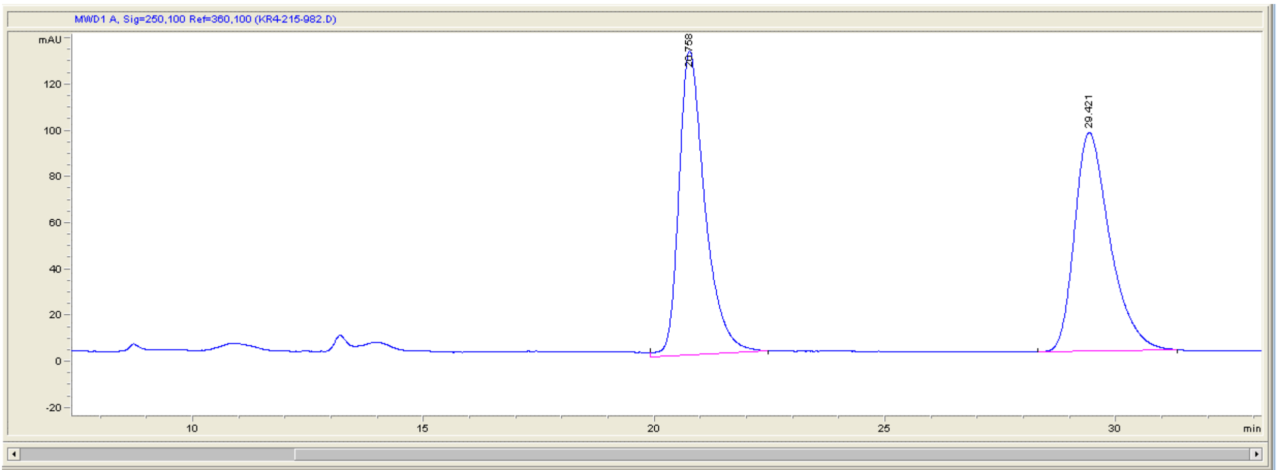

File Information

LC-File	KR4-215-982.D
File Path	C:\CHEM32\2\DATA\ERIN\

#	Time	Area	Height	Width	Area%	Symmetry
1	20.758	5204.2	131.9	0.5843	50.700	0.656
2	29.421	5060.6	94.7	0.7795	49.300	0.678

Rh_2(*S*-TCPTTL)$_4$: 86% ee

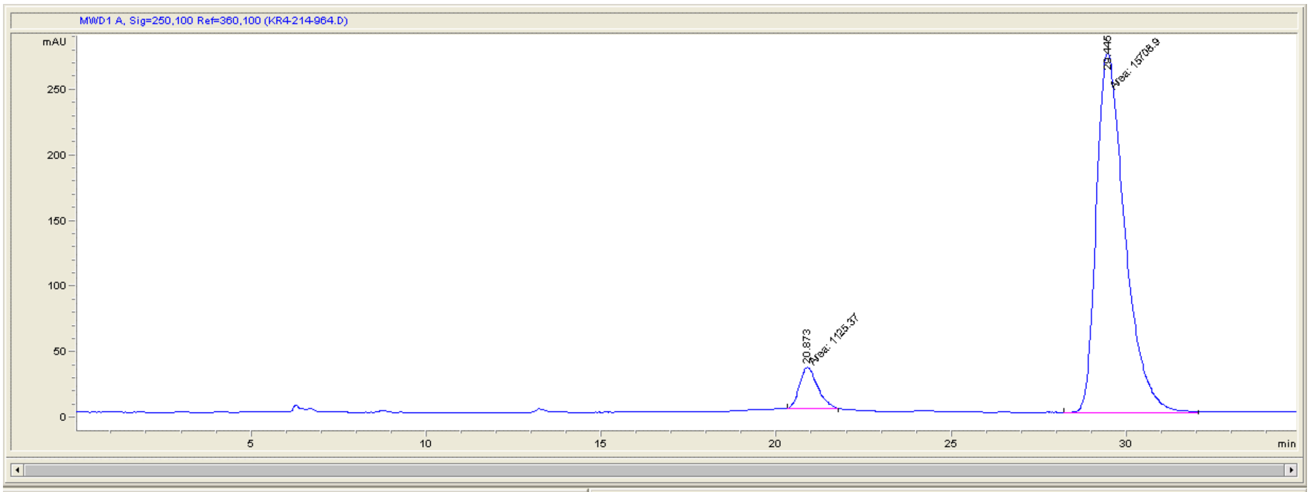

File Information

LC-File	KR4-214-964.D
File Path	C:\CHEM32\2\DATA\ERIN\

#	Time	Area	Height	Width	Area%	Symmetry
1	20.873	1125.4	31.2	0.601	6.685	0.746
2	29.445	15708.9	274.7	0.9529	93.315	0.613

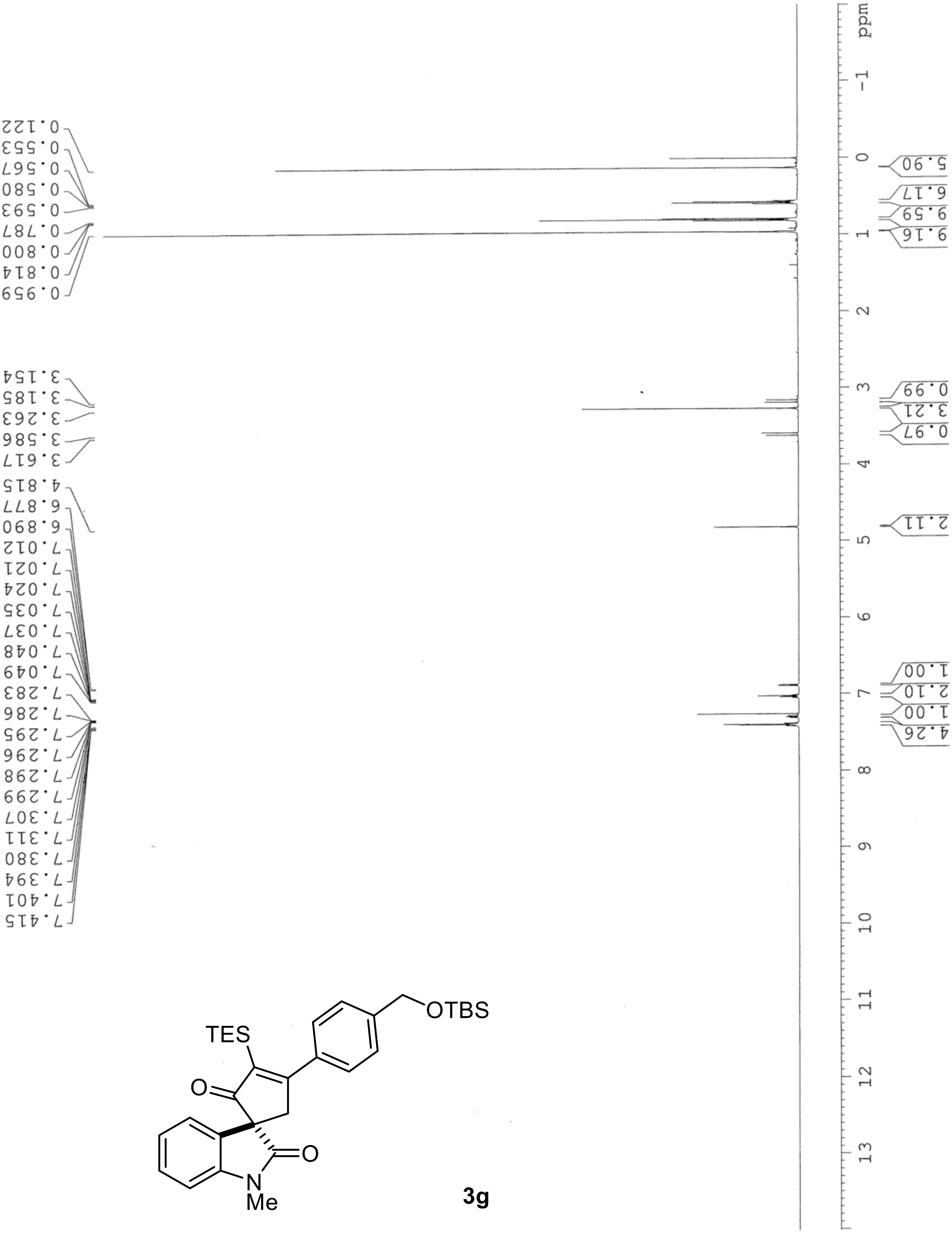
0.122
0.553
0.567
0.580
0.593
0.787
0.800
0.814
0.959
3.154
3.185
3.263
3.586
3.617
4.815
6.877
6.890
7.012
7.021
7.024
7.035
7.037
7.048
7.049
7.283
7.286
7.295
7.296
7.298
7.299
7.307
7.311
7.380
7.394
7.401
7.415
5.90
6.17
9.59
9.16
0.99
3.21
0.97
2.11
1.00
2.10
1.00
4.26
ppm
TES
OTBS
O
O
N
Me

3g

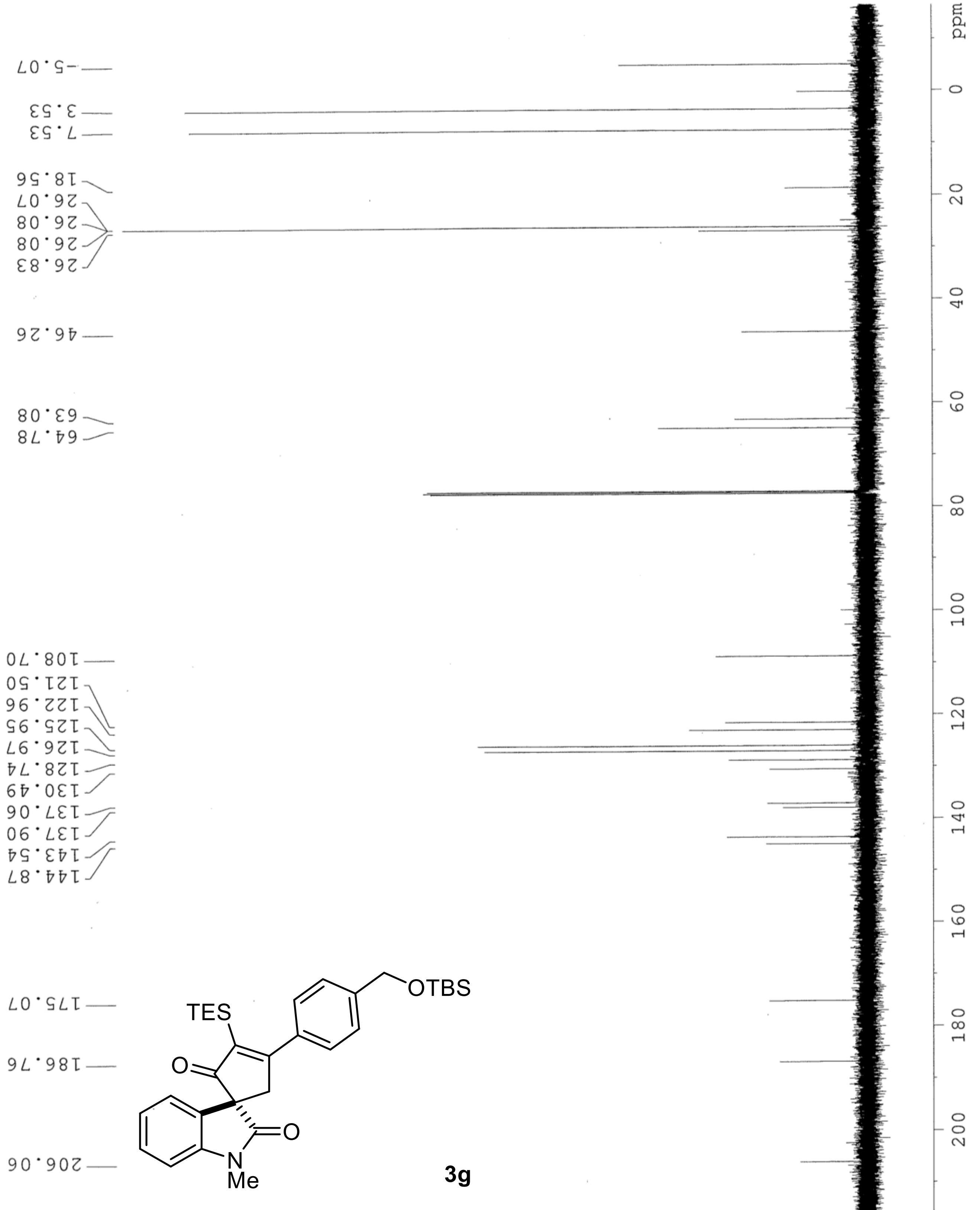
-5.07
3.53
7.53
18.56
26.07
26.08
26.08
26.83
46.26
63.08
64.78
108.70
121.50
122.96
125.95
126.97
128.74
130.49
137.06
137.90
143.54
144.87
175.07
186.76
206.06
ppm
TES
OTBS
O
N
Me

3g

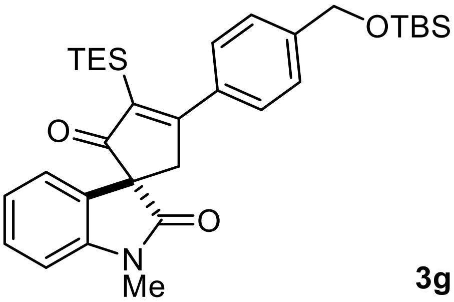

3g

Racemic

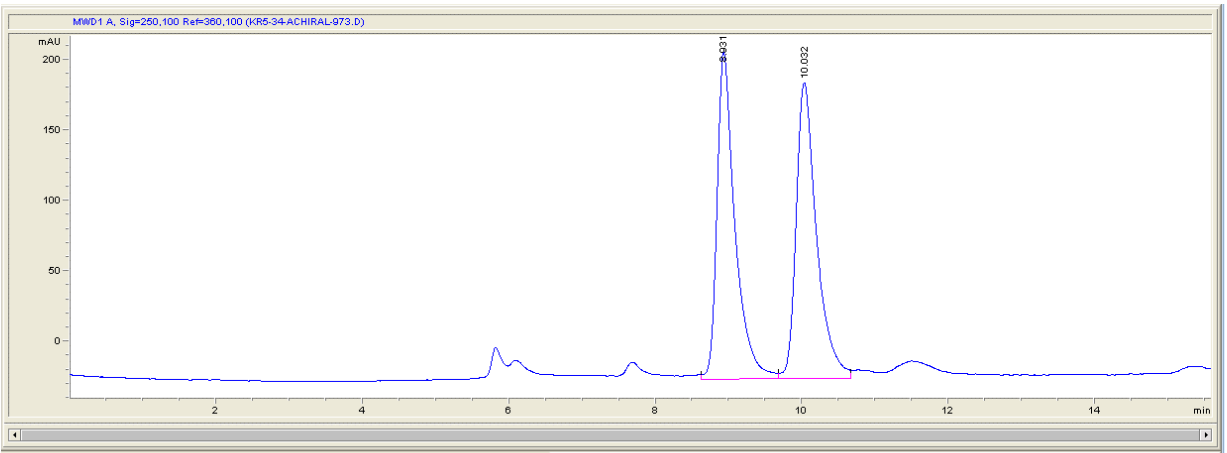

File Information

LC-File	KR5-34-ACHIRAL-973.D
File Path	C:\CHEM32\2\DATA\ERIN\
Date	09-Dec-16, 14:45:49

#	Time	Area	Height	Width	Area%	Symmetry
1	8.931	4022.8	232.5	0.2518	49.713	0.554
2	10.032	4069.3	210.4	0.2876	50.287	0.599

Rh_2(*S*-TCPTTL)$_4$: 84% ee

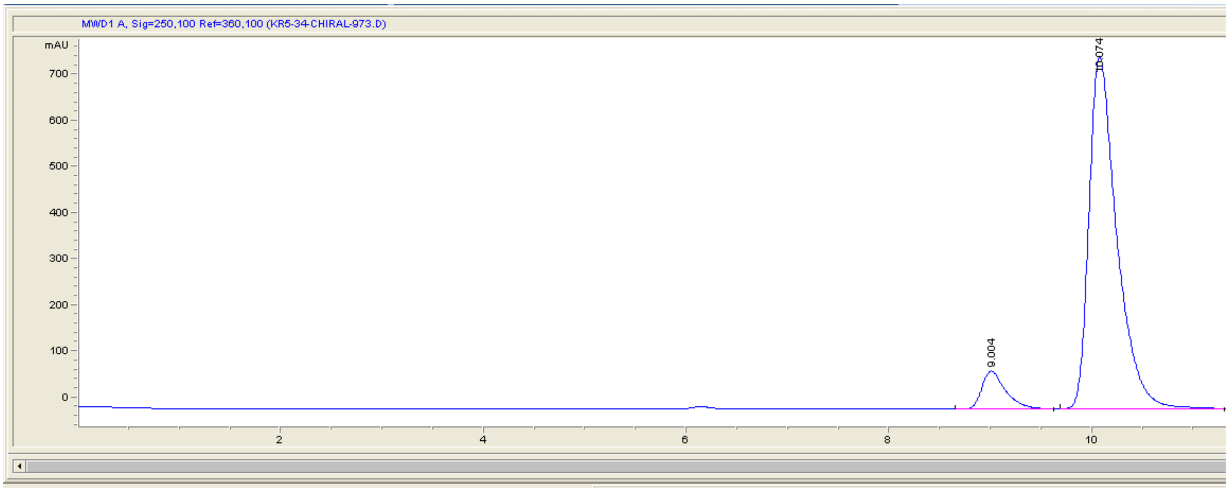

File Information

LC-File	KR5-34-CHIRAL-973.D

#	Time	Area	Height	Width	Area%	Symmetry
1	9.004	1346.3	82.6	0.2401	8.421	0.593
2	10.074	14640.8	765.6	0.2851	91.579	0.569

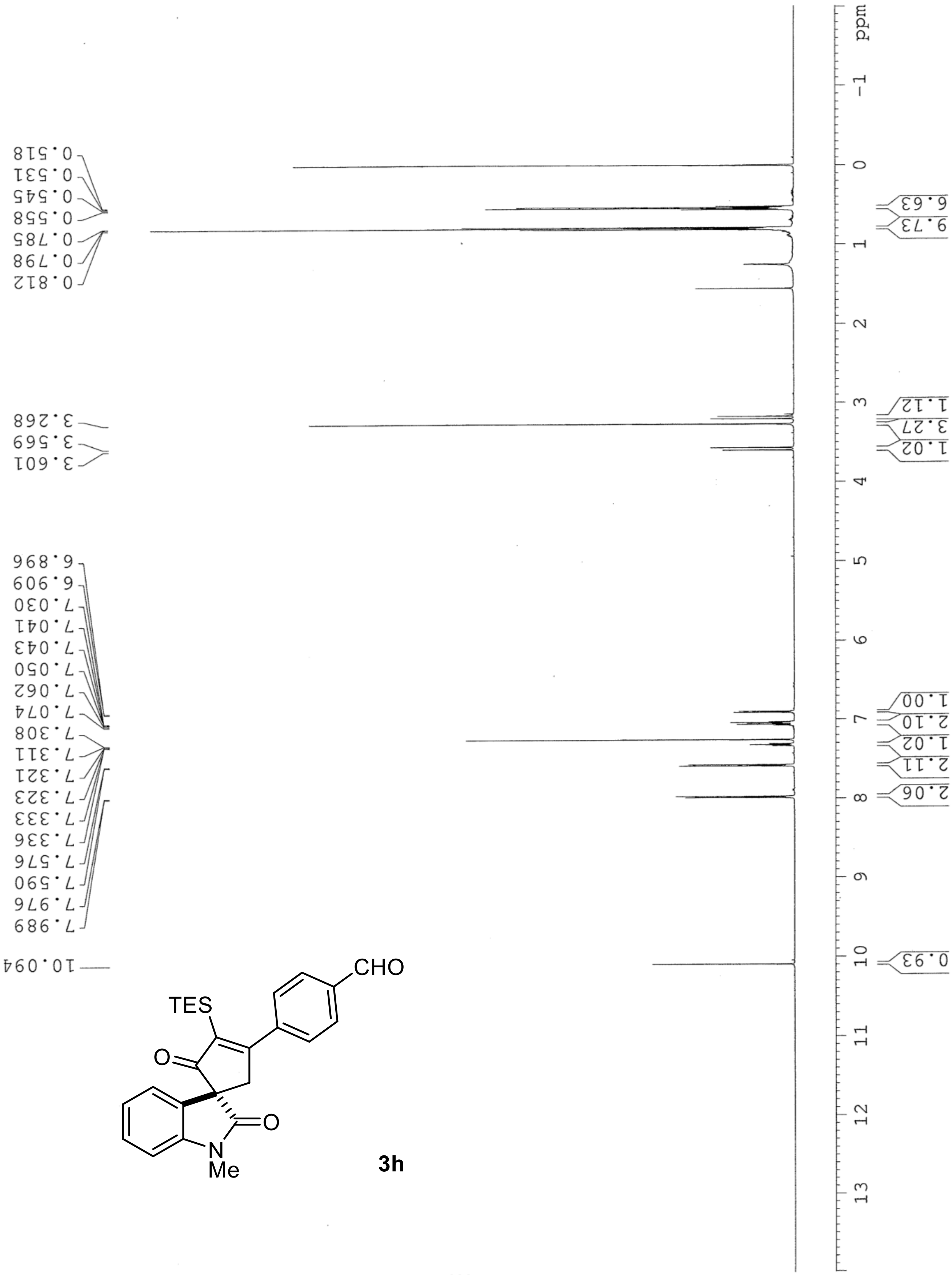

0.518
0.531
0.545
0.558
0.785
0.798
0.812
3.268
3.569
3.601
6.896
6.909
7.030
7.041
7.043
7.050
7.062
7.074
7.308
7.311
7.321
7.323
7.333
7.336
7.576
7.590
7.976
7.989
10.094
6.63
9.73
1.12
3.27
1.02
1.00
2.10
1.02
2.11
2.06
0.93
ppm
TES
CHO
O
N
Me

3h

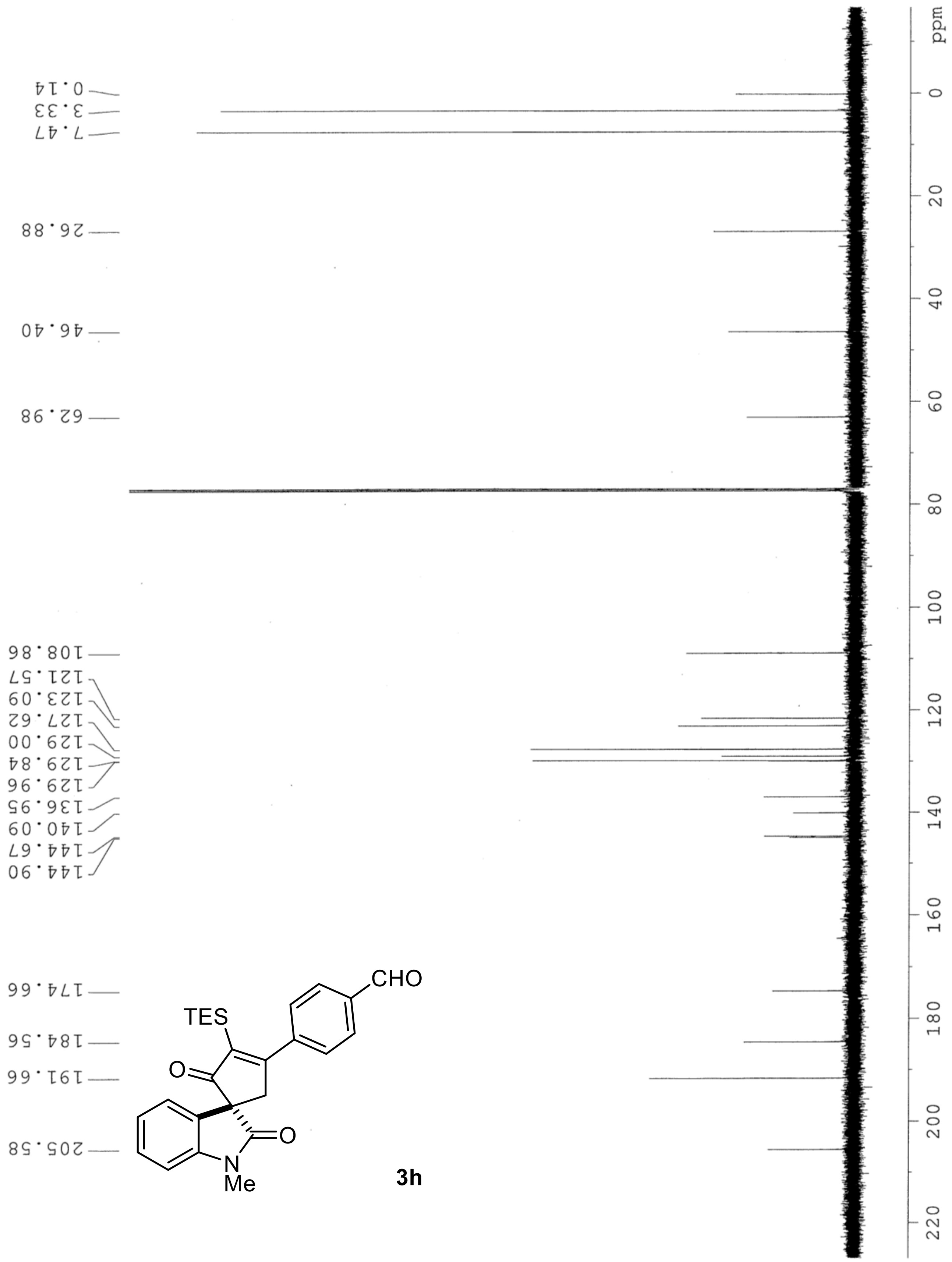
0.14
3.33
7.47
26.88
46.40
62.98
108.86
121.57
123.09
127.62
129.00
129.84
129.96
136.95
140.09
144.67
144.90
174.66
184.56
191.66
205.58
ppm
TES
CHO
O
N
Me

3h

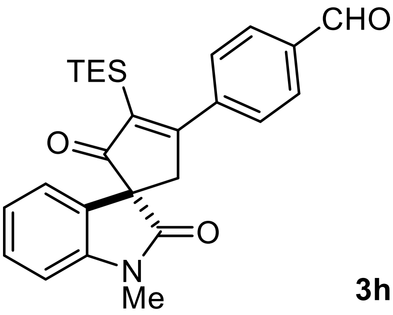

3h

Racemic

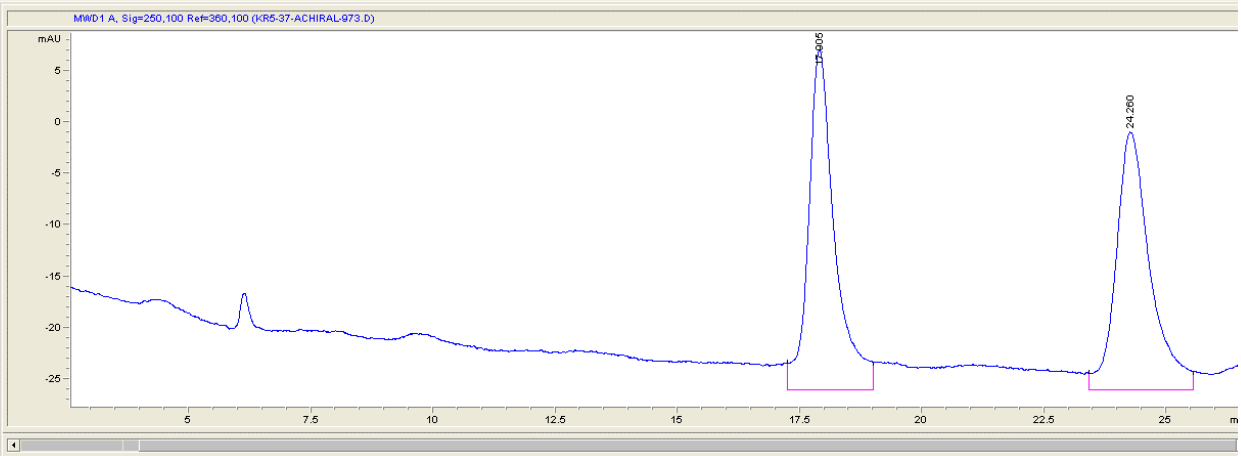

File Information

LC-File | KR5-37-ACHIRAL-973.D

#	Time	Area	Height	Width	Area%	Symmetry
1	17.905	1241.4	33.1	0.5252	50.518	0.668
2	24.26	1215.9	25.1	0.6356	49.482	0.677

Rh_2(*S*-TCPTTL)$_4$: 80% ee

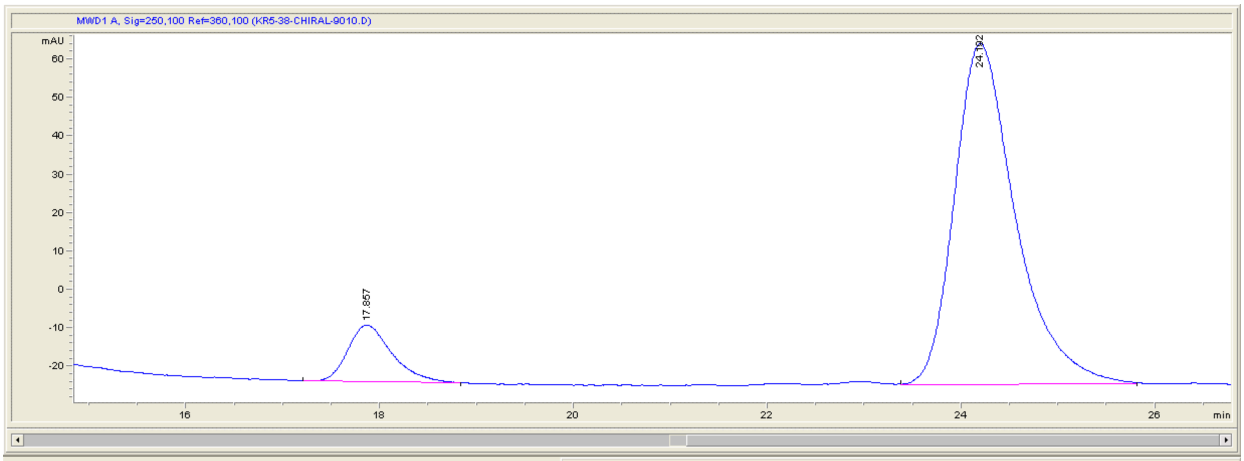

File Information

LC-File | KR5-38-CHIRAL-9010.D

#	Time	Area	Height	Width	Area%	Symmetry
1	17.857	469.5	14.8	0.4476	10.693	0.686
2	24.192	3920.8	89	0.6562	89.307	0.673

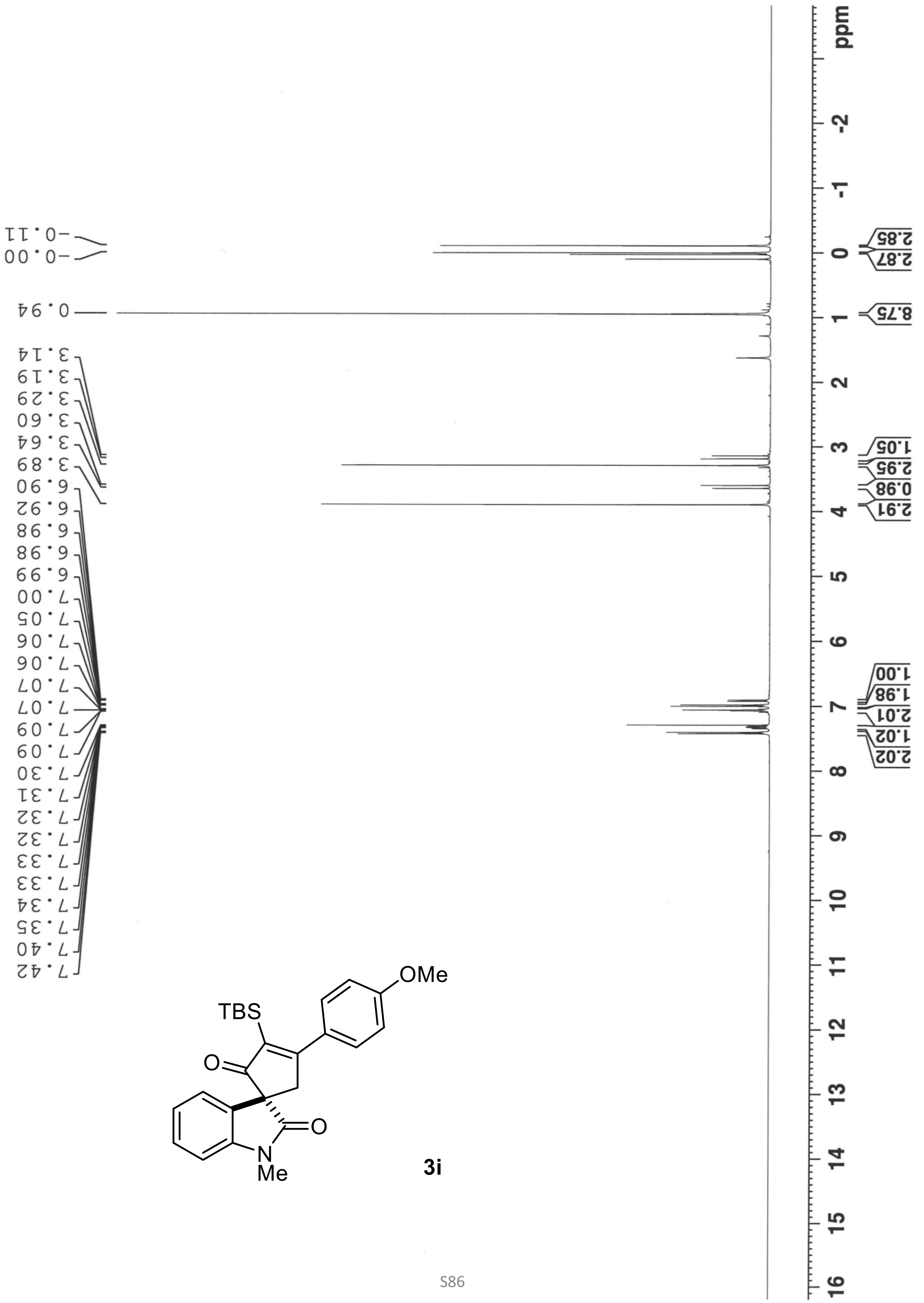

3i

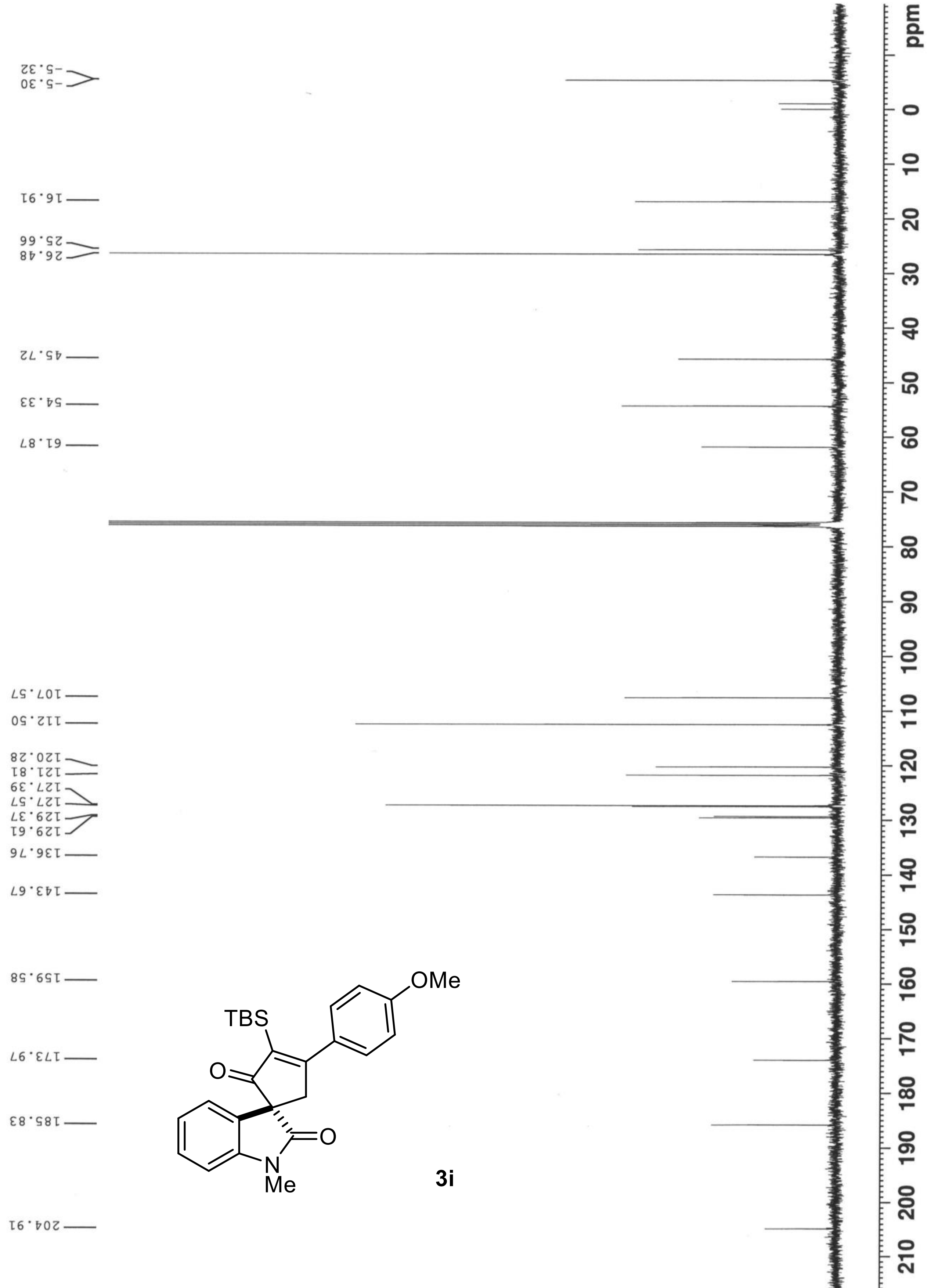
-5.30
-5.32
16.91
25.66
26.48
45.72
54.33
61.87
107.57
112.50
120.28
121.81
127.39
127.57
129.37
129.61
136.76
143.67
159.58
173.97
185.83
204.91
TBS
OMe
O
O
N
Me
ppm

3i

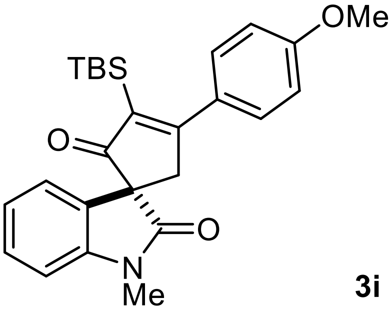

3i

Racemic

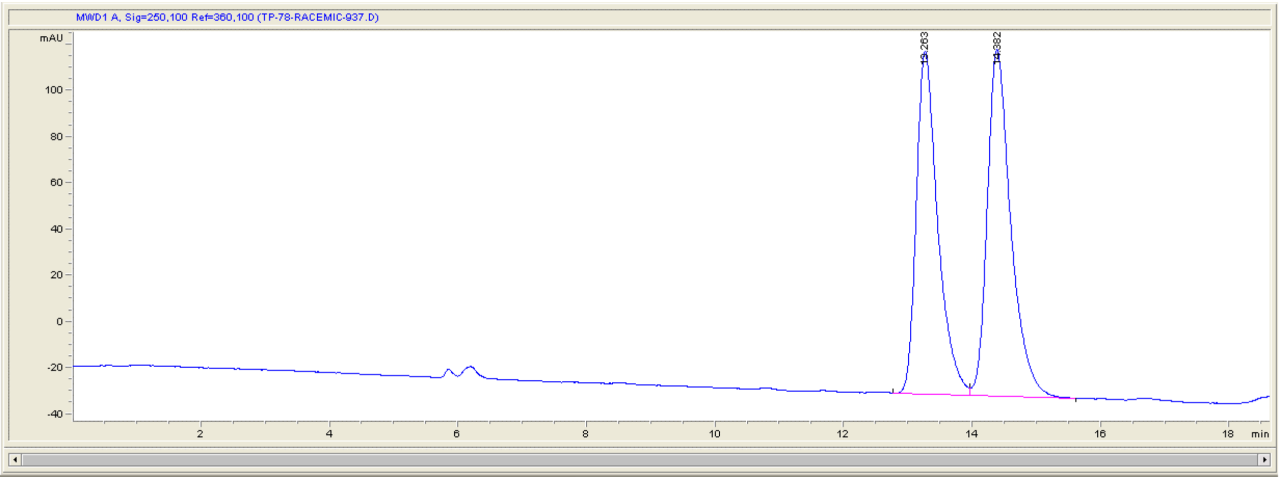

File Information

LC-File	TP-78-RACEMIC-937.D
File Path	C:\CHEM32\2\DATA\ERIN\

#	Time	Area	Height	Width	Area%	Symmetry
1	13.263	3458.1	148.4	0.3459	47.295	0.639
2	14.382	3853.7	150.4	0.381	52.705	0.656

Rh_2(*S*-TCPTTL)$_4$: 75% ee

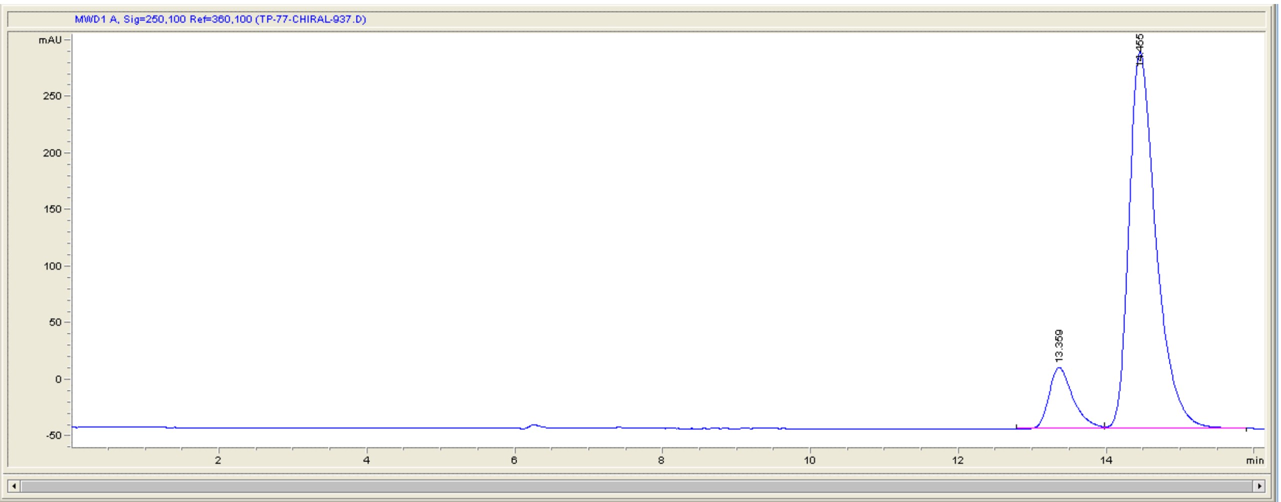

File Information

LC-File	TP-77-CHIRAL-937.D

#	Time	Area	Height	Width	Area%	Symmetry
1	13.359	1230.1	54.3	0.3361	12.625	0.669
2	14.455	8513.7	332.6	0.3867	87.375	0.639

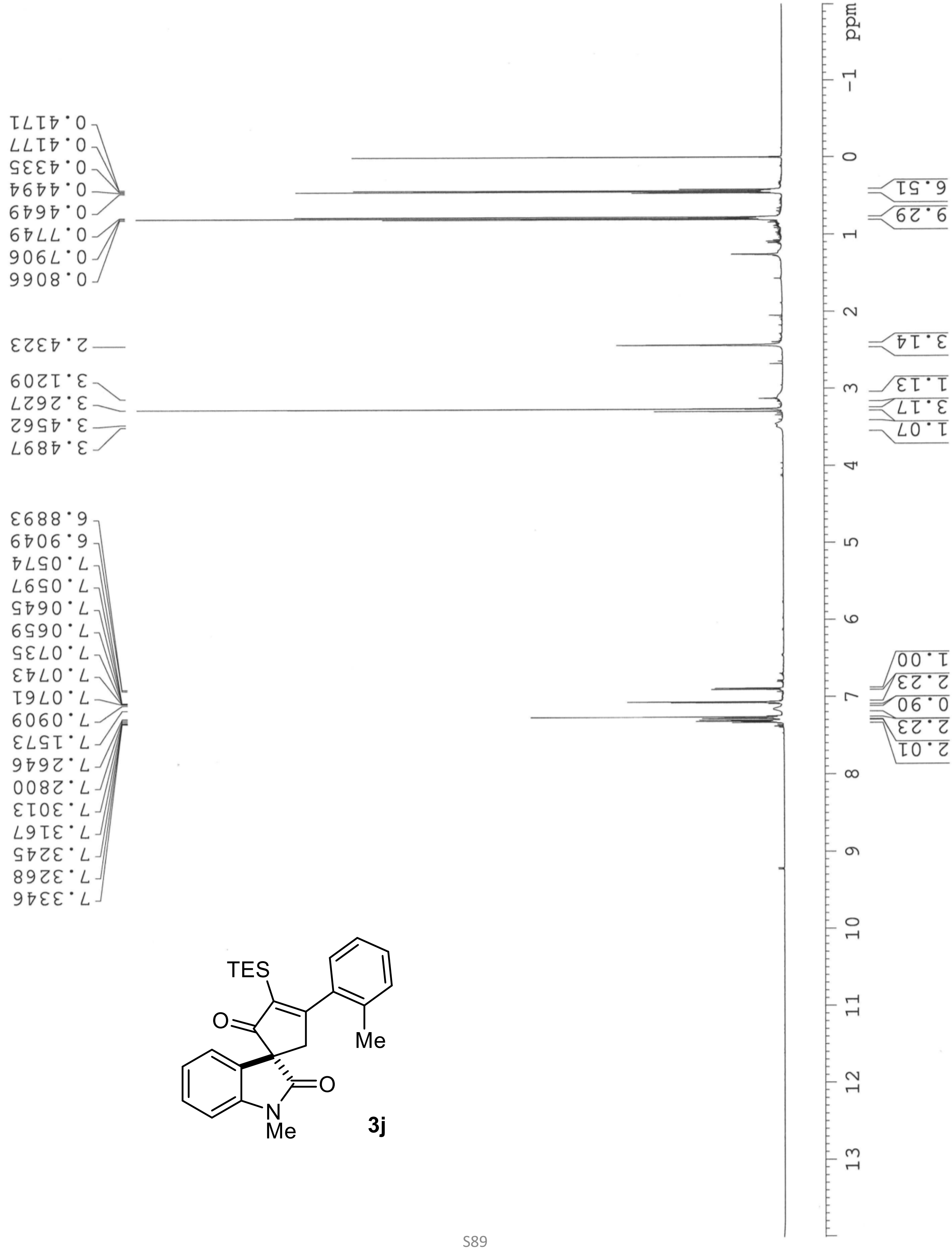
0.4171
0.4177
0.4335
0.4494
0.4649
0.7749
0.7906
0.8066
2.4323
3.1209
3.2627
3.4562
3.4897
6.8893
6.9049
7.0574
7.0597
7.0645
7.0659
7.0735
7.0743
7.0761
7.0909
7.1573
7.2646
7.2800
7.3013
7.3167
7.3245
7.3268
7.3346
6.51
9.29
3.14
1.13
3.17
1.07
1.00
2.23
0.90
2.23
2.01
ppm
TES
O
Me
O
N
Me

3j

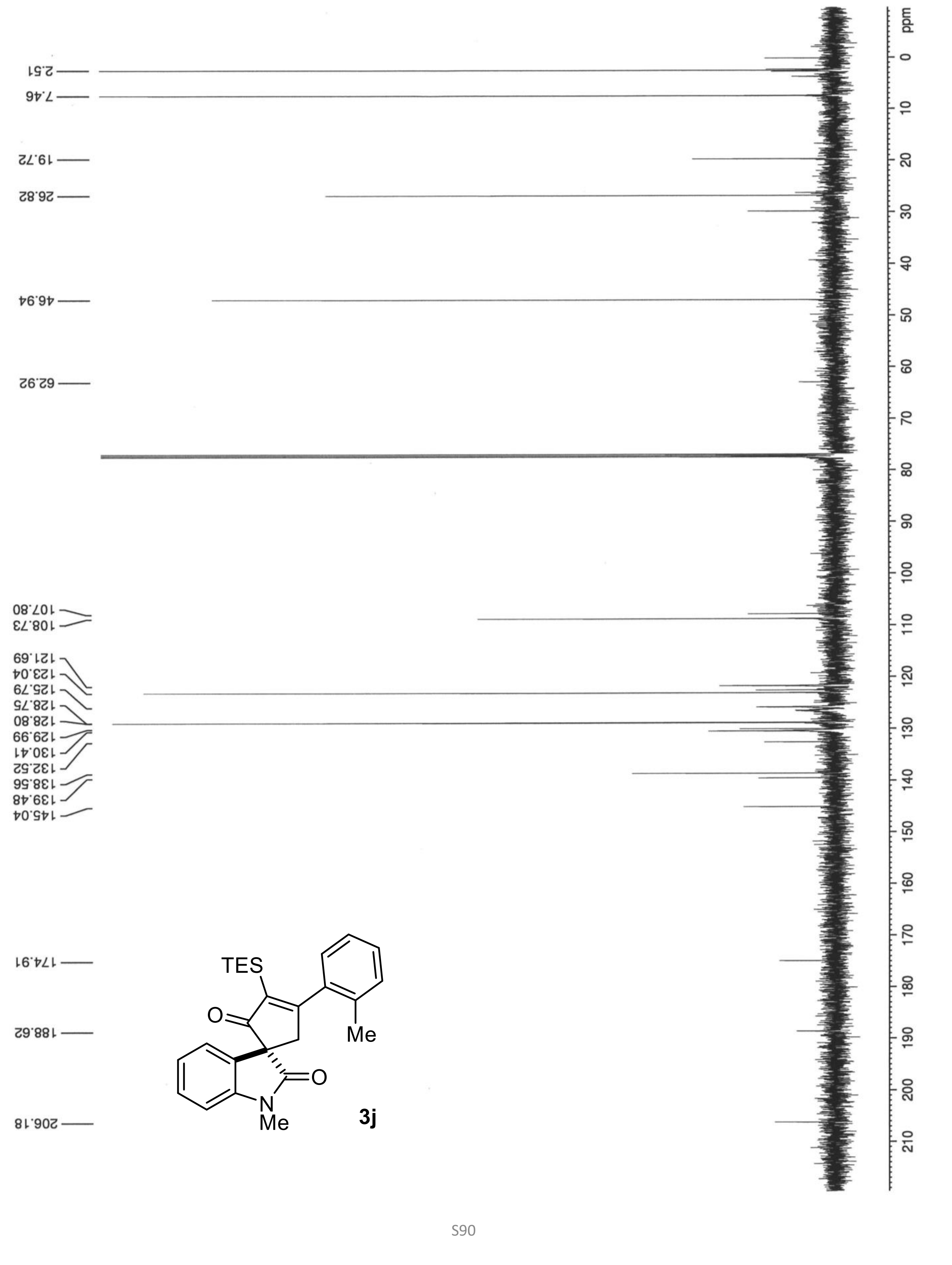
2.51
7.46
19.72
26.82
46.94
62.92
107.80
108.73
121.69
123.04
125.79
128.75
128.80
129.99
130.41
132.52
138.56
139.48
145.04
174.91
188.62
206.18
ppm
TES
O
Me
N
Me
3j

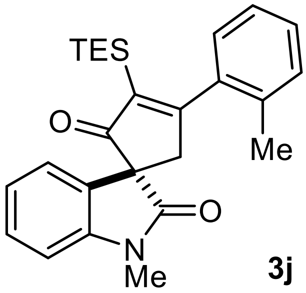

3j

Racemic

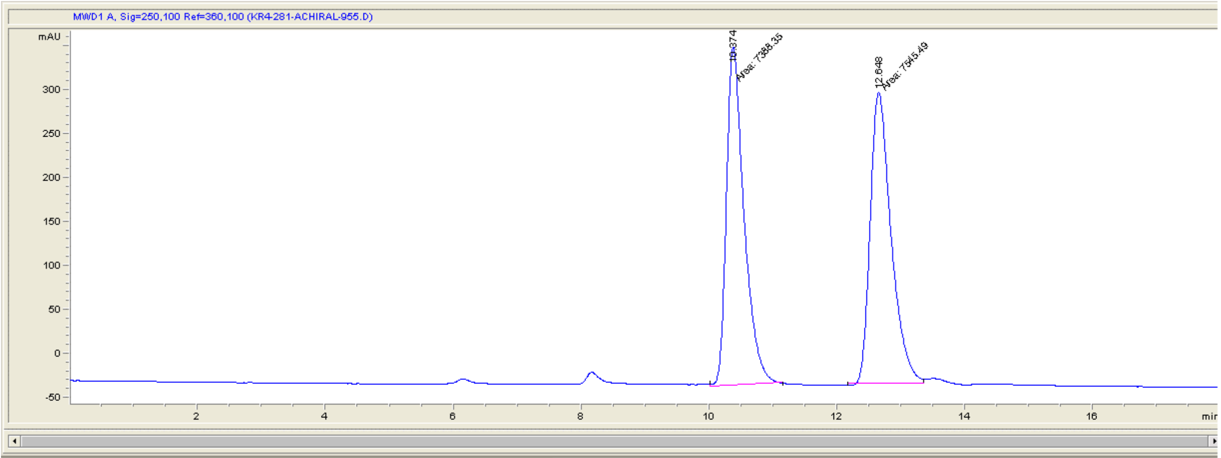

File Information

LC-File | KR4-281-ACHIRAL-955.D

#	Time	Area	Height	Width	Area%	Symmetry
1	10.374	7388.4	384	0.3207	49.474	0.612
2	12.648	7545.5	331.2	0.3797	50.526	0.649

Rh_2(*S*-TCPTTL)$_4$: 64% ee

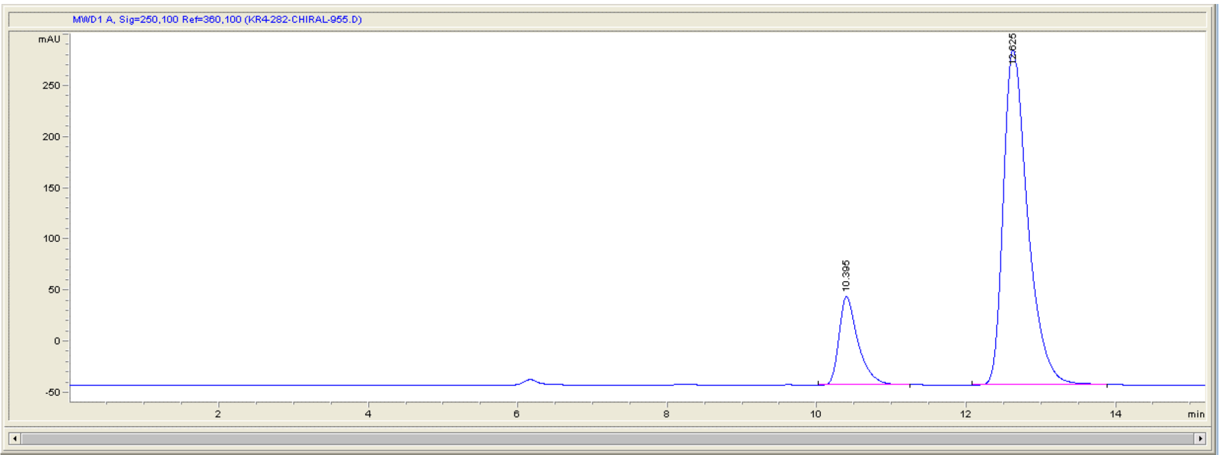

File Information

LC-File | KR4-282-CHIRAL-955.D

#	Time	Area	Height	Width	Area%	Symmetry
1	10.395	1572.6	86.8	0.2694	17.444	0.601
2	12.625	7442.7	327.8	0.3389	82.556	0.627

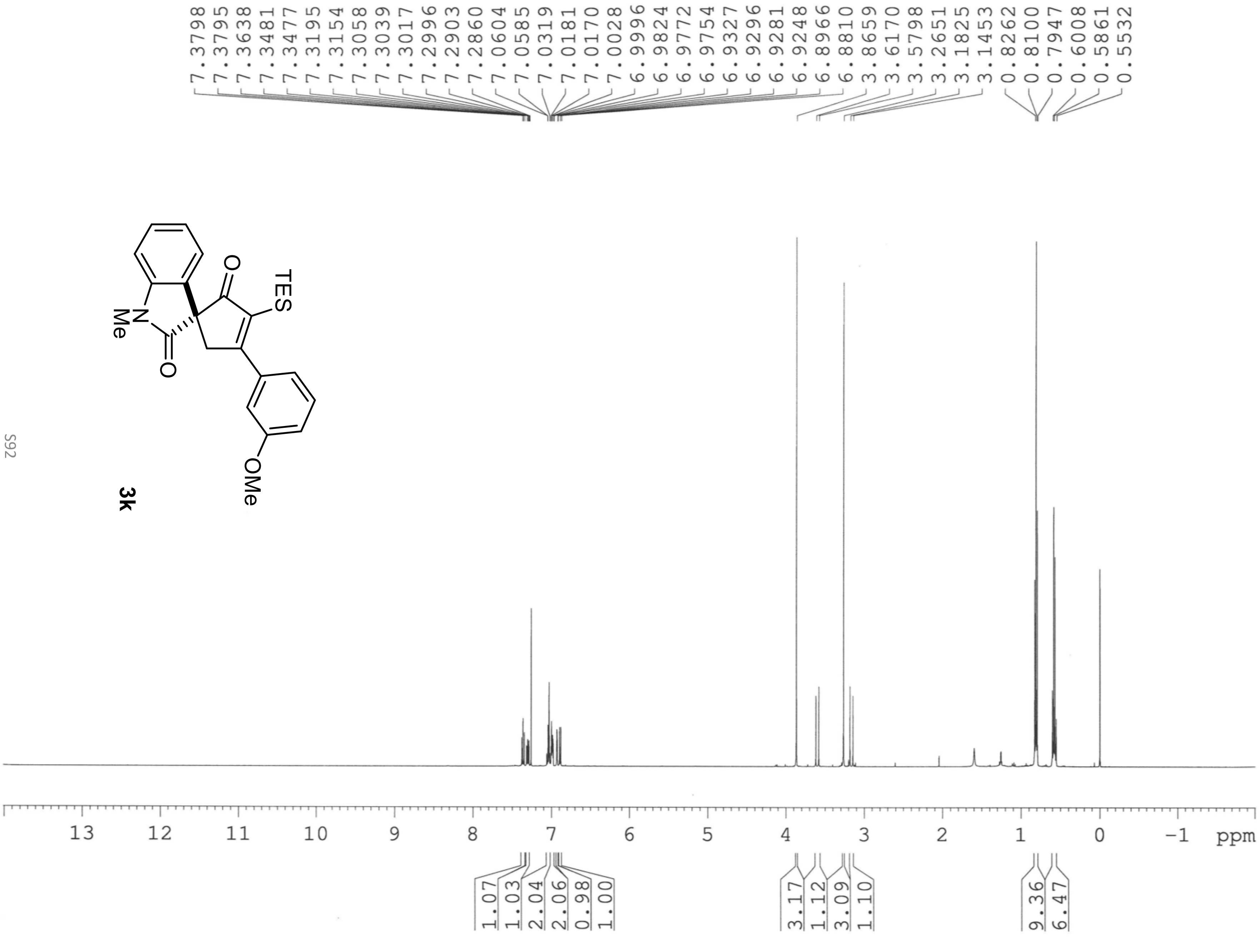
7.3798
7.3795
7.3638
7.3481
7.3477
7.3195
7.3154
7.3058
7.3039
7.3017
7.2996
7.2903
7.2860
7.0604
7.0585
7.0319
7.0181
7.0170
7.0028
6.9996
6.9824
6.9772
6.9754
6.9327
6.9296
6.9281
6.9248
6.8966
6.8810
3.8659
3.6170
3.5798
3.2651
3.1825
3.1453
0.8262
0.8100
0.7947
0.6008
0.5861
0.5532
TES
O
N
Me
O
OMe
13
12
11
10
9
8
7
6
5
4
3
2
1
0
−1
ppm
1.07
1.03
2.04
2.06
0.98
1.00
3.17
1.12
3.09
1.10
9.36
6.47

3k

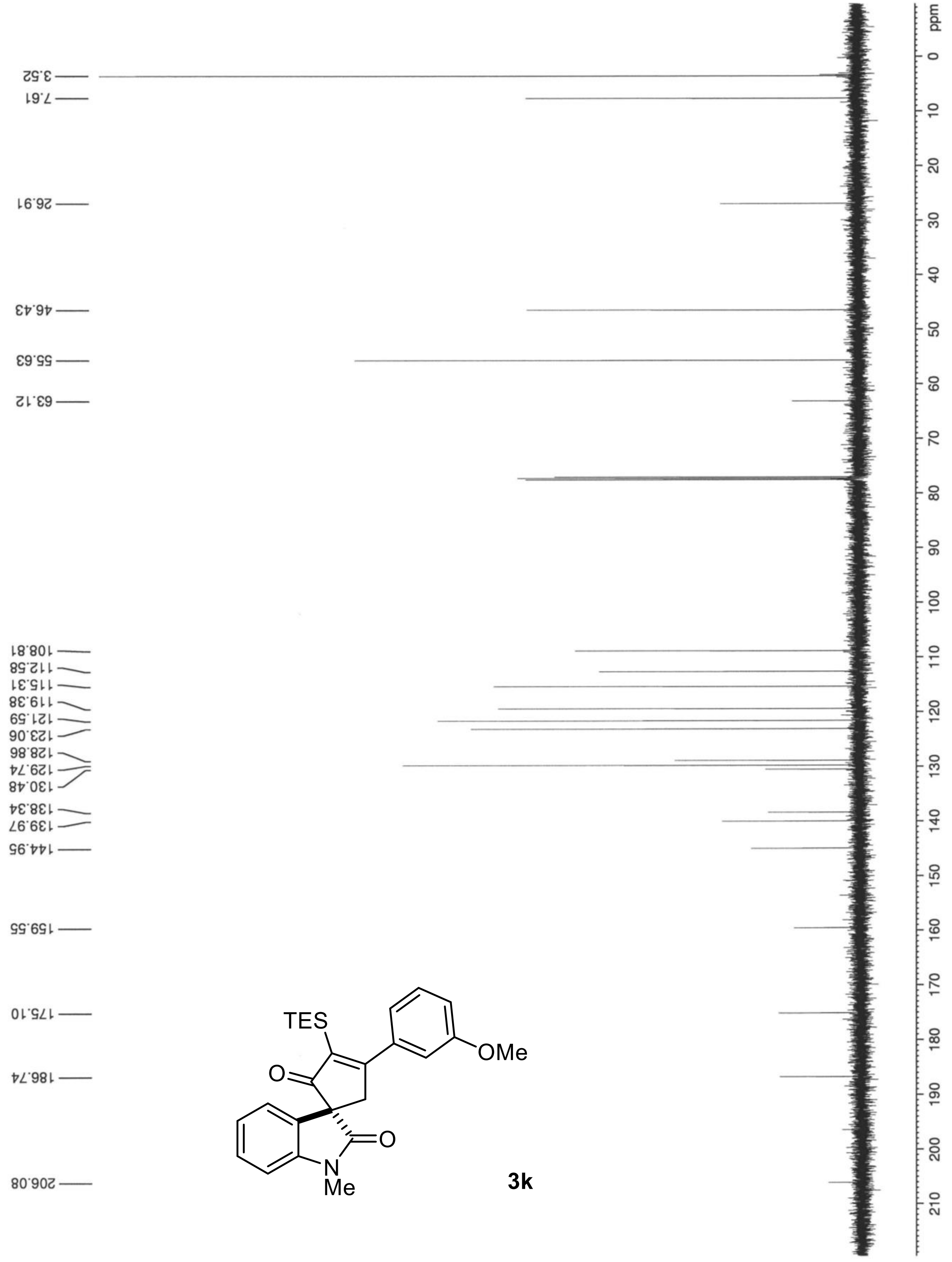
3.52
7.61
26.91
46.43
55.63
63.12
108.81
112.58
115.31
119.38
121.59
123.06
128.86
129.74
130.48
138.34
139.97
144.95
159.55
175.10
186.74
206.08
ppm
0
10
20
30
40
50
60
70
80
90
100
110
120
130
140
150
160
170
180
190
200
210
TES
OMe
O
O
N
Me

3k

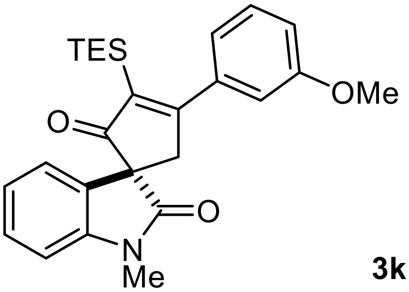

3k

Racemic

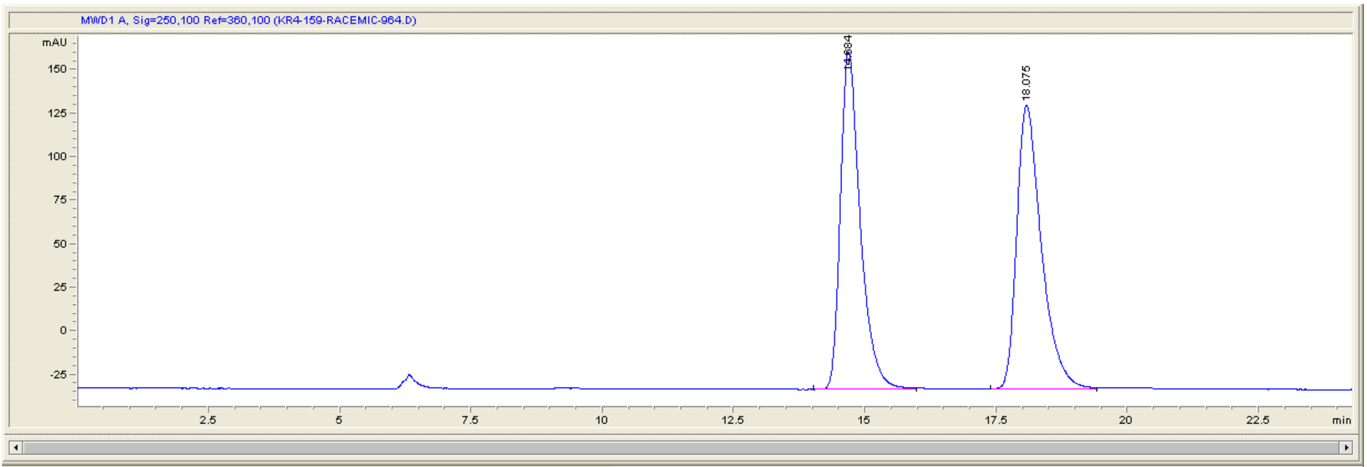

File Information

LC-File	KR4-159-RACEMIC-964.D

#	Time	Area	Height	Width	Area%	Symmetry
1	14.684	5117.3	193.6	0.3964	48.759	0.636
2	18.075	5377.9	163.1	0.4907	51.241	0.611

Rh_2(*S*-TCPTTL)$_4$: 88% ee

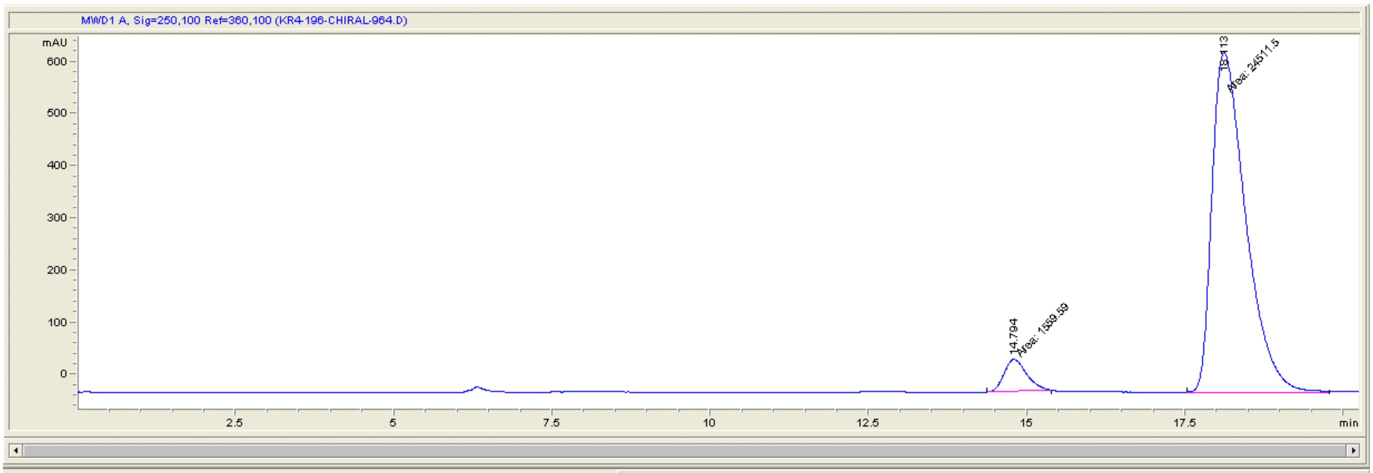

File Information

LC-File	KR4-196-CHIRAL-964.D

#	Time	Area	Height	Width	Area%	Symmetry
1	14.794	1559.6	62	0.4189	5.982	0.772
2	18.113	24511.5	654	0.6247	94.018	0.534

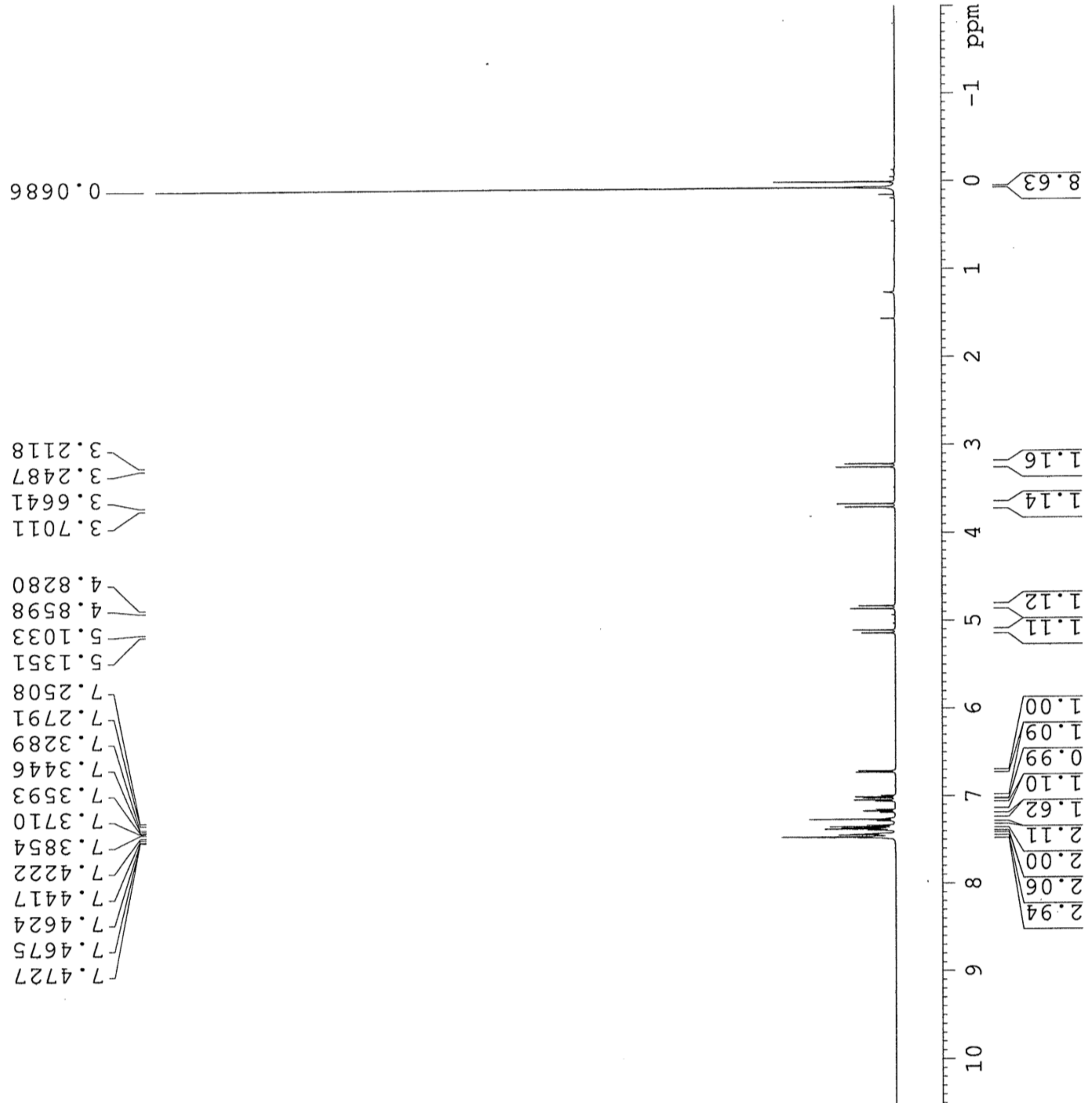
0.0686
3.2118
3.2487
3.6641
3.7011
4.8280
4.8598
5.1033
5.1351
7.2508
7.2791
7.3289
7.3446
7.3593
7.3710
7.3854
7.4222
7.4417
7.4624
7.4675
7.4727
8.63
1.16
1.14
1.12
1.11
1.00
1.09
0.99
1.10
1.62
2.11
2.00
2.06
2.94
ppm

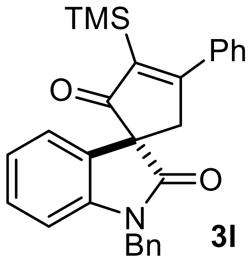
TMS
Ph
O
O
N
Bn

3l

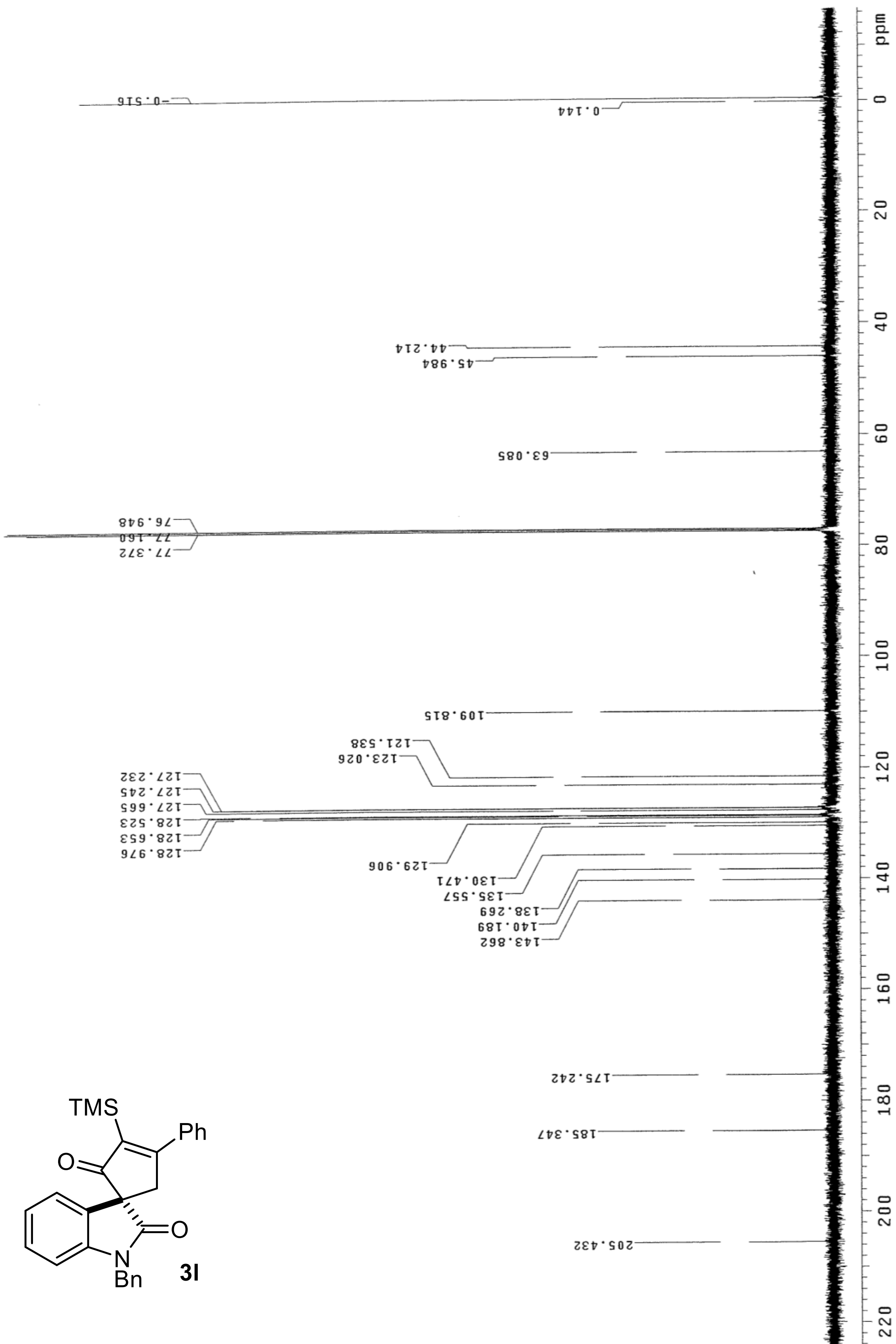
ppm
-0.516
0.144
44.214
45.984
63.085
76.948
77.160
77.372
109.815
121.538
123.026
127.232
127.245
127.665
128.523
128.653
128.976
129.906
130.471
135.557
138.269
140.189
143.862
175.242
185.347
205.432
TMS
Ph
O
O
N
Bn
3l

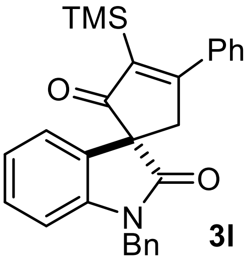

3l

Racemic

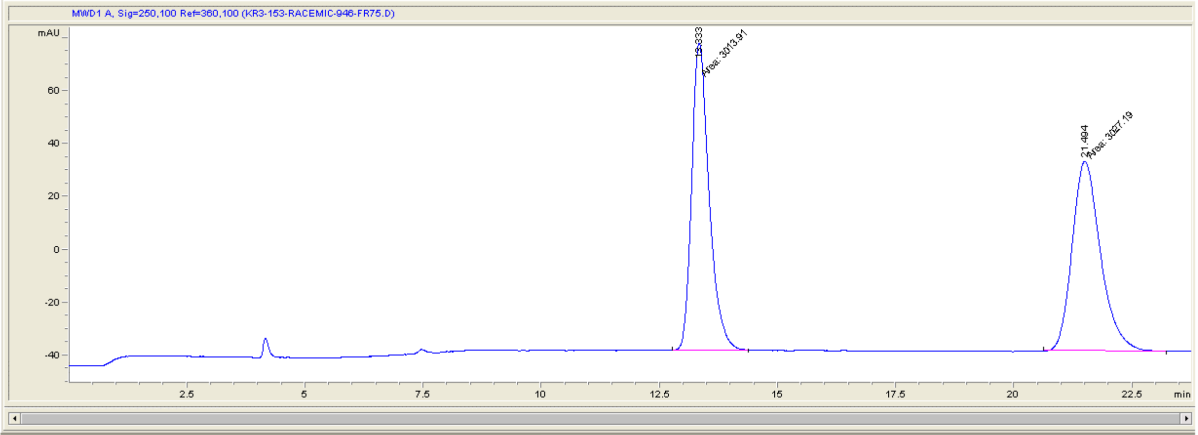

File Information

LC-File	KR3-153-RACEMIC-946-FR75.D
File Path	C:\CHEM32\2\DATA\ERIN\

#	Time	Area	Height	Width	Area%	Symmetry
1	13.333	3013.9	116.5	0.4312	49.890	0.719
2	21.494	3027.2	71.9	0.7015	50.110	0.769

Rh_2(*S*-TCPTTL)$_4$: 90% ee

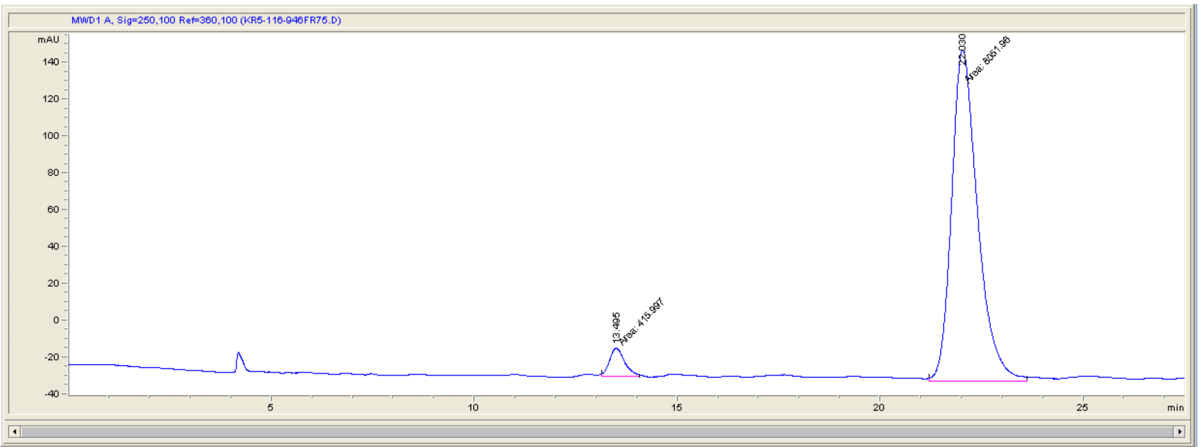

File Information

LC-File	KR5-116-946FR75.D
File Path	C:\CHEM32\2\DATA\ERIN\

#	Time	Area	Height	Width	Area%	Symmetry
1	13.495	416	15.6	0.4437	4.913	0.781
2	22.03	8052	179.9	0.7462	95.087	0.714

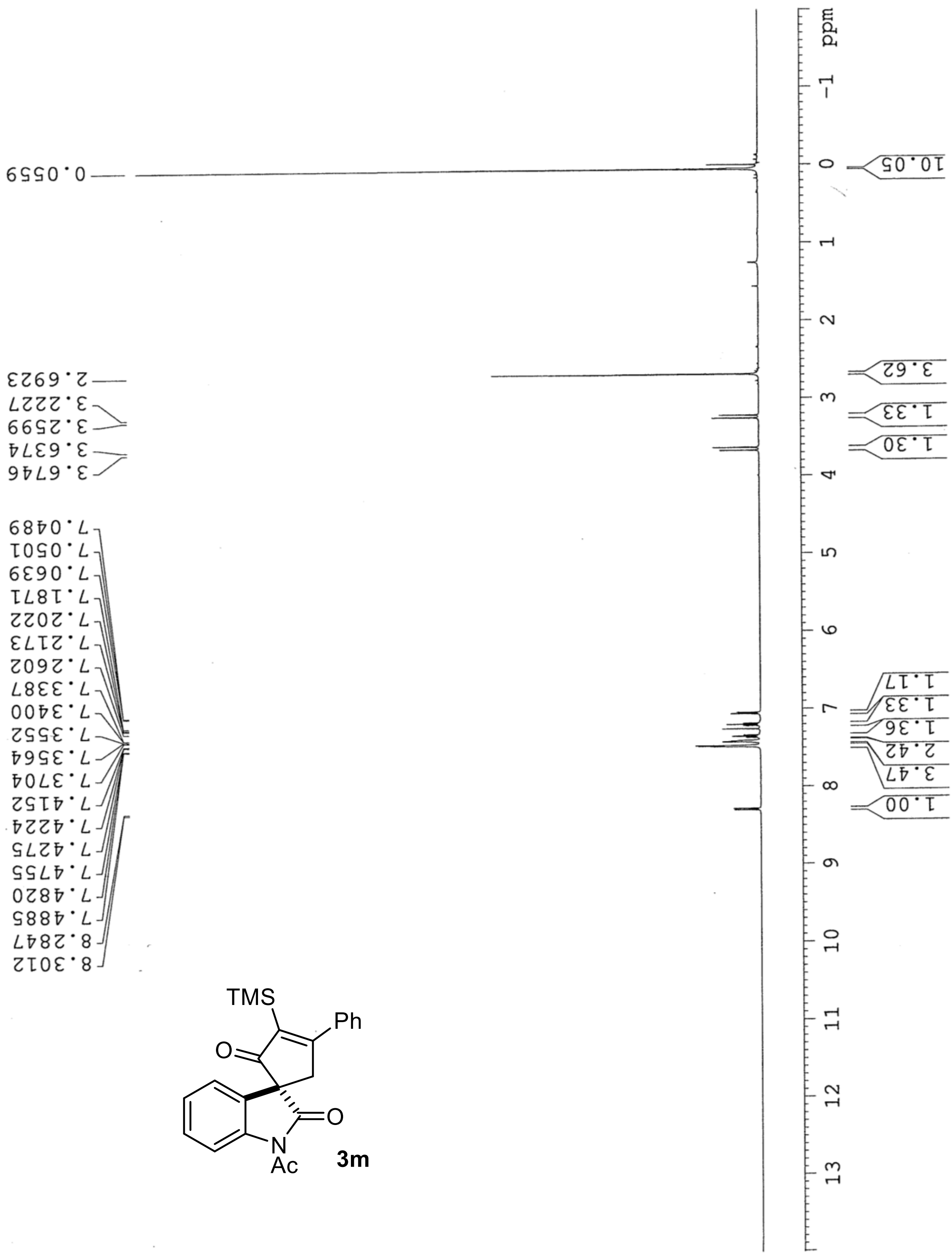
0.0559
2.6923
3.2227
3.2599
3.6374
3.6746
7.0489
7.0501
7.0639
7.1871
7.2022
7.2173
7.2602
7.3387
7.3400
7.3552
7.3564
7.3704
7.4152
7.4224
7.4275
7.4755
7.4820
7.4885
8.2847
8.3012
10.05
3.62
1.33
1.30
1.17
1.33
1.36
2.42
3.47
1.00
ppm
TMS
Ph
O
O
N
Ac
3m

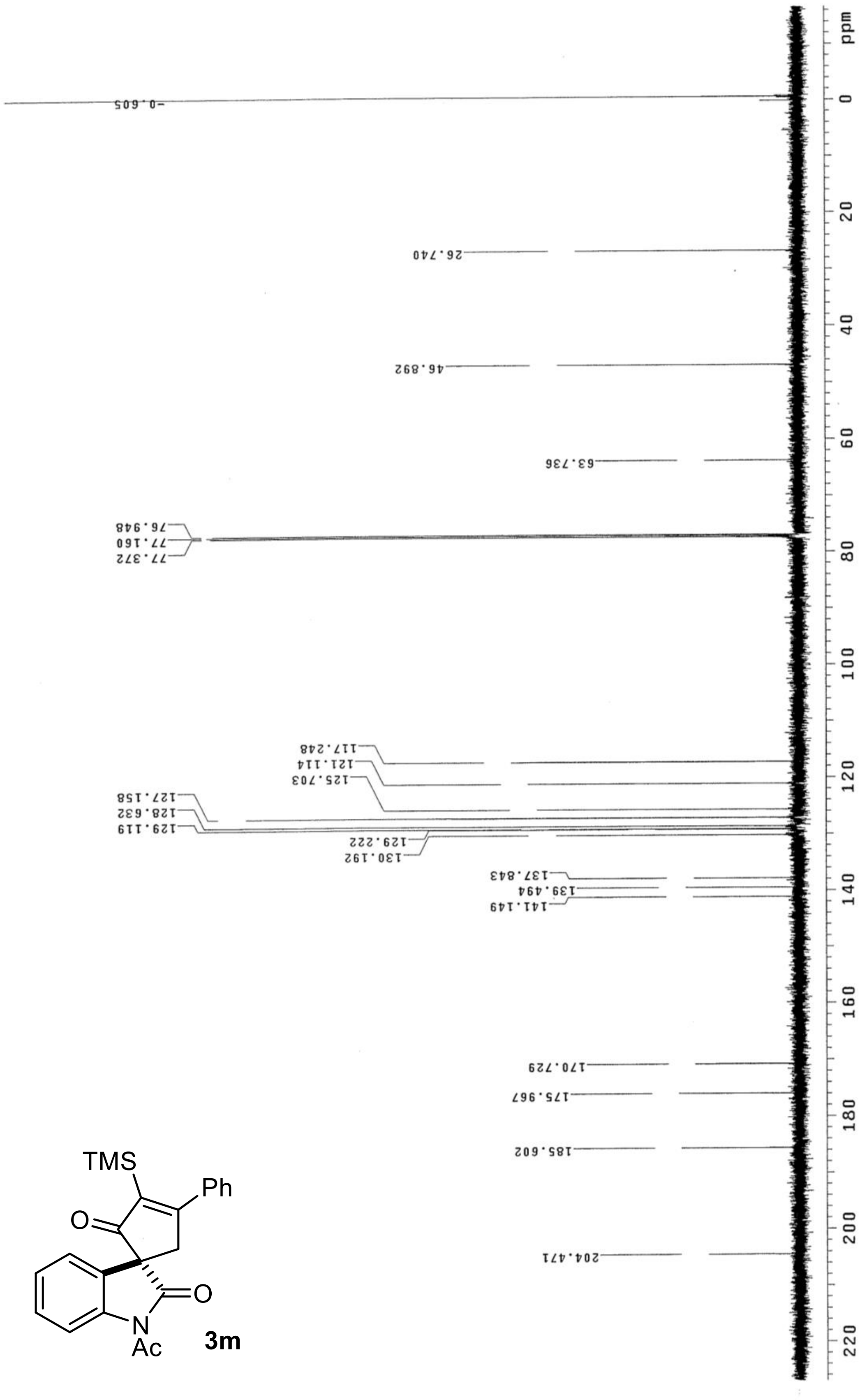

-0.605
26.740
46.892
63.736
76.948
77.160
77.372
117.248
121.114
125.703
127.158
128.632
129.119
129.222
130.192
137.843
139.494
141.149
170.729
175.967
185.602
204.471
ppm
0
20
40
60
80
100
120
140
160
180
200
220
TMS
Ph
O
O
N
Ac

3m

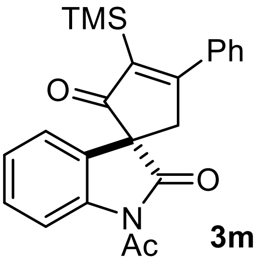

3m

Racemic

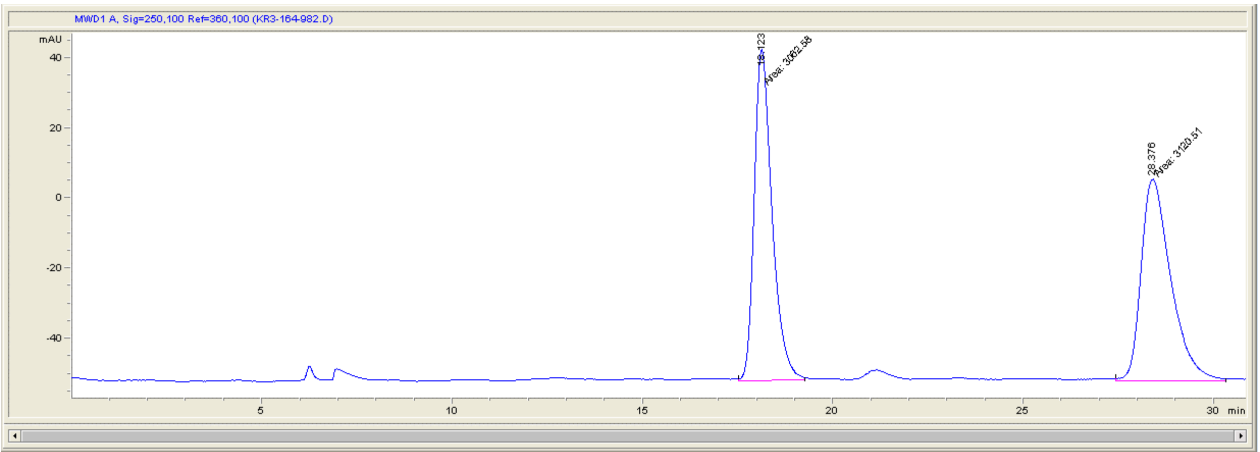

File Information

LC-File KR3-164-982.D

#	Time	Area	Height	Width	Area%	Symmetry
1	18.123	3062.6	94.3	0.5415	49.532	0.686
2	28.376	3120.5	57.4	0.9055	50.468	0.606

Rh_2(*S*-TCPTTL)$_4$: 79% ee

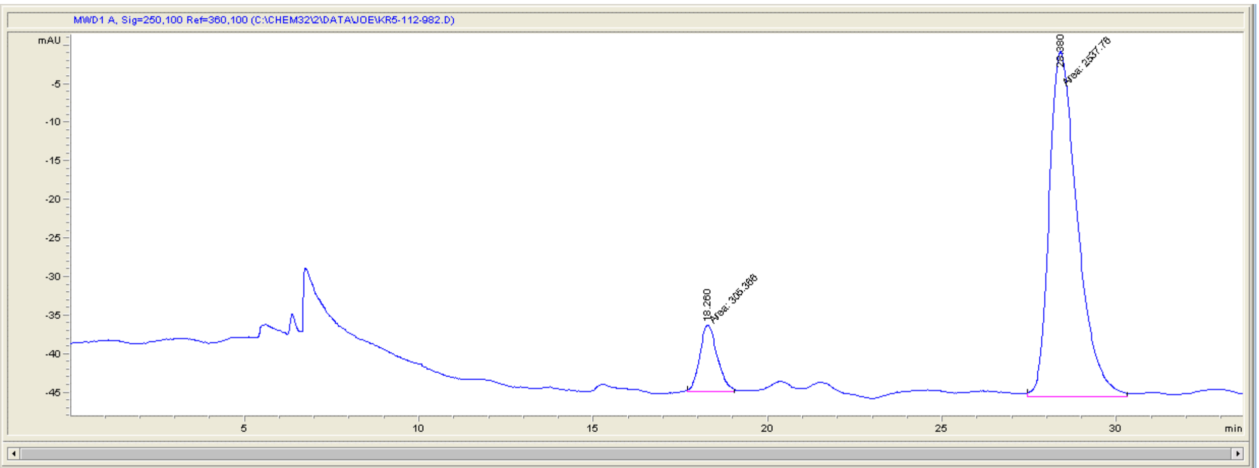

File Information

LC-File KR5-112-982.D

File Path C:\CHEM32\2\DATA\JOE\

#	Time	Area	Height	Width	Area%	Symmetry
1	18.26	305.4	8.6	0.5898	10.740	0.782
2	28.38	2537.8	44.8	0.9435	89.260	0.645

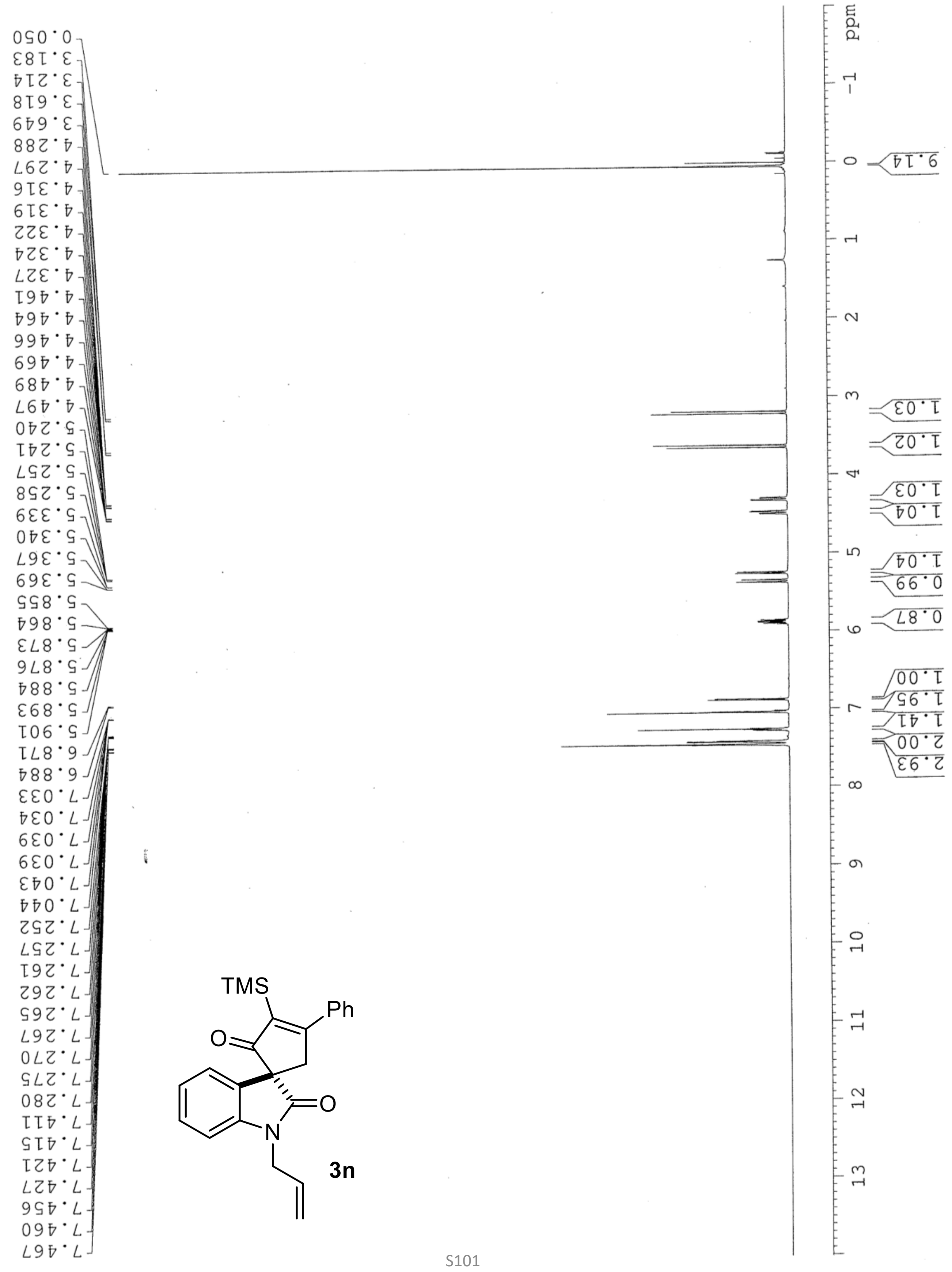
0.050
3.183
3.214
3.618
3.649
4.288
4.297
4.316
4.319
4.322
4.324
4.327
4.461
4.464
4.466
4.469
4.489
4.497
5.240
5.241
5.257
5.258
5.339
5.340
5.367
5.369
5.855
5.864
5.873
5.876
5.884
5.893
5.901
6.871
6.884
7.033
7.034
7.039
7.039
7.043
7.044
7.252
7.257
7.261
7.262
7.265
7.267
7.270
7.275
7.280
7.411
7.415
7.421
7.427
7.456
7.460
7.467
9.14
1.03
1.02
1.03
1.04
1.04
0.99
0.87
1.00
1.95
1.41
2.00
2.93
ppm
TMS
Ph
O
O
N

3n

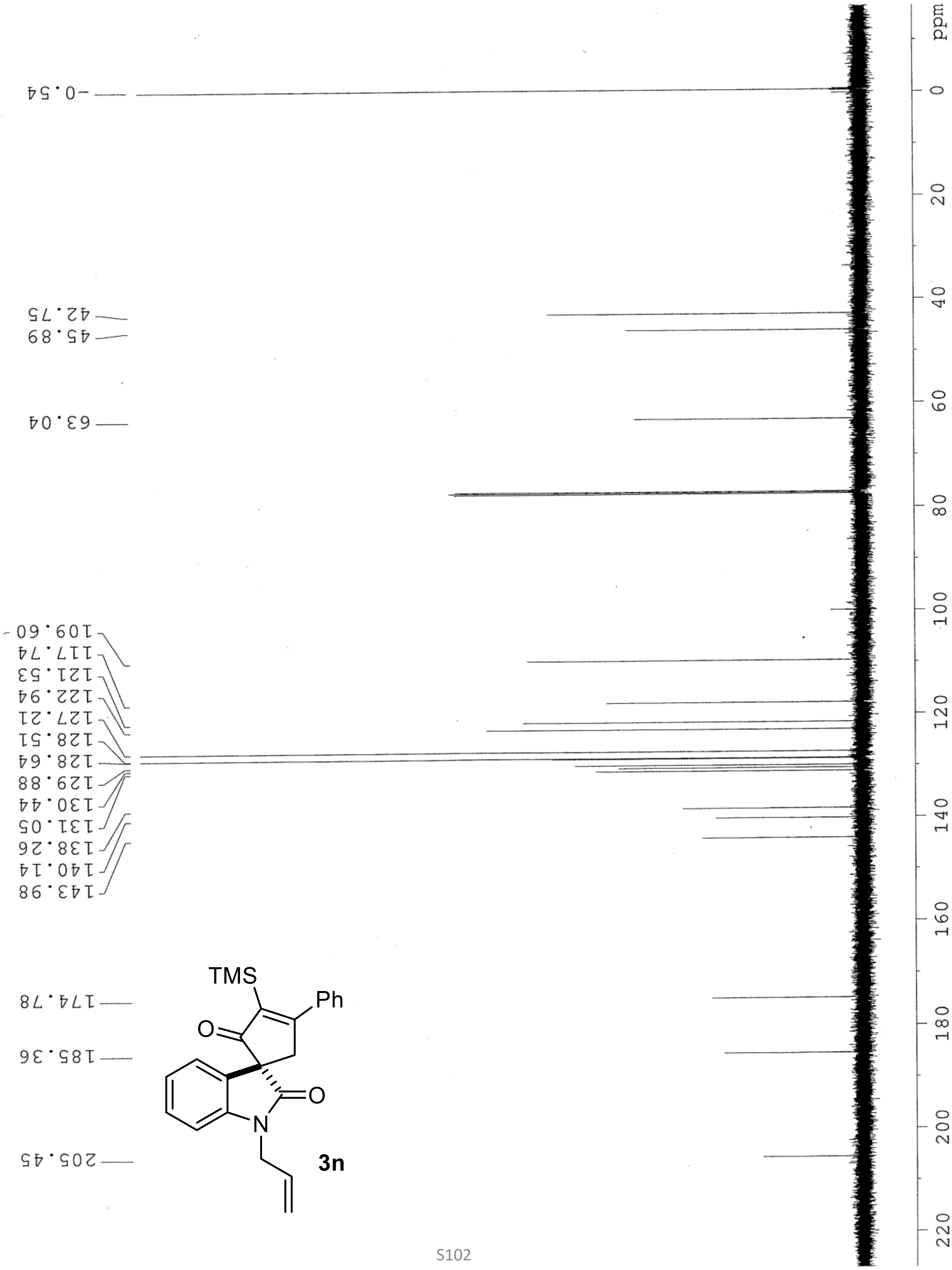
-0.54
42.75
45.89
63.04
109.60
117.74
121.53
122.94
127.21
128.51
128.64
129.88
130.44
131.05
138.26
140.14
143.98
174.78
185.36
205.45
ppm
0
20
40
60
80
100
120
140
160
180
200
220
TMS
Ph
O
O
N
3n

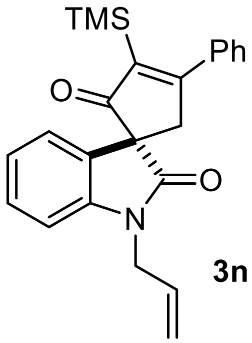

3n

Racemic

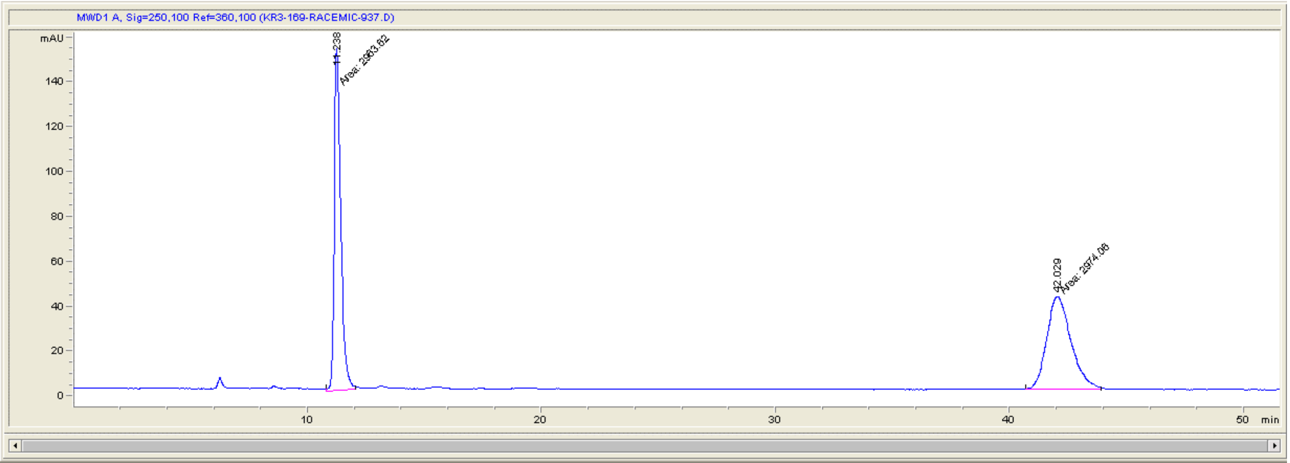

File Information

LC-File | KR3-169-RACEMIC-937.D

#	Time	Area	Height	Width	Area%	Symmetry
1	11.238	2963.6	152.2	0.3245	49.912	0.652
2	42.029	2974.1	41.2	1.2021	50.088	0.772

Rh_2(*S*-TCPTTL)$_4$: 88% ee

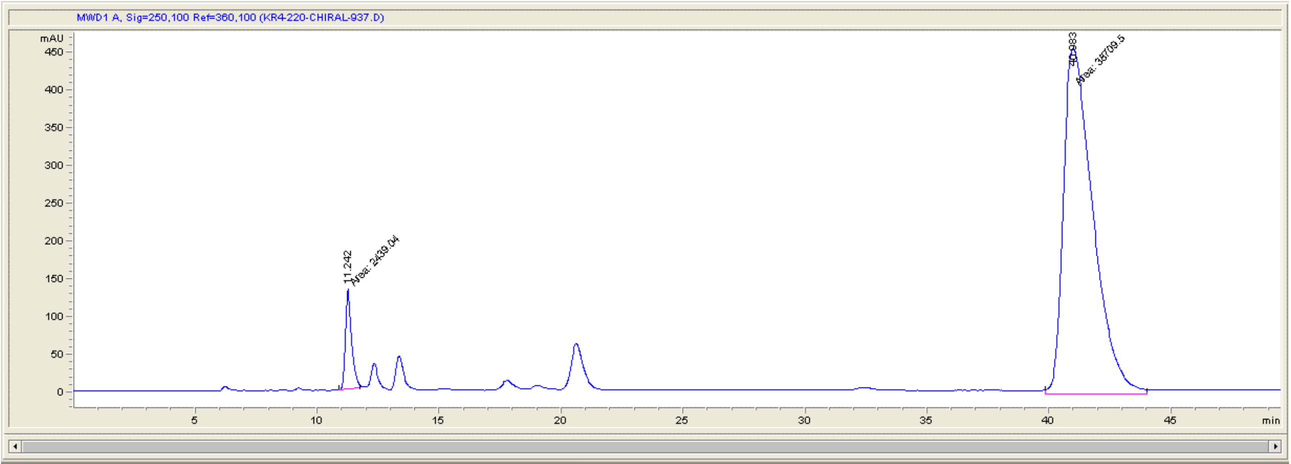

File Information

LC-File | KR4-220-CHIRAL-937.D

#	Time	Area	Height	Width	Area%	Symmetry
1	11.242	2439	132.9	0.3059	5.927	0.685
2	40.983	38709.5	456.7	1.4125	94.073	0.484

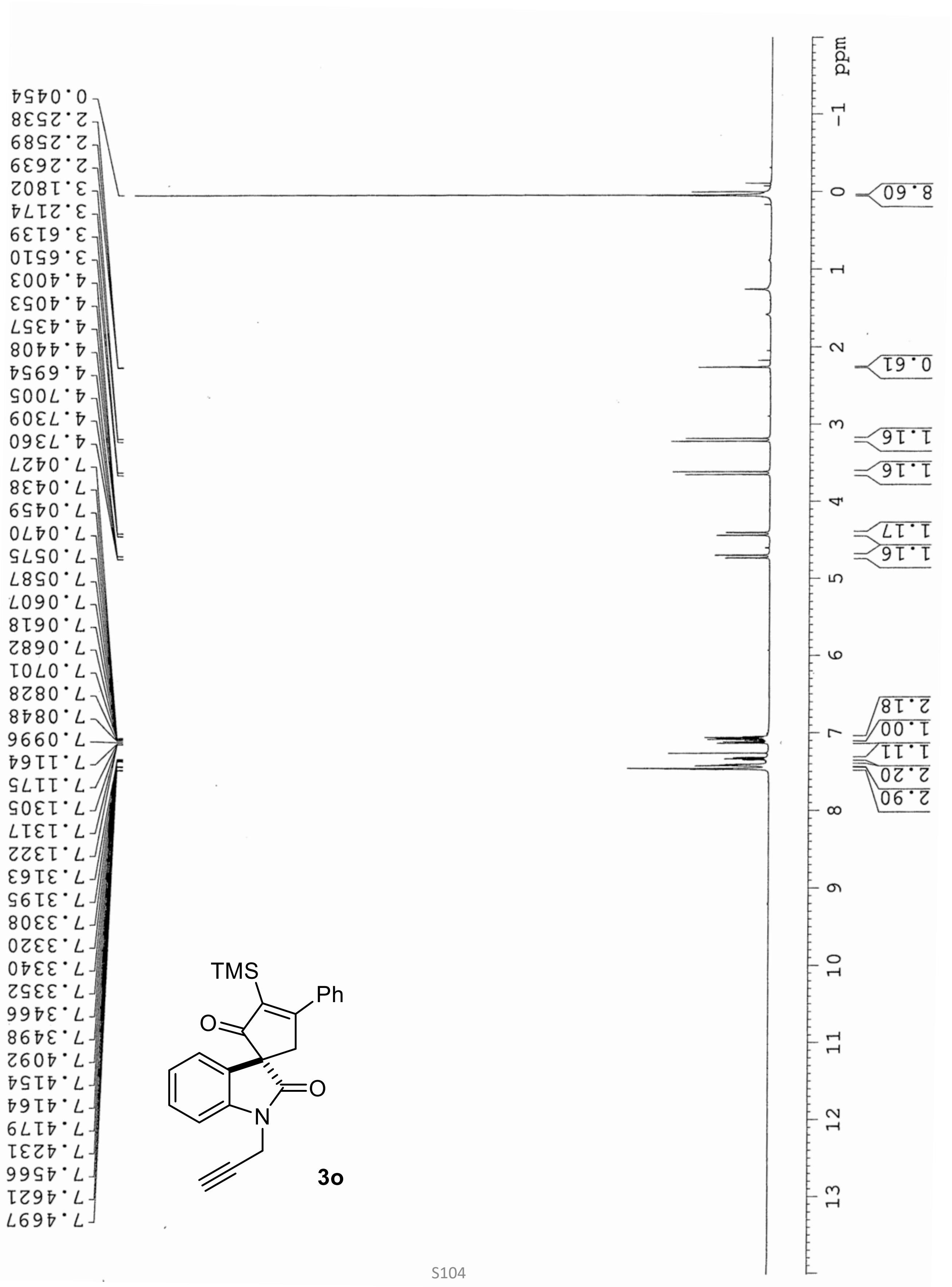
0.0454
2.2538
2.2589
2.2639
3.1802
3.2174
3.6139
3.6510
4.4003
4.4053
4.4357
4.4408
4.6954
4.7005
4.7309
4.7360
7.0427
7.0438
7.0459
7.0470
7.0575
7.0587
7.0607
7.0618
7.0682
7.0701
7.0828
7.0848
7.0996
7.1164
7.1175
7.1305
7.1317
7.1322
7.3163
7.3195
7.3308
7.3320
7.3340
7.3352
7.3466
7.3498
7.4092
7.4154
7.4164
7.4179
7.4231
7.4566
7.4621
7.4697
ppm
-1
0
1
2
3
4
5
6
7
8
9
10
11
12
13
8.60
0.61
1.16
1.16
1.17
1.16
2.18
1.00
1.11
2.20
2.90
TMS
Ph
O
O
N
3o

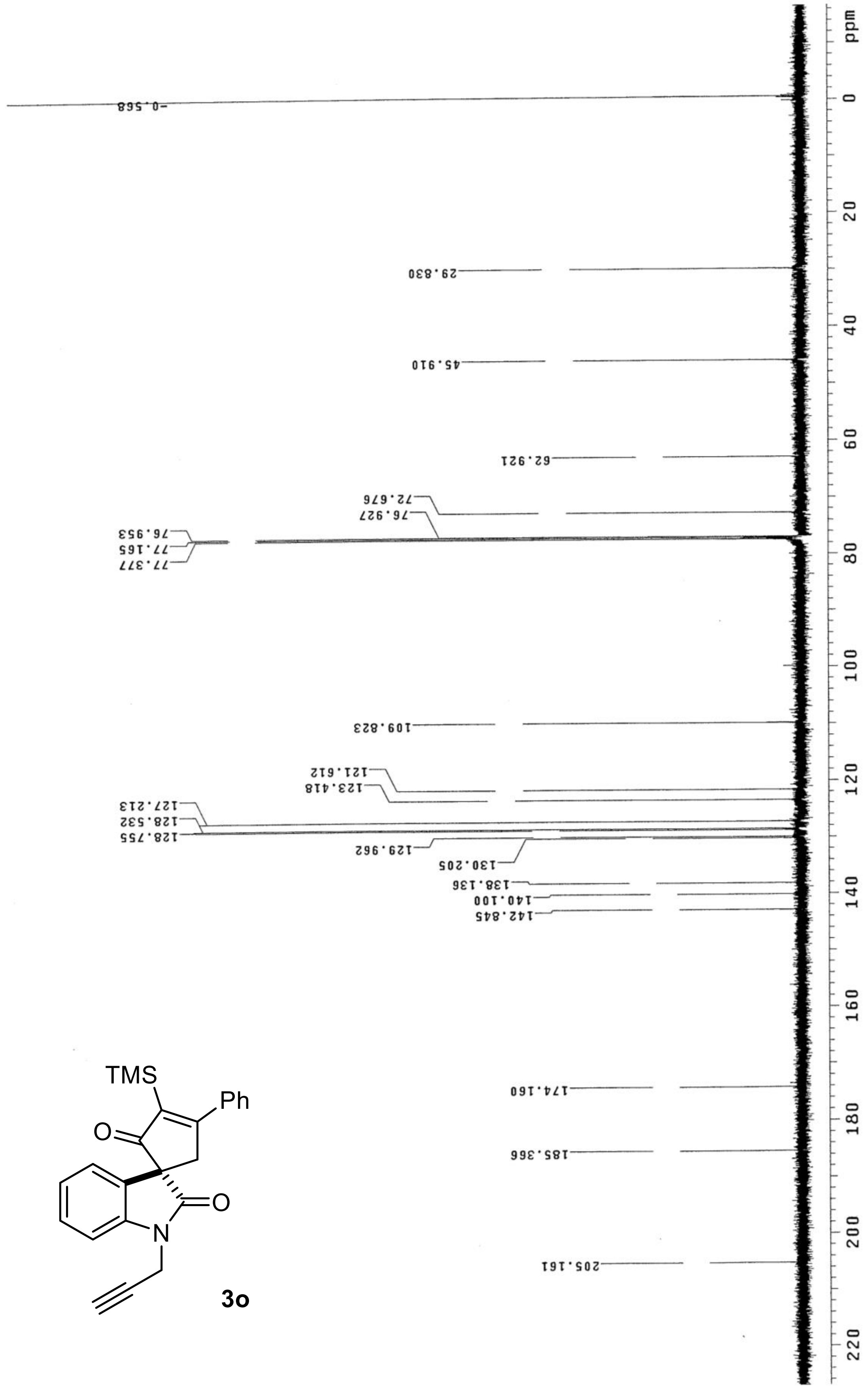

-0.568
29.830
45.910
62.921
72.676
76.927
76.953
77.165
77.377
109.823
121.612
123.418
127.213
128.532
128.755
129.962
130.205
138.136
140.100
142.845
174.160
185.366
205.161
ppm
0
20
40
60
80
100
120
140
160
180
200
220
TMS
Ph
O
O
N
3o

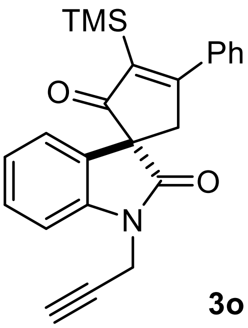

3o

Racemic

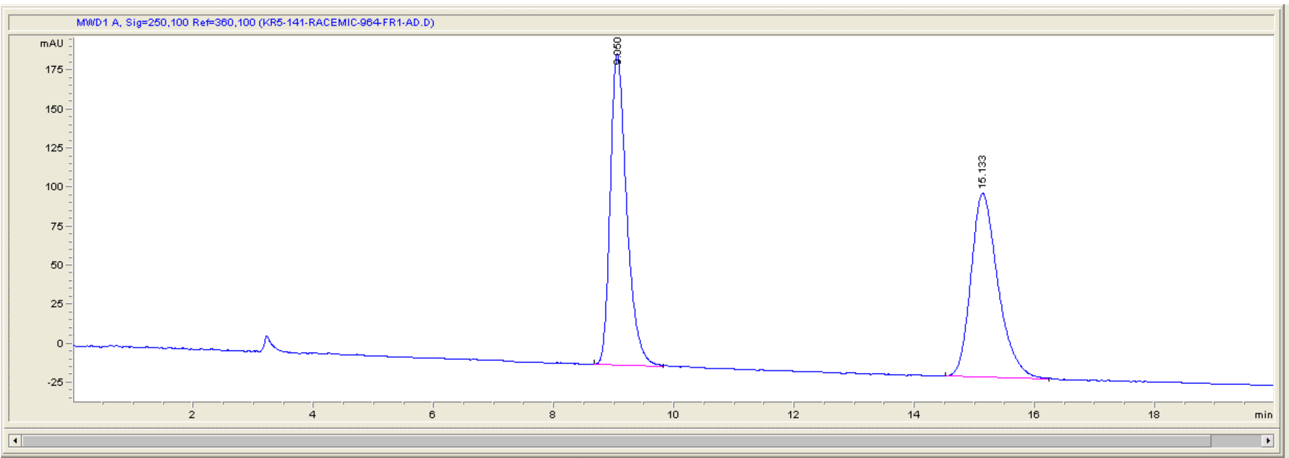

File Information

LC-File	KR5-141-RACEMIC-964-FR1-AD.D
File Path	C:\CHEM32\2\DATA\ERIN\
Date	14-Dec-17, 13:25:25

#	Time	Area	Height	Width	Area%	Symmetry
1	9.05	3820.7	200	0.2909	50.655	0.672
2	15.133	3721.9	118.2	0.4754	49.345	0.715

Rh_2(*S*-TCPTTL)$_4$: 90% ee

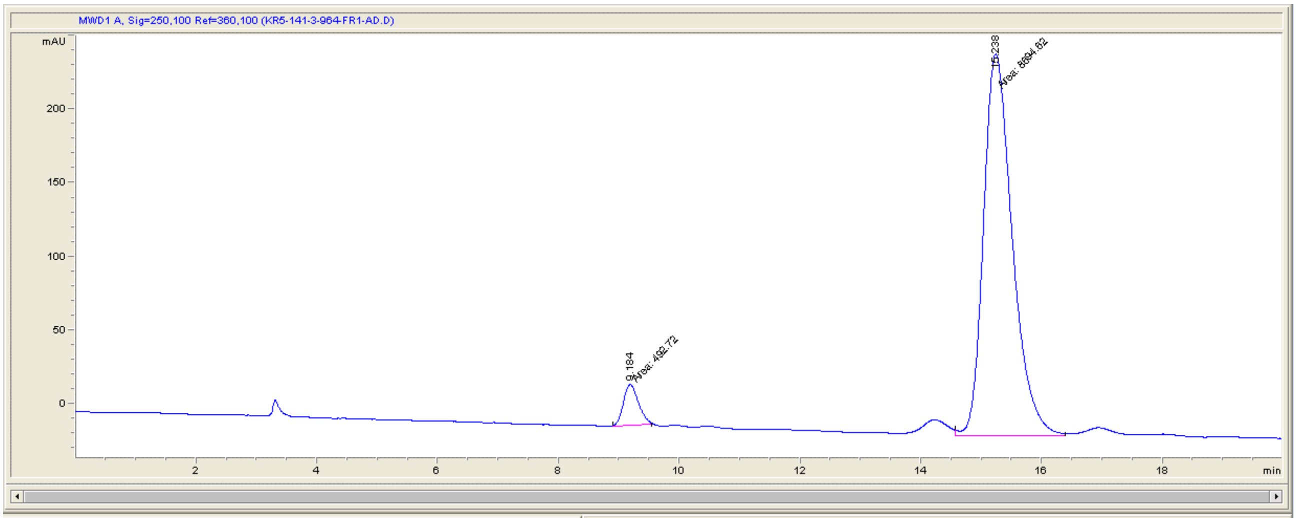

File Information

LC-File	KR5-141-3-964-FR1-AD.D
File Path	C:\CHEM32\2\DATA\ERIN\

#	Time	Area	Height	Width	Area%	Symmetry
1	9.184	492.7	28	0.2928	5.363	0.814
2	15.238	8694.6	260.2	0.5569	94.637	0.714

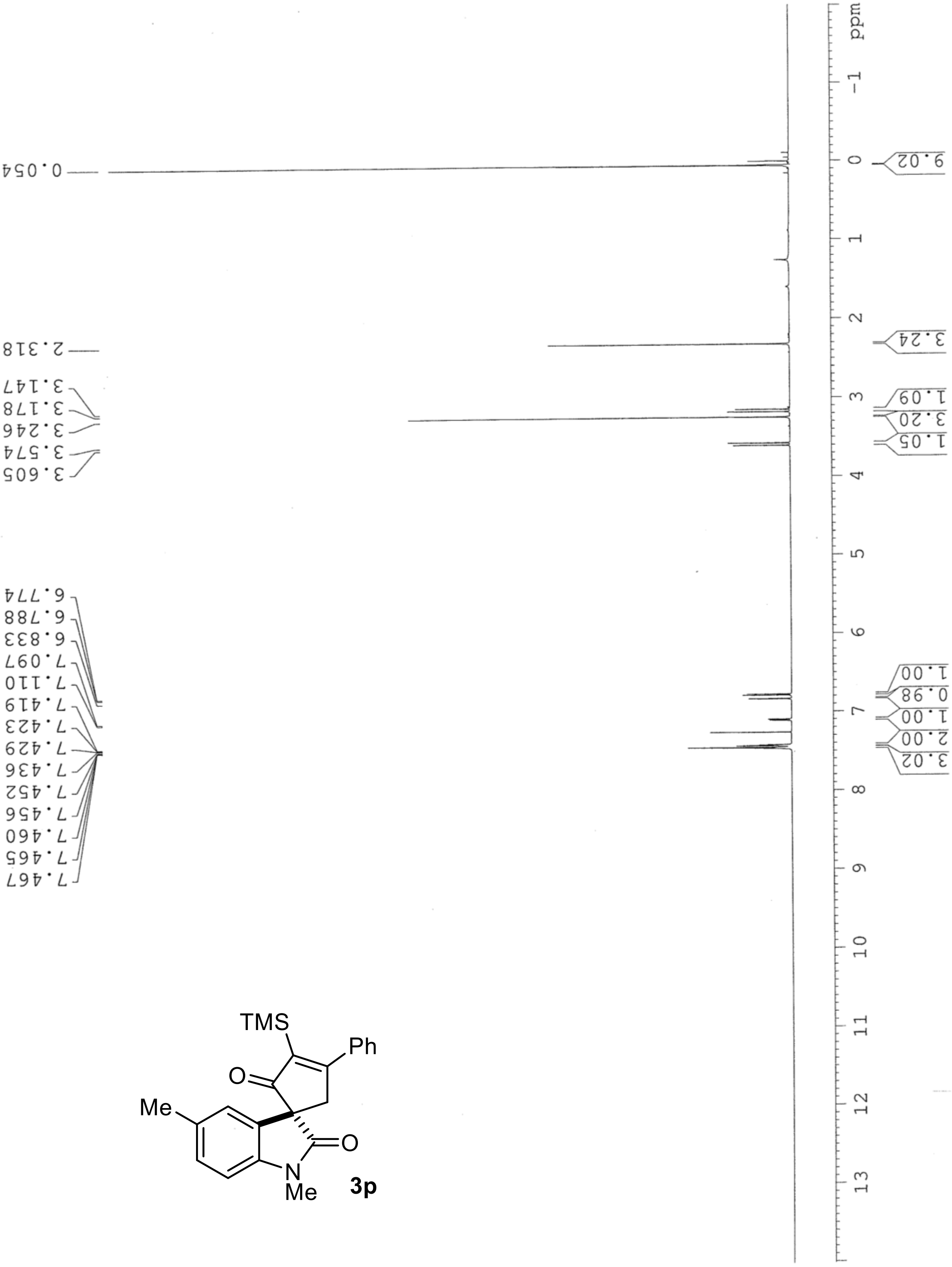

0.054
2.318
3.147
3.178
3.246
3.574
3.605
6.774
6.788
6.833
7.097
7.110
7.419
7.423
7.429
7.436
7.452
7.456
7.460
7.465
7.467
9.02
3.24
1.09
3.20
1.05
1.00
0.98
1.00
2.00
3.02
ppm
-1 0 1 2 3 4 5 6 7 8 9 10 11 12 13
TMS
Ph
O
Me
O
N
Me
3p

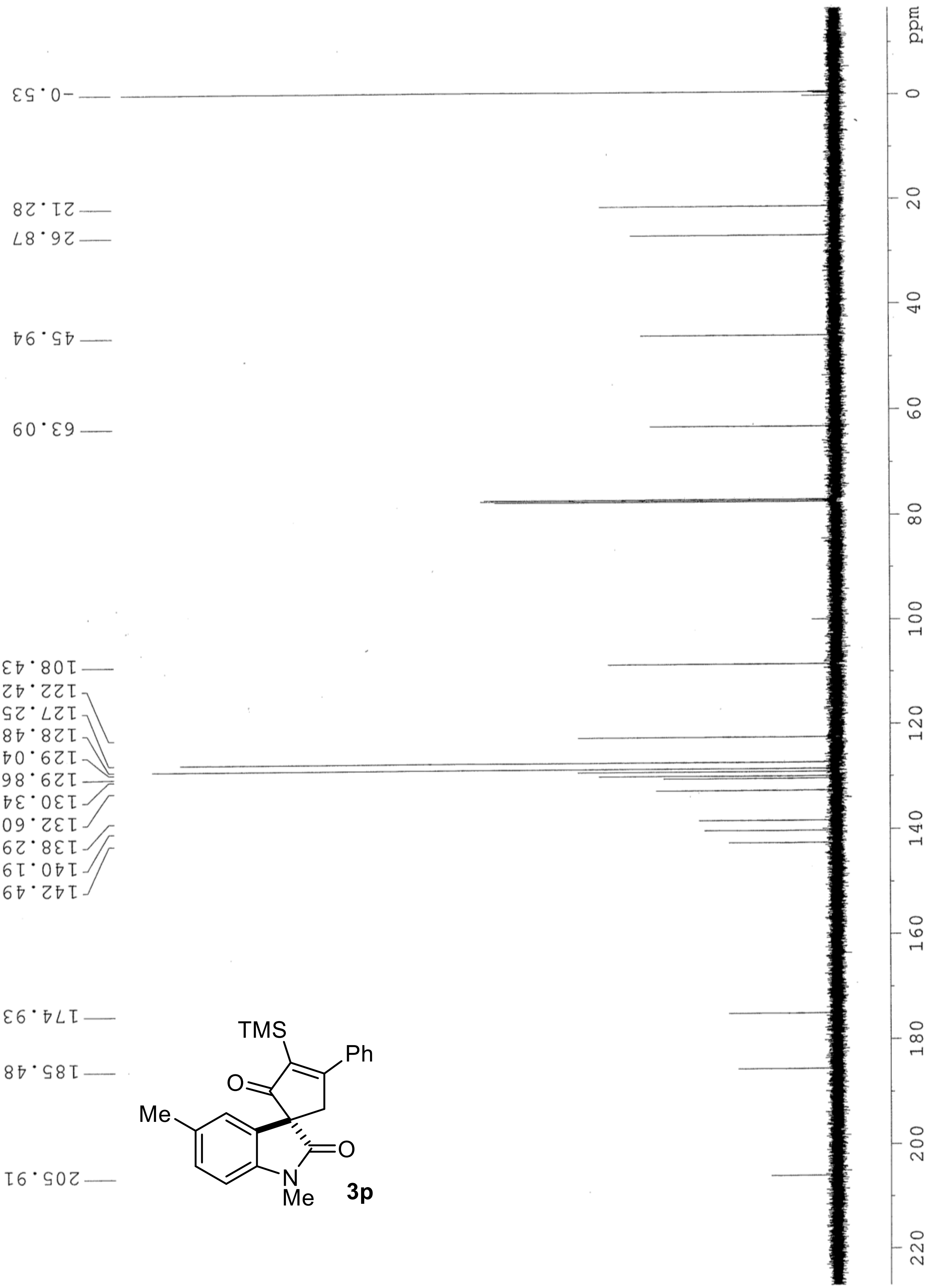
-0.53
21.28
26.87
45.94
63.09
108.43
122.42
127.25
128.48
129.04
129.86
130.34
132.60
138.29
140.19
142.49
174.93
185.48
205.91
ppm
0
20
40
60
80
100
120
140
160
180
200
220
TMS
Ph
O
Me
O
N
Me
3p

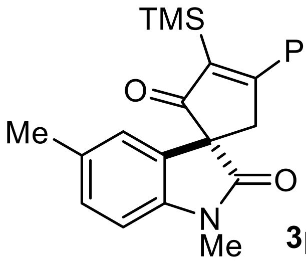

3p

Racemic

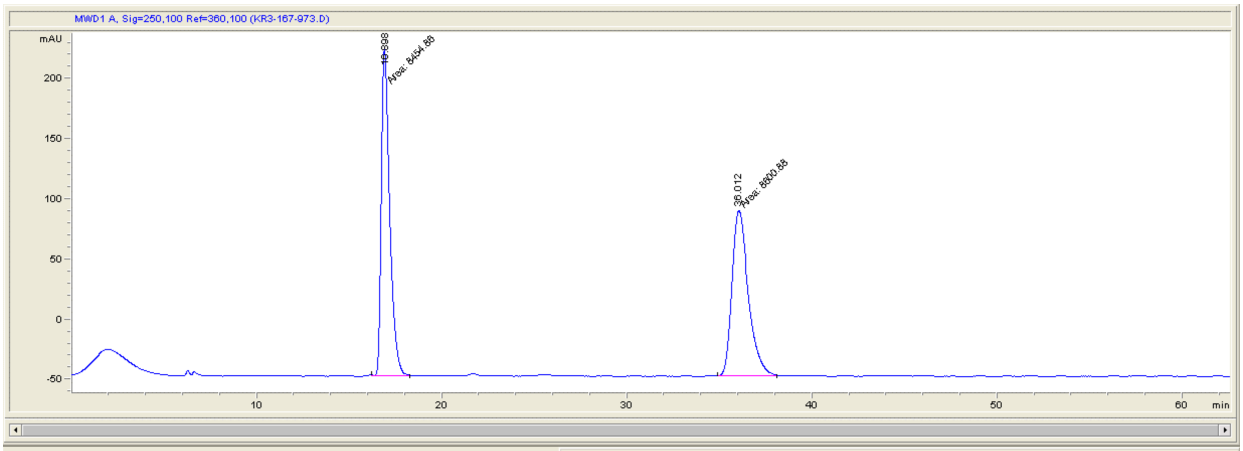

File Information

LC-File	KR3-167-973.D

#	Time	Area	Height	Width	Area%	Symmetry
1	16.898	8454.9	271.8	0.5184	49.572	0.585
2	36.012	8600.9	137.5	1.0424	50.428	0.654

Rh_2(*S*-TCPTTL)$_4$: 90% ee

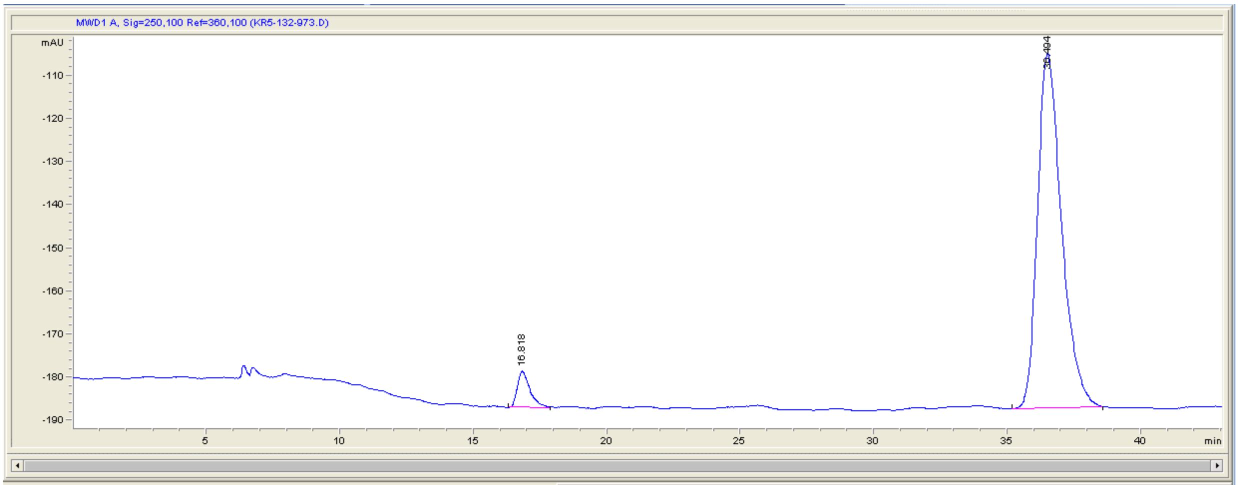

File Information

LC-File	KR5-132-973.D
File Path	C:\CHEM32\2\DATA\ERIN\

#	Time	Area	Height	Width	Area%	Symmetry
1	16.818	283.1	8.6	0.4492	5.193	0.622
2	36.494	5168.3	82.1	0.8966	94.807	0.664

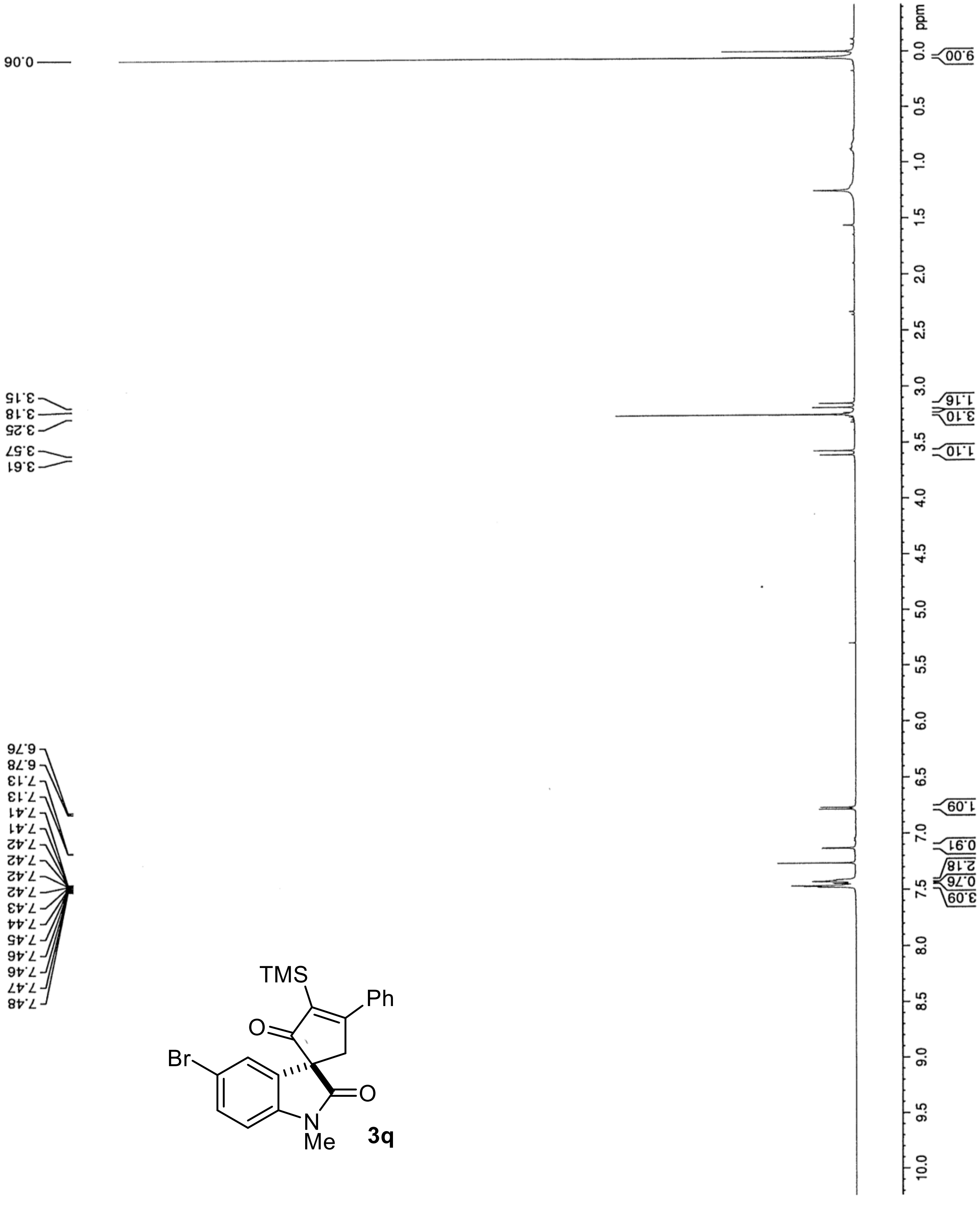
0.06
3.15
3.18
3.25
3.57
3.61
6.76
6.78
7.13
7.13
7.41
7.41
7.42
7.42
7.42
7.42
7.43
7.44
7.45
7.46
7.46
7.47
7.48
ppm
9.00
1.16
3.10
1.10
1.09
0.91
2.18
0.76
3.09
TMS
Ph
O
Br
O
N
Me

3q

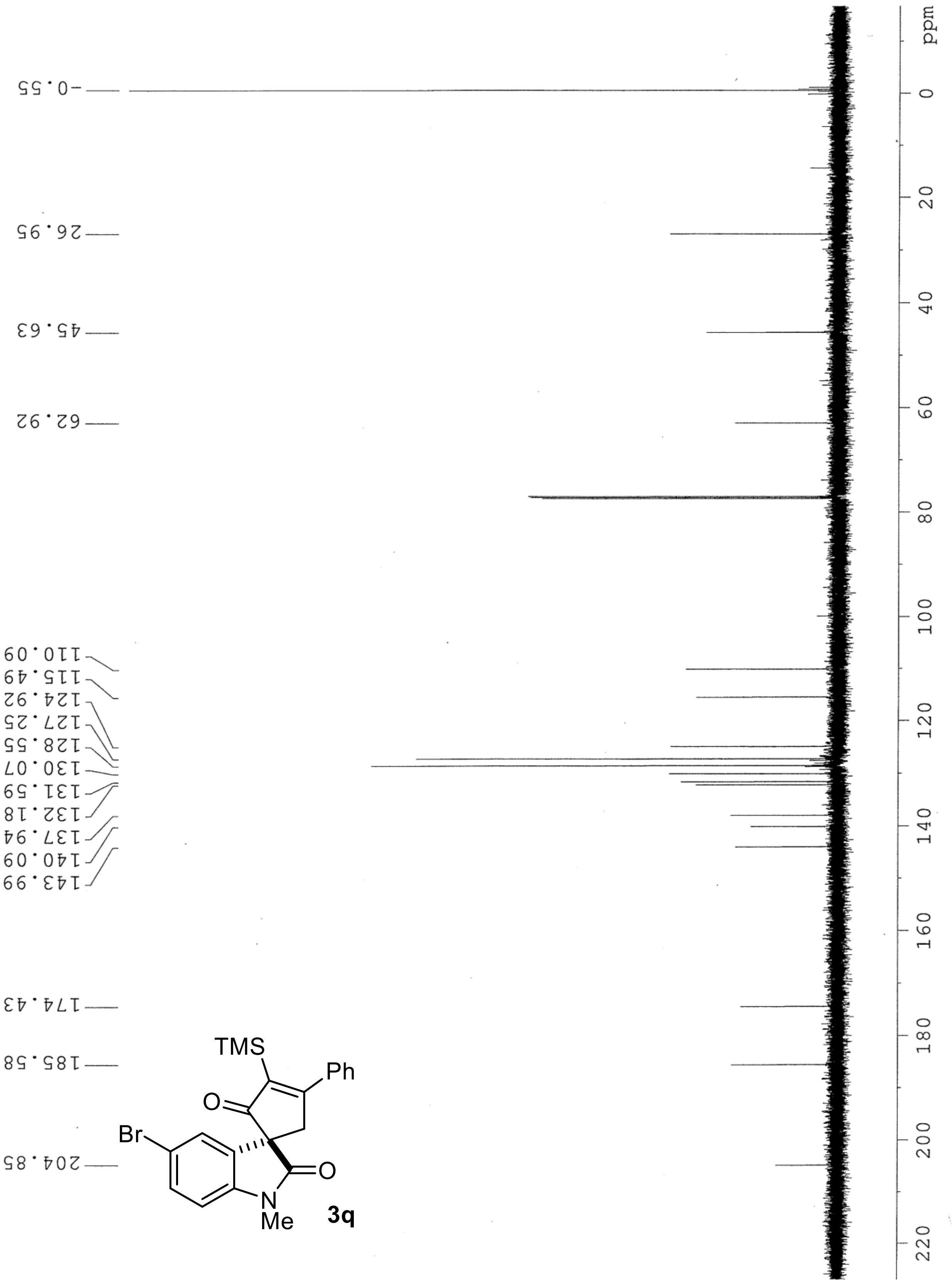
-0.55
26.95
45.63
62.92
110.09
115.49
124.92
127.25
128.55
130.07
131.59
132.18
137.94
140.09
143.99
174.43
185.58
204.85
ppm
TMS
Ph
O
Br
O
N
Me
3q

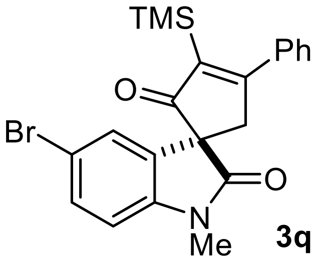

3q

Racemic

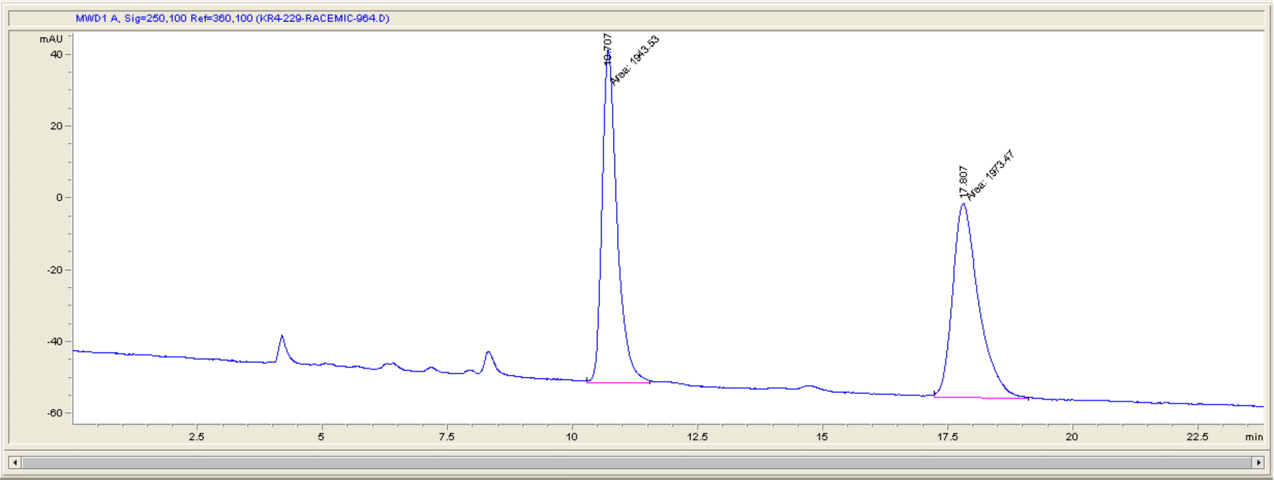

File Information

LC-File	KR4-229-RACEMIC-964.D
File Path	C:\CHEM32\2\DATA\ERIN\
Date	20-Oct-16 14:42:35

#	Time	Area	Height	Width	Area%	Symmetry
1	10.707	1943.5	92.6	0.3496	49.618	0.666
2	17.807	1973.5	54.1	0.6078	50.382	0.665

Rh_2(*S*-TCPTTL)$_4$: 77% ee

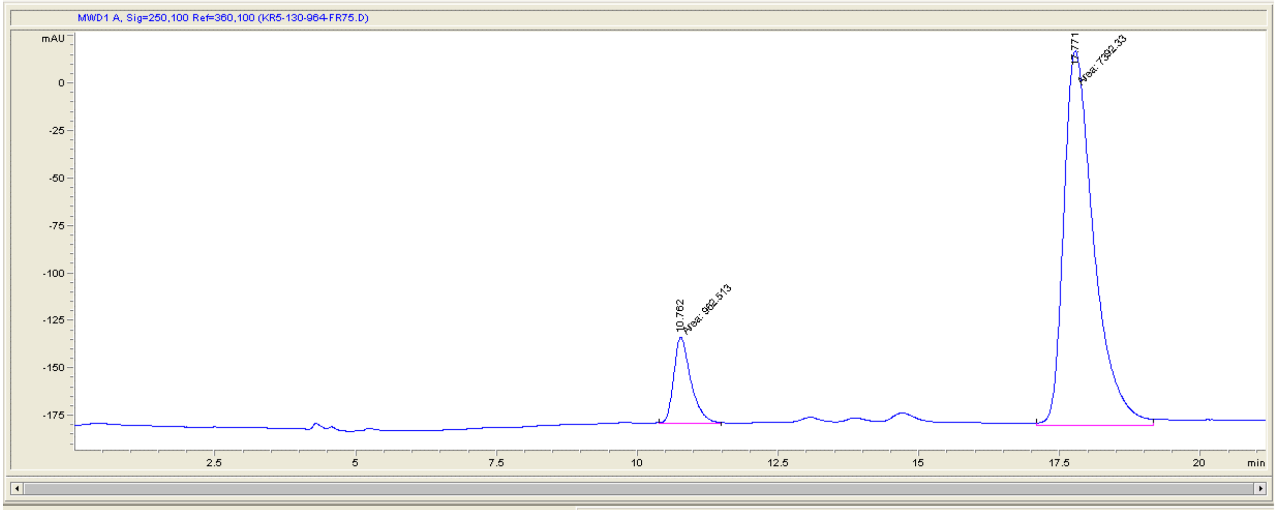

File Information

LC-File	KR5-130-964-FR75.D
File Path	C:\CHEM32\2\DATA\ERIN\

#	Time	Area	Height	Width	Area%	Symmetry
1	10.762	962.5	45.4	0.3535	11.520	0.657
2	17.771	7392.3	197.6	0.6236	88.480	0.601

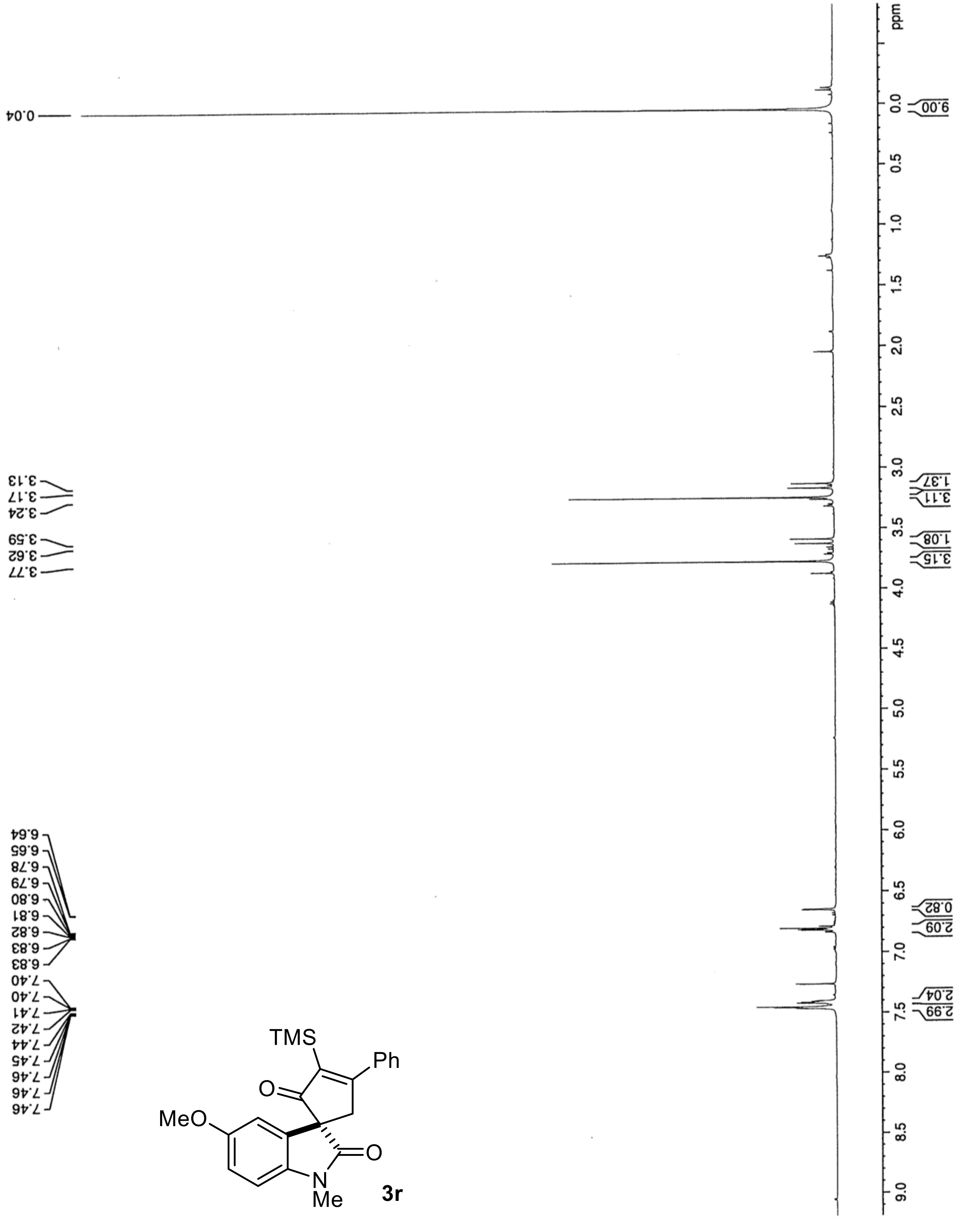
0.04
3.13
3.17
3.24
3.59
3.62
3.77
6.64
6.65
6.78
6.79
6.80
6.81
6.82
6.83
6.83
7.40
7.40
7.41
7.42
7.44
7.45
7.46
7.46
7.46
TMS
Ph
O
MeO
O
N
Me
ppm
9.00
1.37
3.11
1.08
3.15
0.82
2.09
2.04
2.99

3r

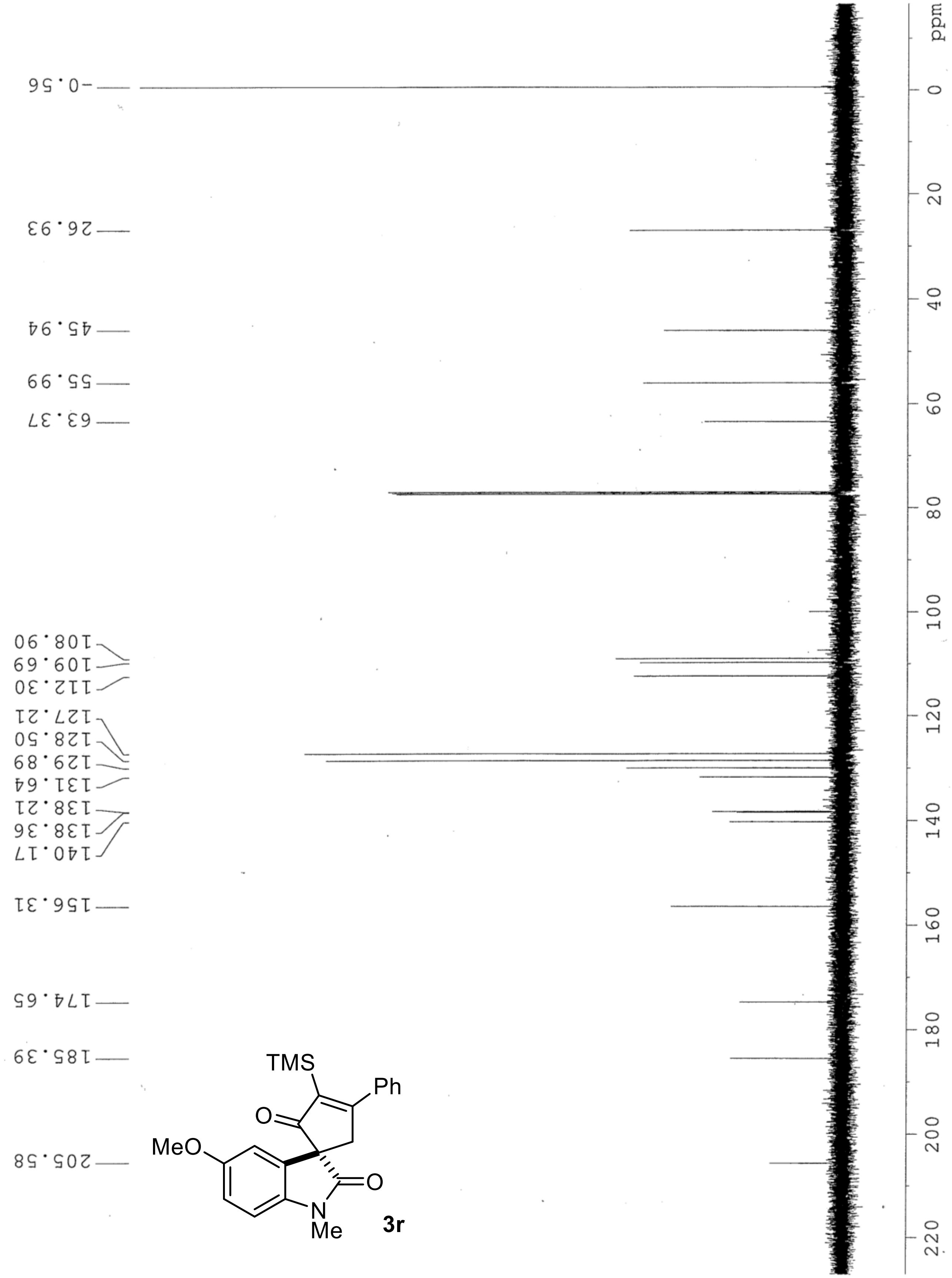
-0.56
26.93
45.94
55.99
63.37
108.90
109.69
112.30
127.21
128.50
129.89
131.64
138.21
138.36
140.17
156.31
174.65
185.39
205.58
ppm
TMS
Ph
MeO
O
N
Me

3r

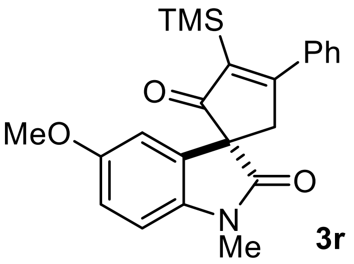

3r

Racemic

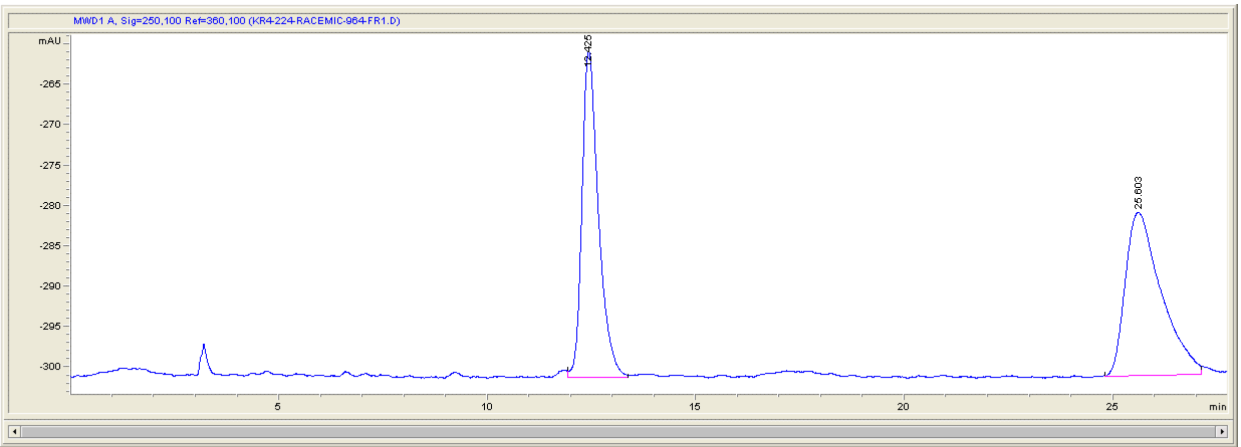

File Information

LC-File | KR4-224-RACEMIC-964-FR1.D

#	Time	Area	Height	Width	Area%	Symmetry
1	12.425	1118.4	40.5	0.4083	47.839	0.665
2	25.603	1219.4	20.3	0.7858	52.161	0.545

Rh_2(*S*-TCPTTL)$_4$: 84% ee

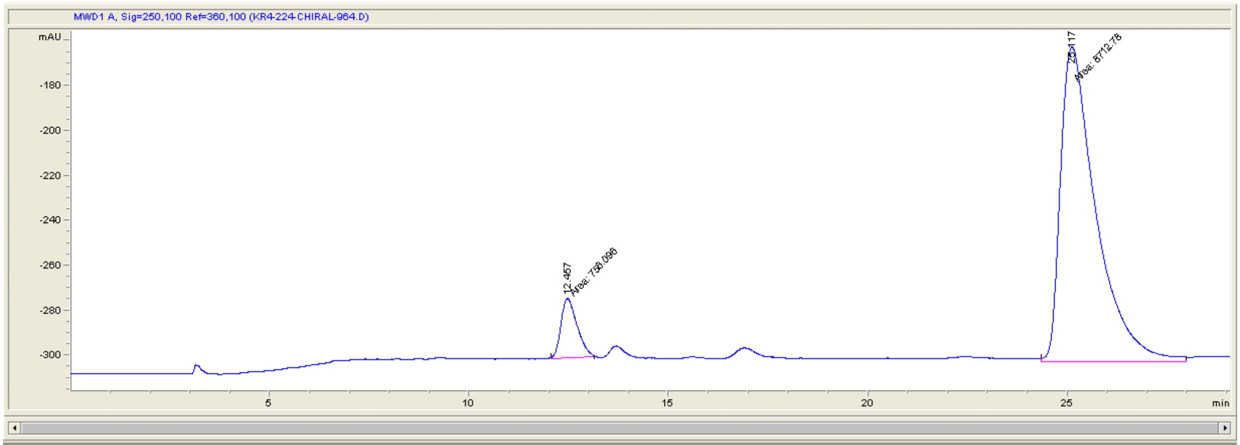

File Information

LC-File | KR4-224-CHIRAL-964.D

#	Time	Area	Height	Width	Area%	Symmetry
1	12.457	756.1	26.5	0.4748	7.985	0.635
2	25.117	8712.8	140.3	1.035	92.015	0.463

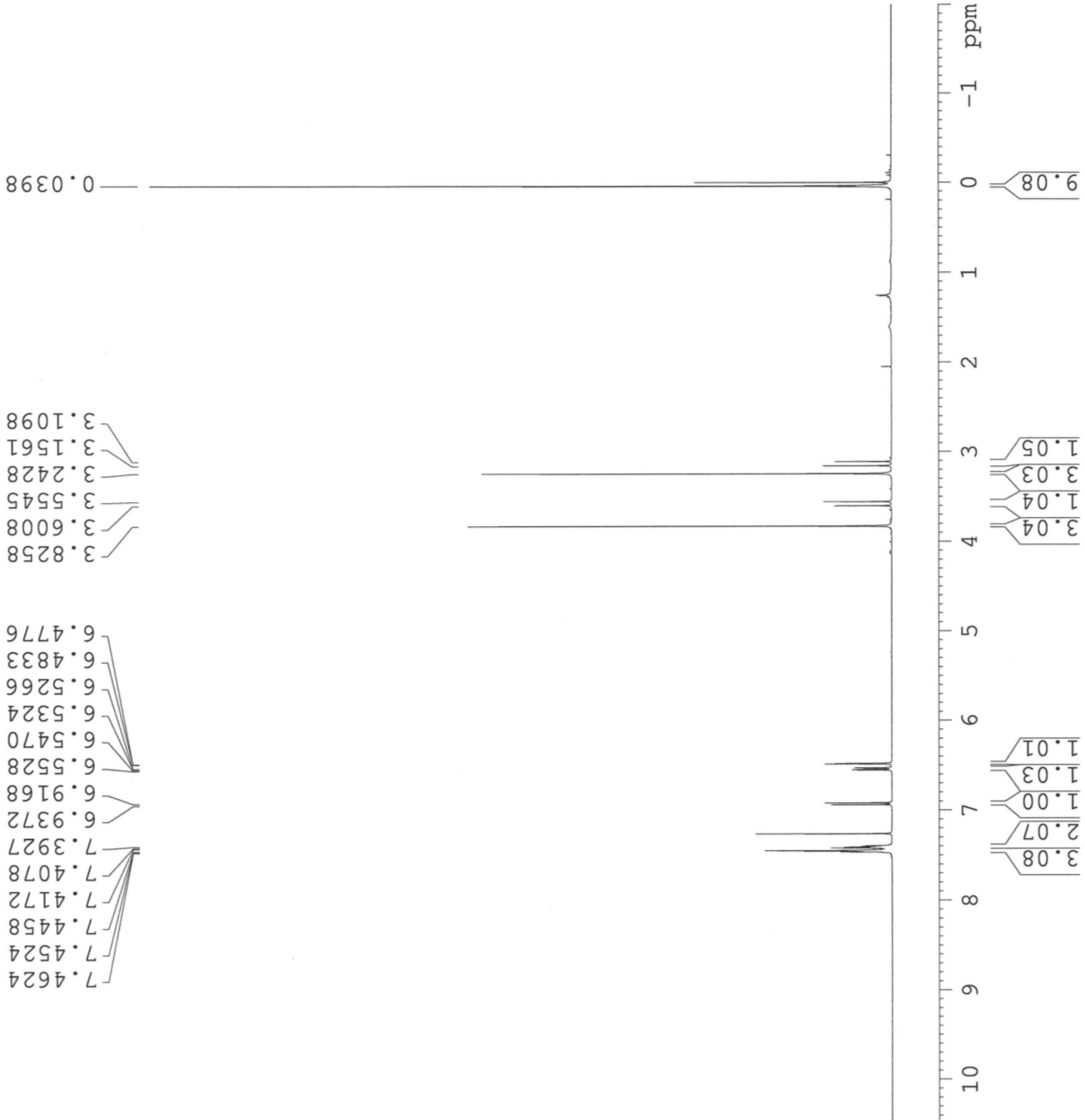
0.0398
3.1098
3.1561
3.2428
3.5545
3.6008
3.8258
6.4776
6.4833
6.5266
6.5324
6.5470
6.5528
6.9168
6.9372
7.3927
7.4078
7.4172
7.4458
7.4524
7.4624
9.08
1.05
3.03
1.04
3.04
1.01
1.03
1.00
2.07
3.08
ppm

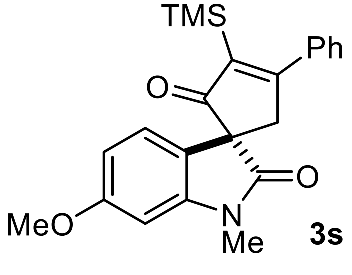
TMS
Ph
O
O
MeO
N
Me

3s

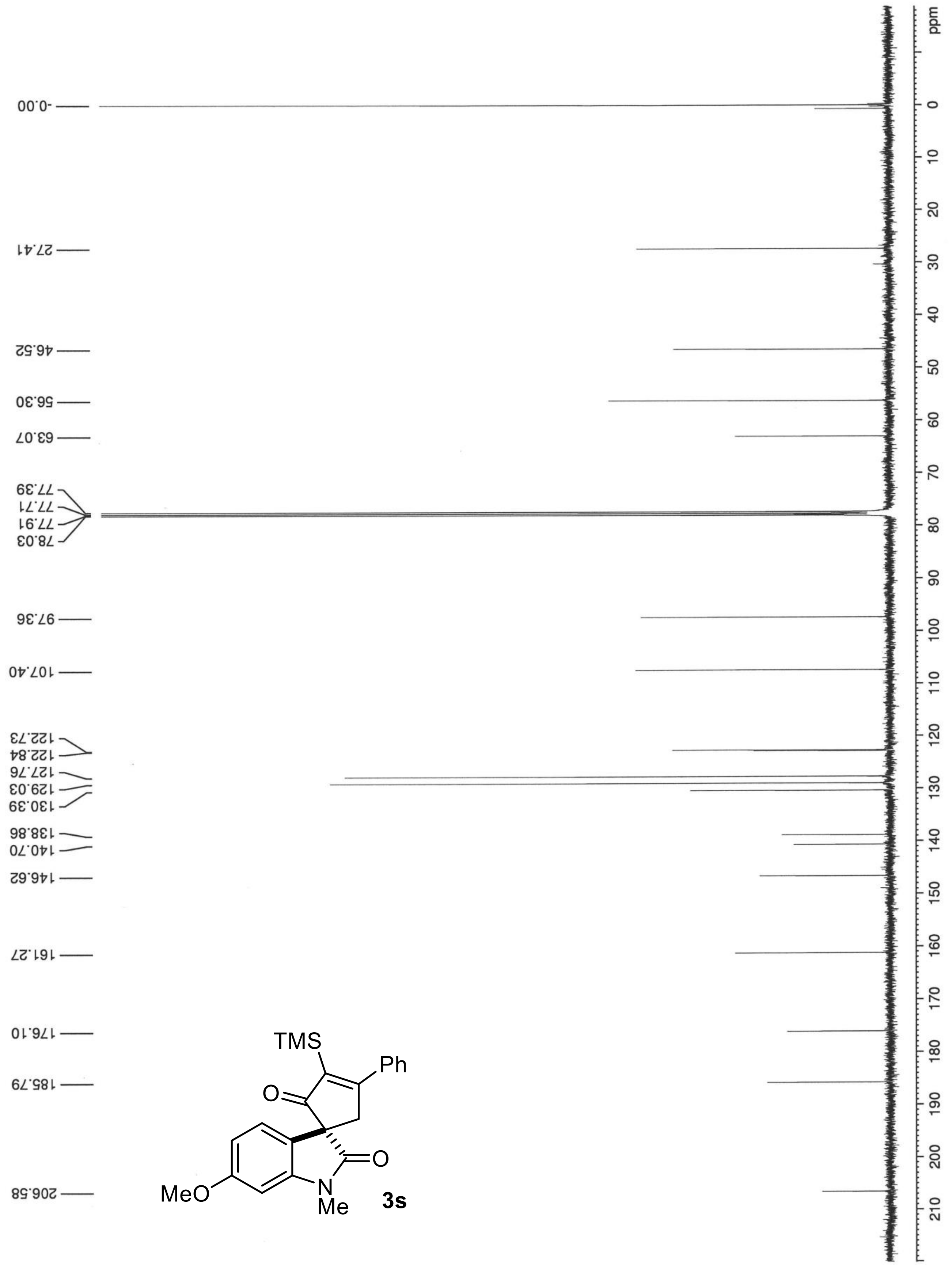
-0.00
27.41
46.52
56.30
63.07
77.39
77.71
77.91
78.03
97.36
107.40
122.73
122.84
127.76
129.03
130.39
138.86
140.70
146.62
161.27
176.10
185.79
206.58
ppm
TMS
Ph
O
MeO
N
Me
3s

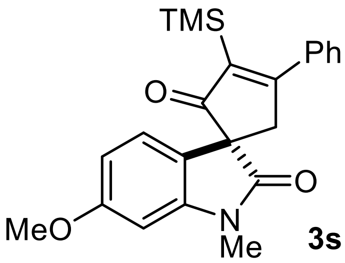

3s

Racemic

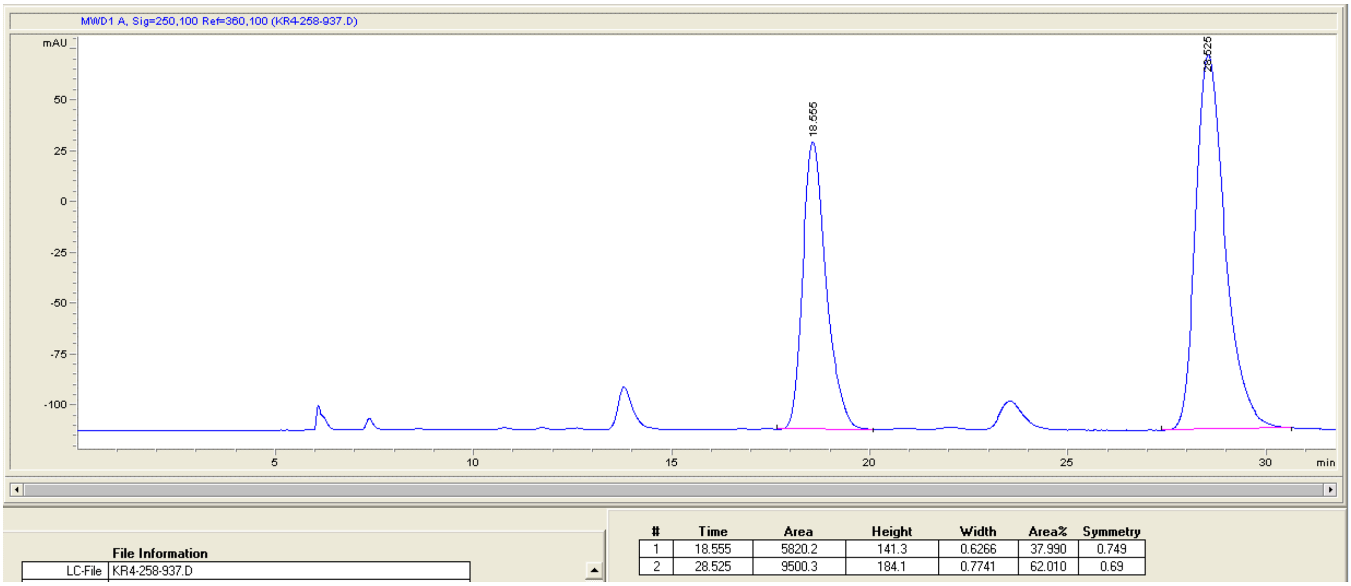

#	Time	Area	Height	Width	Area%	Symmetry
1	18.555	5820.2	141.3	0.6266	37.990	0.749
2	28.525	9500.3	184.1	0.7741	62.010	0.69

Rh_2(*S*-TCPTTL)$_4$: 86% ee

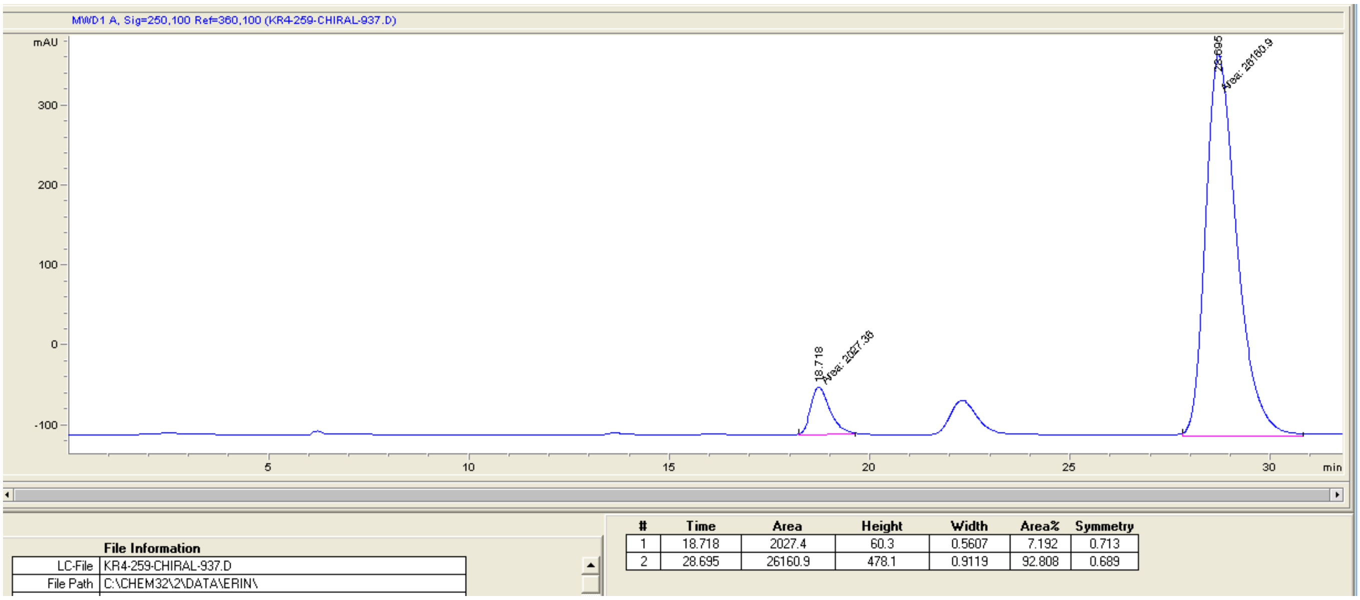

#	Time	Area	Height	Width	Area%	Symmetry
1	18.718	2027.4	60.3	0.5607	7.192	0.713
2	28.695	26160.9	478.1	0.9119	92.808	0.689

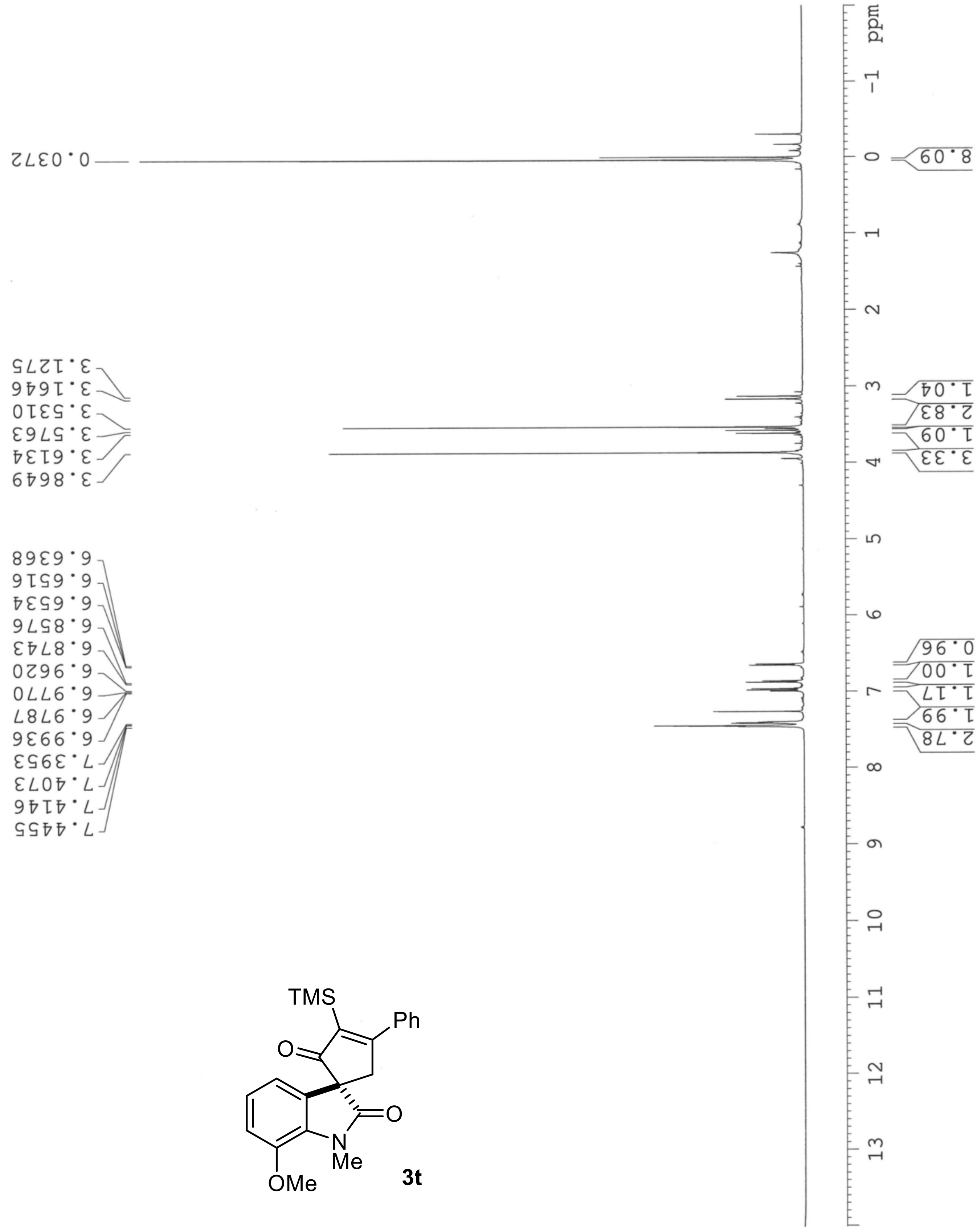

0.0372
3.1275
3.1646
3.5310
3.5763
3.6134
3.8649
6.6368
6.6516
6.6534
6.8576
6.8743
6.9620
6.9770
6.9787
6.9936
7.3953
7.4073
7.4146
7.4455
8.09
1.04
2.83
1.09
3.33
0.96
1.00
1.17
1.99
2.78
ppm
-1
0
1
2
3
4
5
6
7
8
9
10
11
12
13
TMS
Ph
O
O
N
Me
OMe
3t

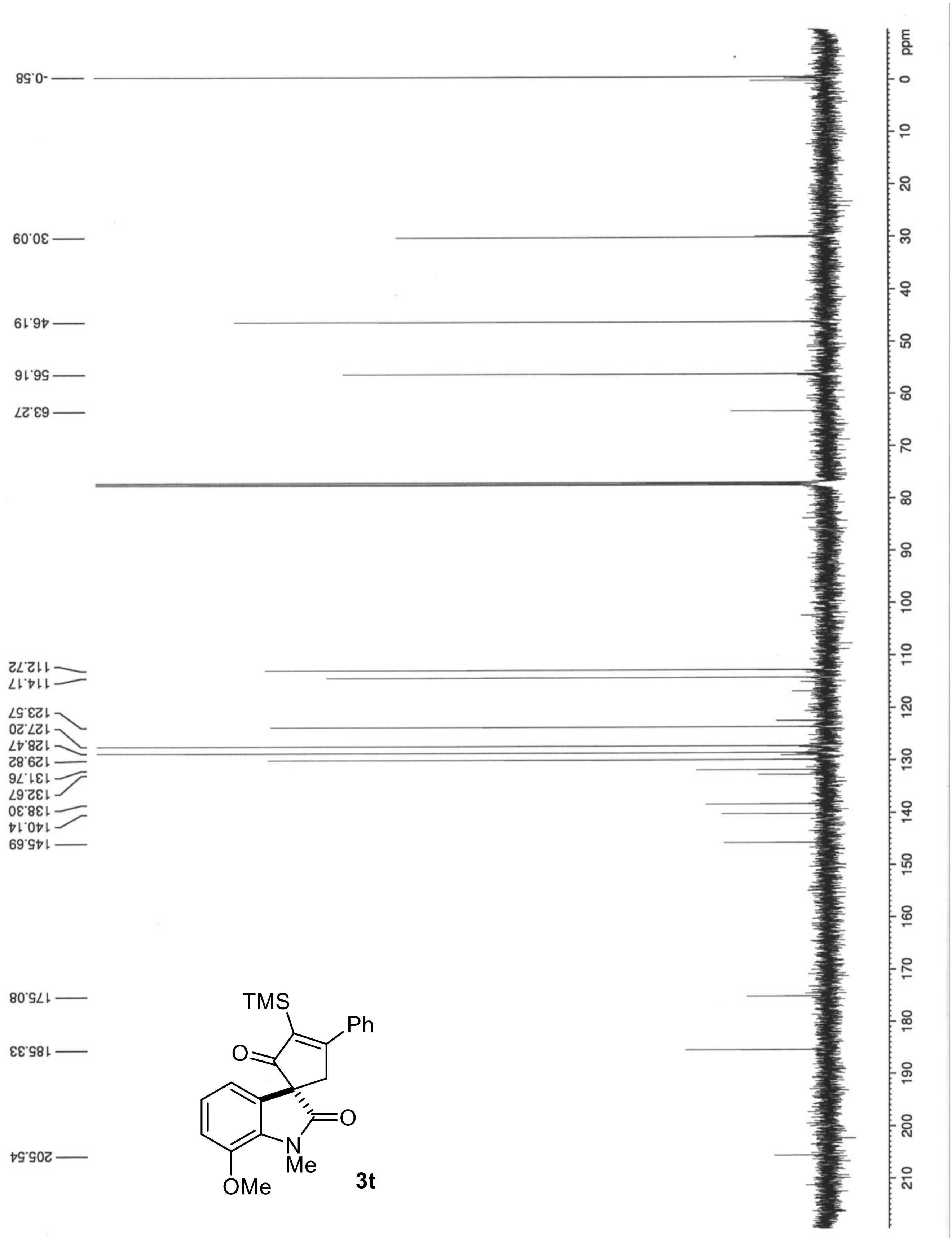
-0.58
30.09
46.19
56.16
63.27
112.72
114.17
123.57
127.20
128.47
129.82
131.76
132.67
138.30
140.14
145.69
175.08
185.33
205.54
ppm
0
10
20
30
40
50
60
70
80
90
100
110
120
130
140
150
160
170
180
190
200
210
TMS
Ph
O
O
N
Me
OMe
3t

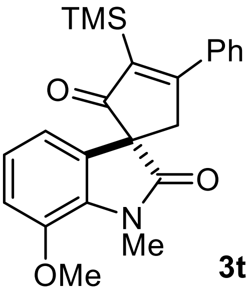

3t

Racemic

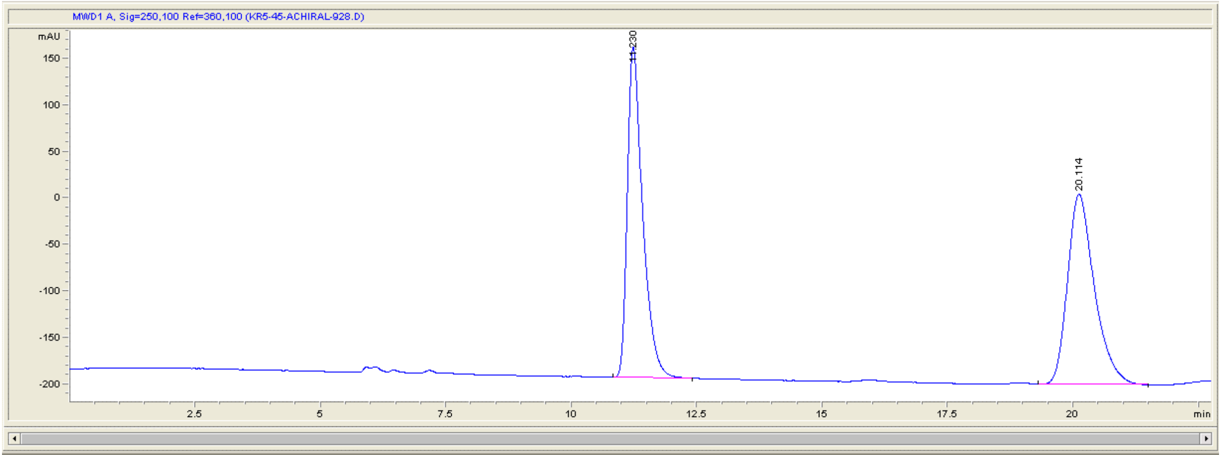

File Information

LC-File KR5-45-ACHIRAL-928.D

#	Time	Area	Height	Width	Area%	Symmetry
1	11.23	7594.3	355.5	0.3172	49.815	0.581
2	20.114	7650.6	204.5	0.5644	50.185	0.696

Rh_2(*S*-TCPTTL)$_4$: 88% ee

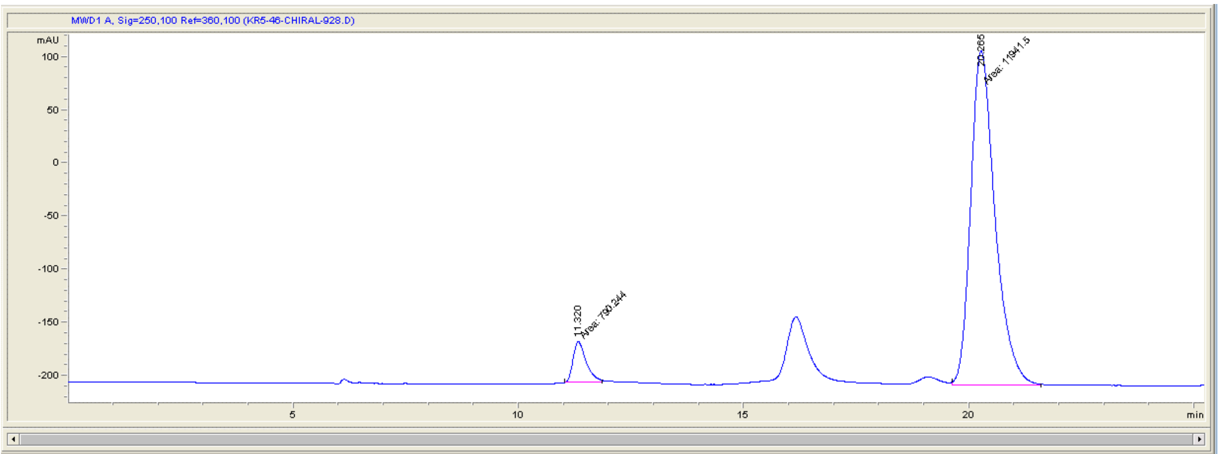

File Information

LC-File KR5-46-CHIRAL-928.D

#	Time	Area	Height	Width	Area%	Symmetry
1	11.32	790.2	38.7	0.3405	6.207	0.633
2	20.265	11941.5	315.2	0.6313	93.793	0.72

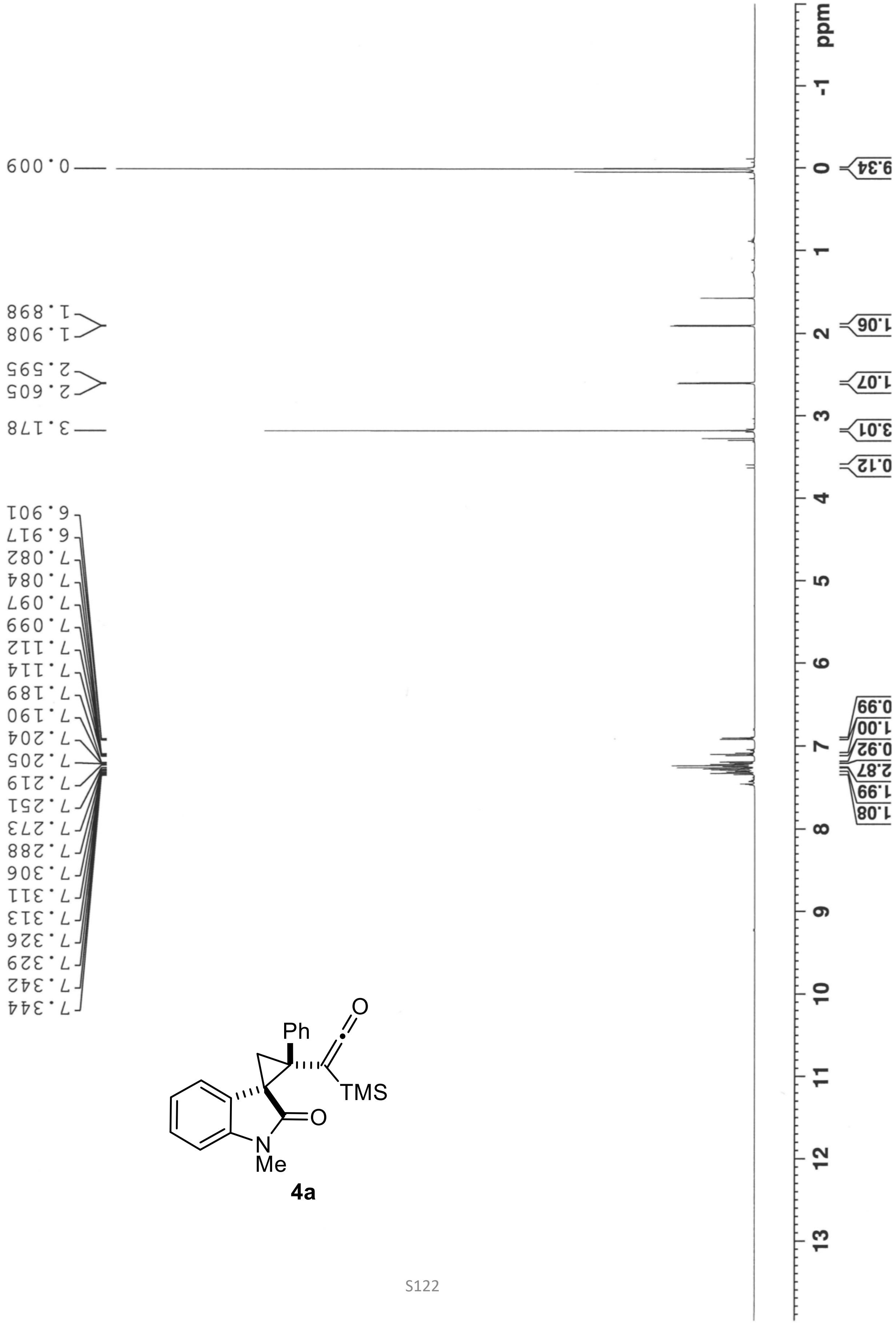
0.009
1.898
1.908
2.595
2.605
3.178
6.901
6.917
7.082
7.084
7.097
7.099
7.112
7.114
7.189
7.190
7.204
7.205
7.219
7.251
7.273
7.288
7.306
7.311
7.313
7.326
7.329
7.342
7.344
9.34
1.06
1.07
3.01
0.12
0.99
1.00
0.92
2.87
1.99
1.08
ppm
-1
0
1
2
3
4
5
6
7
8
9
10
11
12
13
Ph
O
TMS
O
N
Me
4a

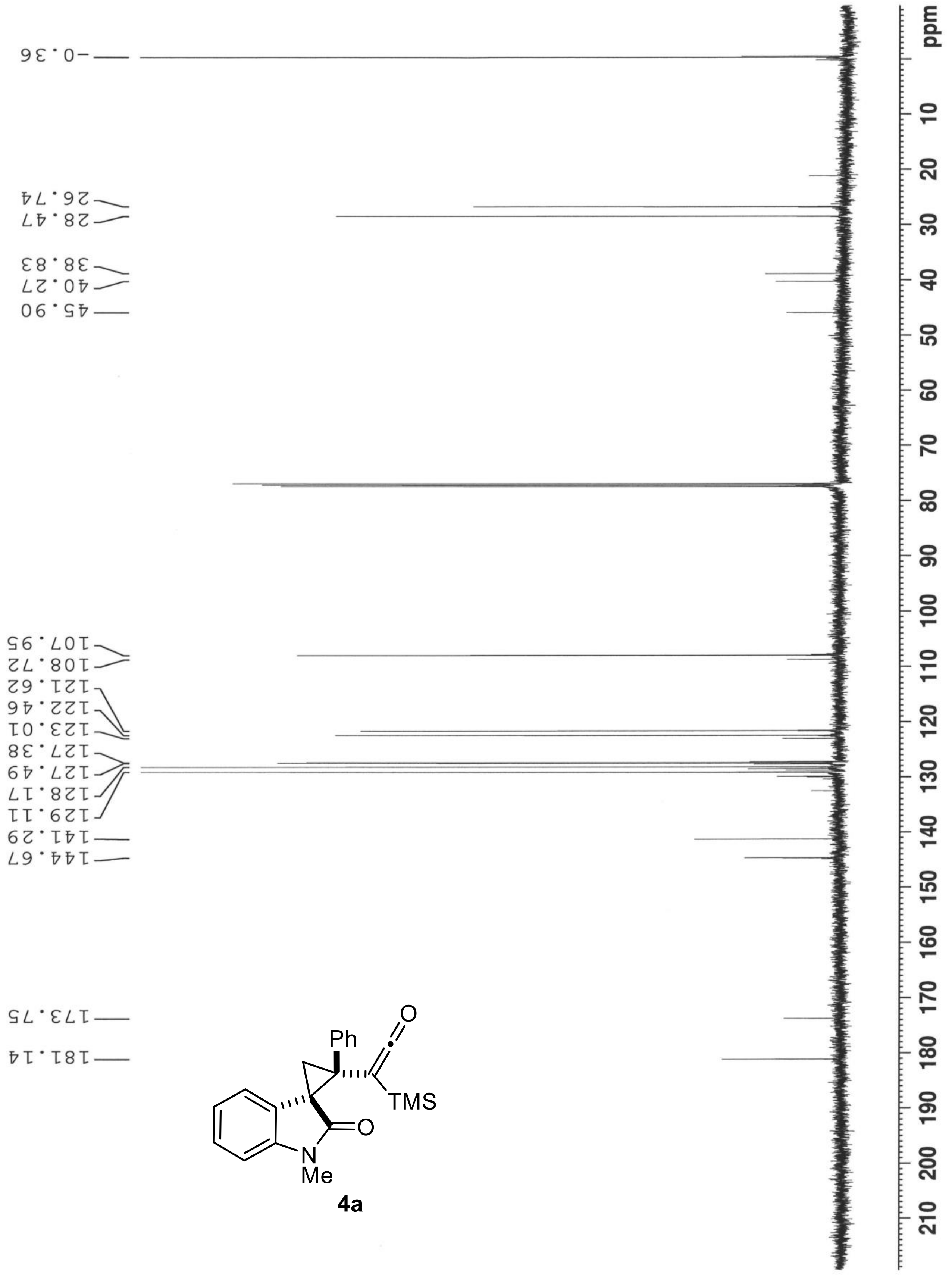

-0.36
26.74
28.47
38.83
40.27
45.90
107.95
108.72
121.62
122.46
123.01
127.38
127.49
128.17
129.11
141.29
144.67
173.75
181.14
ppm
10
20
30
40
50
60
70
80
90
100
110
120
130
140
150
160
170
180
190
200
210
O
Ph
TMS
O
N
Me

4a

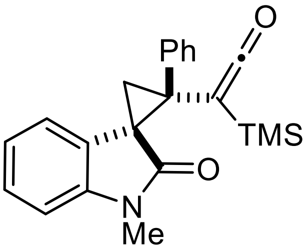

4a

Racemic

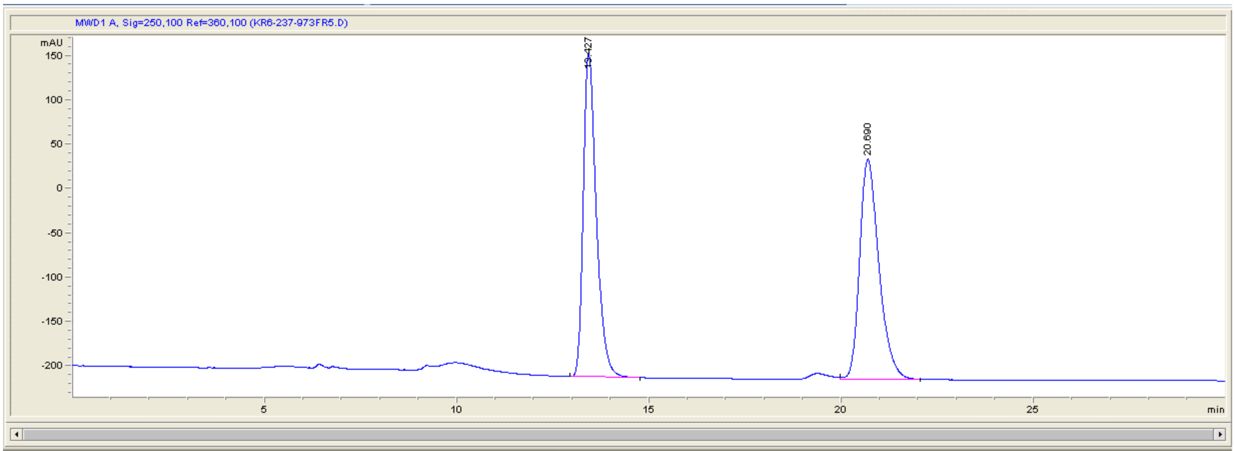

File Information	
LC-File	KR6-237-973FR5.D
File Path	C:\CHEM32\2\DATA\ERIN\

#	Time	Area	Height	Width	Area%	Symmetry
1	13.427	8616.7	365.2	0.3533	49.774	0.651
2	20.69	8695.1	248.4	0.5324	50.226	0.692

Rh_2(*S*-TCPTTL)$_4$: 95% ee

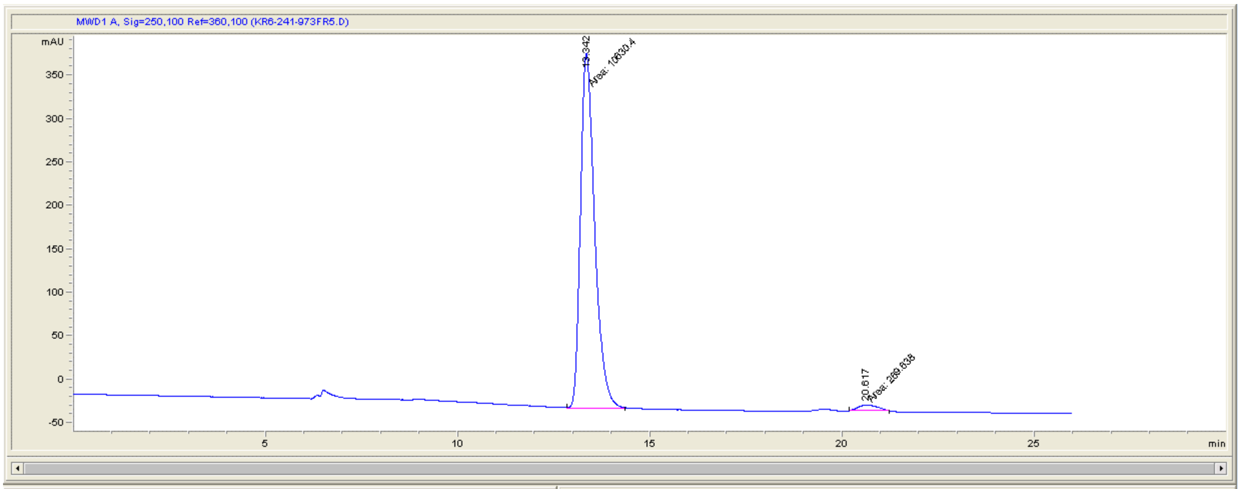

File Information	
LC-File	KR6-241-973FR5.D
File Path	C:\CHEM32\2\DATA\ERIN\
Date	14-Oct-17, 20:54:39

#	Time	Area	Height	Width	Area%	Symmetry
1	13.342	10630.4	409.5	0.4326	97.526	0.681
2	20.617	269.6	7.1	0.6331	2.474	0.665

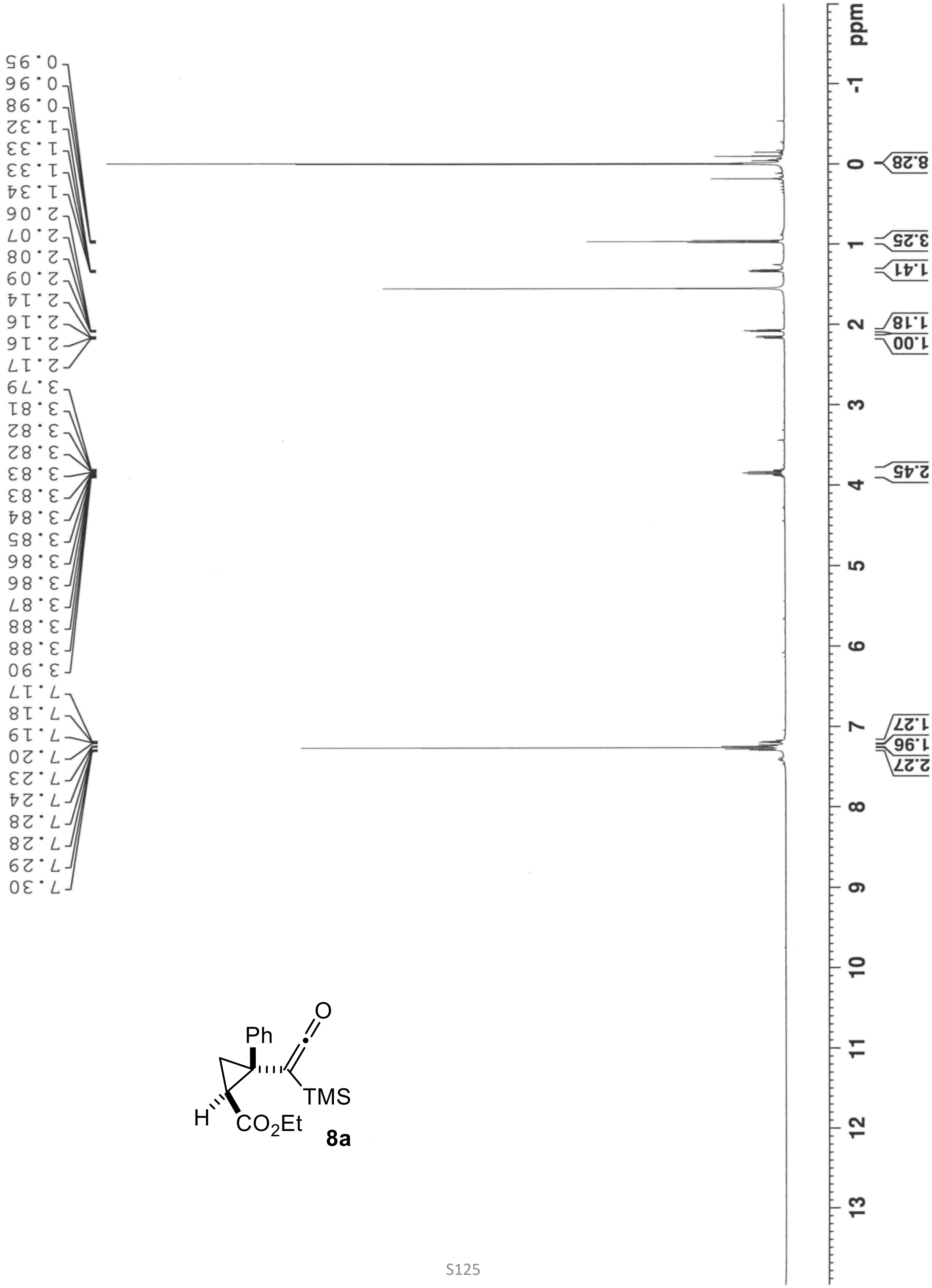
0.95
0.96
0.98
1.32
1.33
1.33
1.34
2.06
2.07
2.08
2.09
2.14
2.16
2.16
2.17
3.79
3.81
3.82
3.82
3.83
3.83
3.84
3.85
3.86
3.86
3.87
3.88
3.88
3.90
7.17
7.18
7.19
7.20
7.23
7.24
7.28
7.28
7.29
7.30
ppm
-1 0 1 2 3 4 5 6 7 8 9 10 11 12 13
8.28
3.25
1.41
1.18
1.00
2.45
1.27
1.96
2.27
Ph
O
TMS
H
CO2Et
8a

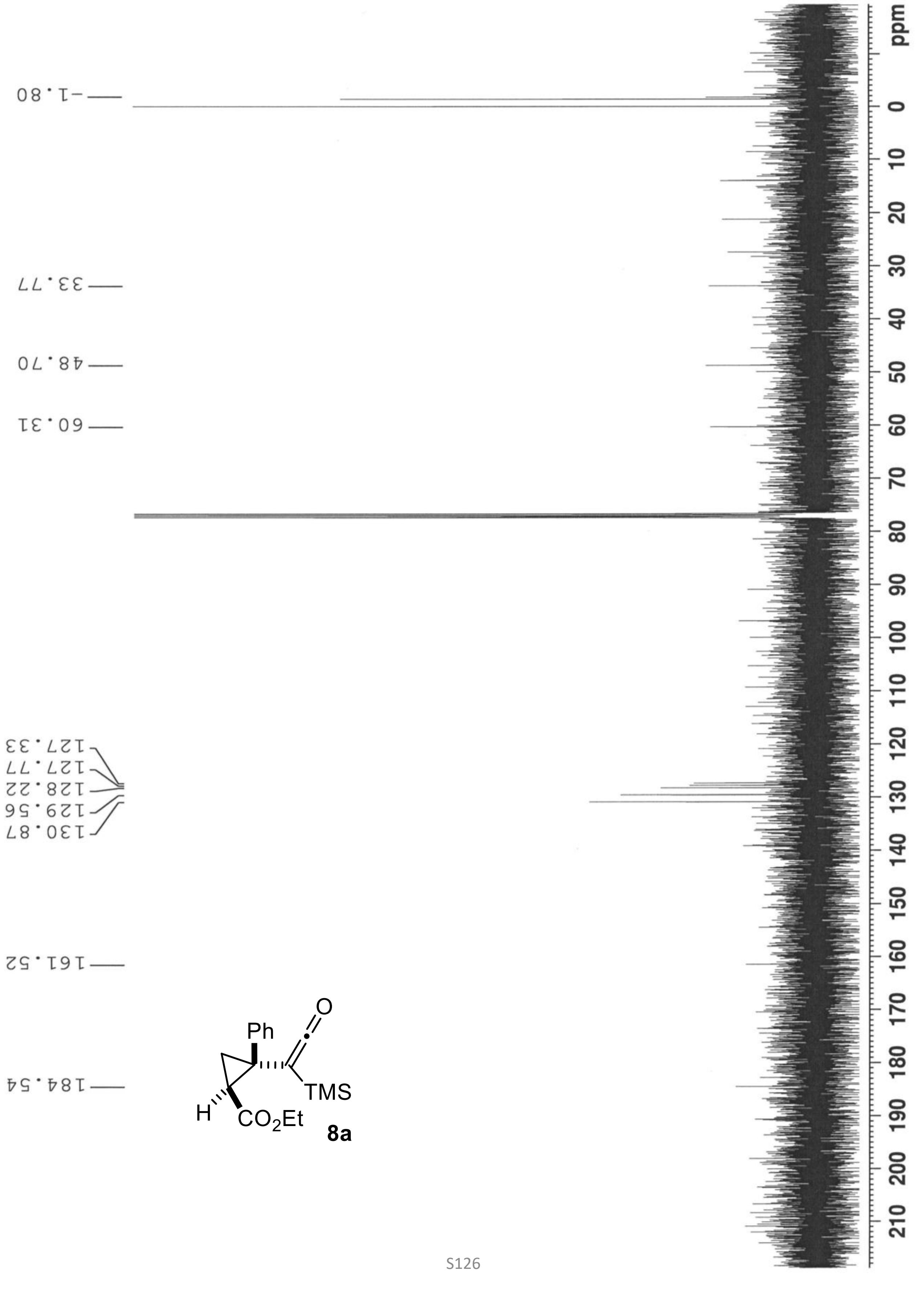

-1.80
33.77
48.70
60.31
127.33
127.77
128.22
129.56
130.87
161.52
184.54
ppm
0
10
20
30
40
50
60
70
80
90
100
110
120
130
140
150
160
170
180
190
200
210
O
Ph
TMS
H
CO_2Et

8a

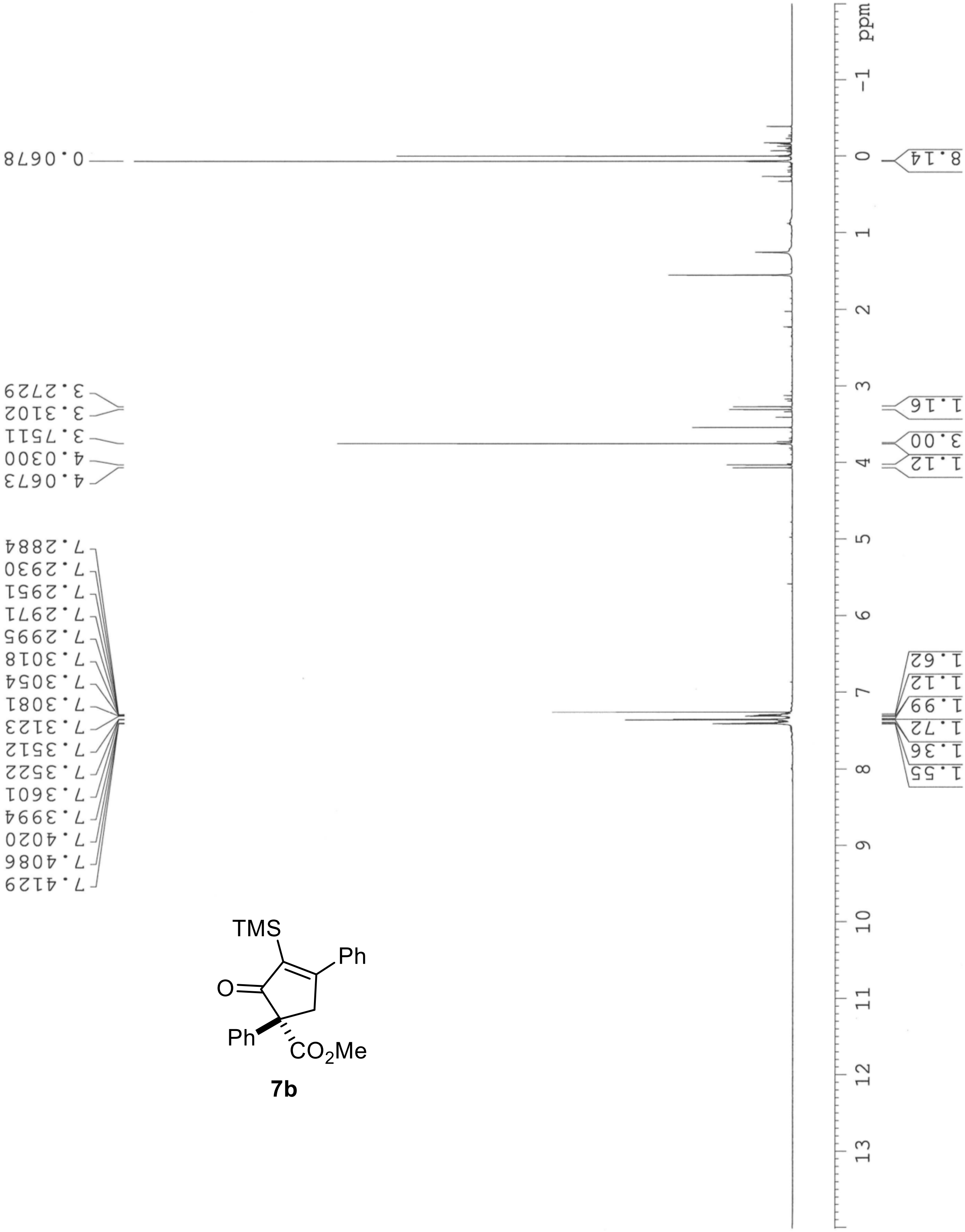
0.0678
3.2729
3.3102
3.7511
4.0300
4.0673
7.2884
7.2930
7.2951
7.2971
7.2995
7.3018
7.3054
7.3081
7.3123
7.3512
7.3522
7.3601
7.3994
7.4020
7.4086
7.4129
8.14
1.16
3.00
1.12
1.62
1.12
1.99
1.72
1.36
1.55
ppm
TMS
Ph
O
Ph
CO2Me

7b

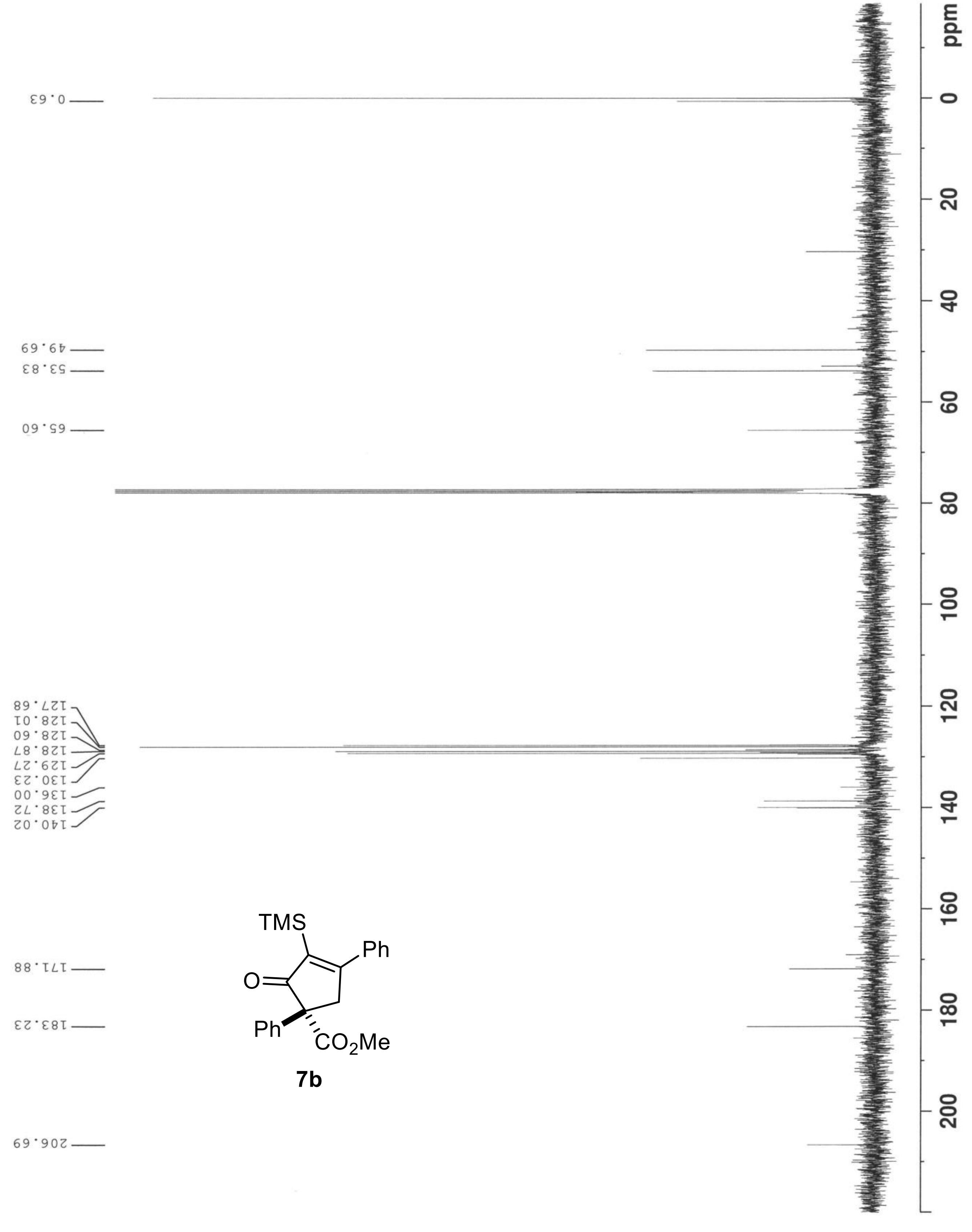
0.63
49.69
53.83
65.60
127.68
128.01
128.60
128.87
129.27
130.23
136.00
138.72
140.02
171.88
183.23
206.69
ppm
0
20
40
60
80
100
120
140
160
180
200
TMS
Ph
O
Ph
CO_2Me

7b

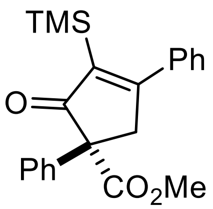

7b

Racemic

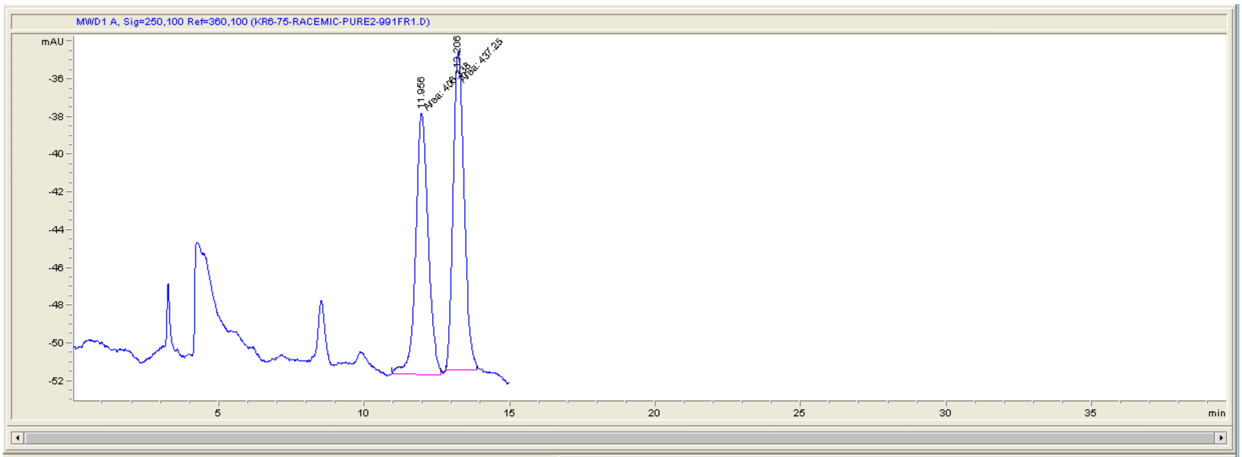

File Information

LC-File	KR6-75-RACEMIC-PURE2-991FR1.D
File Path	C:\CHEM32\2\DATA\ERIN\

#	Time	Area	Height	Width	Area%	Symmetry
1	11.956	406.3	13.9	0.4878	48.168	0.879
2	13.206	437.2	16.9	0.4324	51.832	0.774

Rh_2(*S*-TCPTTL)$_4$: 54% ee

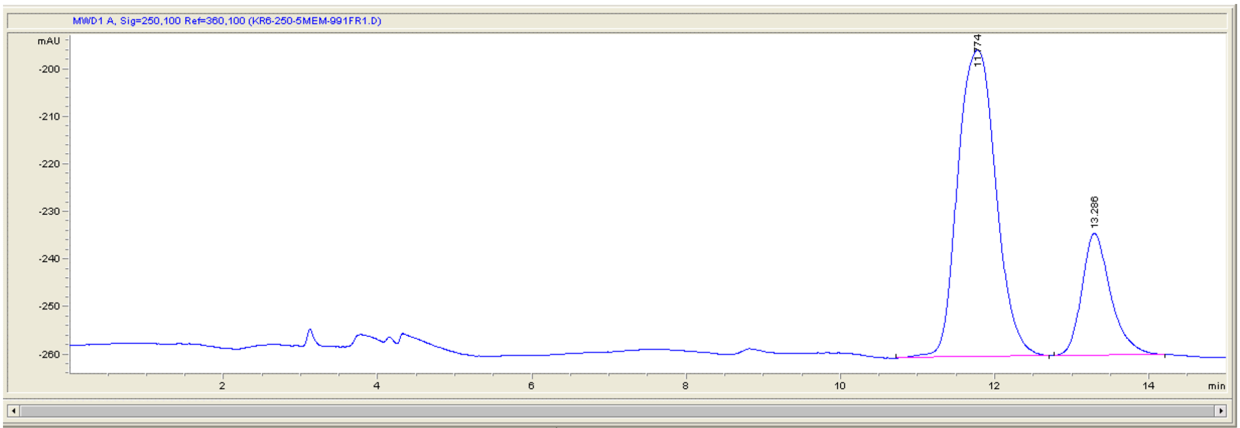

File Information

LC-File	KR6-250-5MEM-991FR1.D
File Path	C:\CHEM32\2\DATA\ERIN\

#	Time	Area	Height	Width	Area%	Symmetry
1	11.774	2230.8	64.6	0.5471	76.713	1.01
2	13.286	677.2	25.7	0.3968	23.287	0.742

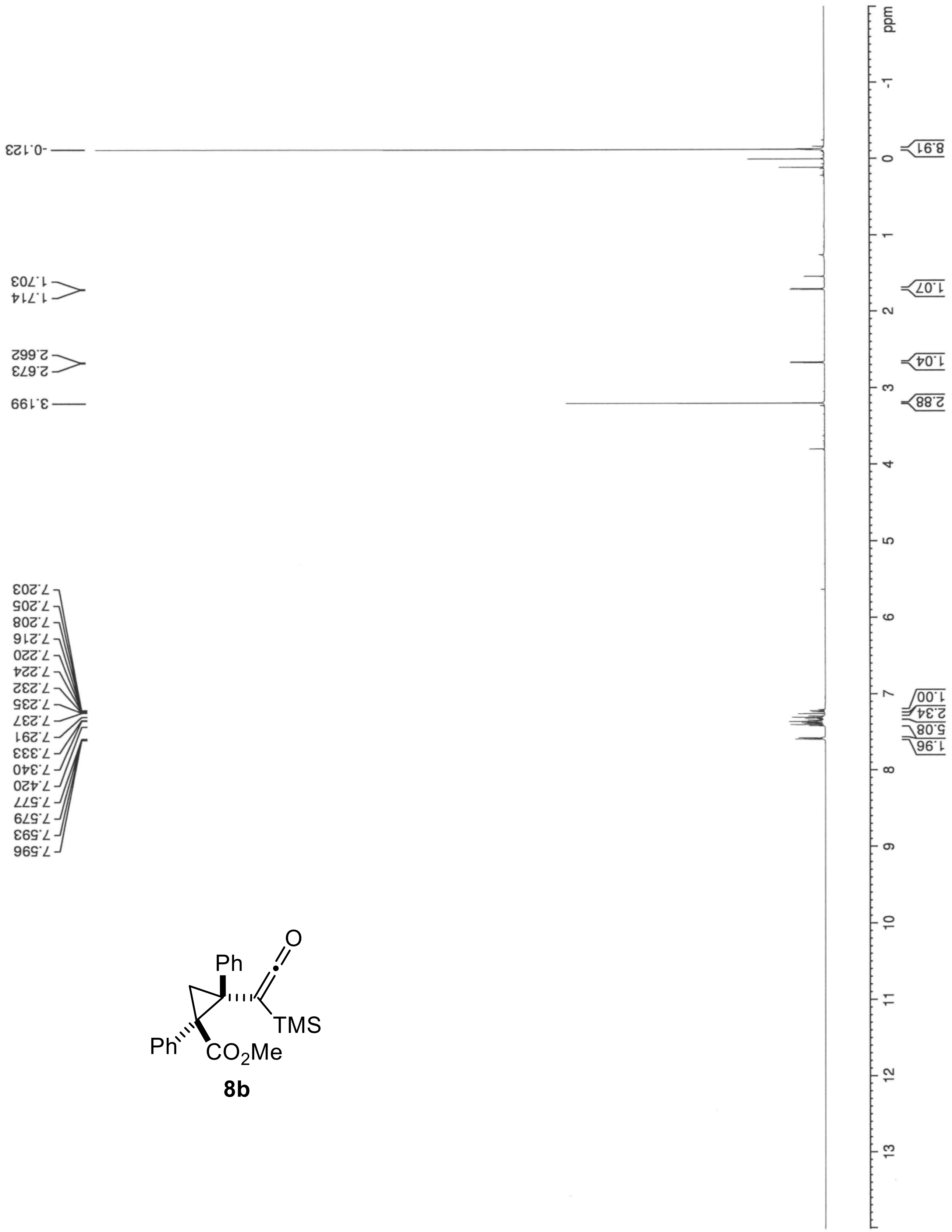
-0.123
1.703
1.714
2.662
2.673
3.199
7.203
7.205
7.208
7.216
7.220
7.224
7.232
7.235
7.237
7.291
7.333
7.340
7.420
7.577
7.579
7.593
7.596
8.91
1.07
1.04
2.88
1.00
2.34
5.08
1.96
ppm
Ph
O
TMS
Ph
CO_2Me

8b

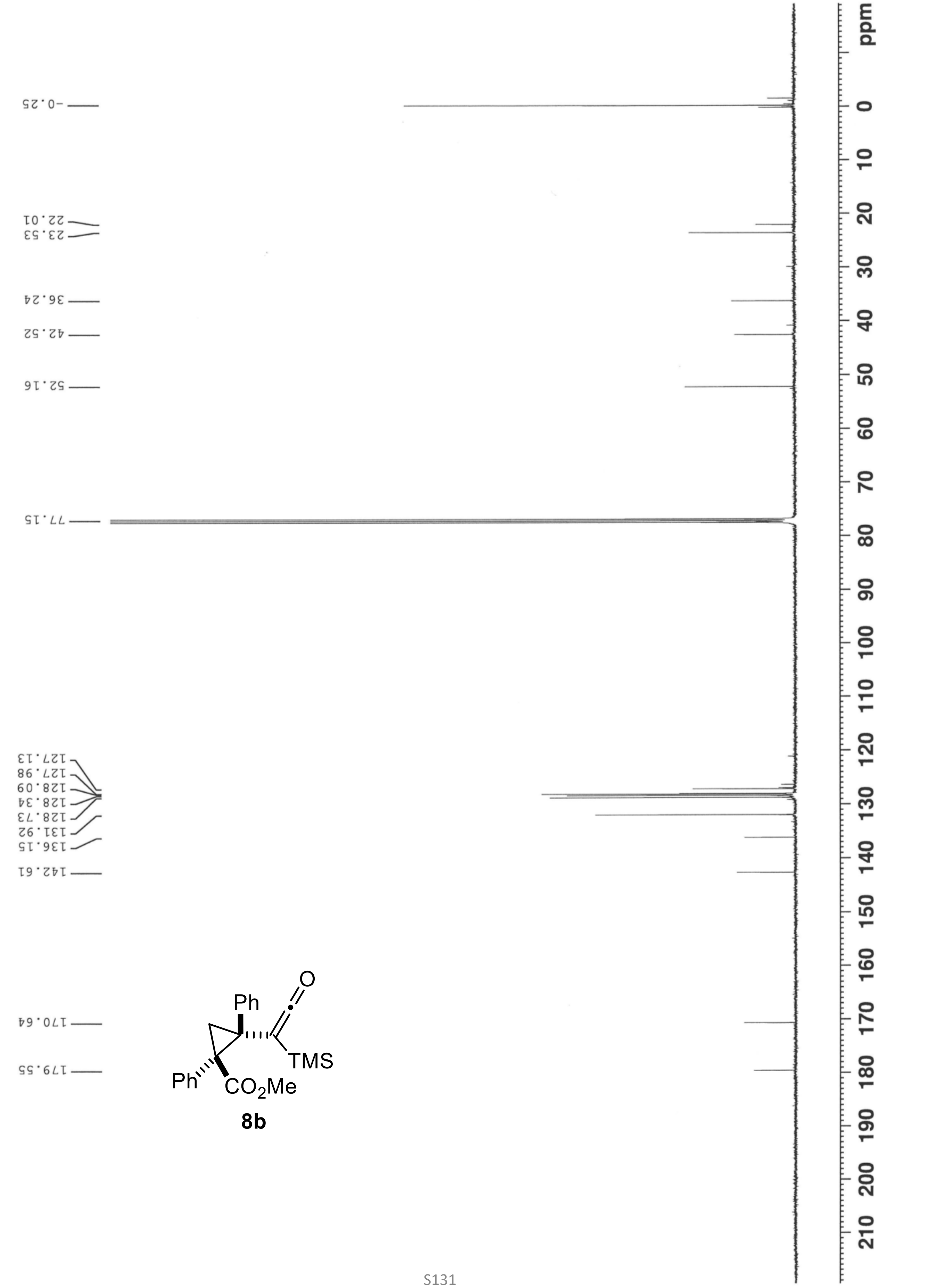
-0.25
22.01
23.53
36.24
42.52
52.16
77.15
127.13
127.98
128.09
128.34
128.73
131.92
136.15
142.61
170.64
179.55
O
Ph
TMS
Ph
CO2Me
8b
ppm

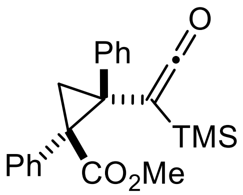

8b

Racemic

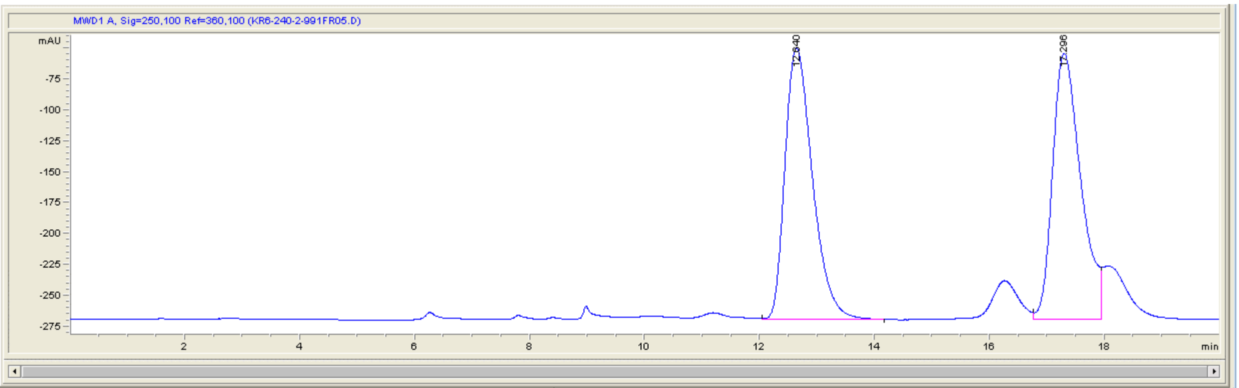

File Information

LC-File	KR6-240-2-991FR05.D
File Path	C:\CHEM32\2\DATA\ERIN\
Date	24-Oct-17, 21:10:17

#	Time	Area	Height	Width	Area%	Symmetry
1	12.64	7337.8	219.5	0.514	49.864	0.671
2	17.296	7377.8	215	0.5243	50.136	0.648

Rh_2(*S*-TCPTTL)$_4$: 52% ee

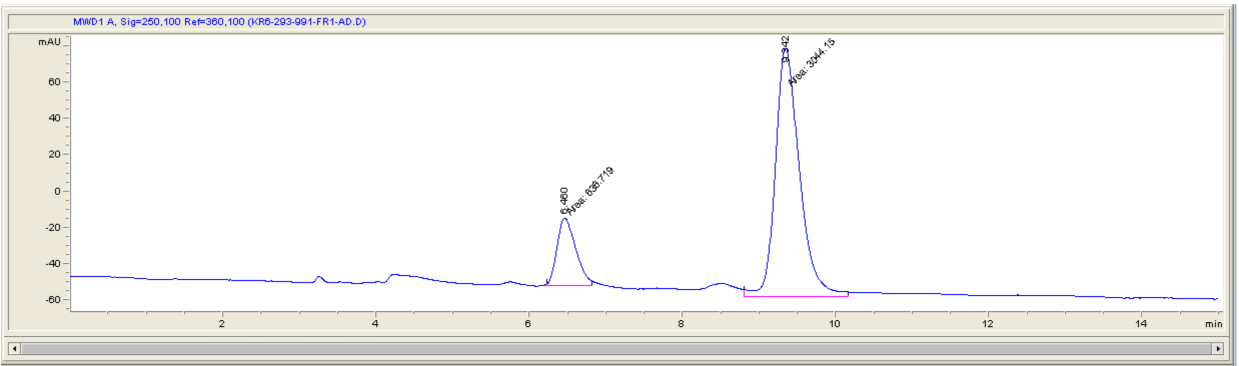

File Information

LC-File	KR6-293-991-FR1-AD.D
File Path	C:\CHEM32\2\DATA\ERIN\
Date	15-Dec-17, 17:52:18

#	Time	Area	Height	Width	Area%	Symmetry
1	6.46	636.7	37.4	0.2837	17.298	0.69
2	9.342	3044.2	137.5	0.3691	82.702	0.728

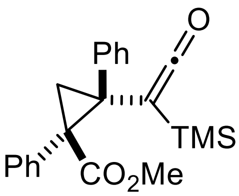

8b

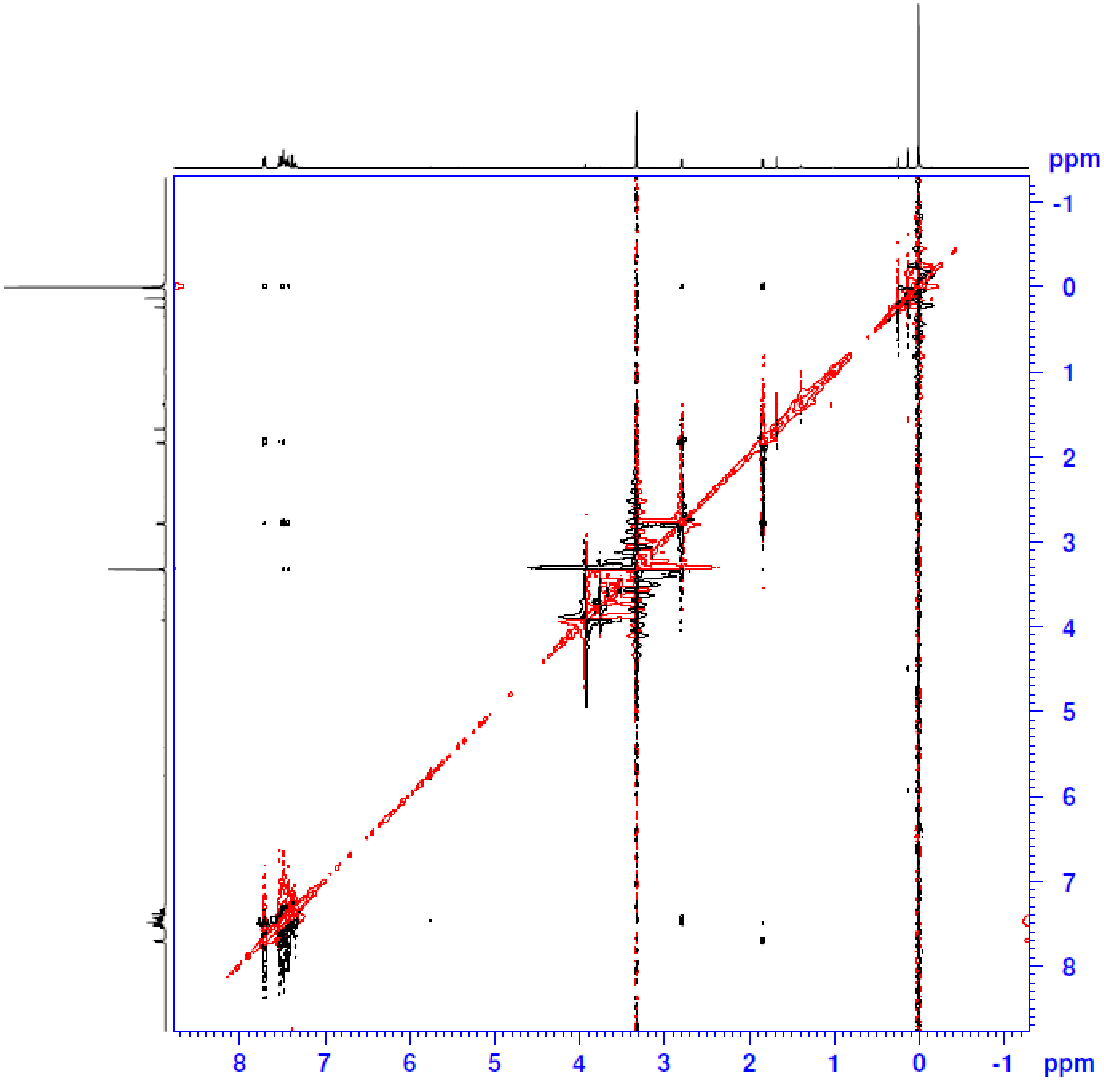

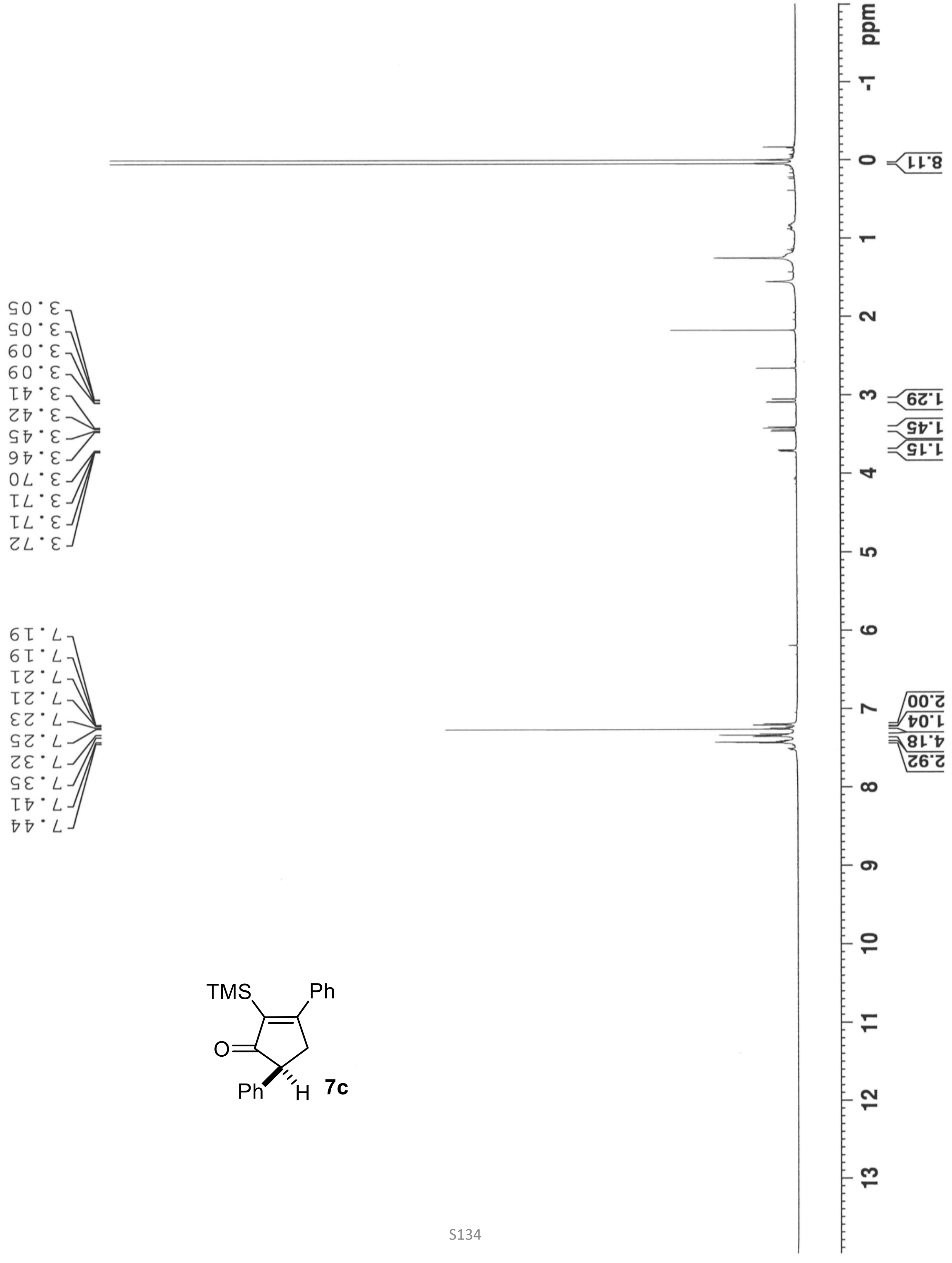
ppm
-1
0
1
2
3
4
5
6
7
8
9
10
11
12
13
8.11
1.29
1.45
1.15
2.00
1.04
4.18
2.92
3.05
3.05
3.09
3.09
3.41
3.42
3.45
3.46
3.70
3.71
3.71
3.72
7.19
7.19
7.21
7.21
7.23
7.25
7.32
7.35
7.41
7.44
TMS
Ph
O
Ph
H
7c

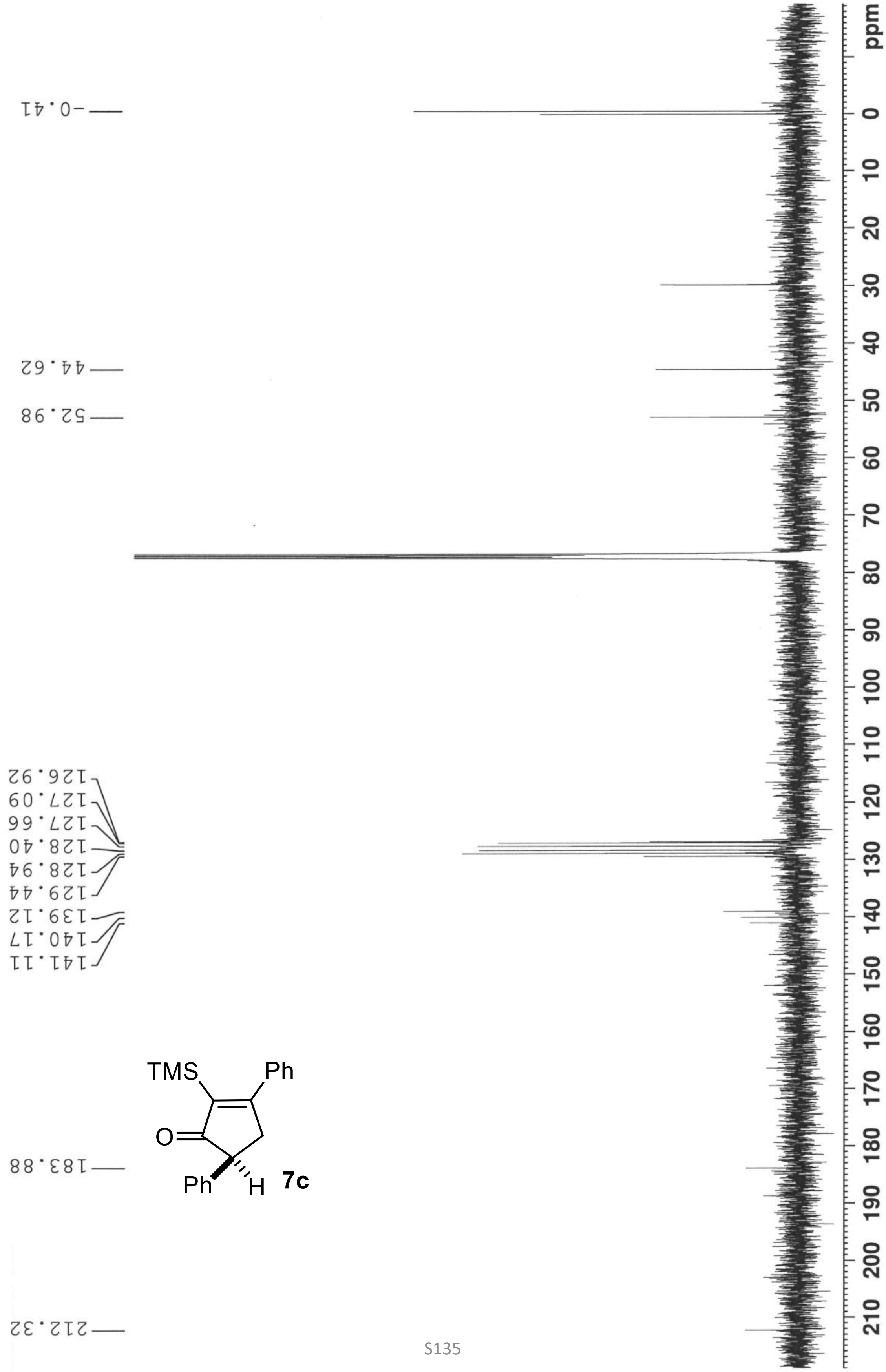
-0.41
44.62
52.98
126.92
127.09
127.66
128.40
128.94
129.44
139.12
140.17
141.11
183.88
212.32
TMS
Ph
O
Ph
H
7c
ppm

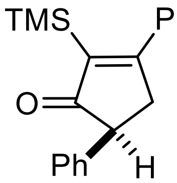

7c

Racemic

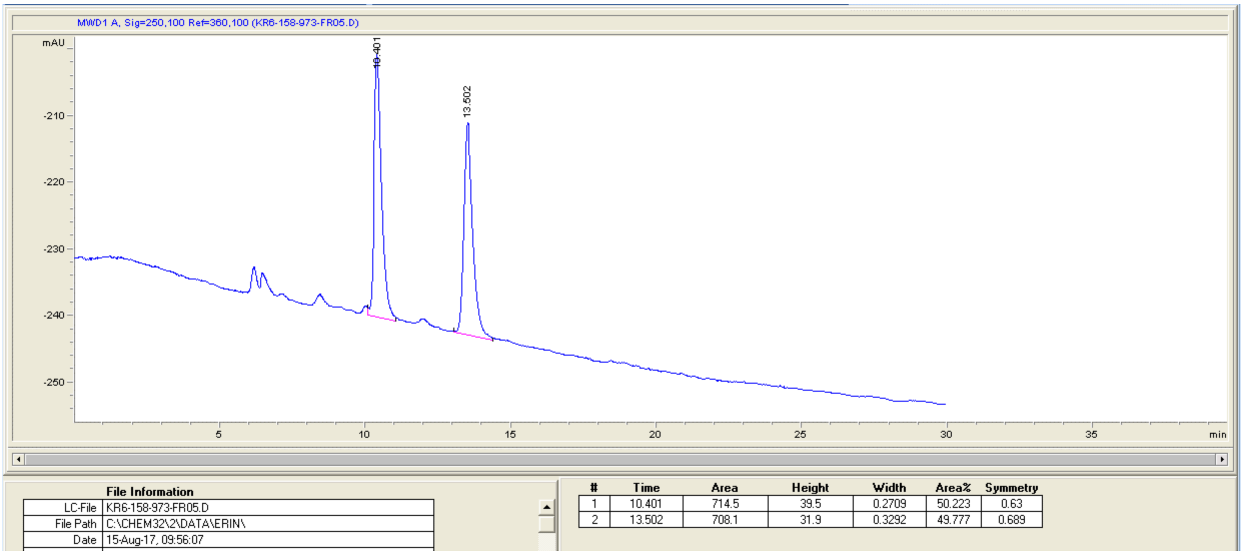

Rh_2(*S*-TCPTTL)$_4$: 82% ee

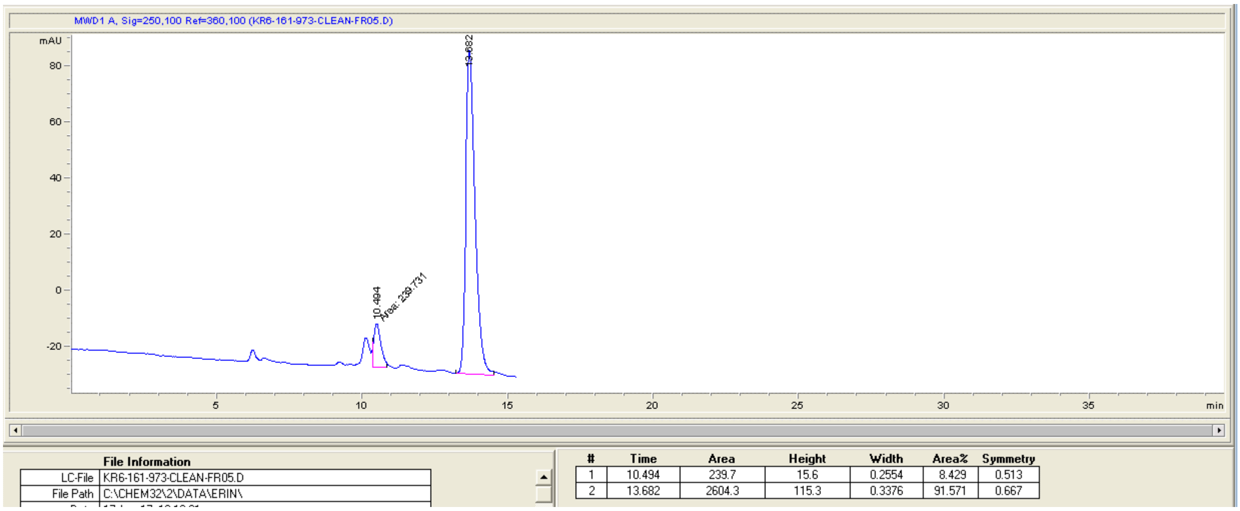

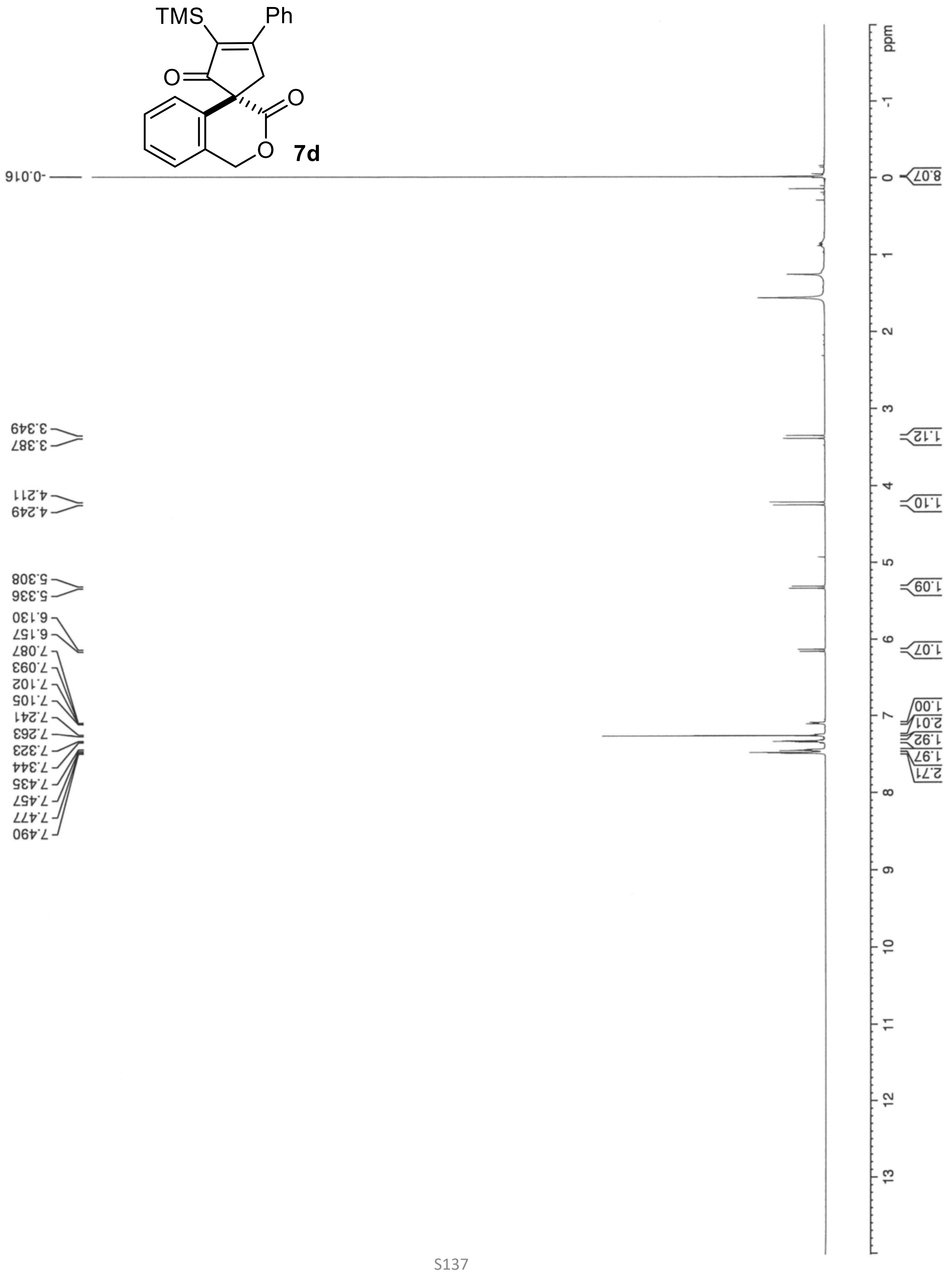
TMS
Ph
O
O
O
7d
-0.016
3.349
3.387
4.211
4.249
5.308
5.336
6.130
6.157
7.087
7.093
7.102
7.105
7.241
7.263
7.323
7.344
7.435
7.457
7.477
7.490
8.07
1.12
1.10
1.09
1.07
1.00
2.01
1.92
1.97
2.71
ppm

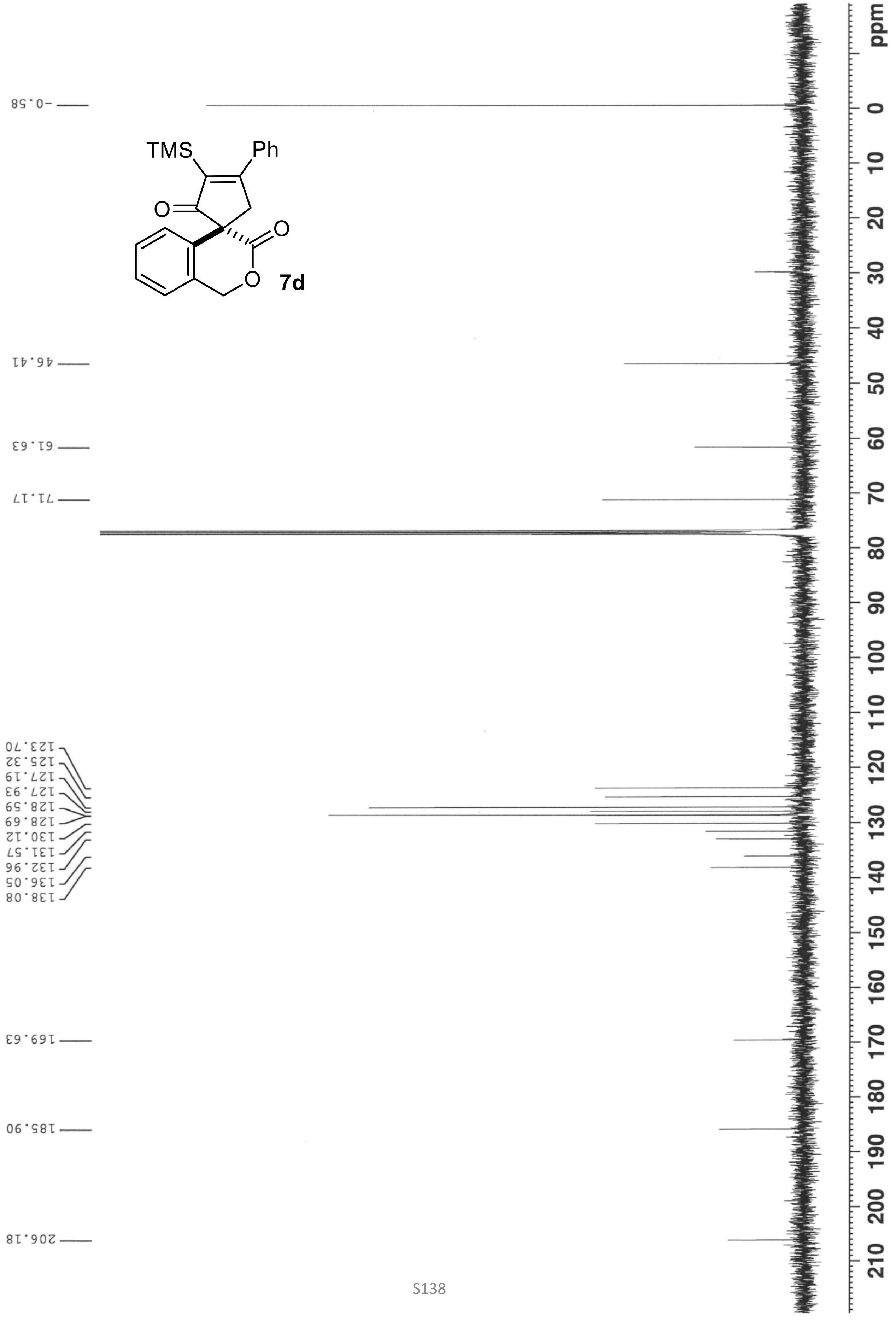
-0.58
46.41
61.63
71.17
123.70
125.32
127.19
127.93
128.59
128.69
130.12
131.57
132.96
136.05
138.08
169.63
185.90
206.18
TMS
Ph
O
O
O
7d
ppm

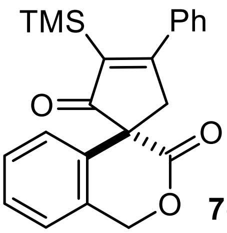

7d

Racemic

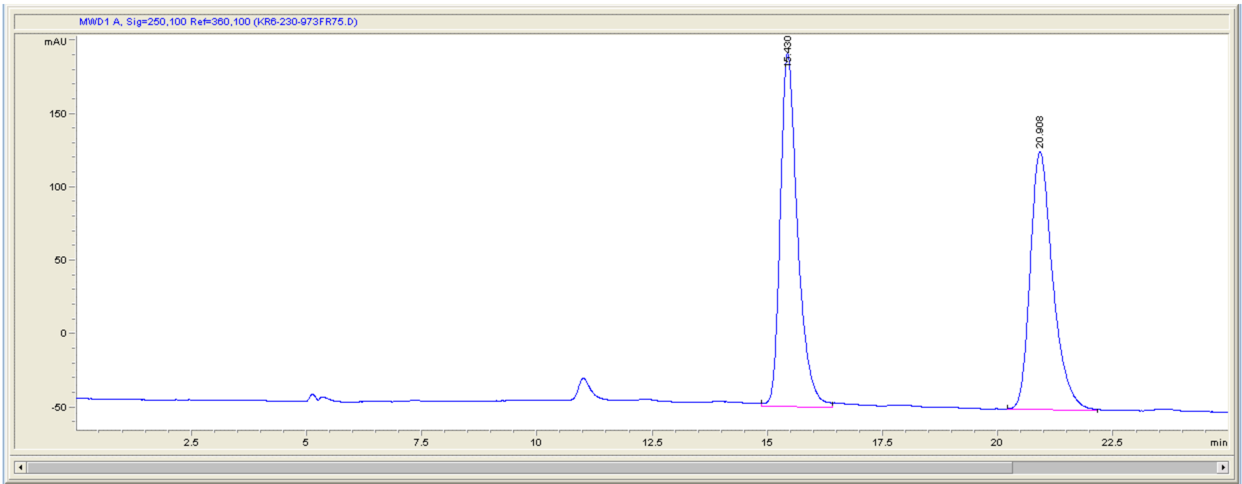

File Information

LC-File	KR6-230-973FR75.D
File Path	C:\CHEM32\2\DATA\ERIN\

#	Time	Area	Height	Width	Area%	Symmetry
1	15.43	6358.7	241	0.3999	51.277	0.673
2	20.908	6041.8	175.9	0.5106	48.723	0.712

Rh_2(*S*-TCPTTL)$_4$: 86% ee

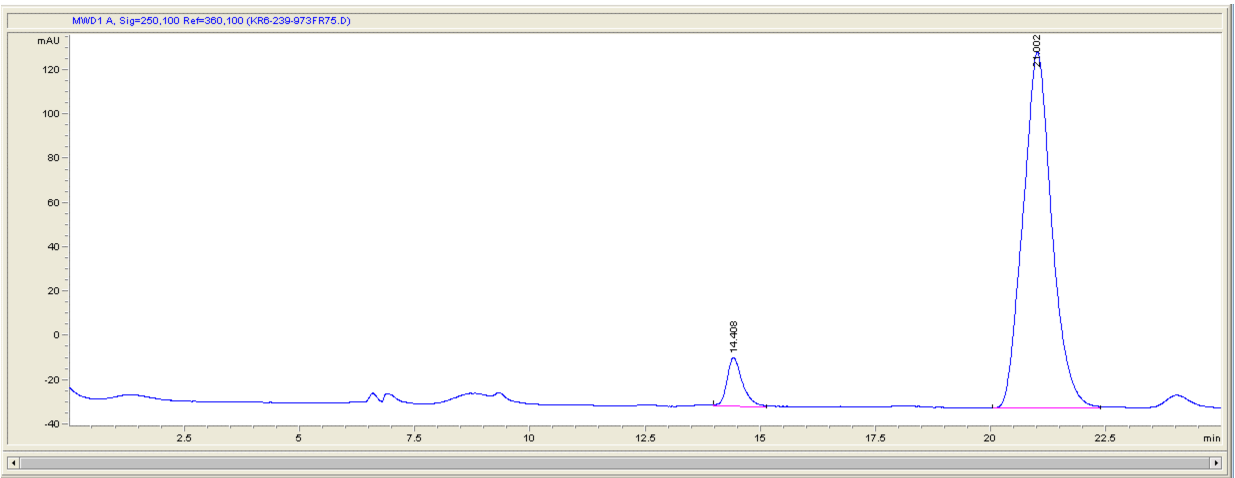

File Information

LC-File	KR6-239-973FR75.D
File Path	C:\CHEM32\2\DATA\ERIN\

#	Time	Area	Height	Width	Area%	Symmetry
1	14.408	526.4	22.2	0.3542	6.987	0.682
2	21.002	7008	160.7	0.6167	93.013	0.937

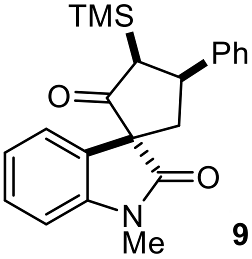
TMS
Ph
O
O
N
Me

9

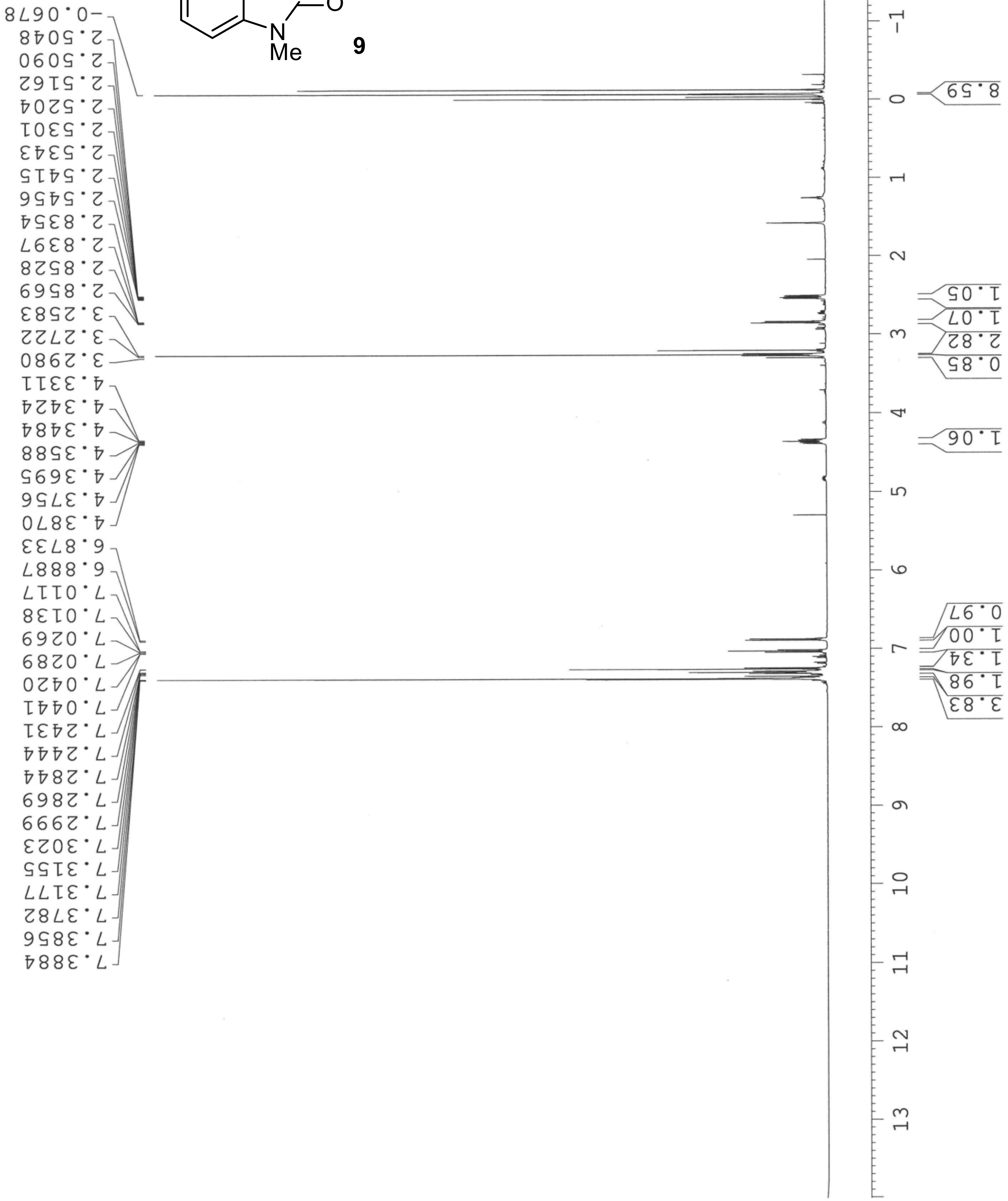
-0.0678
2.5048
2.5090
2.5162
2.5204
2.5301
2.5343
2.5415
2.5456
2.8354
2.8397
2.8528
2.8569
3.2583
3.2722
3.2980
4.3311
4.3424
4.3484
4.3588
4.3695
4.3756
4.3870
6.8733
6.8887
7.0117
7.0138
7.0269
7.0289
7.0420
7.0441
7.2431
7.2444
7.2844
7.2869
7.2999
7.3023
7.3155
7.3177
7.3782
7.3856
7.3884
8.59
1.05
1.07
2.82
0.85
1.06
0.97
1.00
1.34
1.98
3.83
ppm

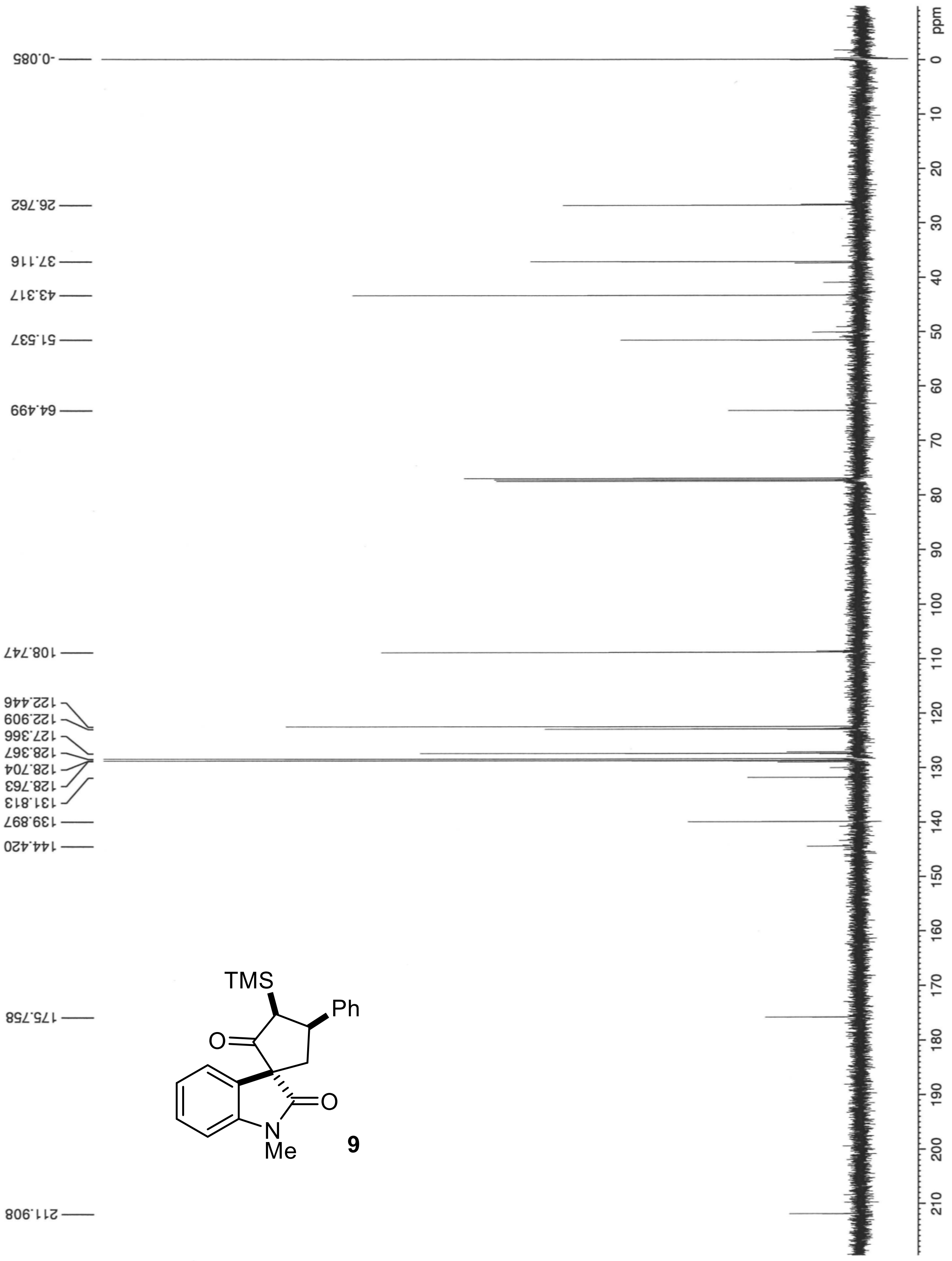
-0.085
26.762
37.116
43.317
51.537
64.499
108.747
122.446
122.909
127.366
128.367
128.704
128.763
131.813
139.897
144.420
175.758
211.908
ppm
TMS
Ph
O
N
Me
9

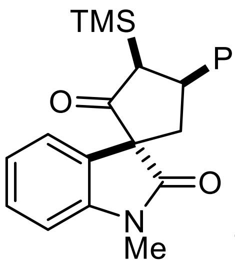

9

Racemic

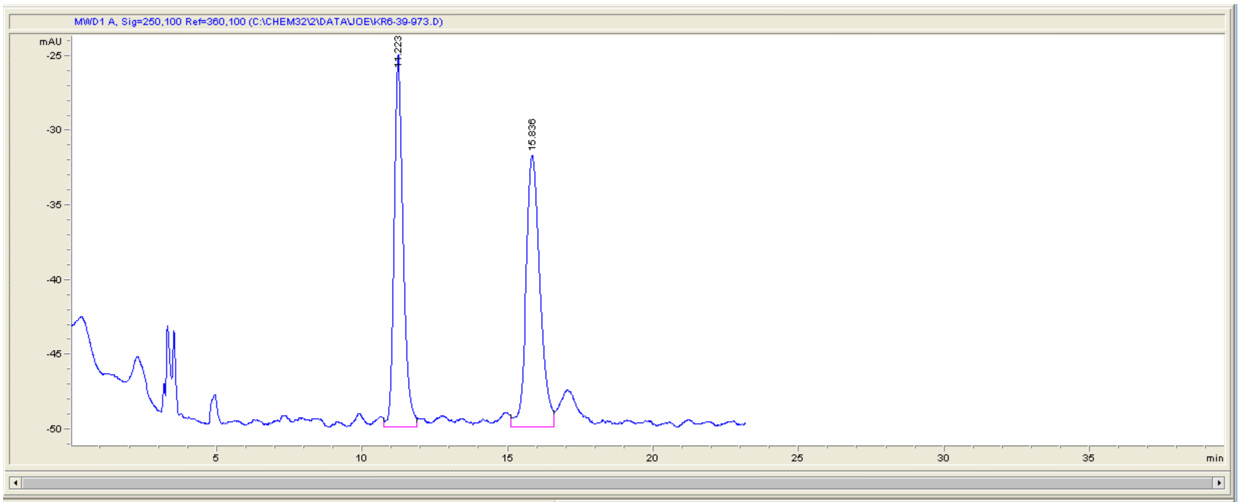

File Information

LC-File	KR6-39-973.D
File Path	C:\CHEM32\2\DATA\JOE\

#	Time	Area	Height	Width	Area%	Symmetry
1	11.223	568	25	0.3474	48.527	0.78
2	15.836	602.5	18.2	0.4684	51.473	0.791

Rh_2(*S*-TCPTTL)$_4$: 99% ee

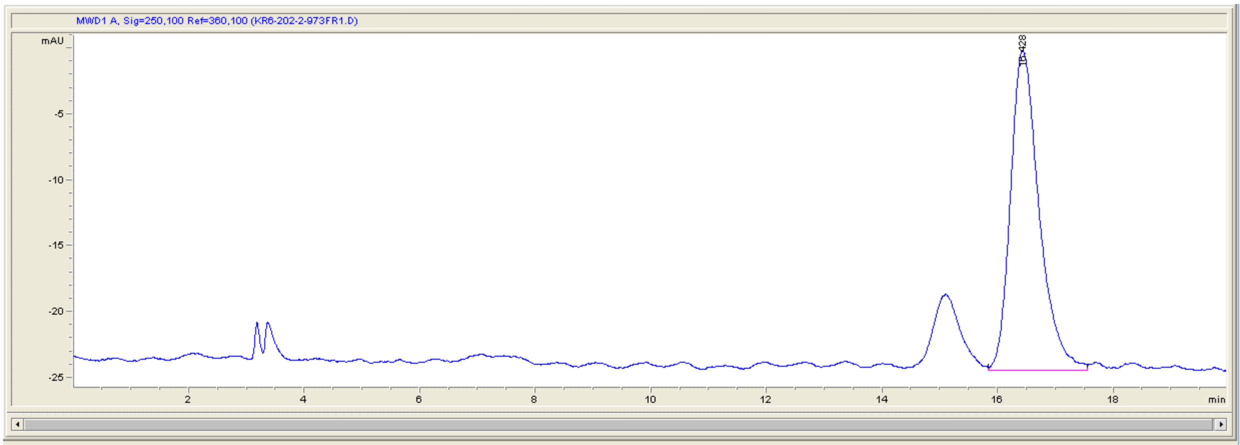

File Information

LC-File	KR6-202-2-973FR1.D
File Path	C:\CHEM32\2\DATA\ERIN\

#	Time	Area	Height	Width	Area%	Symmetry
1	16.428	841.9	24.3	0.4961	100.000	0.675

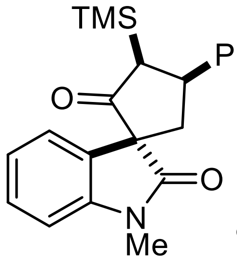

9

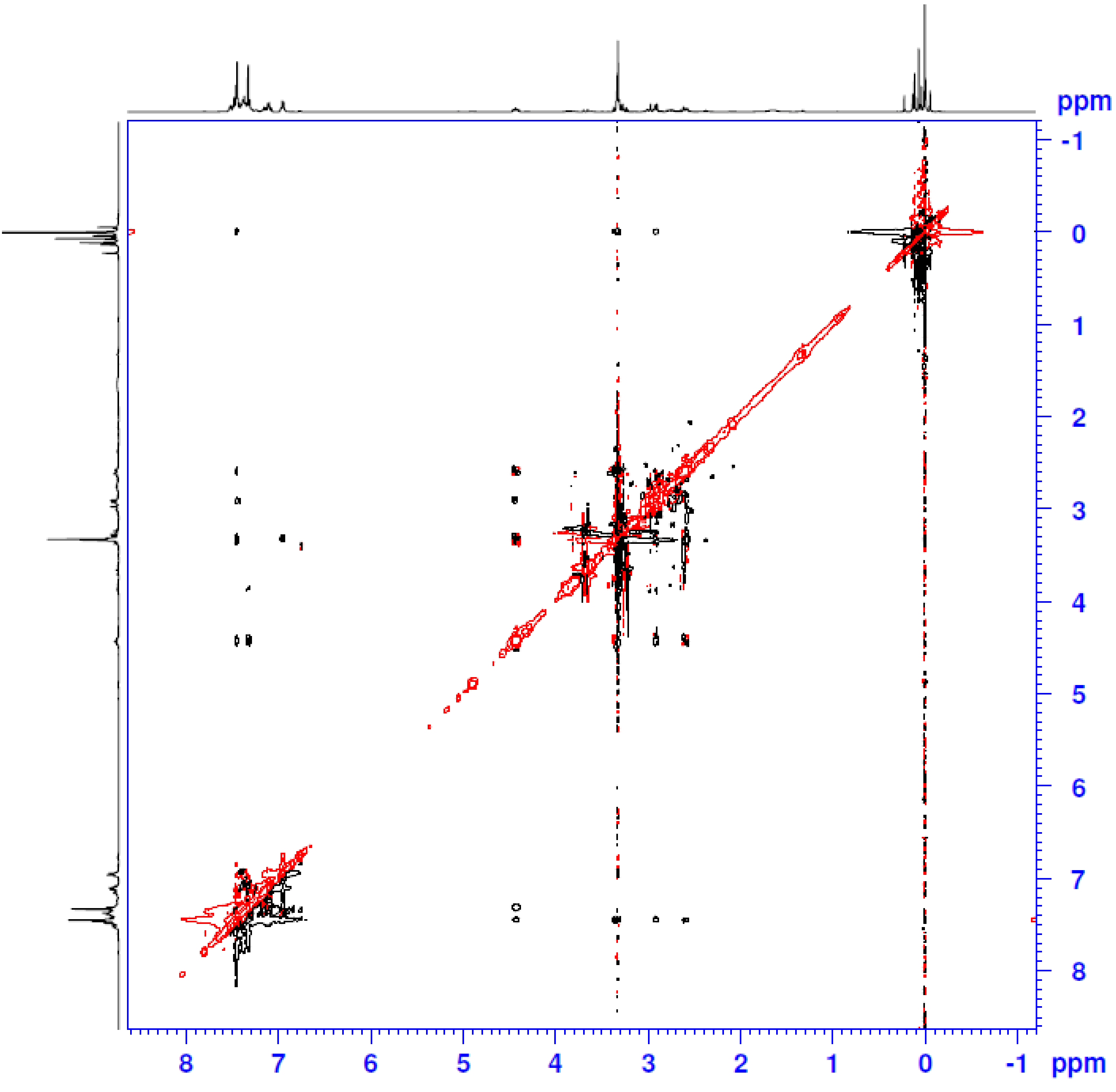

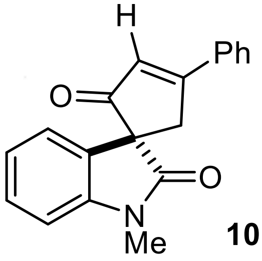

10

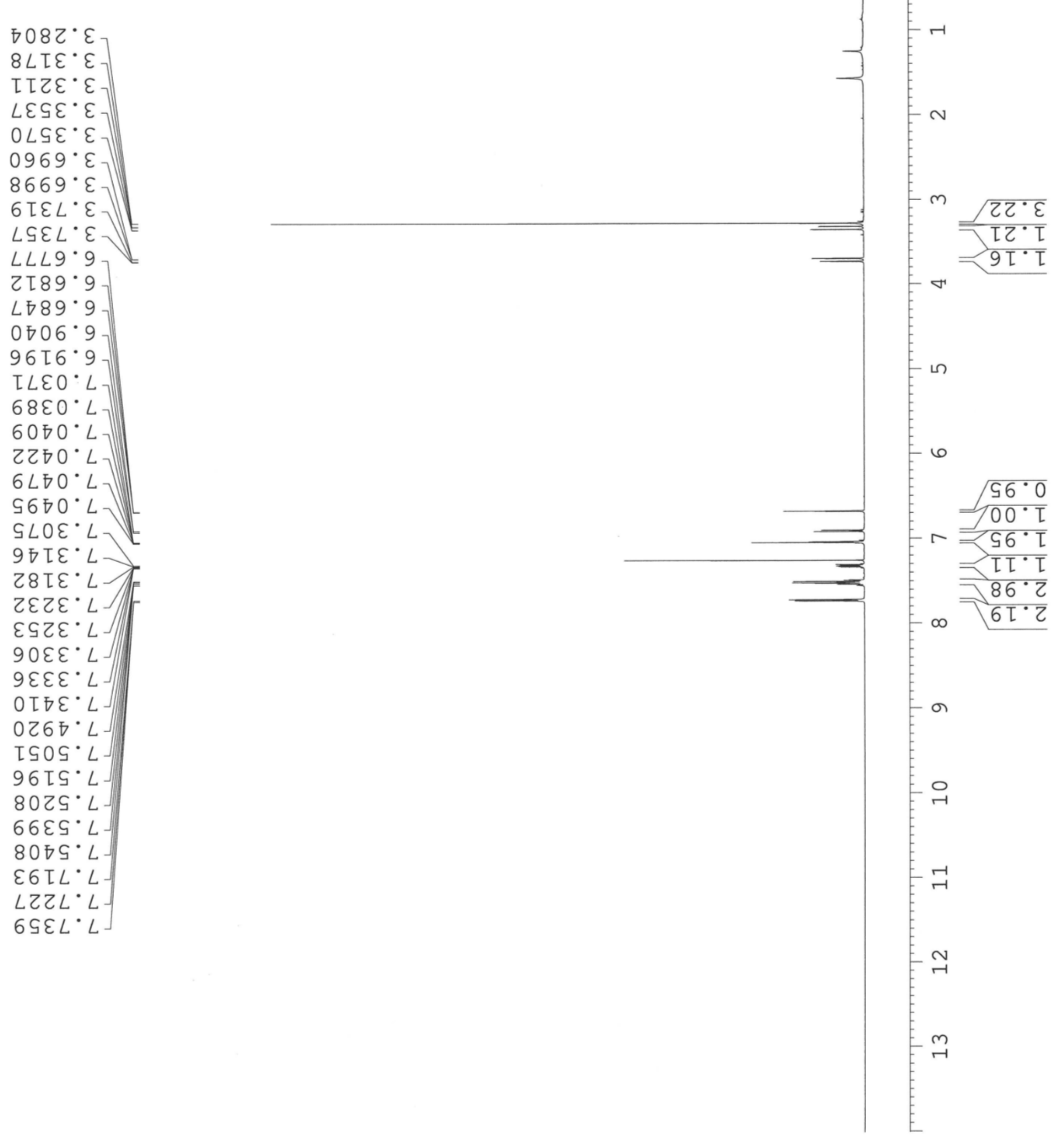
3.2804
3.3178
3.3211
3.3537
3.3570
3.6960
3.6998
3.7316
3.7357
6.6777
6.6812
6.6847
6.9040
6.9196
7.0371
7.0389
7.0409
7.0422
7.0479
7.0495
7.3075
7.3146
7.3182
7.3232
7.3253
7.3306
7.3336
7.3410
7.4920
7.5051
7.5196
7.5208
7.5399
7.5408
7.7193
7.7227
7.7359
3.22
1.21
1.16
0.95
1.00
1.95
1.11
2.98
2.19
ppm
-1
0
1
2
3
4
5
6
7
8
9
10
11
12
13

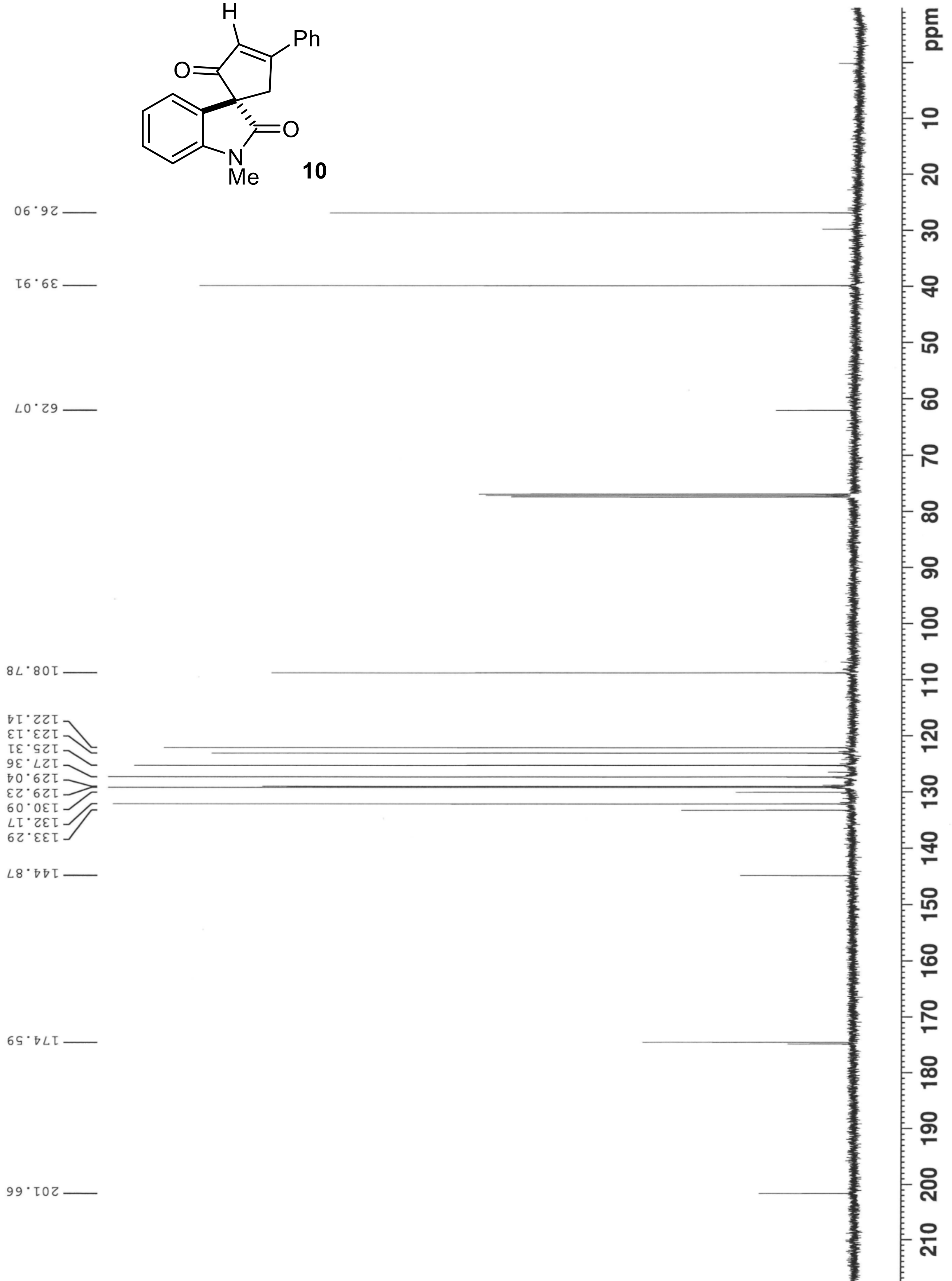
H
Ph
O
O
N
Me
10
26.90
39.91
62.07
108.78
122.14
123.13
125.31
127.36
129.04
129.23
130.09
132.17
133.29
144.87
174.59
201.66
ppm
10
20
30
40
50
60
70
80
90
100
110
120
130
140
150
160
170
180
190
200
210

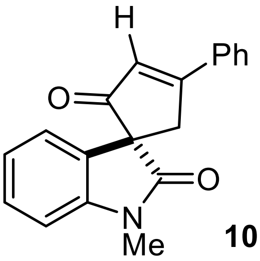

10

Racemic

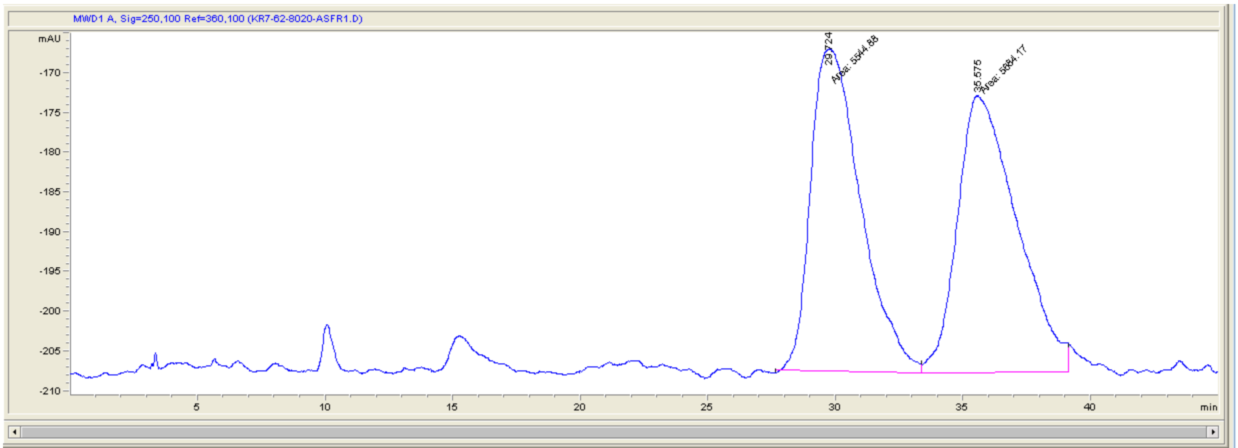

File Information	
LC-File	KR7-62-8020-ASFR1.D
File Path	C:\CHEM32\2\DATA\ERIN\
Date	07-Feb-18, 17:23:51

#	Time	Area	Height	Width	Area%	Symmetry
1	29.724	5544.9	40.5	2.2794	49.380	0.558
2	35.575	5684.2	34.8	2.7186	50.620	0.534

Rh_2(*S*-TCPTTL)$_4$: 99% ee

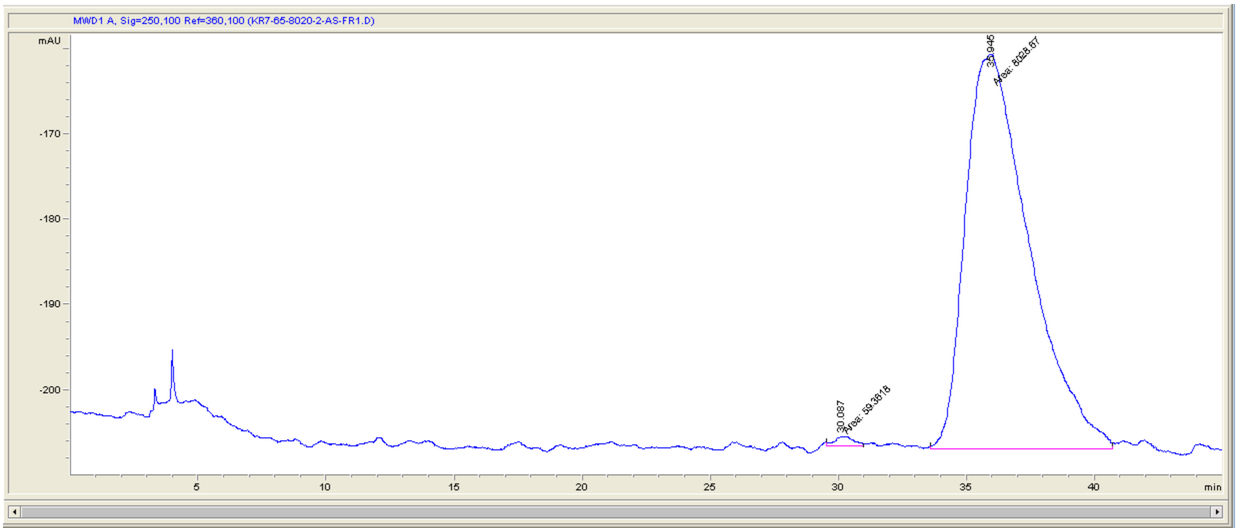

File Information	
LC-File	KR7-65-8020-2-AS-FR1.D
File Path	C:\CHEM32\2\DATA\ERIN\
Date	07-Feb-18, 16:12:41

#	Time	Area	Height	Width	Area%	Symmetry
1	30.087	59.4	1.1	0.8726	0.734	0.543
2	35.945	8028.7	46.2	2.8937	99.266	0.634
